# Cytoplasmic- and extracellular-proteome analysis of *Diplodia seriata*: a phytopathogenic fungus involved in grapevine decline

**DOI:** 10.1186/1477-5956-8-46

**Published:** 2010-09-09

**Authors:** Rebeca Cobos, Carlos Barreiro, Rosa María Mateos, Juan-José R Coque

**Affiliations:** 1 Área de Microbiología, Departamento de Biología Molecular, Universidad de León, 24071-León, Spain; 2 Instituto de Investigación de la Viña y el Vino, Campus de Ponferrada, Universidad de León, 24400-Ponferrada, Spain; 3 Instituto de Biotecnología de León (INBIOTEC), Parque Científico de León, Avenida Real 1, 24006-León, Spain

## Abstract

**Background:**

The phytopathogenic fungus *Diplodia seriata*, whose genome remains unsequenced, produces severe infections in fruit trees (fruit blight) and grapevines. In this crop is recognized as one of the most prominent pathogens involved in grapevine trunk disease (or grapevine decline). This pathology can result in the death of adult plants and therefore it produces severe economical losses all around the world. To date no genes or proteins have been characterized in *D. seriata *that are involved in the pathogenicity process. In an effort to help identify potential gene products associated with pathogenicity and to gain a better understanding of the biology of *D. seriata*, we initiated a proteome-level study of the fungal mycelia and secretome.

**Results:**

Intracellular and secreted proteins from *D. seriata *collected from liquid cultures were separated using two-dimensional gel electrophoresis. About 550 cytoplasmic proteins were reproducibly present in 3 independent extractions, being 53 identified by peptide mass fingerprinting and tandem mass spectrometry. The secretome analysis showed 75 secreted proteins reproducibly present in 3 biological replicates, being 16 identified. Several of the proteins had been previously identified as virulence factors in other fungal strains, although their contribution to pathogenicity in *D. seriata *remained to be analyzed. When *D. seriata *was grown in a medium supplemented with carboxymethylcellulose, 3 proteins were up-regulated and 30 down-regulated. Within the up-regulated proteins, two were identified as alcohol dehydrogenase and mitochondrial peroxyrredoxin-1, suggesting that they could play a significant role in the pathogenicity process. As for the 30 down-regulated proteins, 9 were identified being several of them involved in carbohydrate metabolism.

**Conclusions:**

This study is the first report on proteomics on *D. seriata*. The proteomic data obtained will be important to understand the pathogenicity process. In fact, several of the identified proteins have been reported as pathogenicity factors in other phytopathogenic fungi. Moreover, this proteomic analysis supposes a useful basis for deepening into *D. seriata *knowledge and will contribute to the development of the molecular biology of this fungal strain as it has been demonstrated by cloning the gene *Prx*1 encoding mitochondrial peroxiredoxin-1 of *D. seriata *(the first gene to be cloned in this microorganism; data not shown).

## Background

The fungal family Botryosphaeriaceae (Botryosphaeriales, Ascomycetes) is a complex family that, according to a recent broad-based phylogenetic study, includes ten genera [1]. The majority of these genera comprise members described as fungal endophytes, present in virtually all woody host examined (both gymnosperm and angiosperm species) [1,2], and being the dominating endophyte communities in *Eucalyptus *and *Pinus *in some environments [3]. One of the most important members of this family is *Diplodia *(which includes teleomorphs distributed in several genera). *Diplodia seriata *(before named as *Botryosphaeria obtusa*) is an important phytopathogenic fungus that has been isolated from at least 34 different hosts, causing severe infections in fruit trees (mainly pomes and stone fruits) [4]. Also recently, *D. seriata *has been described as an important pathogen of grapevines (*Vitis vinifera*), involved in grapevine trunk disease causing the death of adult plants [5]. Grapevine decline, or grapevine trunk disease, is currently recognized as the main threat to vineyards all around the world since no effective treatment to fight this fungal pathology is presently available [6,7]. *D. seriata *can be isolated from grapevines exhibiting dieback of shoots, spurs and branches, as well as severe internal wood necrosis symptoms [8]. It is also a cosmopolitan grapevine pathogen which has been detected all around the world [9]. Its contribution to grapevine decline is of major importance since it has been the most frequently isolated pathogen in an extensive survey on affected grapevine plants carried out in Spain. In fact, 63% of the total *Botryosphaeria*-like species isolated from young plants and 28% of the pathogenic isolates from adult plants were identified as *D. seriata*, which represent around 36% of the total isolated pathogen fungi associated to grapevine decline [10]. Despite the importance of *D. seriata *as phytopathogen, little is known about its biological life cycle, its basic molecular aspects and the mechanisms involved in the pathogenicity of this fungal species. Botryosphaeriaceae species are known to produce phytotoxic compounds like exopolysaccharides and other toxic organic compounds which induce phytotoxic effects on plants [11,12], but unfortunately no *D. seriata *pathogenicity-related genes has been deposited yet in databases.

Proteomic studies are one of the most powerful methods to evaluate the final result of gene expression. In the present case two-dimensional gel electrophoresis (2-DE) has been the technique of choice to obtain a referential global picture of the cytoplasmic and extracellular protein maps. Up to now, this technique has been successfully applied to analyze not only the proteome and/or secretome of many different fungal strains [13,14], but also plant-pathogen interactions, including analyses of several phytopathogenic fungi [15-17]. Regarding phytopathogenicity, the phylum Ascomycota comprises highly appreciated species in food and pharmaceutical industries, but also others dreaded as phytopathogens due to their secreted proteins [18]. However, the understanding of the molecular basis of the secretion process in these microorganisms is still very limited. As reviewed above, a considerable number of proteomic analyses have been carried out in fungal species in the last years, mainly concerning strains of medical or agricultural interest. Nevertheless, to our knowledge, no proteomic data have been reported yet for *D. seriata*.

Thus, the main goal of this manuscript is the achievement of an initial approach to define the *D. seriata *proteome and secretome. Protocols for protein extraction from mycelium, protein separation by 2-DE and mass spectrometry (MS) analyses have been optimized. Furthermore, proteome differences have also been tested by growing the strain in the presence of carboxymethylcellulose (CMC). Since cellulose is one of the major components of the plant cell wall, proteins overexpressed during growth on CMC could be expected to play a significant role in the pathogenicity process. In fact, this strategy has been used to identify putative pathogenicity factors in the phytopathogenic fungus *Botrytis cinerea *[19,20]. We decided to try this strategy since *D. seriata *is an endophytic fungi that exerts its pathogenicity by developing inside the vascular system of grapevine, and unfortunately to date no experimental approach is available that mimics the natural conditions in which *D. seriata *is developing its pathogenicity. In this way, this manuscript contributes to the knowledge of biological basic aspects of this important phytopathogenic fungus, and, therefore, means a first step in the development of the molecular biology of this fungal strain. In fact, the gene encoding mithocondrial peroxyrredoxin-1 was cloned, sequenced and characterized (data not shown) based on the amino acid sequence of several internal peptides identified by tandem mass spectrometry (MS/MS).

## Results and discussion

### Analysis of fungal growth in the experimental conditions tested

**Figure 1:**
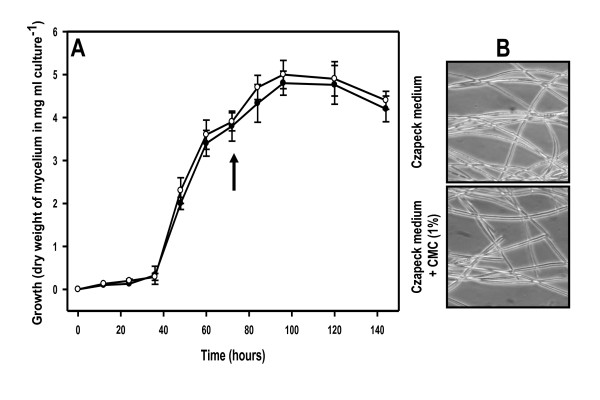
**(A).- Growth curve of *D. seriata *liquid cultures performed in Czapeck liquid medium (●) and the same medium supplemented with CMC (1%) (○)**. The sample collection points (72 h for every culture) are indicated by an arrow. (B).- Microscopic observation (400 ×) of 72 h old mycelia samples (corresponding to the sample collection points) showing that the mycelium exhibited a normal structure and integrity (no cellular lysis symptoms were observed at this time).

Fig. 1A shows the growth curves of *D. seriata *in liquid cultures developed in Czapeck medium and the same medium supplemented with CMC (1%). They were obtained from experiments carried out in the same experimental conditions to perform the proteome analysis. As it can be seen in Fig. 1, both curves are very similar, which obviously indicated that CMC did not have any significant effect over the growth rates. These data clearly indicated that most CMC could not be assimilated by *D. seriata*, as occurs in many others fungi. As it can be deduced from the graphic, the fungal growth in both experimental conditions increased rapidly beyond 36 h, reaching their maximum around 96 h. Samples for proteome analysis were taken at 72 h of growth corresponding approximately to the middle of the exponential phase of growth. The microscopic observation of mycelia samples collected at that time showed (Fig. 1B) a normal mycelium structure with no symptoms of cellular lysis.

### Visualization and analysis of the secreted proteins

**Figure 2:**
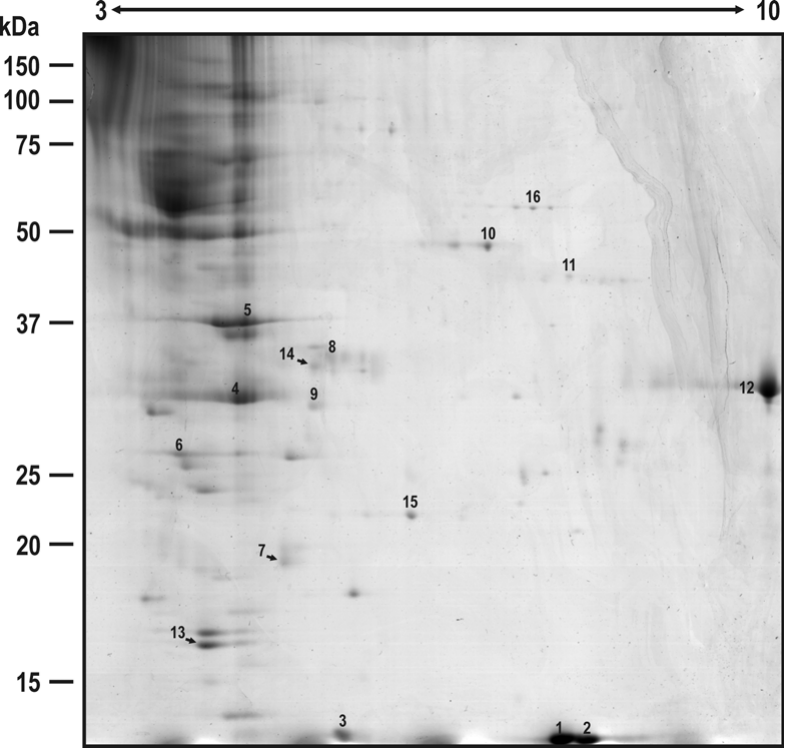
**2-DE of CBB-stained 12.5% SDS-PAGE of the secreted proteins of *D. seriata***.

The secreted proteins from *D. seriata *analyzed by 2-DE are showed in Fig. 2. All the secreted proteins that were subsequently identified by PMF and MS/MS analyses are numbered and listed in Table 1. Seventy-five extracellular proteins were reproducibly visualized in three independent experiments and sixteen could be identified by MS/MS. This low proportion of identified secreted proteins against visualized secreted proteins is not uncommon when the proteome of fungal species is analyzed, specially as in the case of *D. seriata *the genome remains unsequenced, which obviously makes difficult the identification of many proteins. In fact, when the extracellular proteomes of other fungal strains such as *Aspergillus flavus *[21], *Trichoderma reesei *[22], *Trichoderma harzianum *[23], or the phytopathogen *Sclerotinia sclerotiorum *[24] have been analyzed, a low ratio of identified proteins was also obtained. In the case of *D. seriata*, this fact could reflect that many of the non-identified secreted proteins are specific to this fungal species, or perhaps specific to endophytic fungi. The lack of proteomic analyses and of a significant number of characterized genes in endophytic fungi undoubtedly represents a limiting factor in the identification of proteins in these phytopathogenic fungi. In fact, up to our knowledge, no data regarding extense proteome and/or secretome analyses of other endophytic fungi are available (with the exception of a proteome analysis of *Neotyphodium lolii*, an endophytic fungus from the perennial ryegrass *Lolium perenne*) [25] and, in particular, there are no available data of other endophytic ascomycetes taxonomically close to Botryosphaeriales.

**Table 1:** Summary of the proteins secreted by *D. seriata*.

Spot	Acc. number	**Protein identity**^***a***^	**PM/Seq cov./score**^***b***^	Best peptides (PRO-BLAST)	Identified by	Experimental MW/p*I*	Theoretical MW/p*I*	Organism	Signal P
S1	gi|256724272	Hypothetical protein	2/20%/83		MASCOT	13.2/7.6	13.5/9.6	*Nectria haematococca*	NN^*c*^
S2	gi|255935271	Pc13g02210	2/14%/79	KIIYPAYTDK ARIKGDVVKPDKSYAPKAIP	PRO-BLAST	13.2/7.8	14.5/3.7	*Penicillium chrysogenum*	0.999
S3	gi|256724272	Hypothetical protein	2/20%/82		MASCOT	13.4/5.6	13.5/9.6	*Nectria haematococca*	NN
S4	gi|27924024	glucosidase	4/11%/97	GFNIGATNADGSC*K^*d*^ EGGHVSISAVSRSEDLYRGDSDASK	PRO-BLAST	30.2/4.7	33.4/9.3	*Fusarium sporotrichioides*	0.999
S5	gi|256732553	Predicted protein *(necrosis and ethylene inducing peptide 2 precursor)*	2/7%/97	GELHNDAFALFYAWYFAPG AVLAHDAVVGFAETVPFVGVWHAHK	PRO-BLAST	35.7/4.7	24.69/7.75	*Nectria haematococca*	0.999
S6	gi|239931549	Secreted protein	2/9%/105	SGASSWTAAR SSSFPHWLTLSGSC*NTR	PRO-BLAST	26.3/4.2	23.03/8.35	*Streptomyces ghanaensis*	0.989
S7	gi|189192036	Hypothetical protein	3/18%/94		MASCOT	19.4/5.1	22.5/7.6	*Pyrenophora tritici-repentis*	0.999
S8	gi|256726602	Hypothetical protein *(necrosis-and ethylene-inducing protein 1)*	3/8%/77	FGLFYAWYFPK ALSPHDSLTKDSYVR	PRO-BLAST	33.8/5.5	26.99/9.2	*Nectria haematococca*	1.000
S9	gi|156061869	Hypothetical protein *(necrosis-and ethylene-inducing protein 1)*	6/21%/77		MASCOT	26.0/5.2	26.9/6.6	*Sclerotinia sclerotiorum*	0.994
S10	gi|189196154	NADP-specific glutamate DH	9/31%/302		MASCOT	48.0/6.9	49.1/5.9	*Pyrenophora tritici-repentis*	No
S11	gi|171677093	Unnamed protein product *(aspartate aminotransferase)*	5/19%/80		MASCOT	43.4/7.6	43.0/8.3	*Podospora anserina*	No
S12	gi|237868680	Peptidase 1	4/8%/109	KTSGNFIASVASMSLGFESR KC*FPTEEDRRSGVDVYVVDTGLYTAHSEF	PRO-BLAST	30.9/9.4	38.7/7.9	*Pleurotus ostreatus*	1.000
S13	gi|255946596	Pc22g00190 *(cell wall protein PhiA)*	2/9%/81	KGIGYTTGAEPTPK ASTPKKLYVDR	PRO-BLAST	16.2/4.4	18.9/5.6	*Penicillium chrysogenum*	0.996
S14	gi|182412796	Hypothetical protein	2/2%/81	FGHWSPNASLATAAK YGPLSFVKGK	PRO-BLAST	33.1/5.4	74.5/6.8	*Opitutus terrae*	0.990
S15	gi|261355012	Superoxide dismutase	5/18%/111	GHGLYHDHSLFWENLAC*NLWPK SYIPPLPYAYDAIEPAISR	PRO-BLAST	22.0/6.3	26.8/10.3	*Verticillium albo-atrum*	No
S16	gi|189196326	Glutathione reductase	2/4%/90	SFDPIIKDTITKEYER VAPHYLVLNGSGGLASGR	PRO-BLAST	58.1/7.3	55.8/6.1	*Pyrenophora tritici-repentis*	No

^*a *^The assignment of the biological or enzymatic function to the hypothetical proteins (based on BlastP analyses) is shown between parentheses and in italic characters.

^*b *^Number of peptides matched/Sequence coverage (%)/Score.

^*c *^NN: Non-classically secreted proteins.

^*d *^C*: Cys as S carbamidomethyl derivative

A clear biological or enzymatic function could be solely attributed to ten out of the sixteen secreted proteins annotated (spots S4, S5, S8, S9, S10, S11, S12, S13, S15, and S16), whereas the rest (spots S1-S3, S6, S7, and S14) were annotated as hypothetical, putative uncharacterized protein or secreted proteins. In order to achieve a better identification of these proteins, their biological or enzymatic functions were determined. For this, the amino acid sequence of the hypothetical protein was used to perform a BlastP search against the non-redundant protein sequence database at NCBI (in these cases the putative biological functions are indicated between parentheses and in italics in Tables 1 and 2). In this way, spots S8 and S9 were determined to correspond to a necrosis and ethylene-inducing protein 1 (Nep1), whereas the spot 5 was determined to correspond to a necrosis and ethylene-inducing protein 2 precursor (Nep2). By the same approach, S11 was identified as an aspartate aminotransferase and S13 as a protein similar to the cell wall protein PhiA.

**Table 2:** List of cytoplamic proteins of *D. seriata*.

Spot	Acc. number	**Protein identity**^***a***^	**PM/Seq cov./score**^***b***^	Identified by	Total Ion score^*c*^	Experimental MW/p*I*	Theoretical MW/p*I*	Organism
1	gi|238498522	Phosphoglycerate kinase	12/30%/251	MASCOT		44.0/8.3	44.4/7.7	*Aspergillus flavus*
2	gi|67903236	Hypothetical protein *(peptidyl-prolyl cis-trans isomerase or cyclophilin 1)*	5/47%/219	MASCOT		18.2/8.5	17.8/8.9	*Aspergillus nidulans*
3	gi|169601412	Hypothetical protein *(enolase)*	11/30%/595	MASCOT		48.1/6.2	47.4/5.2	*Phaeosphaeria nodorum*
4	gi|198425409	Predicted Zn-finger U1-like	12/54%/88	MASCOT		12.3/4.9	22.6/9.5	*Ciona intestinalis*
5	gi|156053161	Hypothetical protein (*aldehyde reductase)*	2/5%/82	MASCOT	77	36.2/7.8	37.9/8.8	*Sclerotinia sclerotiorum*
6	gi|169625443	Hypothetical protein *(triosephosphate isomerase)*	7/23%/257	MASCOT		25.0/6.9	27.1/6.1	*Phaeosphaeria nodorum*
7	gi|46581334	Hypothetical protein	6/23%/112	MASCOT		30.2/7.4	16.8/6.3	*Desulfovibrio vulgaris*
8	gi|189210148	Signal transduction protein *(CipC-like antibiotic response protein)*	3/19%/117	MASCOT		13.1/4.8	14.9/4.9	*Pyrenophora tritici-repentis*
9	gi|115400267	Transaldolase	6/18%/165	MASCOT		35.2/5.2	35.2/6.2	*Aspergillus terreus*
10	gi|85107722	Hypothetical protein *(phosphoglycerate kinase)*	10/22%/217	MASCOT		45.2/7.9	45.3/6.2	*Neurospora crassa*
11	gi|120691	Glyceraldehyde-3-phosphate dehydrogenase	8/23%/334	MASCOT		38.3/8.2	36.6/6.7	*Cochliobolus lunatus*
12	gi|189204622	Fructose-bisphosphate aldolase	4/9%/156	MASCOT	140	38.4/6.7	40.2/5.5	*Pyrenophora tritici-repentis*
13	gi|189197705	Glycolipid transfer protein	5/25%/98	MASCOT		22.6/6.9	24.0/5.8	*Pyrenophora tritici-repentis*
14	gi|126139808	Hypothetical protein *(thiamine biosynthesis enzyme)*	5/20%/124	MASCOT		39.1/6.5	38.1/5.4	*Pichia stipitis*
15	gi|212542889	Glycerol dehydrogenase Gcy1 putative	4/17%/306	MASCOT		33.6/8.4	33.7/6.0	*Aspergillus fumigatus*
16	gi|67903236	Hypothetical protein *(peptidyl-prolyl cis-trans isomerase or cyclophilin 1)*	6/50%/214	MASCOT		18.5/7.9	17.8/8.9	*Aspergillus nidulans*
17	gi|70985178	Thiamine biosynthesis protein	8/25%/325	MASCOT		37.2/6.0	38.6/6.0	*Aspergillus fumigatus*
18	gi|255943905	Malate dehydrogenase	9/26%/471	MASCOT		34.6/7.9	35.8/8.4	*Penicillium chrysogenum*
19	gi|254583736	Hypothetical protein *(fumarate reductase)*	5/9%/161	MASCOT	144	56.9/6.7	54.0/8.6	*Zygosaccharomyces rouxii*
20	gi|154285406	Nucleoside-diphosphate kinase	6/38%/234	MASCOT		13.3/9.4	16.9/7.8	*Ajellomyces capsulatus*
21	gi|164429080	Adenosine kinase	5/12%/252	MASCOT	236	38.1/5.3	49.4/6.0	*Neurospora crassa*
22	gi|218722748	Conserved hypothetical protein	4/19%/157	MASCOT		51.4/4.9	42.8/5.8	*Talaromyces stipitatus*
23	gi|189196154	NADP-specific glutamate dehydrogenase	10/22%/504	MASCOT		46.4/7.5	49.1/5.9	*Pyrenophora tritici-repentis*
24	gi|58265982	Eukaryotic translation initiation factor 5A-1	5/30%/391	MASCOT		20.0/4.9	17.9/5.3	*Cryptococcus neoformans*
25	gi|145613040	Conserved hypothetical protein *(mitochondrial peroxiredoxin PRX1)*	4/21%/306	MASCOT		25.4/6.6	25.2/5.7	*Magnaporthe grisea*
26	gi|225877962	Putative saccharopine dehydrogenase	4/11%/146	MASCOT	131	45.2/5.6	43.5/5.2	*Gibberella fujikuroi*
27	gi|194669270	Endonuclease reverse transcriptase	23/24%/88	MASCOT		37.8/6.8	150.3/9.7	*Bos taurus*
28	gi|169625443	Hypothetical protein *(triosephosphate isomerase)*	5/19%/178	MASCOT		25.4/6.4	27.1/6.1	*Phaeosphaeria nodorum*
29	gi|255711238	KLTH0B02596p	9/70%/73	MASCOT		39.5/6.0	16.3/5.4	*Lachancea thermotolerans*
30	gi|154318339	Hypothetical protein *(Elongation factor 1β)*	2/20%/87	PRO-BLAST^*d*^		35.2/4.1	17.7/4.7	*Botryotinia fuckeliana*
31	gi|67903236	Hypothetical protein *(peptidyl-prolyl cis-trans isomerase or cyclophilin 1)*	5/35%/168	MASCOT		19.0/8.0	17.8/8.9	*Aspergillus nidulans*
32	gi|189196154	NADP-specific glutamate dehydrogenase	11/29%/541	MASCOT		46.4/7.2	49.1/5.9	*Pyrenophora tritici-repentis*
33	gi|145613040	Conserved hypothetical protein *(mitochondrial peroxiredoxin PRX1)*	4/21%/305	MASCOT		26.0/6.5	25.2/5.7	*Magnaporthe grisea*
34	gi|156057169	ATP sulfurylase	11/22%/98	MASCOT		74.6/4.4	64.2/6.6	*Sclerotinia sclerotiorum*
35	gi|169596136	Hypothetical protein *(piruvate kinase)*	12/28%/271	MASCOT		72.8/6.8	58.4/5.7	*Phaeosphaeria nodorum*
36	gi|154315126	S-Adenosylmethionine synthetase	5/18%/136	MASCOT		43.9/5.8	58.4/5.7	*Botryotinia fuckeliana*
37	gi|148361511	Vacuolar serine protease	7/20/115	MASCOT		15.7/6.1	41.0/5.8	*Cladosporium cladosporioides*
38	gi|85094513	Hypothetical protein *(phosphoglyceromutase)*	9/17/222	MASCOT		64.5/6.1	57.0/5.4	*Neurospora crassa*
39	gi|145232889	Hypothetical protein *(pyruvate decarboxylase)*	6/18/123	MASCOT		75.1/5.3	63.0/6.3	*Aspergillus niger*
40	gi|116196014	Hypothetical protein *(Heat shock 70 kDa protein, mitochondrial)*	12/20/369	MASCOT		84.8/5.4	68.2/5.6	*Chaetomium globosum*
41	gi|258575103	3'(2'),5'-Bisphosphate nucleotidase	10/25/335	MASCOT		41.5/5.6	38.1/5.0	*Uncinocarpus reesii*
42	gi|258577135	Rab GPD dissociation inhibitor alpha	9/25/396	MASCOT		61.8/5.5	52.0/5.7	*Uncinocarpus reesii*
43	gi|255936655	Pc13g09300 *(pyruvate decarboxylase)*	7/19/107	MASCOT		75.4/5.4	63.3/5.7	*Penicillium chrysogenum*
44	gi|114321348	Peptidoglycan synthetase FtsI	11/17/93	MASCOT		30.6/6.9	62.7/8.0	*Alkalilimnicola ehrlichii*
45	gi|39945784	Hypothetical protein (*UDP-glucose 4-epimerase Gal10*)	5/15/344	MASCOT	323	39.8/6.6	40.9/5.9	*Magnaporthe grisea*
46	gi|6647556	3-Isopropylmalate dehydrogenase A	5/16/198	MASCOT		41.4/5.8	38.8/5.7	*Aspergillus niger*
47	gi|74627960	Mannitol-1-phosphate 5-dehydrogenase	7/17/191	MASCOT		45.7/5.7	43.7/6.0	*Alternaria alternata*
48	gi|255942351	Hypothetical protein *(UDP-galactoyranose mutase)*	9/21/250	MASCOT		60.1/7.0	57.2/5.6	*Penicillium chrysogenum*
49	gi|145232889	Hypothetical protein *(pyruvate decarboxylase)*	6/18/118	MASCOT		74.0/5.9	63.0/6.3	*Aspergillus niger*
50	gi|576627	Alcohol dehydrogenase	4/16/240	MASCOT		36.0/8.2	37.6/6.4	*Candida albicans*
51	gi|255933774	Pc12g14620 (*flavohemoprotein*)	04/08/1972	PRO-BLAST^*e*^		42.5/7.5	46.0/6.0	*Penicillium chrysogenum*
52	gi|261359248	Aldose reductase	02/05/1991	PRO-BLAST^*f*^		37.0/7.5	33.6/5.4	*Verticillium albo-atrum*
53	gi|242812056	Alcohol dehydrogenase	04/06/1999	PRO-BLAST^*g*^		36.1/8.1	38.9/6.0	*Talaromyces stipitatus*

^*a *^The assignment of the biological or enzymatic function to the hypothetical proteins (based on BlastP analyses) is shown between parentheses and in italic characters.

^*b *^Number of peptides matched/Sequence coverage (%)/Score

^*c *^Total and best ion scores are shown for proteins showing a sequence coverage value less than 15%

^*d *^Two best peptides (PRO-BLAST): SYLVGYASPKADV/APDAAKFPHAAR

^*e *^Two best peptides (PRO-BLAST): RDPIIAEHGNTITTR/SMAPHINHIAATRNPVR

^*f *^Two best peptides (PRO-BLAST): DIGTDYVDLYLIHDAKKTGGR/ALGLSNFSR

^*g *^Two best peptides (PRO-BLAST): EFPANEDHIAFAAR/KIVHPPKVR

Twelve of the proteins visualized in the secretome were predicted to contain a Signal P motif (proteins S2, S4-S9, S12-S14) or to contain a non-classical protein secretion motif (proteins S1 and S3) indicating their extracellular location. Four intracellular proteins (lacking of Signal P motif) were present in the secretome of *D. seriata*: NADP-specific glutamate dehydrogenase (spot S10), aspartate aminotransferase (spot S11), superoxide dismutase (spot S15) and glutathione reductase (spot S16). Their presence might indicate that some cell lysis may have occurred during fungal growth (although the microscopy observation of mycelia at 72 h of growth did not let detect any sign of cellular lysis in the liquid cultures. See Fig. 1B), and/or preparation of the secretion protein samples, resulting in the release of some cytoplasmic proteins, even though care was taken to minimize this possibility. This problem has also been encountered by other authors analyzing the secretome of phytopathogenic microorganisms, concluding that the release of cytoplasmic proteins is likely an unavoidable consequence of sample preparation [24,26]. Alternatively, these intracellular proteins could be secretion-signal-less proteins that in some conditions are located at the cell surface, or detected in culture supernatants, like it has been reported by other fungi. These secretion-signal-less proteins are secreted by some unknown mechanism. Many of these proteins are glycolytic enzymes, chaperones, translation factors and others suggesting that they could be 'moonlighting' (multifunctional) proteins. In fact, a glutathione reductase (spot S16) has been identified as a secretion-signal-less protein in *S. cerevisiae *[27]. Also, Murad and colleagues have described a extracellular aminotransferase secreted by the entomopathogenic fungi *Metarhizium anisopliae *[28].

As it might be expected, some of the secreted proteins identified are proteins or enzymes that could be involved in pathogenesis. Thus, an enzyme like glucosidase (spot S4), responsible for the degradation of the polysaccharydes found in the plant cell wall, would allow the invading phytopathogenic fungus to penetrate more easily the plant cells. Other proteins involved in peptide and/or protein degradation such as peptidase 1 (spot S12) could be responsible for the degradation of cell wall proteins and the inactivation or inhibition of some plant defense response proteins, whose expression may be induced by pathogen challenge. This possibility has been suggested for some secreted proteases from the phytopathogenic ascomycete *Sclerotinia sclerotiorum *[24]. Spots S5, S8 and S9 were identified as necrosis and ethylene-inducing proteins (Nep1 and Nep2). Nep proteins represent a new class of necrotic elicitors. Indeed, the extracellular Nep1 protein, initially identified from the culture filtrate of *Fusarium oxysporum*, has the ability to induce different kind of injuries in susceptible plants, such as necrosis in cacao or cell death and necrotic spots in *Arabidopsis thaliana *[29,30]. Plant responses to Nep proteins include induction of pathogen related (PR) genes, accumulation of ROS and ethylene, changes in K^+ ^and H^+ ^channel fluxes, callose apposition, altered cell respiration, induction of the hypersensitive response and localized cell death [29]. Finally, spot S13 could correspond to a cell wall protein PhiA. In *Aspergillus nidulans*, PhiA protein is highly up-regulated by exposition to several antifungal compounds [31] and it is involved in phialide and conidium normal development [32]. These data suggest that this protein could play a significant role as a defense mechanism against some antifungal responses, as when the plant produces toxic metabolites and, at the same time, the synthesis of PhiA initiates to ensure fungi survival by producing conidia [32].

Although we also made several attempts in order to characterize the secretome of *D. seriata *when growing in the presence of CMC the gels obtained were always of very poor quality due to irresoluble technical problems. The capability of *D. seriata *to metabolize CMC is limited and therefore high amounts of CMC remained in the supernatant cultures that interfered with the protein precipitation protocols. We tried to remove CMC from supernatants by several methods, including extensive dialysis and precipitation of CMC by using ammonium sulfate (80%), or PEG-CaCl_2_, but in all cases no satisfactory results were obtained.

### Visualization and analysis of cytoplasmic proteins

**Figure 3:**
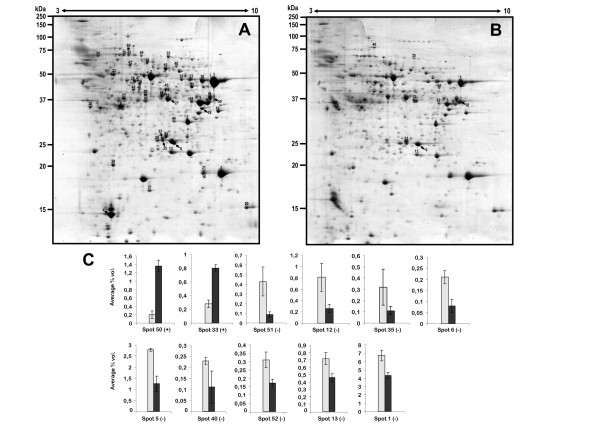
**2-DE CBB-stained 12.5% SDS-PAGE gels of *D. seriata *cytoplasmic proteins corresponding to a liquid culture carried out in normal conditions (A), and to a liquid cultured developed in the presence of CMC**. (C).- Density plots graphically representing the (%) volumen average corresponding to the proteins up-regulated (+) and down-regulated (-) are shown. (%) Vol. corresponds to the volume of each spot divided by the total volume of all the spots in the gel. Differentially expressed proteins were considered when the ratio of the (%) Vol. average (3 gels) between conditions was higher than 1.5 and the *p*-value deduced from the *t*-test was <0.05. The density plots corresponding to each category (up- and down-regulated proteins) are ordered from higher to lower ratios (as showed in Table 3).

The proteins extracted from the whole mycelia of *D. seriata *and separated by 2-DE are showed in Fig. 3. Of the 550 mycelial proteins reproducibly visualized in the 2-DE gels from three independent protein extractions, 53 proteins were identified by PMF and MS/MS as shown in Table 2. However, 25 out of these 53 proteins were annotated in databases as hypothetical or putative uncharacterized proteins. As indicated above for the secreted proteins, a BlastP search against the non-redundant protein sequence database (NCBI) was performed by using their amino acid sequences (their putative biological functions are indicated between parentheses and in italics in Table 2) in order to achieve a better identification of the biological or enzymatic functions of these hypothetical proteins. This strategy let us assign a clear biological or enzymatic function to 22 out of the 25 hypothetical proteins.

The 53 annotated proteins represented 44.7% of the total spot volume (%Vol), whereas it is remarkable that the first 12 spots identified (numbered 1 to 12 in Table 2) corresponded to 29.75% of the total spot volume. These values obviously indicated that these proteins are highly abundant in the cytoplasm of *D. seriata *and probably suggest a relevant cellular function. In fact, 6 out of the 12 more abundant proteins were glycolytic enzymes like phosphoglycerate kinase (spots 1 and 10), triose phosphate isomerase (spot 6), enolase or phosphopyruvate dehydratase (spot 3), glyceraldehyde 3-phosphate dehydrogenase or GAPDH (spot 11) and fructose bisphosphate aldolase (spot 12). This can be explained biologically if we take into account that the glycolytic pathway plays a central role in the cellular energetic metabolism, and obviously glycolytic enzymes are found among the most expressed proteins in many different organisms.

As *D. seriata *is a phytopathogenic fungus, the annotation of proteins of importance in the pathogenesis process is of special interest. In this way, the case of GAPDH is significant since it is a very complex enzyme which possesses, besides its role in the glycolytic cycle, up to other fifteen different functions in mammalian cells and microbial pathogens [33], including a role as virulence factor for a vast variety of microbial pathogens [34,35]. Curiously, the second most abundant cytoplasmic protein (spot 2; 4.33%Vol) was identified as a peptidyl prolyl cis-trans isomerase (also spots 16 and 31) or cyclophilin. Cyclophilins have peptidyl prolyl cis-trans isomerase activity that performs protein folding by catalyzing the isomerization of peptide bonds preceding proline residues [36]. More interestingly, cyclophilins have been reported as an important virulence factor in the plant infection of rice by the ascomycete fungus *Magnaporthe grisea *[37], in the yeast *Cryptococcus neoformans *[38] and in the later infection stages or plant colonization by *Botrytis cinerea *[39].

Another abundant protein (spot 8) was identified as a signal transduction protein. A BlastP search let us confirm that this protein showed high amino acid identity (58%) to a CipC-like antibiotic response protein from *Aspergillus fumigatus *(gi|70997948). CipC-like antibiotic response protein (or concanamycin-induced protein) has been identified as an up-regulated protein in several fungal strains exposed to antibiotics, e.g. *A. nidulans*. In addition, in *S. sclerotiorum*, seven ESTs corresponding to CipC were identified during interactions with *Brassica napus *and classified as pathogenesis-associated genes [40]. In the case of the phytopathogenic soilborne fungus *Verticillium dahlia*, CipC protein has been suggested to help to its survival in soil by inactivating antibiotics produced by other soil microorganisms [41]. Spot 18 was identified as malate dehydrogenase (MDH). This enzyme catalyzes the reversible conversion of oxalacetate and malate. Oxalacetate is an oxalic acid precursor which has been described as a pathogenicity factor in *B. cinerea *[42], since the secretion of this acid is supposed to create an acidic environment that makes easier the pathogenic activity of the fungus. A role of MDH in pathogenesis has also been suggested for *B. cinerea *since this enzyme is produced exclusively or at higher levels in a more virulent *B. cinerea *2100 strain [43]. Apart from the above-mentioned proteins, we could also identify (spot 40) a putative mitochondrial heat shock 70 kDa protein (HSP70) which is a molecular chaperone essential for tolerance to protein denaturing stresses as well as for protein biogenesis. HSP70 seems to be involved in fungal pathogenesis since it has been reported to facilitate the infection of coffee leaves by the fungus *Colletotrichum gloeosporioides *[44].

Some of the in-gel visualized proteins that we tried to identify could not be annotated, probably due to the fact that homologous proteins are not present in databases, which obviously could indicate that most of them might be novel or uncharacterized. In fact, proteomic analysis of many fungal species, particularly filamentous fungi, is difficult due to the lack of publicly available genome sequence data and the problems associated with cross-species comparisons [15].

### Functional classification of cytoplasmic proteins

**Figure 4:**
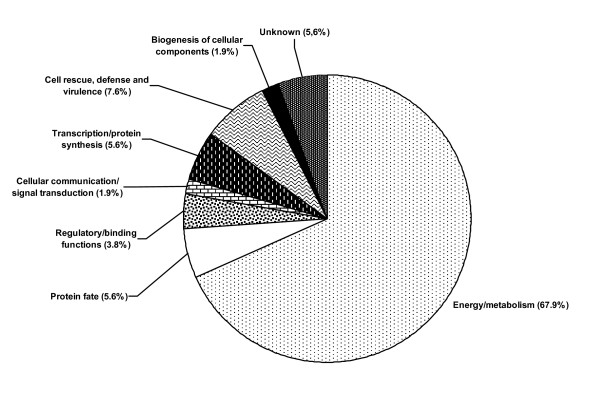
**Functional classification of identified cytoplasmic proteins of *D. seriata *according to the MIPS Functional Catalogue (FunCat description)**. The listed percentage corresponds solely to the proportion of the identified proteins and does not take into account the proteins that were not annotated.

Fig. 4 shows a graphical representation of the distribution of the identified cytoplasmic proteins based on a functional classification carried out according to the MIPS Functional Catalogue (FunCat description). Most of the identified proteins (67.9%) were metabolic enzymes or proteins involved in energy production. The proteins involved in energy production could be categorized into two groups. First, those involved in the glycolytic pathway like phosphoglycerate kinase (spots 1 and 10), enolase (spot 3), triosephosphate isomerase (spots 6 and 28), glyceraldehyde-3-phosphate dehydrogenase (spot 11), aldolase (spot 12), phosphoglyceromutase (spot 38) and pyruvate kinase (spot 35). Second, those that are components of the tricarboxylic acid pathway and mithocondrial proteins: pyruvate decarboxylase (spots 39, 43, 49) and malate dehydrogenase (spot 18). Several of the proteins identified are involved in carbohydrate metabolism such as UDP-glucose 4-epimerase (spot 45), mannitol-1-phophate-5-dehydrogenase (spot 47), UDP-galactopyranoside mutase (spot 48), alcohol dehydrogenase (spots 50 and 53) and aldose reductase (spot 52); whereas others such as NADP-specific glutamate dehydrogenase (spots 23 and 32), saccharopine dehydrogenase (spot 26) and S-adenosylmethionine synthetase (spot 36) are engaged in amino acid metabolism. Besides, several proteins involved in nucleic acid metabolism were annotated such as a nucleoside-diphosphate kinase (spot 20), adenosine kinase (spot 21), biphosphate nucleotidase (spot 41) and two proteins related to thiamine biosynthesis (spots 14 and 17).

There were also proteins (7.6%) implicated in cell rescue, defense and virulence like mitochondrial peroxiredoxin 1 (spots 25 and 33), a heat shock 70 kDa protein (spot 40) and a flavohemoprotein (spot 51).

The remaining functional categories included a lower number of proteins. Thus 5.6% were classified as unknown proteins. The same percentage (5.6%) was estimated to be proteins involved in protein fate, like peptidyl-prolyl cis-trans isomerase (spots 2, 16, and 31). Proteins with regulatory and binding functions, like a predicted Zn-finger U1-like protein (spot 4) and a Rab GDP dissociation inhibitor alpha (spot 42) represented 3.8%; whereas proteins with a role in the transcription/translation processes, like an eukaryotic translation initiation factor 5A-1 (spot 24), an endonuclease reverse transcriptase (spot 27) and an elongation factor 1β (spot 30) represented 5.6% of the characterized proteins. As for spot 44 (biogenesis of cellular components, 1.9%), it was identified as a putative peptidoglycan synthetase FtsI, which obviously does not make any sense in a fungal strain, indicating that the primary role of this protein is still to be determined. Finally, solely one protein (spot 8; 1.9%) was classified into the cellular communication/signal transduction category.

### Differentially expressed cytoplasmic proteins in the presence of CMC

Since cellulose is one of the major components of the plant cell wall, those proteins up-regulated when a phytopathogenic fungal strain grows in its presence are supposed to have a significant role in the pathogenicity process. This approach has been recently postulated as useful for the identification of putative pathogenicity factors in *Botrytis cinerea *[19,20]. As reported above, the analysis of the corresponding sets of 2-DE gels revealed an average of 550 protein spots in the proteome of *D. seriata*. Three proteins were overexpressed when the fungus grew in the presence of CMC [CMC is a cellulose derivative with carboxymethyl groups bound to the hydroxyl groups of the glucose unit. There can be up to three substituents on each glucose unit, thus the maximal degree of substitution is 3]. Two of them were identified as mitochondrial peroxiredoxin-1 and alcohol dehydrogenase (Table 3), suggesting a putative role in the pathogenicity process. The third protein could not be identified despite the good quality of the PMF spectra, probably due to the absence of similar proteins in protein databases.

**Table 3:** Identified proteins of *D. seriata *up-regulated (+) and down-regulated (-) in the presence of CMC^a^

Spot no.	Expression level	Ratio	p-value	**Protein identity**^***b***^
50	+	6.72	0.0001	Alcohol dehydrogenase
33	+	2.82	0.0001	Conserved hypothetical protein (*Mitochondrial peroxiredoxin PRX1)*
51	-	5.00	0.0082	Pc12g14620 *(flavohemoprotein)*
12	-	3.16	0.0102	Fructose-bisphosphate aldolase
35	-	2.96	0.0444	Hypothetical protein *(piruvate kinase)*
6	-	2.77	0.0025	Hypothetical protein *(triosephosphate isomerase)*
5	-	2.24	0.0008	Hypothetical protein *(aldo/keto reductase)*
40	-	2.07	0.0254	Hypothetical protein *(heat shock 70 kDa protein, mitochondrial)*
52	-	1.87	0.0045	Aldose reductase
13	-	1.56	0.0053	Glycolipid transfer protein
1	-	1.54	0.0022	Phosphoglycerate kinase

^*a *^Differentially expressed proteins were considered when the ratio of the %Vol average (3 gels) between strains was higher than 1.5 and the p-value deduced from the t-test was <0.05.

^*b *^The assignment of the biological or enzymatic function to the hypothetical proteins (based on BlastP analyses) is shown between parentheses and in italic characters.

Peroxiredoxins (PRXs) are an ubiquitous family of thiol-specific antioxidant proteins that help to control intracellular peroxide levels. They also control cytokine-induced peroxide levels which mediate signal transduction in mammalian cells. Their broad distribution and high levels of expression suggest that they are both an ancient and important enzyme family [45]. On the other hand, as a ROS, hydrogen peroxide seems to play a significant role in plants under severe environmental conditions, including biotic stresses produced by phytopathogen infection. In fact, hydrogen peroxide participates in many resistance mechanisms, including reinforcement of the plant cell wall, phytoalexin production, and enhancement of resistance to various stresses [46]. Taking together all these evidences, we might speculate that an increase in the H_2_O_2 _levels in grapevine, as a result of fungal infection, could be counteracted by an increase in the peroxiredoxin levels in the fungal cells. In fact, peroxiredoxins have been proved to be important not only for resistance to H_2_O_2 _stress but also for virulence in the dimorphic fungus *Cryptococcus neoformans*, since they contribute to virulence in a mouse model [47]. Also it has been reported that peroxiredoxin Tsa1 is highly induced during 37°C hyphal growth in *Candida albicans *(the pathogenic form of this dimorphic yeast), and that the thioredoxin system genes are induced after peroxide exposure [48]. All these data support that an increase in the levels of peroxiredoxins in fungal strains could significantly contribute to the development of the pathological response in phytopathogenic fungi.

Alcohol dehydrogenases (ADH) are complex enzymes. Up to 7 different ADHs (*ADH1 *to *ADH7*) have been identified in *Sacharomyces cerevisiae *[49]. Although the primary role of ADH (*ADH1*) is to catalyze the production of ethanol from acetaldehyde in cells grown anaerobically or in the presence of a glucose excess, they can also achieve other cellular functions such as the catalyzation of the opposite reaction (*ADH2 *and *ADH5 *can convert ethanol accumulated during aerobic growth to acetaldehyde) or the participation in aldehyde reduction in oxidative conditions (*ADH6*), allowing the use of 2,3-butanediol as carbon and energy source [50]. The putative meaning of increased levels of ADH observed in the presence of CMC (and its putative role in virulence) seems more difficult to decipher. In fact, in the pathogen *C. albicans*, it has been reported that Adh1p restricts its ability to form biofilms on catheter surfaces through an ethanol-based mechanism and that the disruption of the *ADH1 *gene does not affect its ability to form the pathogenic hyphal form of this fungus [51]. We might speculate that the higher levels of ADH detected in the presence of CMC could be related to a tendency of the fungus growth to perform an anaerobic metabolism under this condition. Perhaps, this could reflect some difficulty to access oxygen when *D. seriata *grows inside the trunk of grapevines (in the vascular system), where availability of oxygen may be reduced. In these conditions, a higher ADH activity could be beneficial in order to obtain energy. However, we must not forget that in the presence of CMC up to four glycolytic enzymes are down-regulated (Table 3): fructose-bisphosphate aldolase, triose phosphate isomerare, phosphoglycerate kinase and pyruvate kinase, which obviously implicated a slower rate of efficiency of the glycolytic pathway, and therefore lower intracellular pyruvate levels available for ethanol production. This fact could be compensated by the nitrate reduction (remember that nitrate is the sole nitrogen source in the Czapeck media used) to nitrite, and the subsequent reduction to ammonia, coupled with the catabolic oxidation of ethanol to acetate and substrate-level phosphorylation that supports growth under anaerobic conditions [52].

Out of the 30 proteins down-regulated in the presence of CMC, solely 9 could be identified (Table 3). Most of these proteins are glycolytic enzymes (spots 12, 6, 35 and 1) or proteins involved in carbohydrate metabolism (aldose reductase, spot 52), which is difficult to understand if we bear in mind that the fungal strain is growing in a culture media supplemented with CMC and containing sucrose. Regarding this point, we could speculate that the metabolism of CMC could have some negative/inhibitory effect on some key enzymes of the glycolytic pathway. It should be noted that CMC can be degraded at different rates depending on the effectiveness of the different types of cellulases produced by the fungal strain to attack CMC. Different sugars (glucose, cellobiose, cellotriose, cellotetraose and cellopentaose) can be generated as degradation products [53]. The identification of these CMC-degradation products suggests that the carboxy-methyl groups of CMC could be released as acetate, which might exert a repressive effect on the levels of different glycolytic enzymes, as it has been shown for the bacteria *Escherichia coli *[54]. In this way, the CMC metabolism could be the cause in the observed diminishing of the levels of several glycolytic enzymes. Alternatively, it could be also possible that the reduced amounts of some glycolytic enzymes could be due by an increased carbon flow through the pentose phosphate pathway.

Finally, we could emphasize that although, as indicated above, the growth of phytopathogenic fungi in the presence of CMC has been postulated as a promising tool for the identification of putative proteins important for pathogenicity, our results indicated that, at least in the case of *D. seriata*, no significant differences could be detected in the proteome of this fungus grown in a defined medium and in the same medium supplemented with CMC, since a limited number of proteins were up- or down-regulated. Unfortunately, Fernández-Acero and colleagues [20] did not compare the growth of *Botrytis cinerea *proteome under normal conditions and cellulose-degradation conditions, in order to establish differences in the proteomes.

## Conclusion

The main aim of this study was to perform a proteome analysis of the significant phytopathogenic and endophytic fungus *D. seriata *in order to understand better its lifecycle as well as its ability to infect grapevine plants producing trunk diseases. This is the first approach to study the proteome of this fungal strain which contributes to cause severe economic losses to wine industry all around the world. Notoriously, a high proportion of the identified proteins in the secretome could have a significant role in the pathogenicity process, including a glucosidase and a peptidase that could be involved in the degradation of the vegetable cell wall. More interestingly, three necrosis and induced proteins (Nep) were identified in the secretome of *D. seriata*. This is particularly intriguing since Nep proteins share a high degree of sequence similarity and they are able to induce an hypersensitive-like death response in a variety of plants, also inducing necrotic responses in plant tissues [55]. The putative role of *D. seriata *Nep proteins in the initiation or progression of the grapevine infection process needs to be further investigated in order to determine their significance for pathogenicity. Moreover, the detection of a putative secreted PhiA protein needs to be highlighted, since a high production of this protein has been suggested to ensure fungi survival by inducing a higher conidia production rate in response to the presence of toxic metabolites.

Regarding cytoplasmic proteins and in addition to the annotated proteins involved in basic cellular functions such as energy and metabolism, protein fate or transcription and protein synthesis, several proteins identified as virulence factor in other fungal strains were annotated. Among them, we can cite GAPDH, 3 peptidyl prolyl cis-trans isomerases (or cyclophilins), a CipC-like antibiotic response protein, malate dehydrogenase and a mitochondrial heat shock 70 kDa protein.

The differences observed in the proteome by growing the fungi in the presence of CMC were minor than expected, since solely three proteins were up-regulated and thirty down-regulated. These data could mean that this strategy might not be very effective to identify putative pathogenicity factors in phytopathogenic fungi. Finally, we would like to add that we hope that this study means a first step to advance in the necessary molecular biology studies of *D. seriata *that can contribute to deciphering the role of these putative pathogenicity factors in the development of grapevine decline diseases.

Finally, we hope that the proteomic data obtained will let us in a future develop further studies. In fact, and based on the peptide sequences obtained in this work, we have cloned and sequenced the *D. seriata Prx*1 gene (data not shown). This is the first gene of this important phytopathogen to be cloned, which supposes a significant contribution for the developing of the Molecular Biology in this microorganism. In fact, a transformation methodology for *D. seriata *is currently being developed to try to interrupt or attenuate the expression of *Prx*1 gene in order to check its putative role in the virulence process. The development of this methodology will let us in a future to check the involvement of other putative virulence proteins identified in this study in the pathogenicity process.

## Materials and methods

### Culture conditions

The fungal pathogen *Diplodia seriata *(isolated from an affected *Vitis vinifera *plant) was routinely maintained on potato dextrose agar (PDA) (Scharlau Chemie S.A., Barcelona, Spain) plates at 4°C. Liquid cultures were performed through the following protocol: a total of 12 Czapeck agar plates (sucrose, 3%; NaNO_3_, 0.4%; K_2_HPO_4_, 0.1%; MgSO_4_·7H_2_0, 0.1%; FeSO_4_·7H_2_0, 0.001%; and agar, 2%) were surface-covered with cellophane sheets. Plates were inoculated in the center with an agar plug (0.5 cm) removed from the outer edge of a 6-day-old fungal colony grown on Czapeck agar plates and incubated at 25°C for 5 days. The mycelia from two Czapeck plates were removed from the cellophane and disrupted with a beater. Next, mycelia were transferred to flasks containing 150 ml of Czapeck liquid medium (control conditions), or Czapeck liquid medium supplemented with 1% carboxymethylcellulose (CMC) (Sigma, St. Louis, MO, USA). The cultures were incubated in an orbital shaker at 120 rpm and 25°C for up to 7 days. The whole fungal proteome and secretome of *D. seriata *were prepared respectively from fungal mycelia and culture supernatants from 3-day-old liquid cultures. Three independent replicates were analyzed. Culture supernatants were collected by filtration and secreted proteins were immediately precipitated. Mycelia were harvested by filtration, washed with sterile water and stored at -20°C until use. Fungal growth was estimated as dry-weight of mycelia by filtering 5 ml of liquid cultures through a glass fiber filter (0.5 μm pore size). Mycelia were washed 3 times with deionized water and dried at 80°C for 48 h before weighing.

### Protein extraction

Cytoplasmic proteins were isolated by using a protocol based on that of Fernández-Acero and colleagues [56], which was modified as follows. Frozen mycelia were ground to a fine powder with a pre-cooled mortar and pestle using liquid nitrogen. Two grams of powder were stirred (4°C for 2 h) in 10 ml of 10 mM potassium-phosphate buffer (K_2_HPO_4_-KH_2_PO_4_) (pH 7.4) containing 0.07% (w/v) 1,4-DTT and supplemented with Complete™ protease inhibitor cocktail (Roche, Mannheim, Germany) (one tablet per 10 ml of buffer). The cellular extracts were clarified twice by centrifugation (5 min, 15 000 × *g*). Proteins were precipitated for at least 1 h at -20°C after the addition of 1 volume of 20% trichloroacetic acid (TCA) in acetone containing 0.14% (w/v) DTT. The protein pellet was washed twice with acetone followed by an additional washing step with 80% acetone. The proteins were finally resuspended in 500 μl of sample buffer: 8 M urea, 2% CHAPS, 0.5% IPG Buffer pH 3-10 NL (GE Healthcare, Uppsala, Sweden), 20 mM DTT, and 0.002% bromophenol blue. The insoluble fraction was discarded by centrifugation at 15 000 × *g *for 5 min. The supernatant was collected and protein concentration was determined using the Bio-Rad Protein Reagent (Bio-Rad, Hercules, CA, USA) and bovine serum albumin as standard.

To prepare the secreted proteins for the secretome analysis the proteins from culture supernatants (100 ml) were precipitated for at least 1 h at -20°C after the addition of 1 volume of 20% TCA in acetone containing 0.14% (w/v) DTT. The precipitated proteins were harvested by centrifugation at 15 000 × *g *for 20 min at 4°C. The protein pellet was washed twice with acetone followed by an extra washing step with 80% acetone, and then resuspended in 500 μl of sample buffer. The insoluble fraction was discarded by centrifugation (5 min, 15 000 × g). Protein concentration was determined as indicated above.

### 2-D gel electrophoresis

For the analysis of the cytoplasmic and extracellular proteomes 18-cm IPG strips [pH 3-10 NL (non-linear), GE Healthcare] were loaded with 350 μg of soluble proteins in the sample buffer (see above). Proteins were focused using an Ettan IPGphor II (GE Healthcare) at 20°C according to the following program: 1 h, 0 V; and 12 h, 30 V (rehydratation); 2 h, 60 V; 1 h, 500 V; 1 h, 1 000 V; 30 min gradient up to 8000 V; and finally 7 h at 8,000 V until 50 kVh. Focused IPG gels were equilibrated twice for 15 min in a buffer containing 50 mM Tris-HCl (pH 8.8), 6 M urea, 30% glycerol, 2% SDS, 0.002% bromophenol blue and 1% DTT. For the second equilibration step, DTT was replaced by 4.0% iodoacetamide. The second dimension was run on a 12.5% SDS-PAGE gel in an Ettan Dalt Six apparatus (GE Healthcare) for 45 min at 3 W per gel, and then for 3.5 h at 18 W per gel. Precision Plus Protein Standards (Bio-Rad) were used as protein markers. Gels were stained following the "Blue Silver" staining method [57] using 0.12% CBB G-250 (Sigma), 10% ammonium sulfate, 10% phosphoric acid and 20% methanol.

### Gel analysis

2-D images were captured by scanning stained gels using a ImageScanner II (GE Healthcare) previously calibrated with a greyscale marker (Kodak, Rochester, NY, USA), digitalized with Labscan 5.00 (v 1.0.8) software (GE Healthcare) and analyzed with the ImageMaster™ 2D Platinum v 5.0 software (GE Healthcare). Three gels of each strain obtained from three independent cultures (biological replicates) were analyzed to guarantee representative results. After automated spot detection, spots were checked manually to eliminate any possible artifacts, such as streaks or background noise. Biological replicates showed less than 10% and 5% of variability in the number of protein spots detected for intracellular and extracellular proteomes, respectively. The patterns of each sample were overlapped and matched, using landmark features, to detect potential differentially expressed proteins. The gel presenting the highest spot number was selected as Master gel and the coefficient of determination (r2) for each gel pair was calculated in order to ensure the analyses reliability (pair average r2: 0.82). Spot normalization, as an internal calibration to make data independent of experimental variations among gels, was made using relative volumes (%Vol) to quantify and compare the gel spots. %Vol corresponds to the volume of each spot divided by the total volume of all the spots in the gel. Differentially expressed proteins were considered when the ratio of the %Vol average (3 gels) between conditions was higher than 1.5 and the *p*-value deduced from the *t*-test was <0.05.

### Protein Identification by MALDI-TOF/TOF MS and MS/MS

The protein spots of interest were manually excised from CBB-stained gels by biopsy punches, placed in a microcentrifuge tube, and washed twice with ddH_2_O. The proteins were digested following the method of Havlis *et al. *[58]: samples were dehydrated with ACN (Carlo Erba, Barcelona, Spain) for 5 min, reduced with 10 mM DTT (GE Healthcare) in 25 mM ammonium bicarbonate (Fluka, Buchs, Switzerland) for 30 min at 56°C and subsequently alkylated with 55 mM iodoacetamide (MS grade; Sigma) in 25 mM ammonium bicarbonate for 15 min in the dark. Finally, samples were digested overnight at 37°C with 10.0 ng/μl of sequencing-grade modified porcine trypsin (Promega, Madison, WI, USA) in 25 mM ammonium bicarbonate (pH 8.5). After digestion, the supernatant was collected, speed-vacuum dried and resuspended in 4 μl of 50% ACN, 0.1% TFA in ddH_2_O. One μl was spotted on a MALDI target plate and air dried for 10 min at room temperature. Subsequently, 0.5 μl of matrix [3 mg/ml of α-cyano-4-hydroxy-trans-cinnamic acid (LaserBio Labs, Sophia-Antipolis, France)] diluted in 0.1% TFA-ACN/H_2_O (1:1, v/v) was added to the dried peptide digestion and air dried for another 5 min at room temperature. The samples were analyzed with a 4800 Proteomics Analyzer MALDI-TOF/TOF mass spectrometer (Applied Biosystems, Foster City, CA, USA). A 4700 proteomics analyzer calibration mixture (Cal Mix 5; Applied Biosystems) was used as external calibration. All MS spectra were internally calibrated using peptides from the trypsin digestion. The peptides observed (up to 65 peptides per spot) were collected and represented as a list of monoisotopic molecular weights with a S/N greater than 20 using the 4000 Series Explorer v3.5.3 software (Applied Biosystems). All known contaminant ions (such as trypsin- and keratin-derived peptides) were excluded from further consideration in protein database search and later MS/MS analysis. Hence, from each MS spectrum, the fifteen most intensive precursors with a S/N greater than 20 were selected for MS/MS analyses with CID (atmospheric gas was used) in 2-kV ion reflector mode and precursor mass windows of ±7 Da. The default calibration was optimized for the MS/MS spectra processing.

For protein identification, Mascot Generic Files combining MS and MS/MS spectra were automatically created and used to interrogate a non-redundant protein database using a local license of Mascot v 2.2 from Matrix Science through the Global Protein Server (GPS) v 3.6 (Applied Biosystems). The search parameters for the PMF and MS/MS spectra were set as follows: i) NCBInr (2009.11.03) sequence databases were used; ii) taxonomy: All entries (9993394 sequences), Fungi (644746 sequences); iii) fixed and variable modifications were considered (Cys as S carbamidomethyl derivative and Met as oxidized methionine); iv) one missed cleavage site was allowed; v) precursor tolerance was set at 100 ppm and MS/MS fragment tolerance at 0.3 Da; vi) peptide charge was 1+; vii) the algorithm was set to use trypsin as the enzyme. Protein candidates provided by this combined PMF and MS/MS search were considered as valid when the global Mascot score was greater than 83 (total database) or 71 (fungi database) with a significance level of p < 0.05. Further additional criteria were taken into account for confident identification. First, the protein match should have at least 15% sequence coverage. Second, only those proteins with a Mascot ions score (see Table 2: Total Ion Score) above 77 in the MS/MS analysis (with a significance level of p < 0.05) should be considered as valid. Due to the almost absolute absence of protein or DNA sequences deposited in databases for *D. seriata*, those non-confirmed proteins after Mascot database search were identified by *de novo *sequencing and BLAST similarity searching following the procedure described by Liska and Shevchenko [59]. All MS/MS spectra for a sample were sequenced *de novo *using ProBLAST software (Applied Biosystems). The best fragmented ions were manually selected and the top ten candidate peptide sequences for each MS/MS were combined into a single text-format search string by means of the DeNovo Explorer software (Version 3.6) included in GPS Explorer Software (Applied Biosystems). This search string was used for similarity searches using the BLASTP algorithm [version 1.4.0] against an in-house protein sequence database containing the NCBInr sequence database (date: 2010.01.28; 10385566 sequences; 3543119913 residues) under the following parameters: i) fixed modifications: Cys as S carbamidomethyl derivative; ii) mass tolerance: 0.3 Da iii) E-value threshold: 20; iv) scoring matrix: PAM30MS; v) algorithm enzyme: trypsin. Protein identification significance was judged using the BLASTP scoring algorithm. Only those proteins matched by a minimum of two peptide sequences with a score higher than 52 according to DeNovo Explorer software were included in the results list, providing more stringent criteria for the identification. Information about the peptides identified can be found in the additional file 1 (Cytoplasmic PRO-BLAST peptides), additional file 2 (Secretome PRO-BLAST peptides), and additional file 3 (MASCOT Search Results).

### Protein functional annotation

For proteins with no assigned function, homology searches were performed using the BlastP program against all non-redundant protein sequences deposited in the NCBI database http://blast.ncbi.nlm.nih.gov/Blast.cgi. Protein alignments were considered to be significant when their *e*-value was below 10^-30^. The secretion mechanism was predicted using SignalP (for classical secretion signal motifs) and SecretomeP (for non-classical signal motifs) software [60,61]. The identified proteins were distributed into categories according to their involvement in the biological process based on the MIPS Functional Catalogue (FunCat: http://mips.helmholtz-muenchen.de/proj/funcatDB/search_main_frame.html) [62].

## Competing interests

The authors declare that they have no competing interests.

## Authors' contributions

RC carried out the *D. seriata *cultures and protein isolation, interpreted data and performed the gene cloning. CB performed 2DGE and acquired of MS/MS data and *de novo *sequencing. RMM helped with the design of experiments, the construction of the genomic library and cloning of Prx1 gene. JJRC carried out the design of experiments and the coordination of the study, helped with the data analysis and drafted the manuscript. All authors read and approved the final manuscript.

## Supplementary Material

Additional file 1 **Cytoplasmic proteins identified by means of PRO-BLAST search**. Fragmentation spectra obtained from cytoplasmic proteins after *de novo *sequencing using ProBLAST software and properly identified by BLAST similarity searching are showed. Click here for file

Additional file 2 **Extracellular proteins identified by means of PRO-BLAST search**. Fragmentation spectra obtained from secreted proteins after *de novo *sequencing by means of ProBLAST software and suitably identified by BLAST similarity searching are showed. Click here for file

Additional file 3 **MASCOT Search Results**. Data (observed, expected and calculated mases, ppm and misscleavages) corresponding to those proteins identified by combined PMF and MS/MS search using MASCOT are indicated. Click here for file
