# Supplementary material for: Cytoplasmic- and extracellular-proteome analysis of Diplodia seriata: a phytopathogenic fungus involved in grapevine decline
Source: Proteome Sci. 2010 Sep 9;8:46. doi: 10.1186/1477-5956-8-46 (PMC2944164; doi:10.1186/1477-5956-8-46)

Spot 30 Hypothetical protein (Elongation factor 1 alpha)

Spectrum Label: E11_4 - Precursor: 1814.9329 **Peptide Sequence: SYLVGYASPKADVEC*R Score: 89.47**

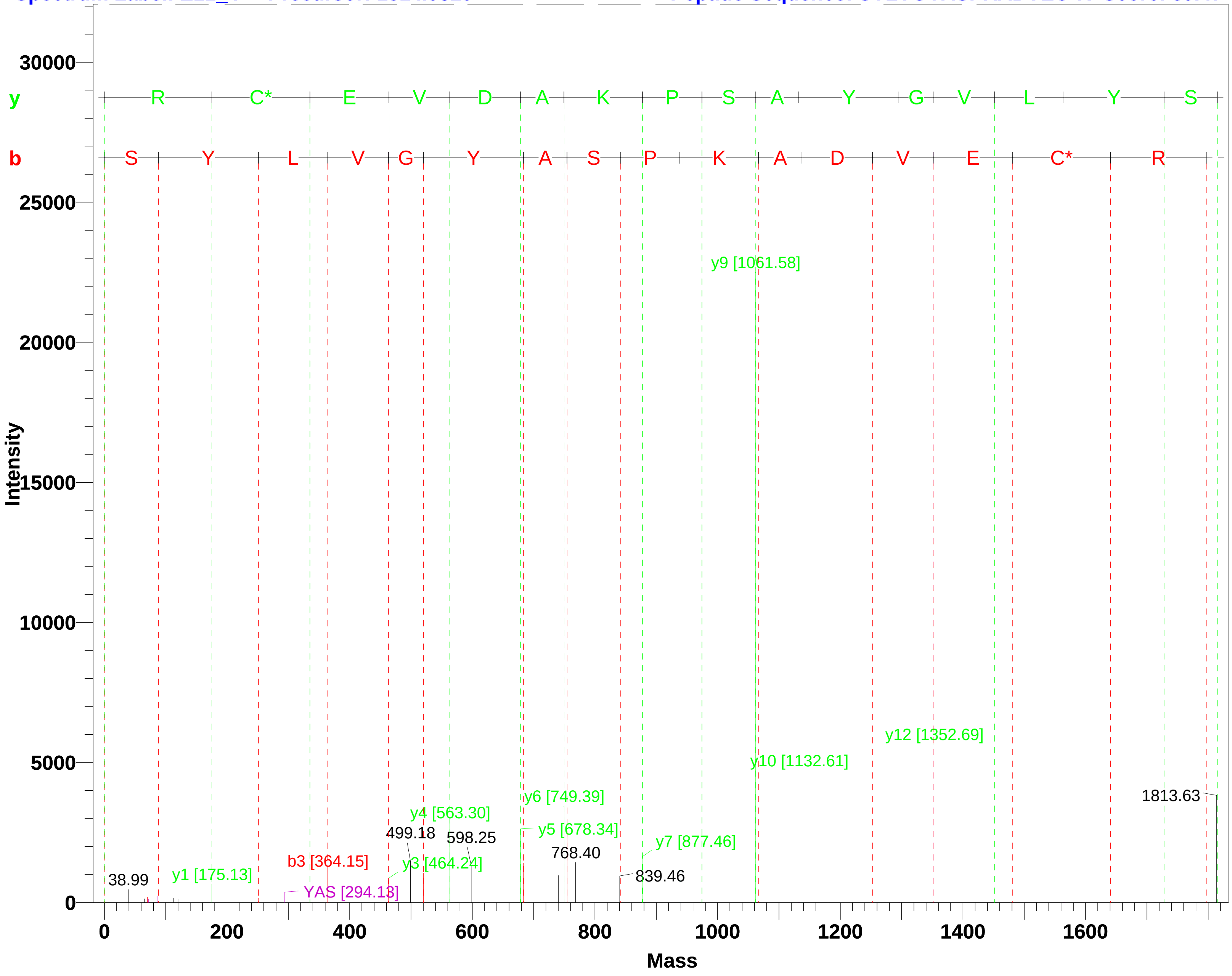

Spot 30 Hypothetical protein (Elongation factor 1 alpha)

3/12/2010 3:02:52 PM

Spectrum Label: E11_2 - Precursor: 1579.8392 **Peptide Sequence: RTAAPDAAKFPHAAR Score: 92.77**

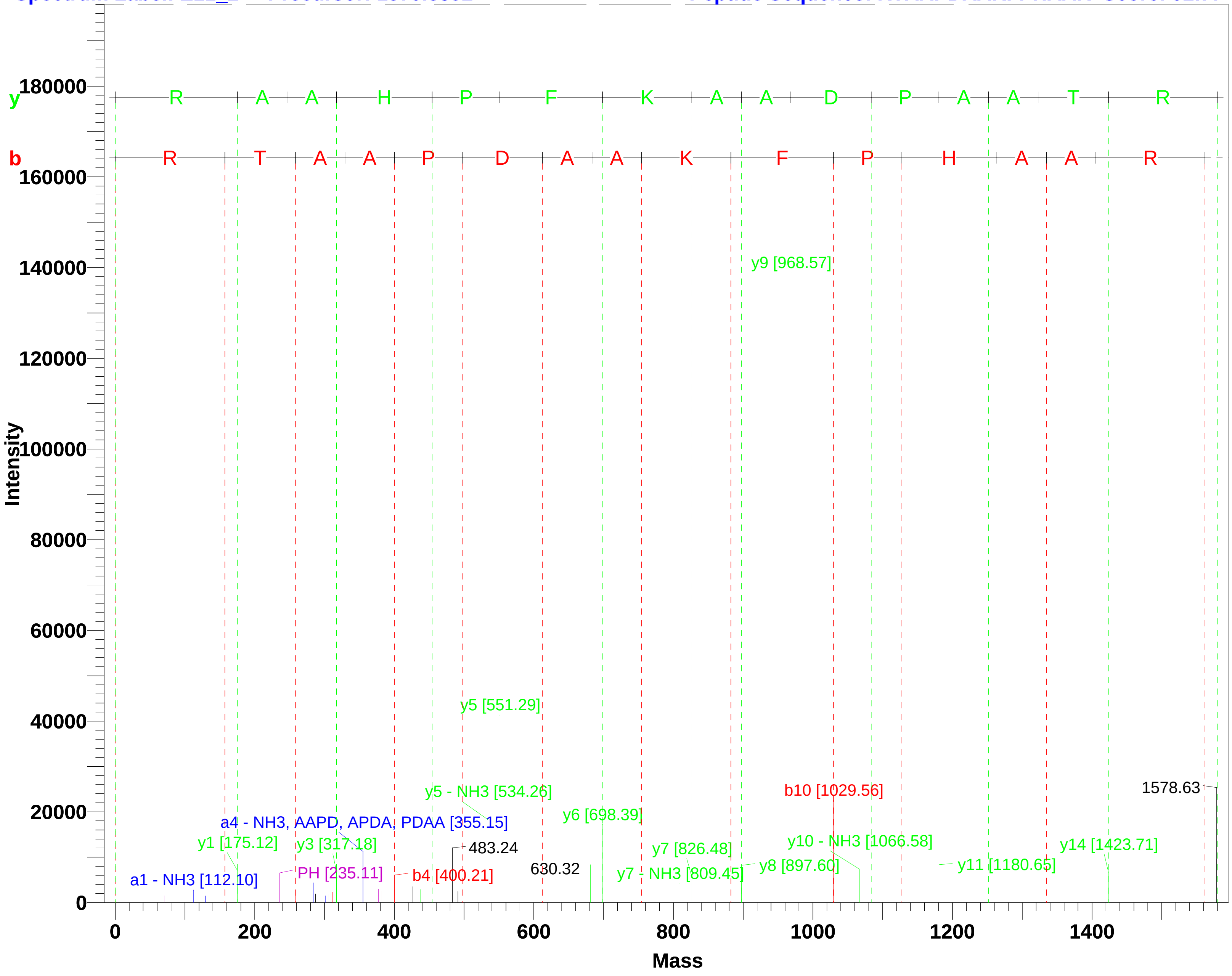

Spot 51 Pc12g14620 (flavohemoprotein)

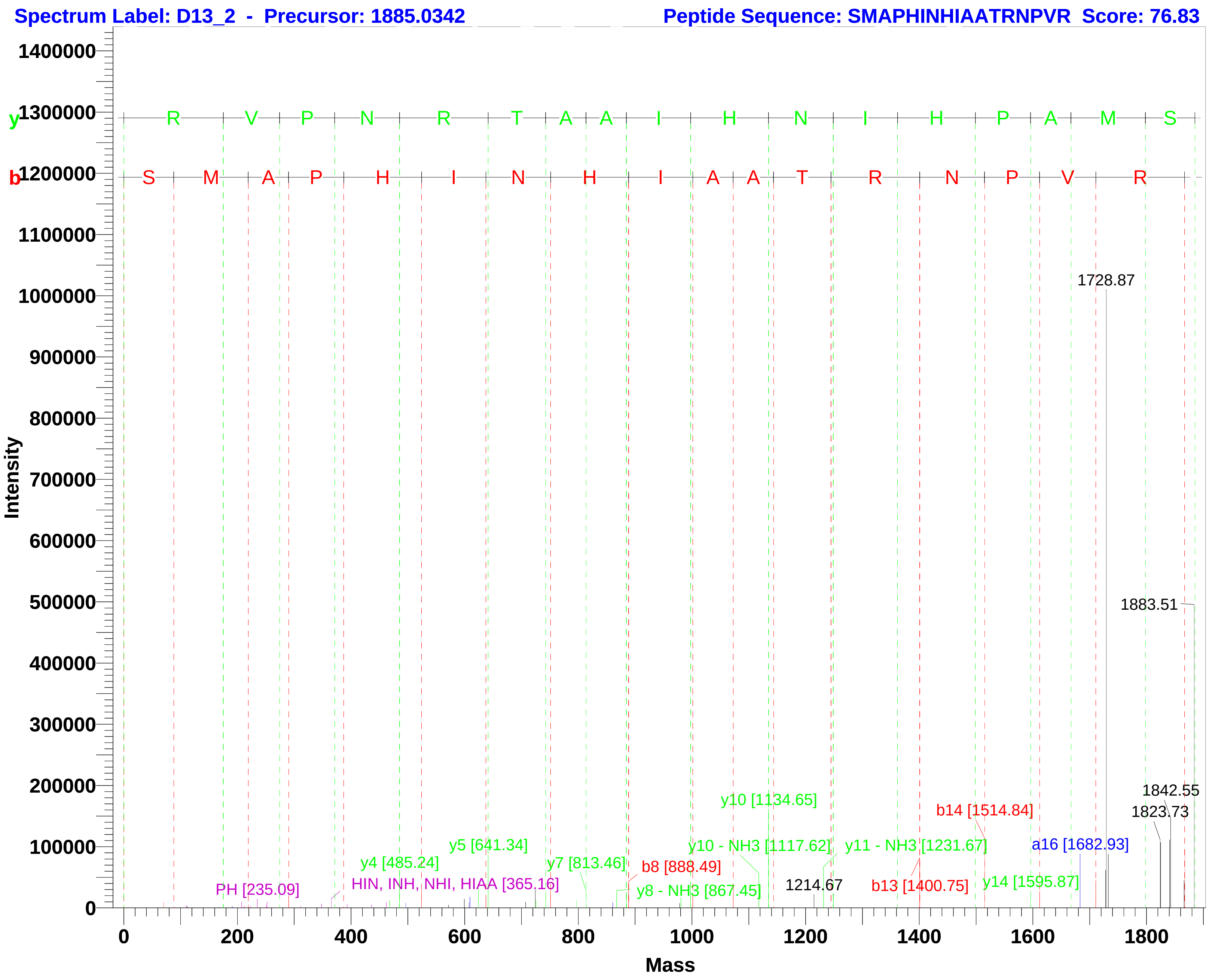

Spot 51 Pc12g14620 (flavohemoprotein)

Spectrum Label: D13_5 - Precursor: 1693.9253 **Peptide Sequence: RDPIIAEHGNTITTR Score: 82.72**

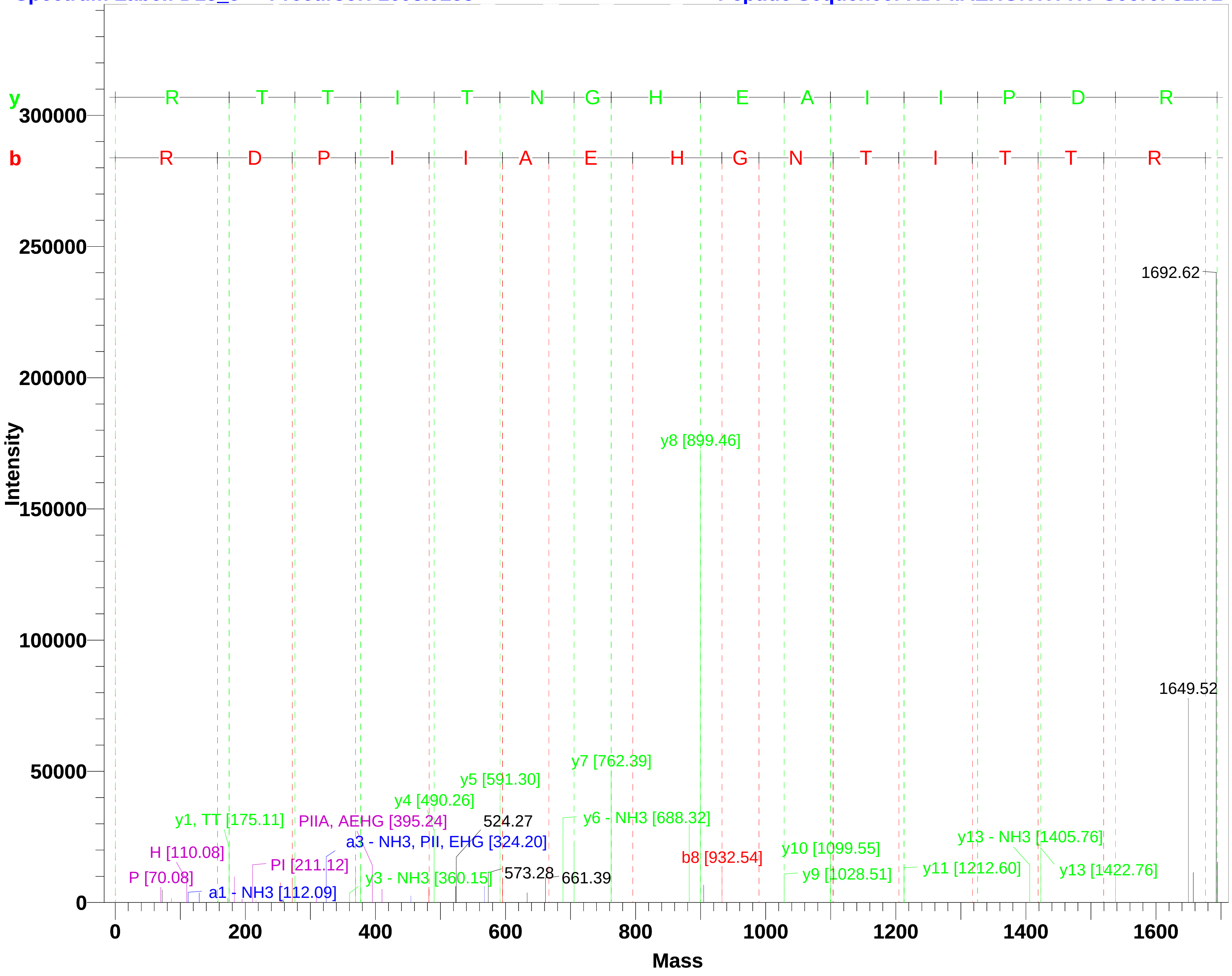

Spot 52 Aldose reductase

2/22/2010 3:11:00 PM

Spectrum Label: D5_6 - Precursor: 2350.1924 Peptide Sequence: DIGTDYVDLYLIHDAKKTGGR Score: 86.17

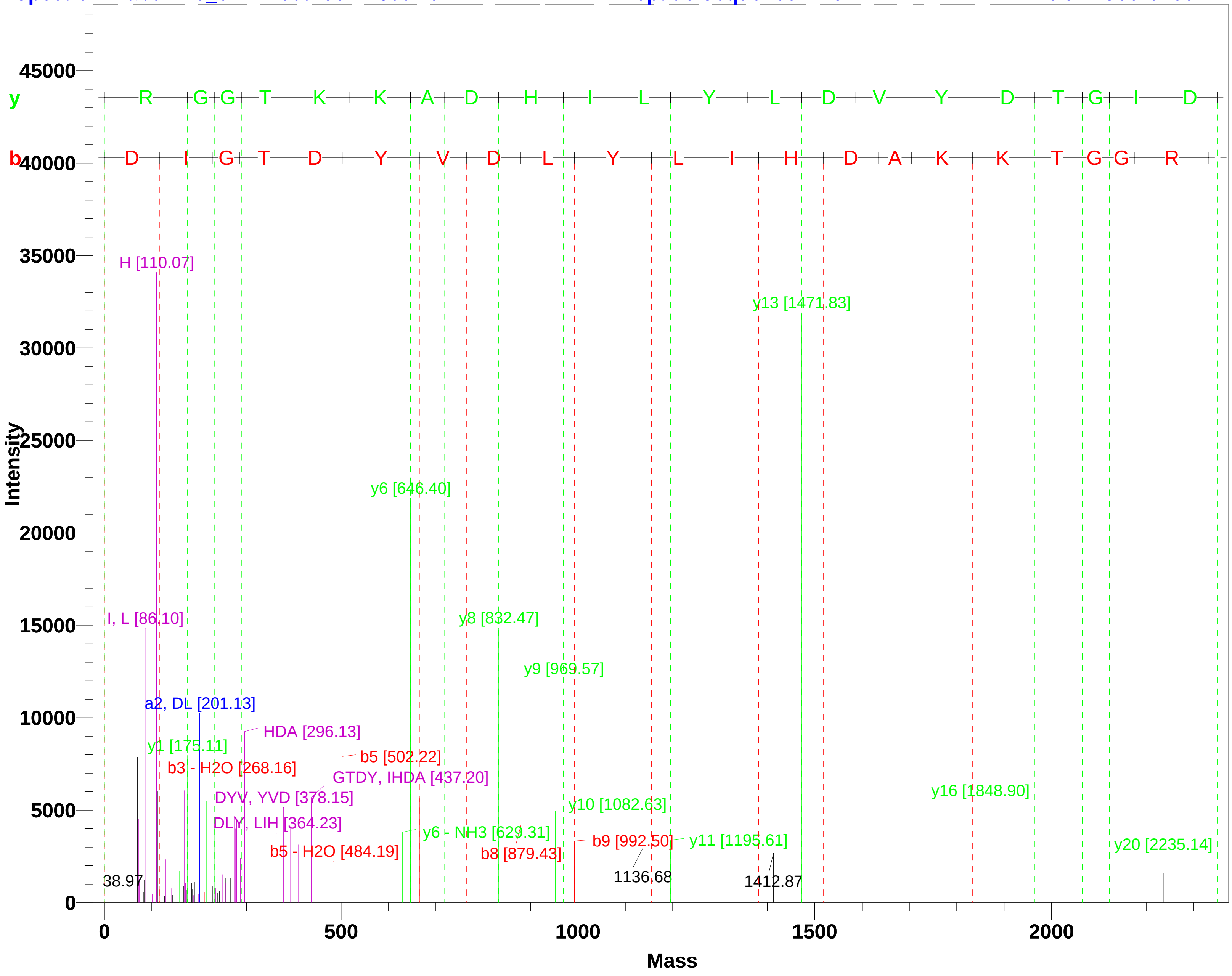

Spot 52 Aldose reductase

Spectrum Label: D5_4 - Precursor: 964.5203 **Peptide Sequence: ALGLSNFSR Score: 80.49**

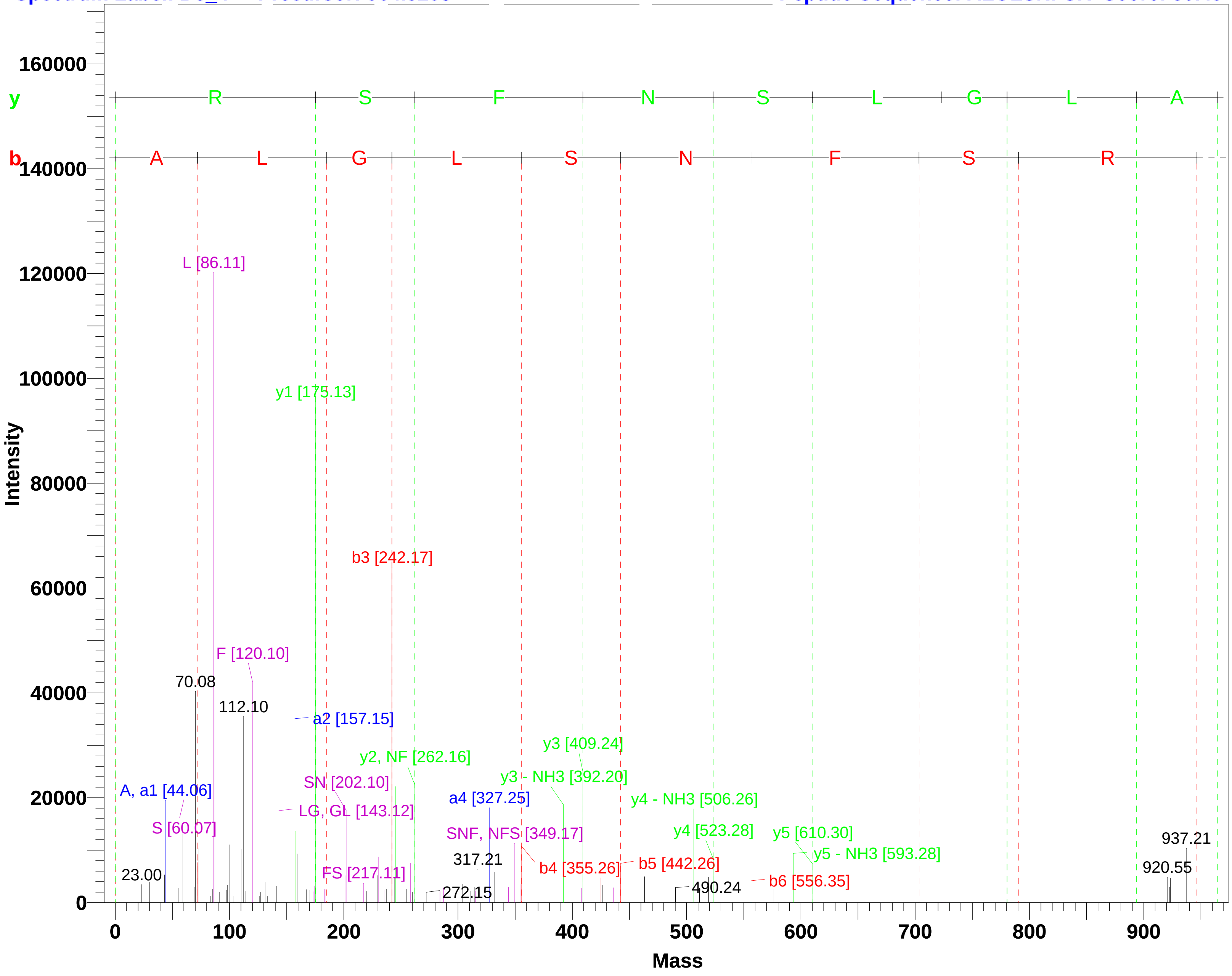

Spot 53 Alcohol dehydrogenase

Spectrum Label: D9_8 - Precursor: 1073.6525 Peptide Sequence: KIVHPPKVR Score: 86...

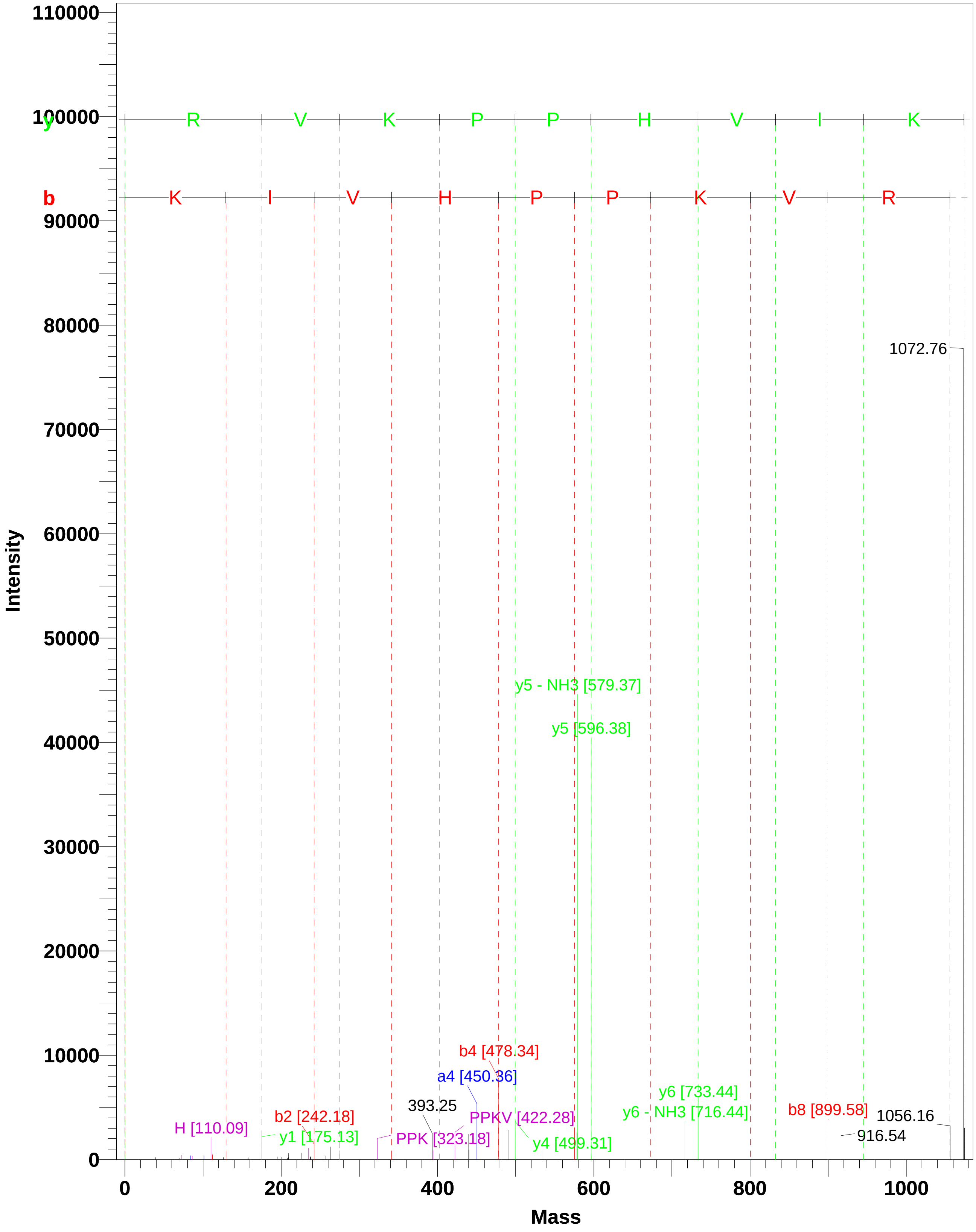

Spot 53 Alcohol dehydrogenase

Spectrum Label: D9_2 - Precursor: 1587.7560 **Peptide Sequence: EFPANEDHIAFAAR Score: 91.38**

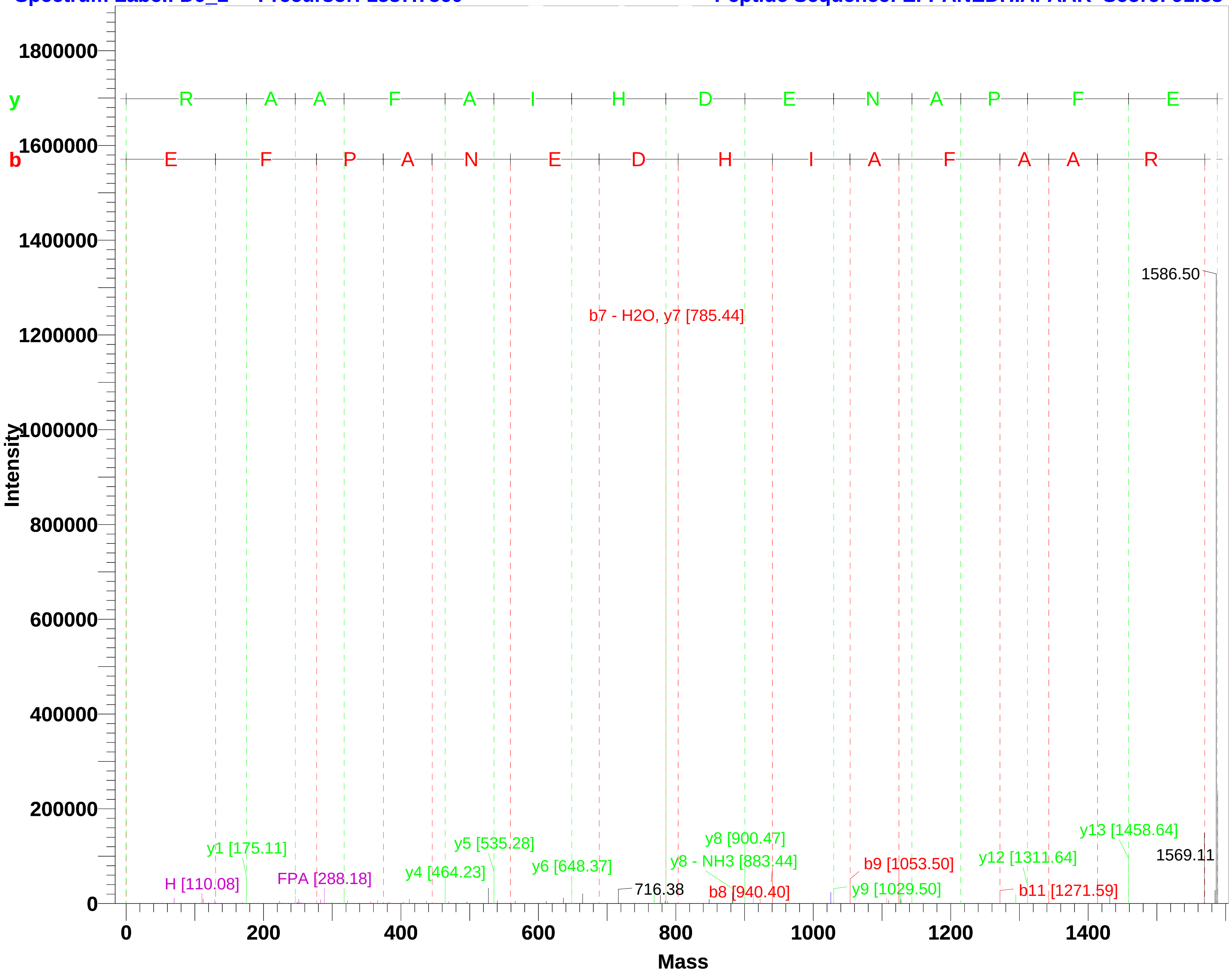

Supplement: Additional file 1 — Cytoplasmic proteins identified by means of PRO-BLAST search. Fragmentation spectra obtained from cytoplasmic proteins after de novo sequencing using ProBLAST software and properly identified by BLAST similarity searching are showed. [file 1477-5956-8-46-S1.PDF]
