# Supplementary material for: Cytoplasmic- and extracellular-proteome analysis of Diplodia seriata: a phytopathogenic fungus involved in grapevine decline
Source: Proteome Sci. 2010 Sep 9;8:46. doi: 10.1186/1477-5956-8-46 (PMC2944164; doi:10.1186/1477-5956-8-46)

Spot S2 Pc13g02210

Spectrum Label: D9_5 - Precursor: 1211.6324 **Peptide Sequence: KIIYPAYTDK Score: 91.20**

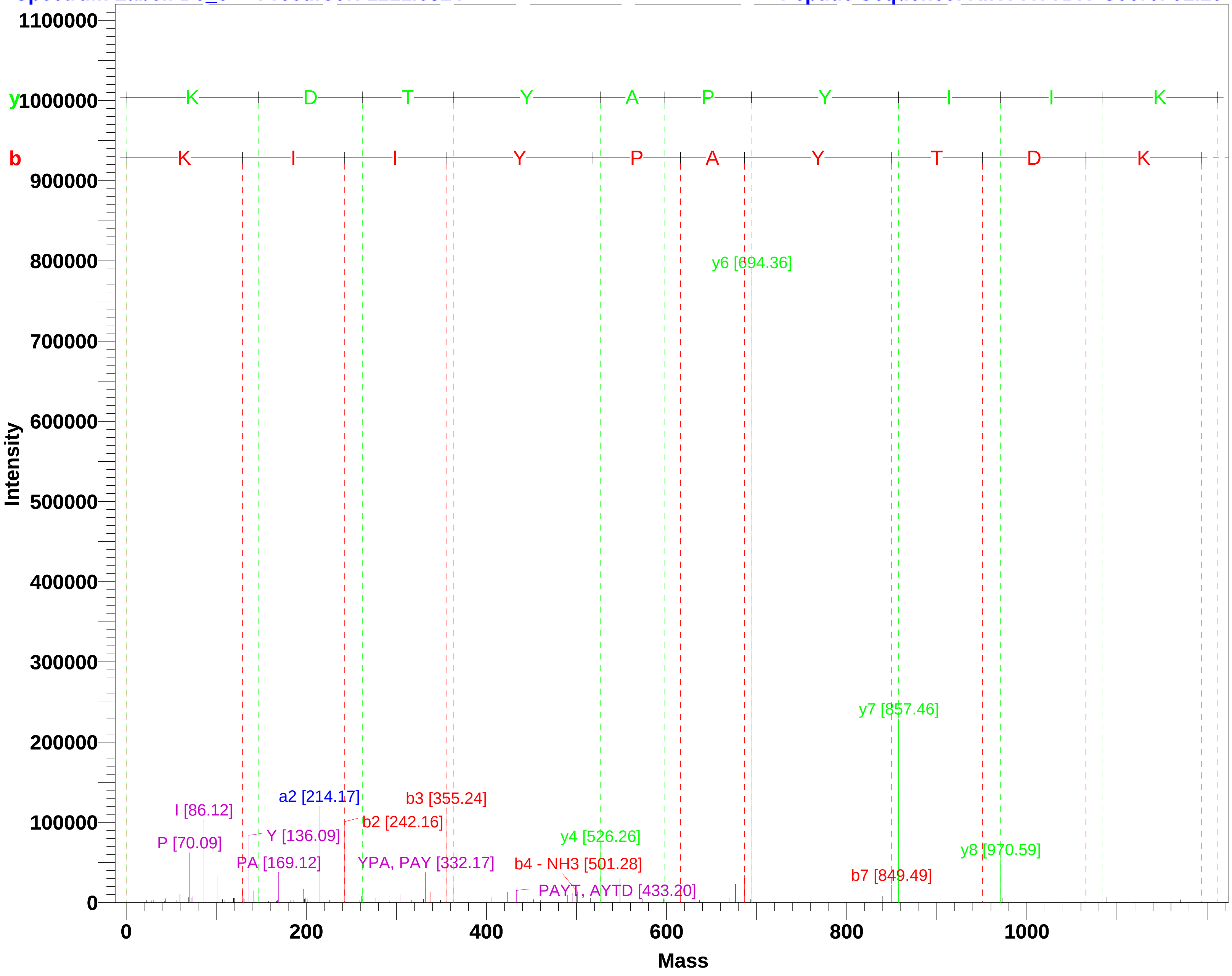

Spot S2 Pc13g02210

Spectrum Label: D9_4 - Precursor: 2153.1309 **Peptide Sequence: ARIKGDVVKPDKSYAPKAIP Score: 60.38**

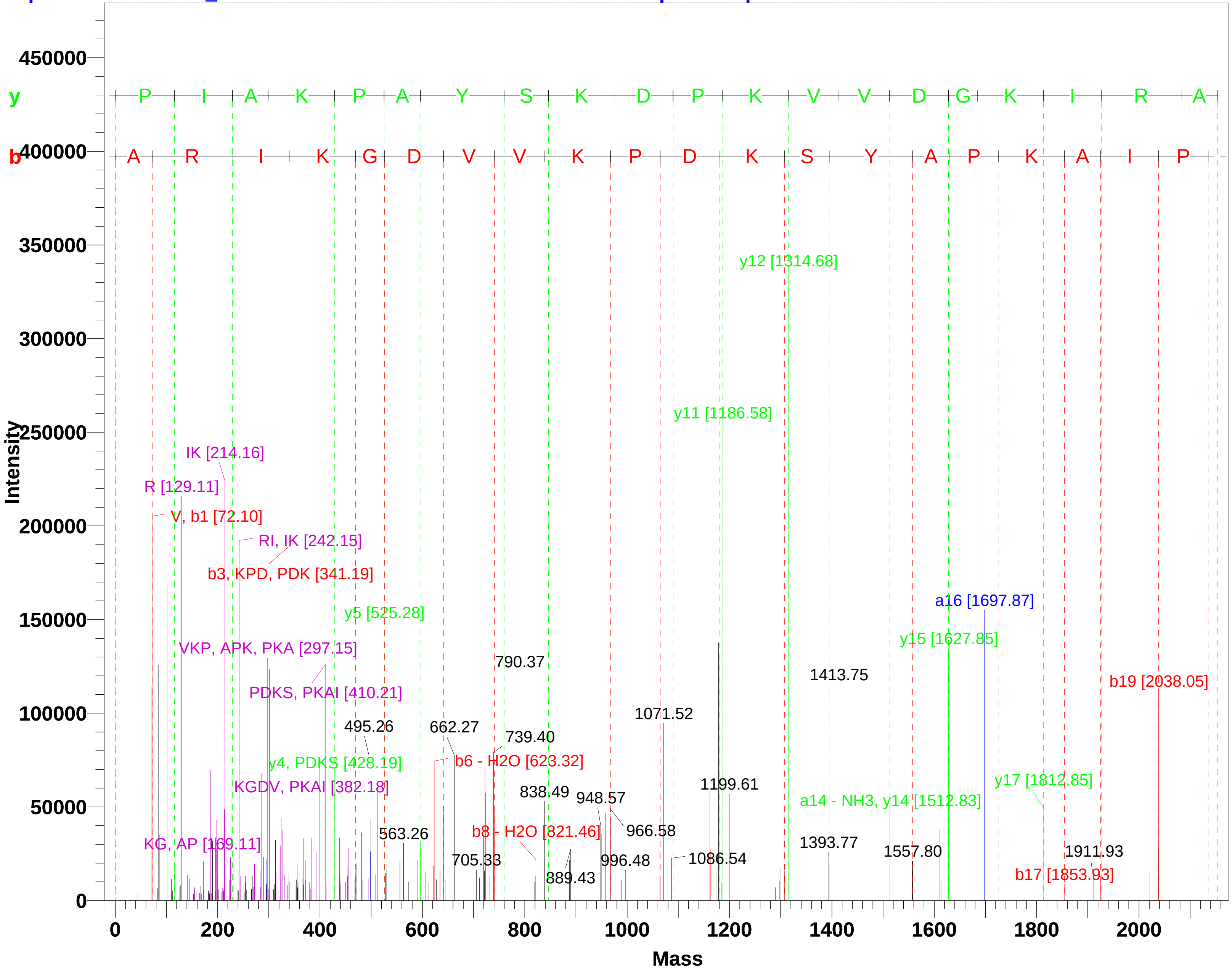

Spot S4 glucosidase

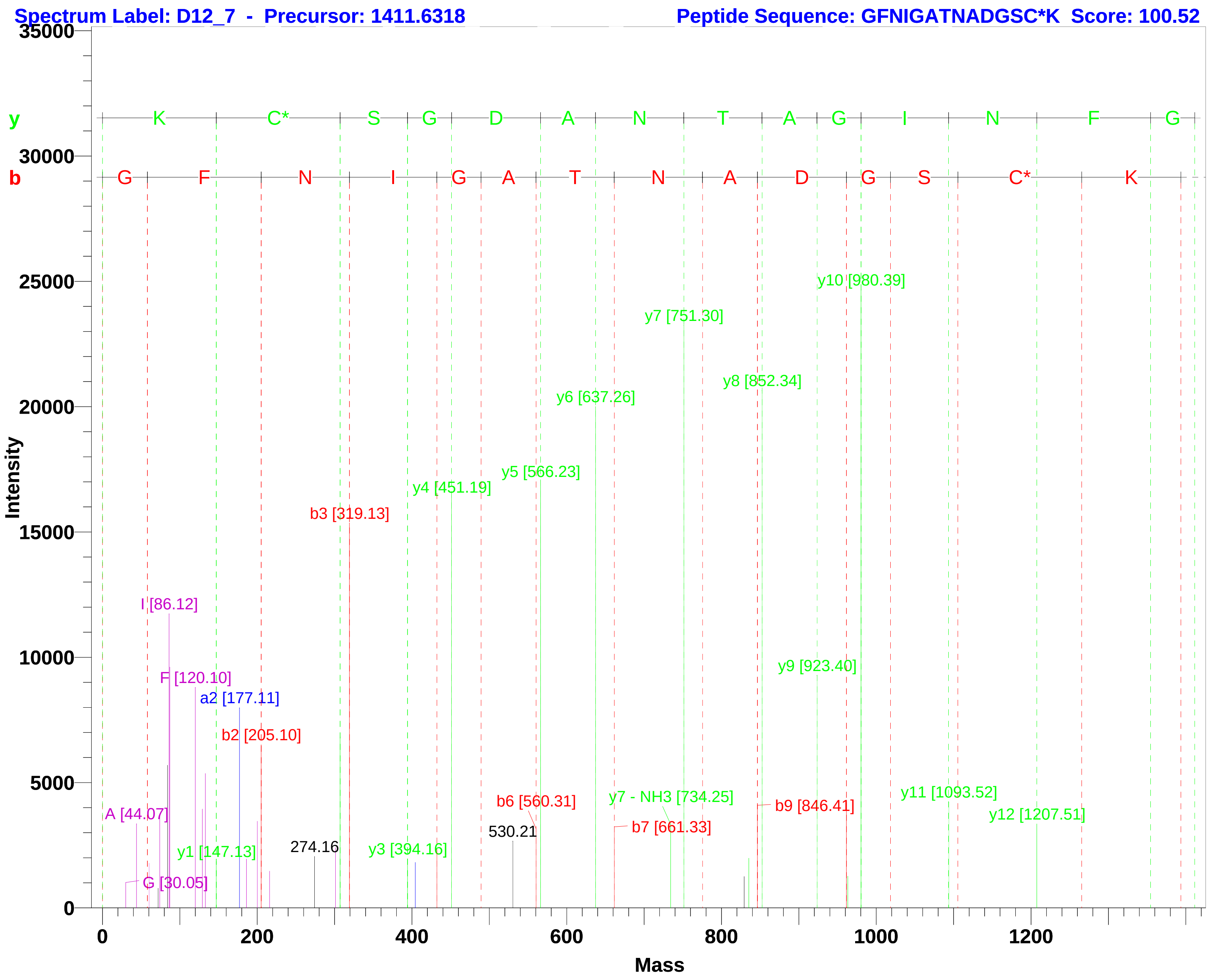

Spot S4 glucosidase

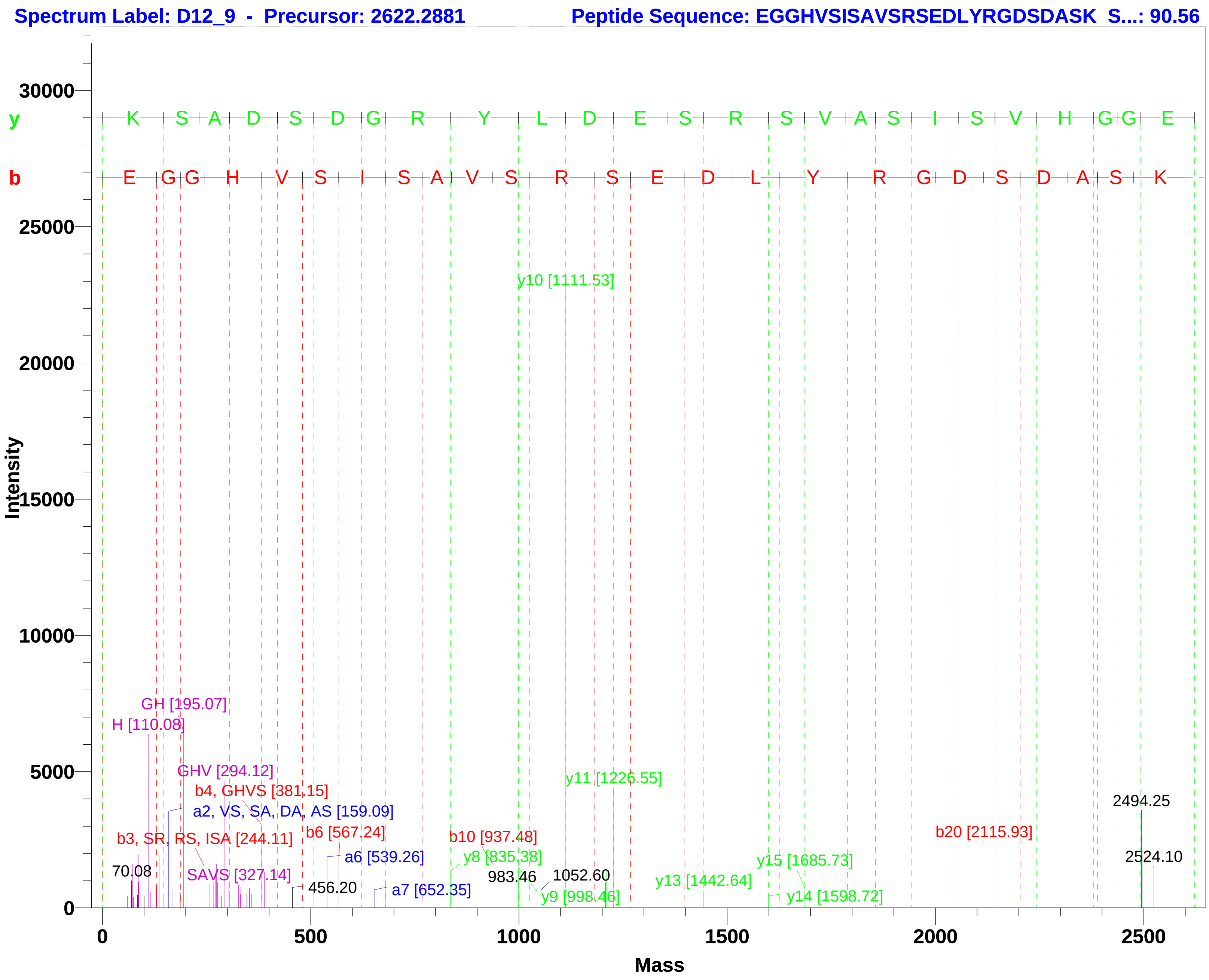

Spot S5 Predicted protein (necrosis and ethylene inducing peptide 2 precursor)

Spectrum Label: D15_2 - Precursor: 2657.4148 Peptide Sequence: AVLAHDAVVGFAETVPFVGVWHAHK ...: 84.60

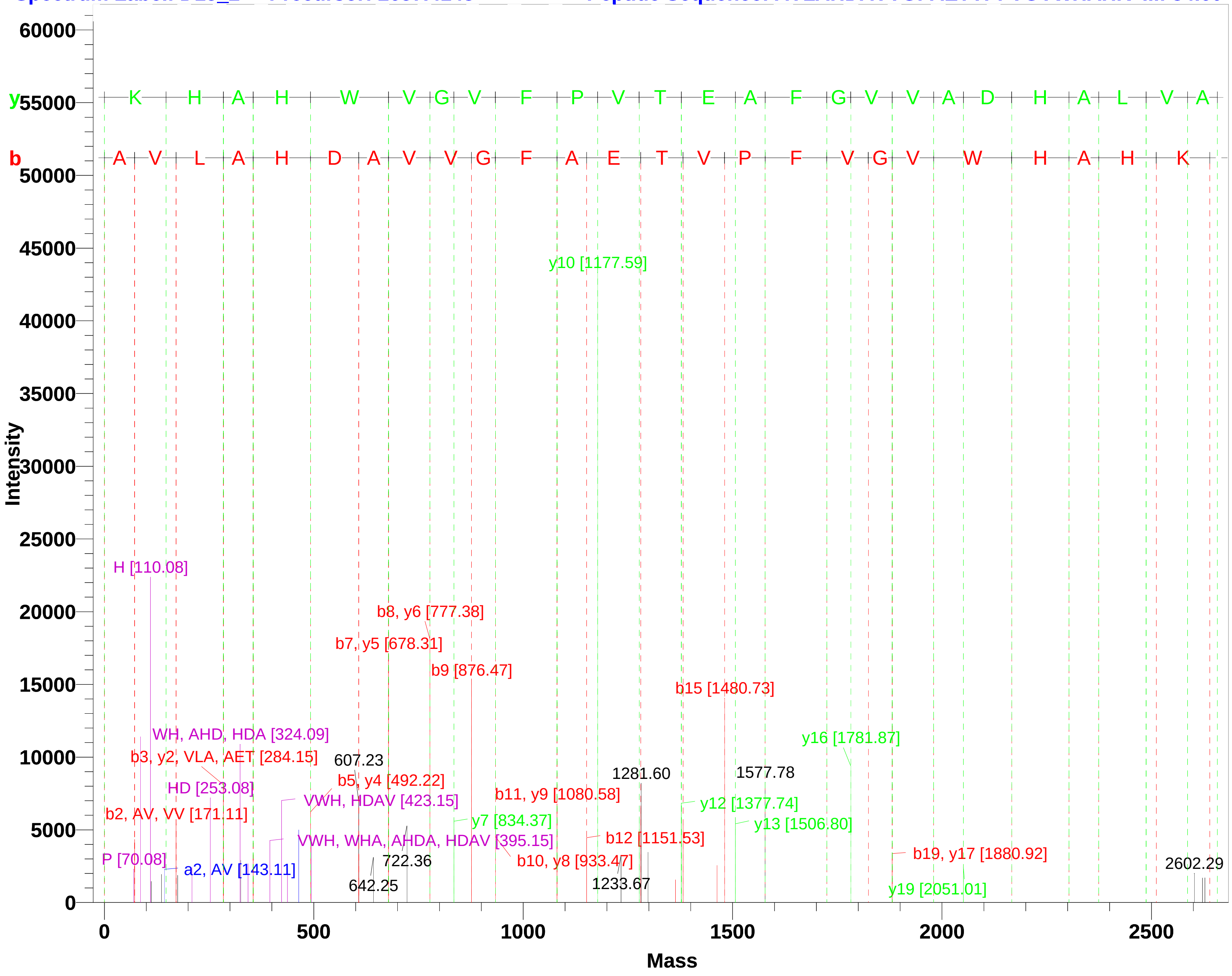

Spot S5 Predicted protein (necrosis and ethylene inducing peptide 2 precursor)

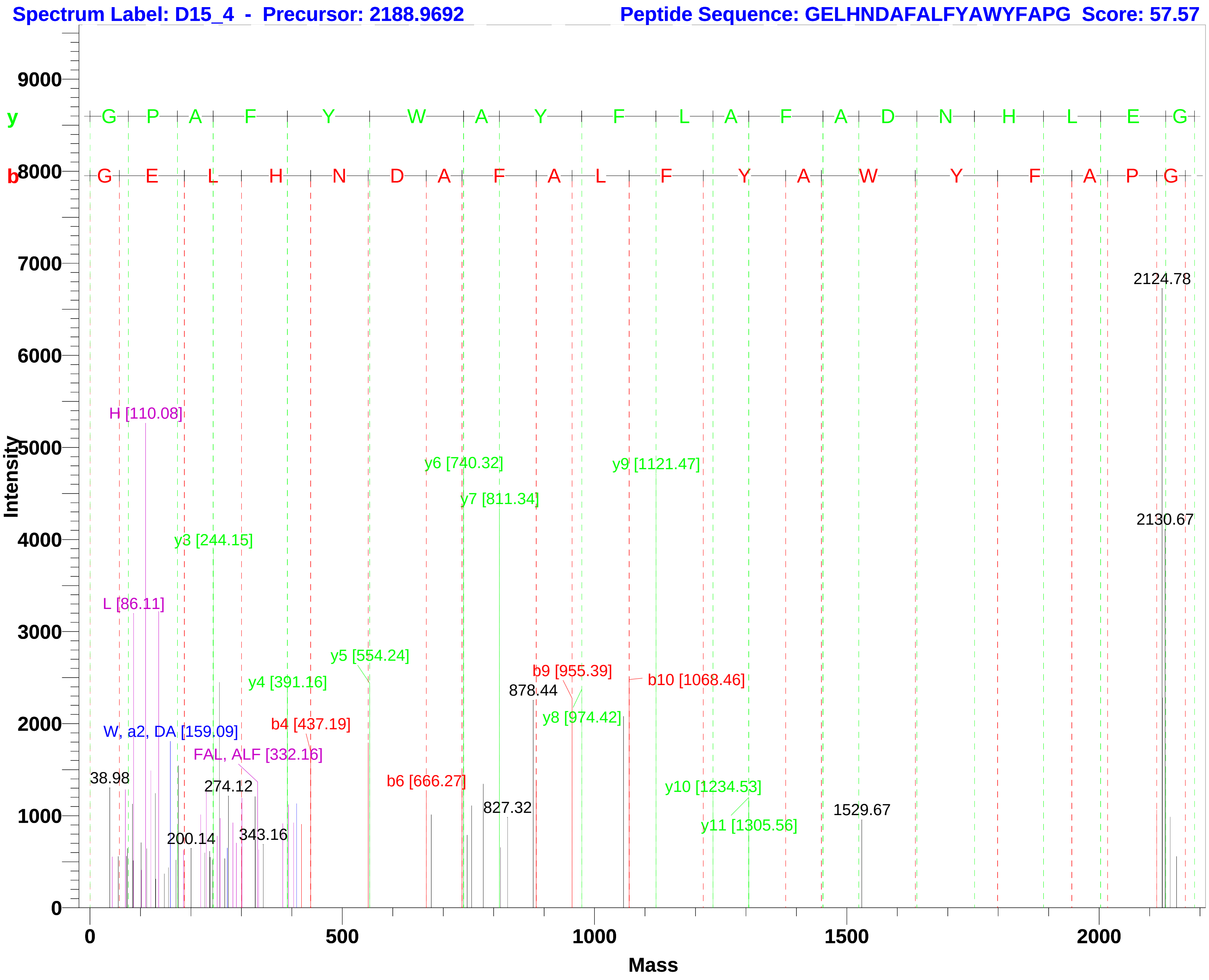

Spot S6 secreted protein

Spectrum Label: D16_4 - Precursor: 1936.8666 **Peptide Sequence: SSSFPHWLTLSGSC*NTR Score: 86.85**

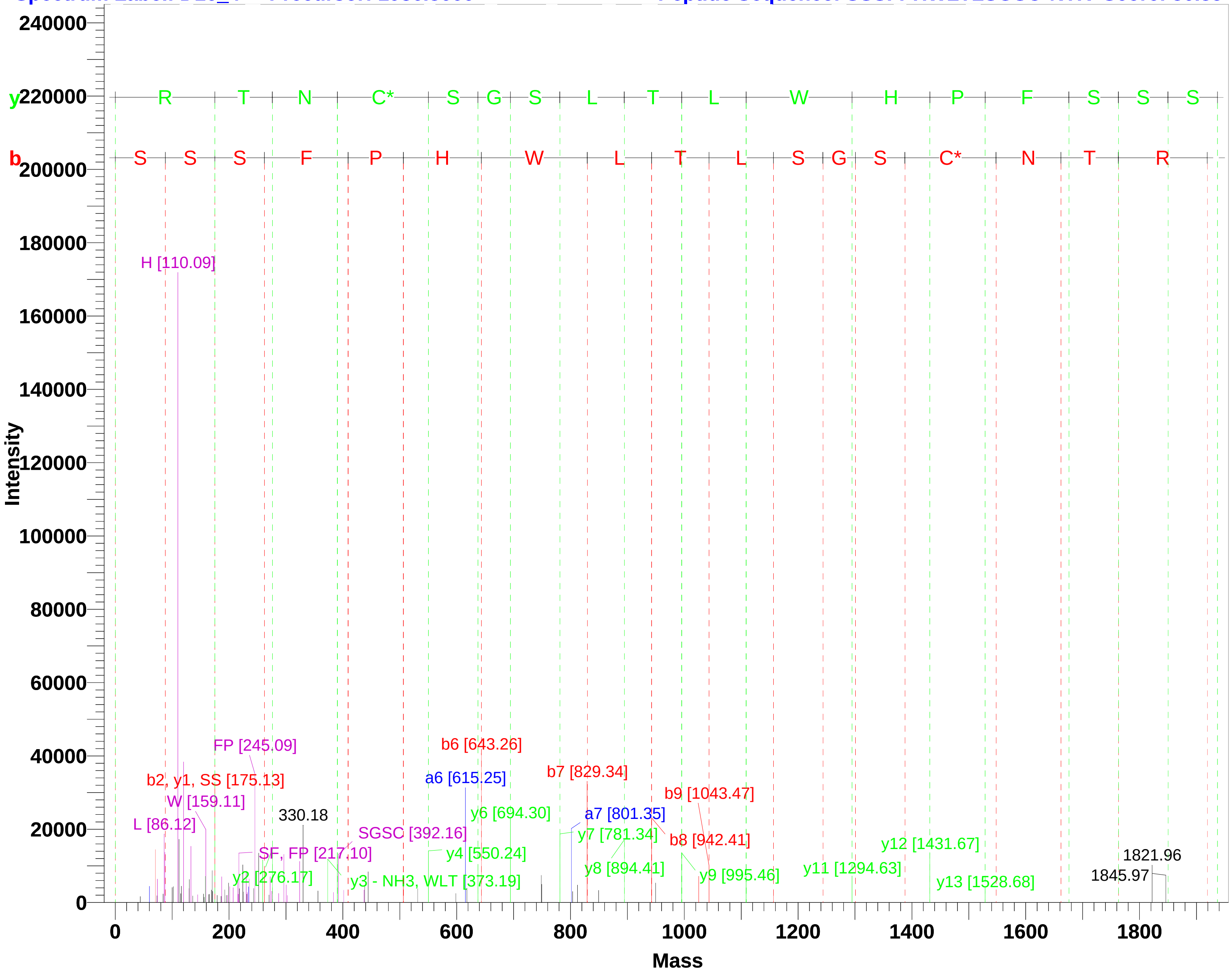

Spot S6 secreted protein

Spectrum Label: D16_6 - Precursor: 993.4746 **Peptide Sequence: SGASSWTAAR Score: 63.67**

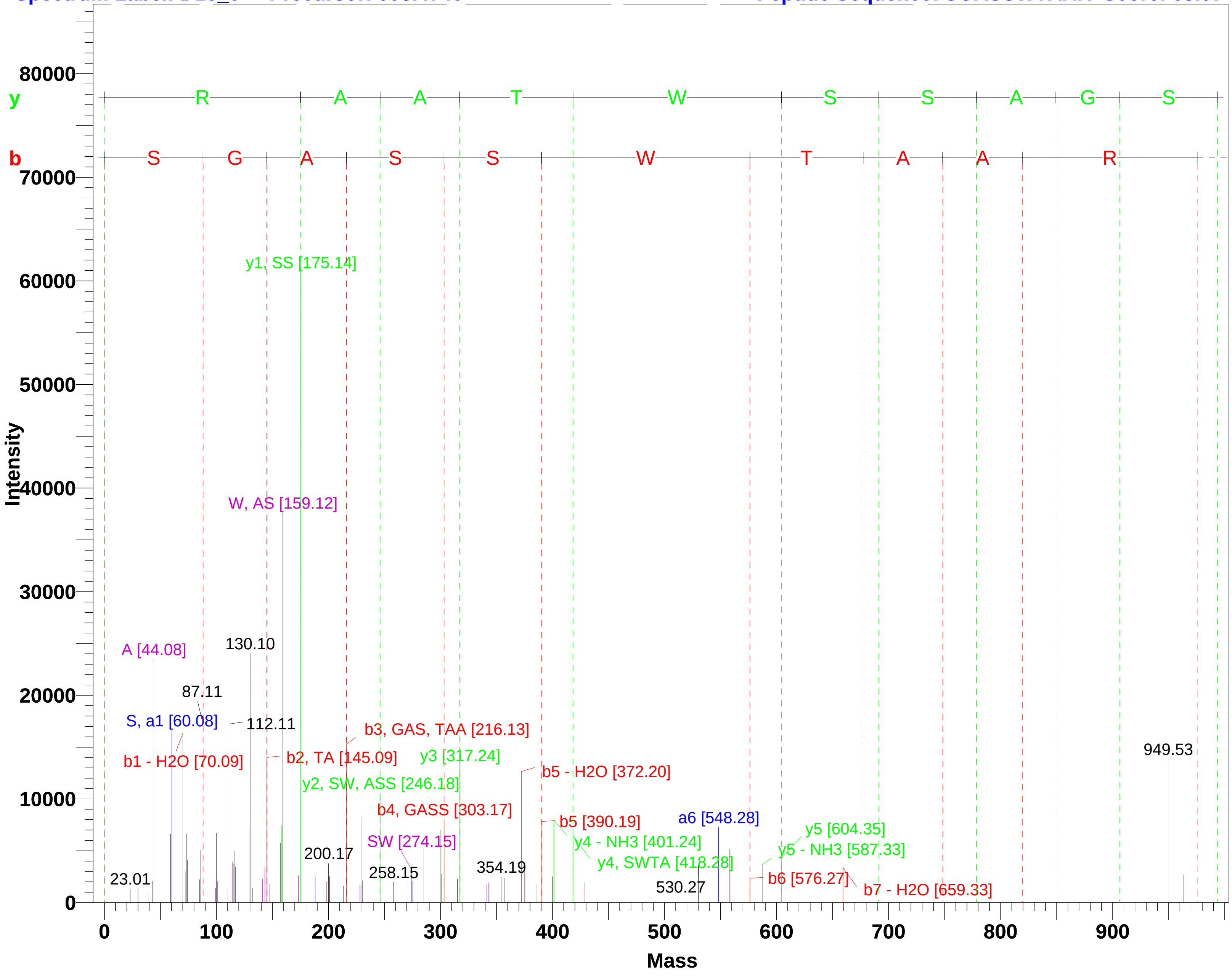

Spot S8 Hypothetical protein (necrosis and ethylene inducing protein 1)

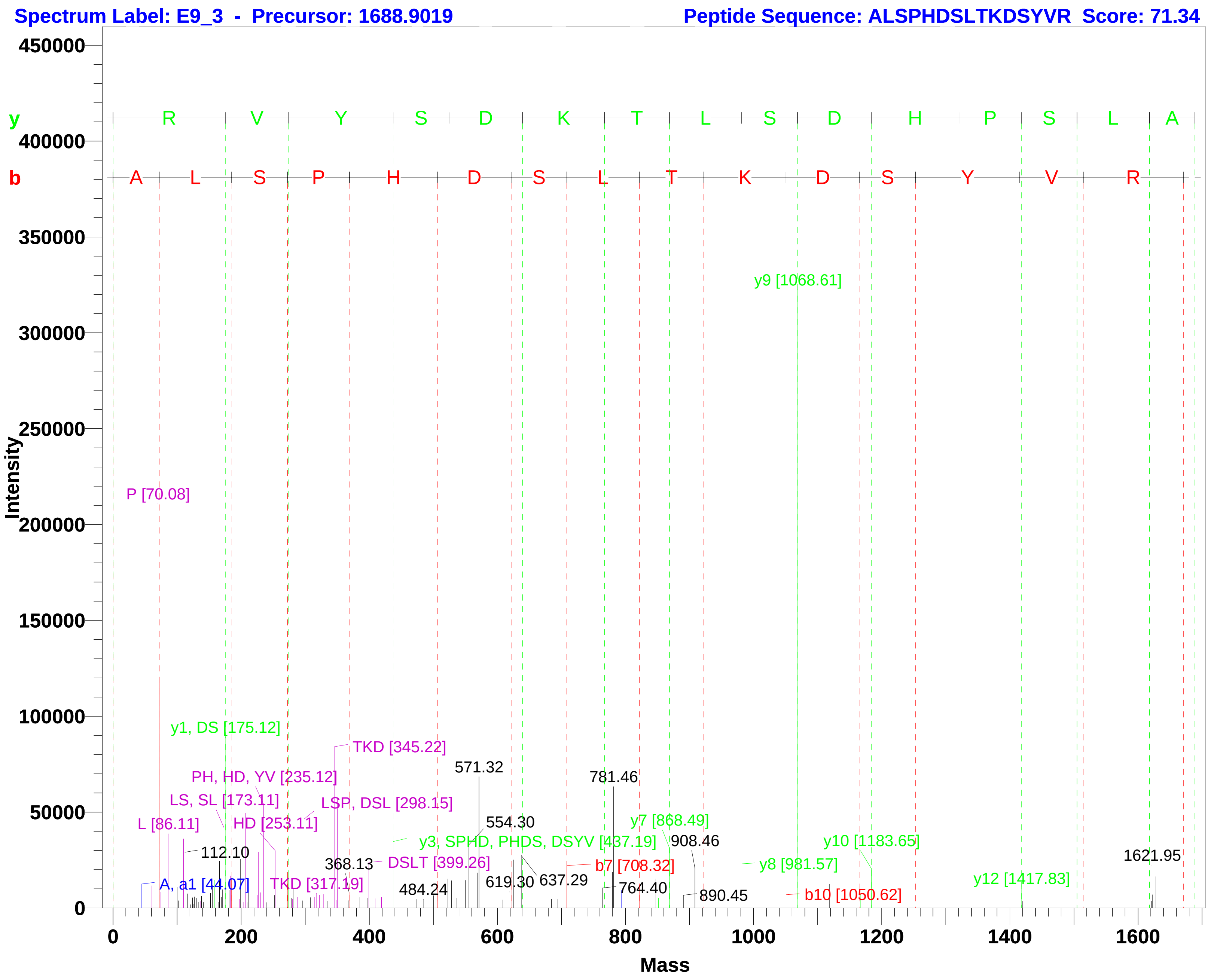

Spot S8 Hypothetical protein (necrosis and ethylene inducing protein 1)

Spectrum Label: E9_7 - Precursor: 1438.6901 **Peptide Sequence: FGLFYAWYFPK Score: 52.23**

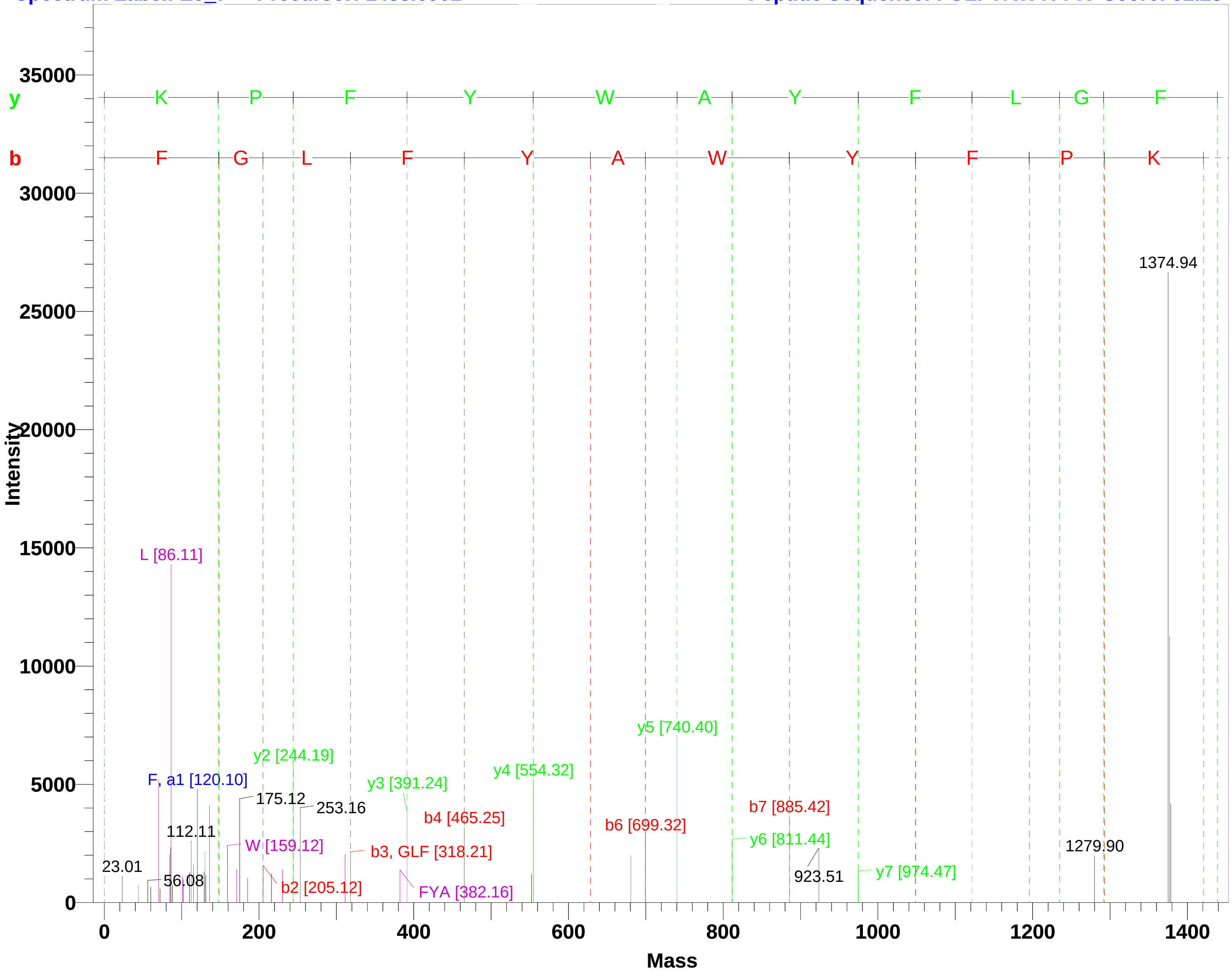

Spectrum Label: D8_6 - Precursor: 3647.6943 Peptide Sequence: KC*FPTEEDRRSGVDVYYVVDTGLYTAHSEF...: 96.82

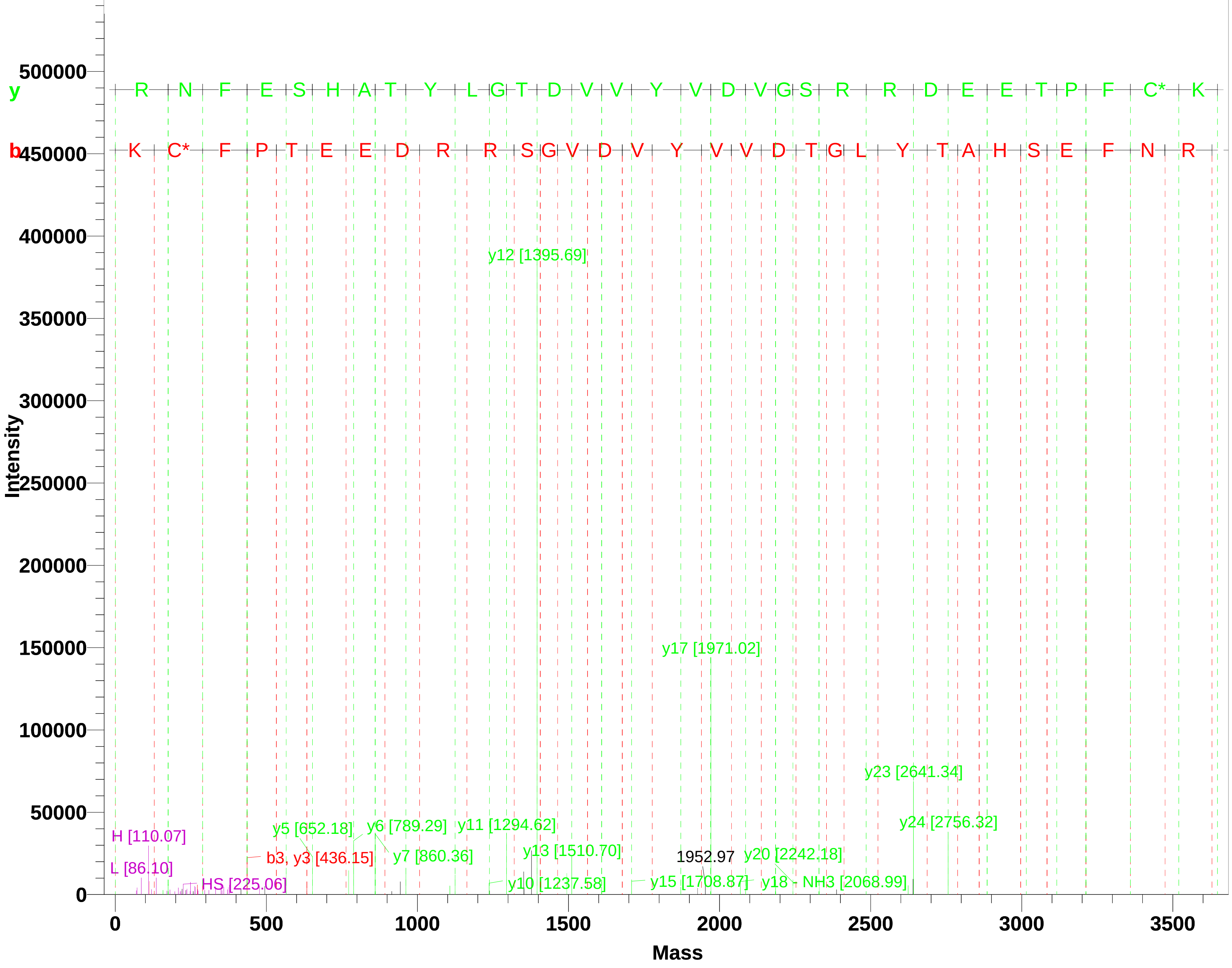

Spot S12 Peptidase 1

Spectrum Label: D8_9 - Precursor: 2089.0500 **Peptide Sequence: KTSGNFIASVASMSLGFESR Score: 89.09**

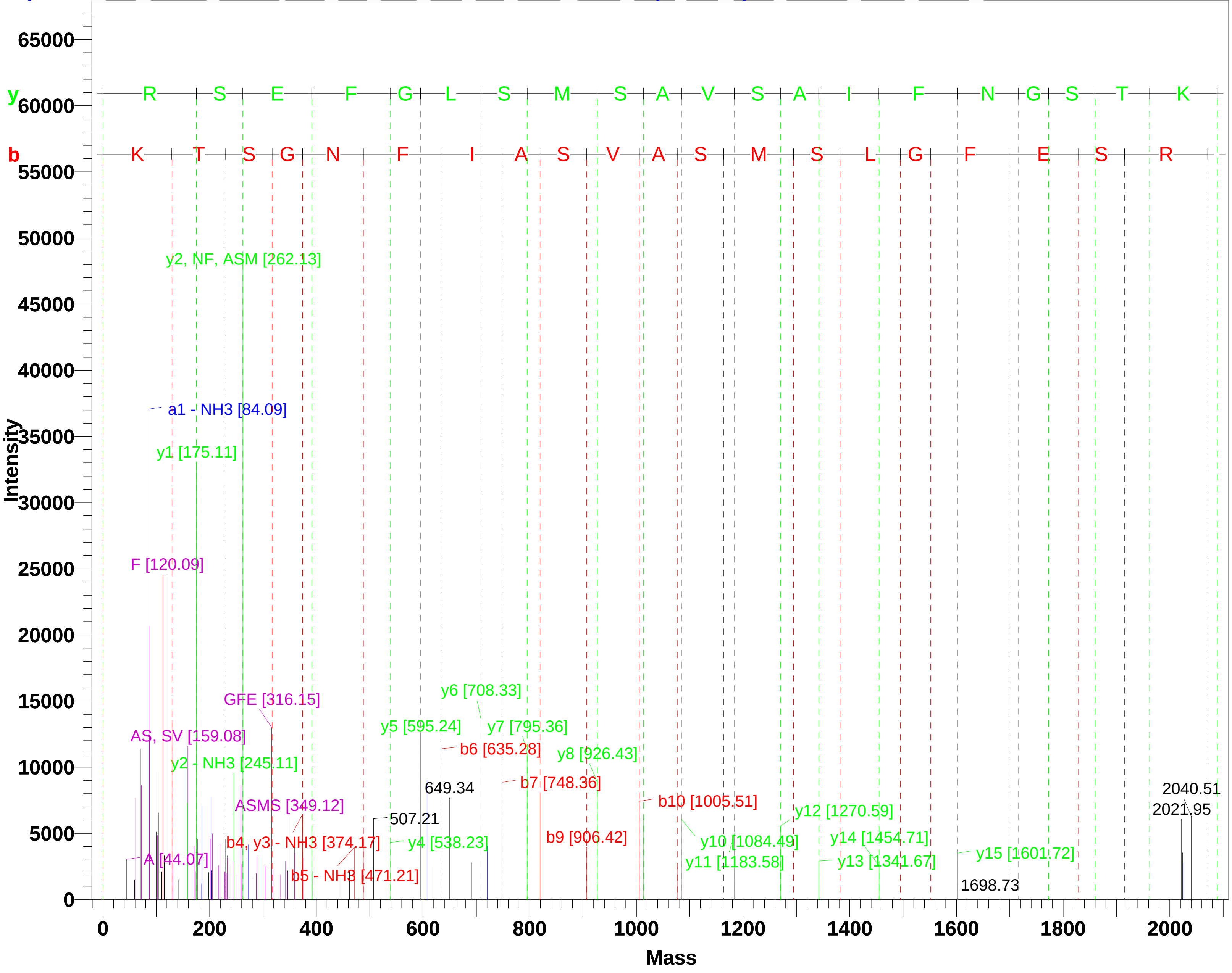

Spot S13 Pc22g00190 (cell wall protein PhiA)

Spectrum Label: D11_2 - Precursor: 1419.7527 **Peptide Sequence: KGIGYTTGAEPTPK Score: 96.82**

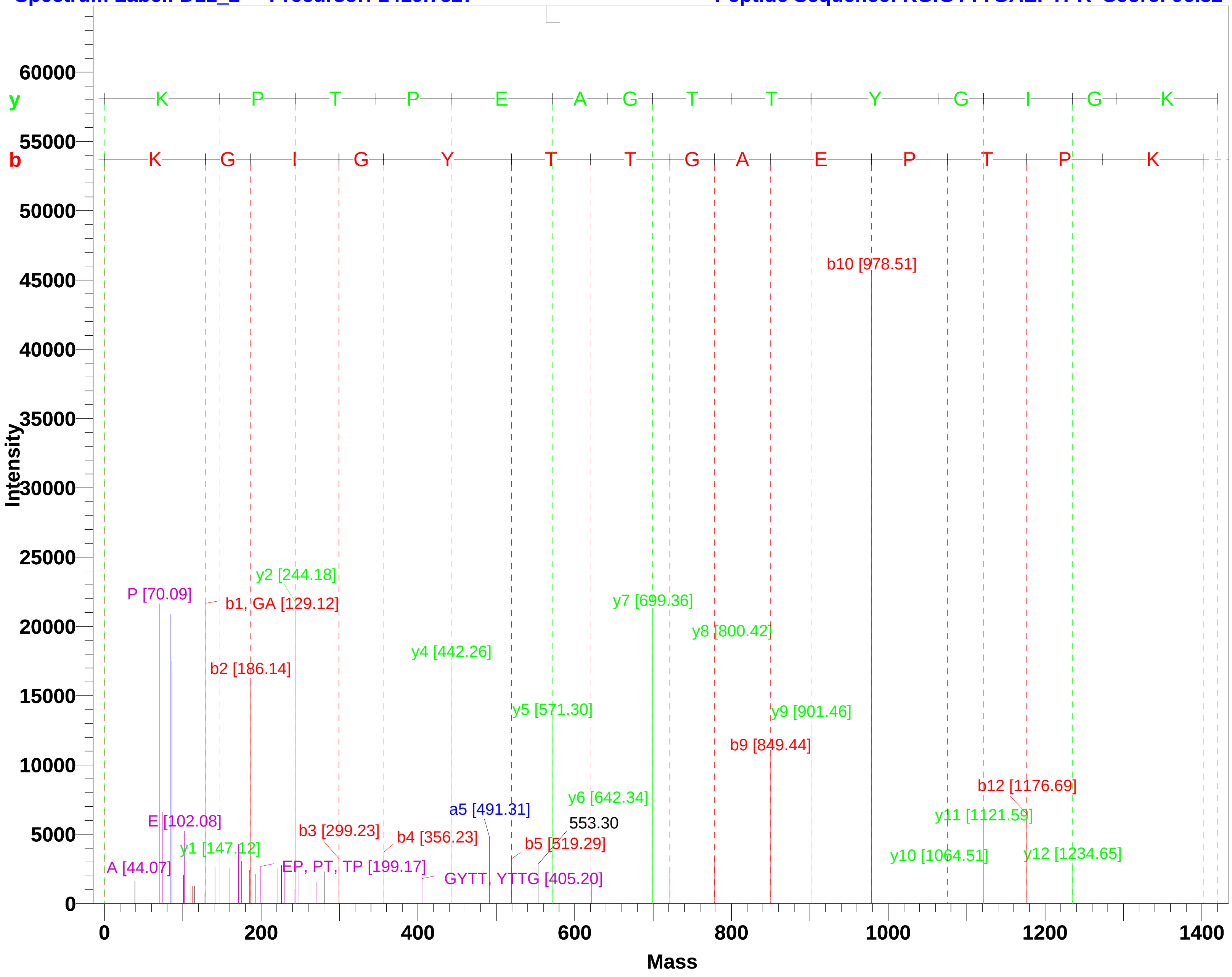

Spot S13 Pc22g00190 (cell wall protein PhiA)

Spectrum Label: D11_3 - Precursor: 1277.6554 **Peptide Sequence: ASTPKKLYVDR Score: 75.76**

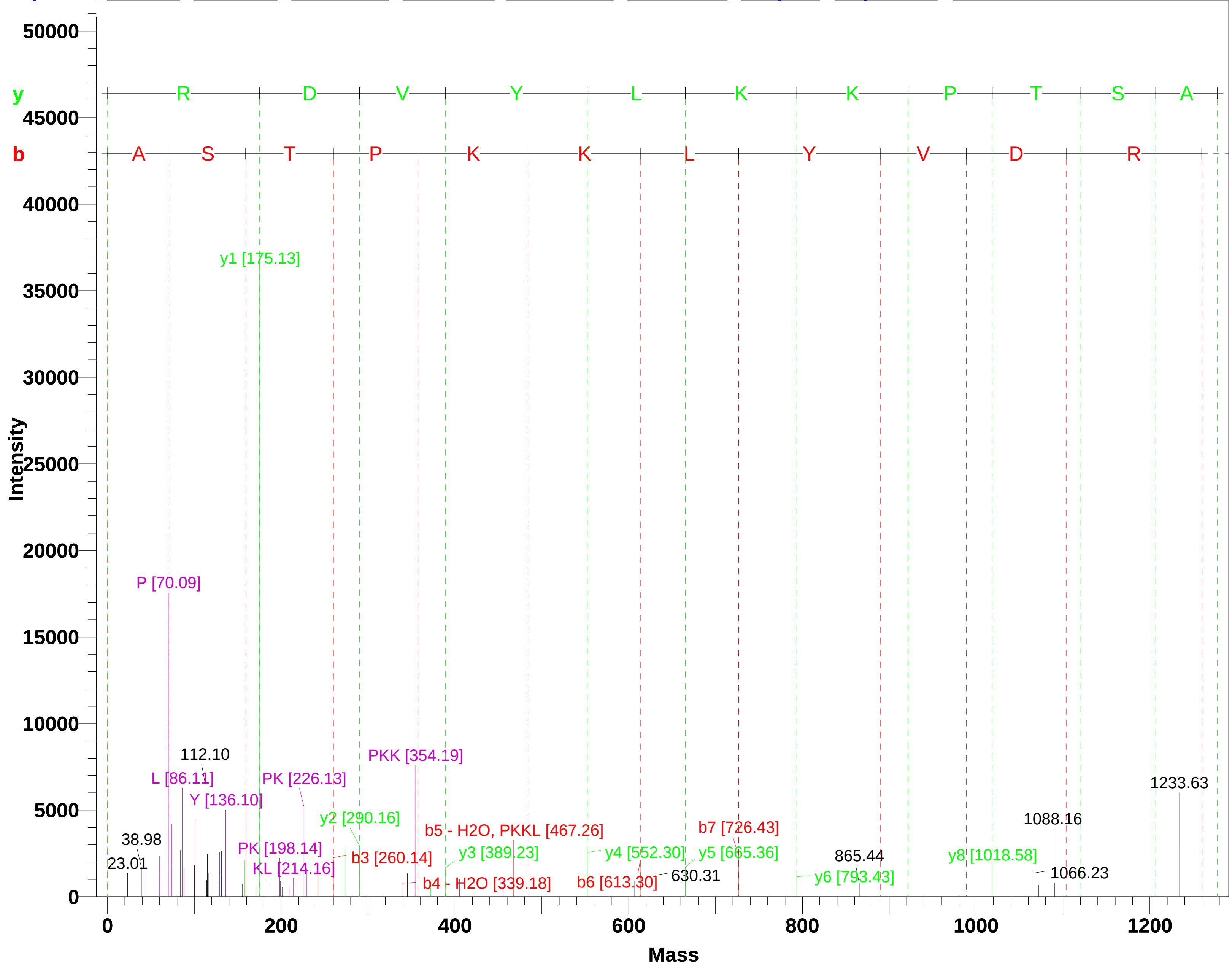

Spot S14 Hypothetical protein

Spectrum Label: E13_7 - Precursor: 1557.7471 **Peptide Sequence: FGHWSPNASLATAAK Score: 90.87**

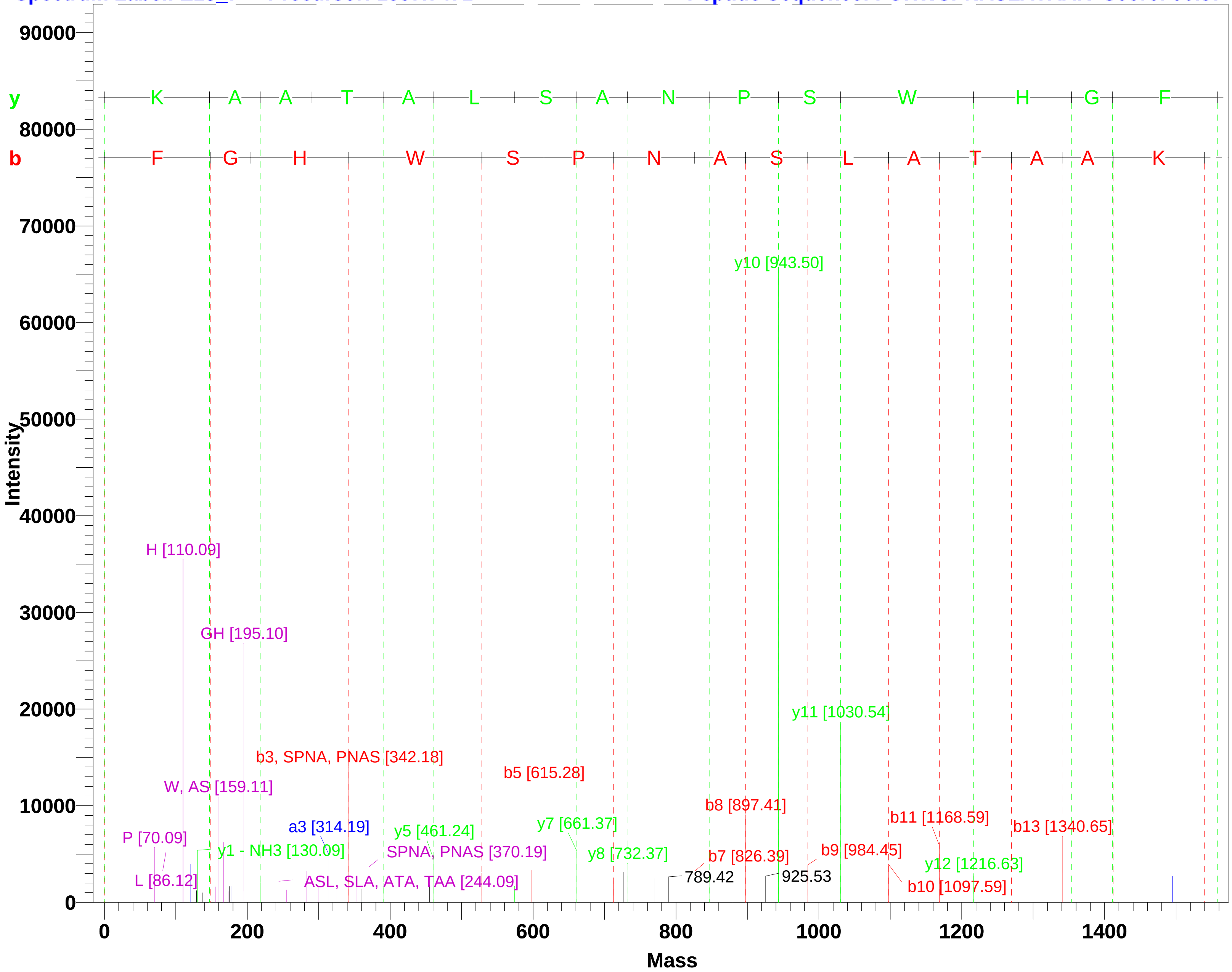

Spot S14 Hypothetical protein

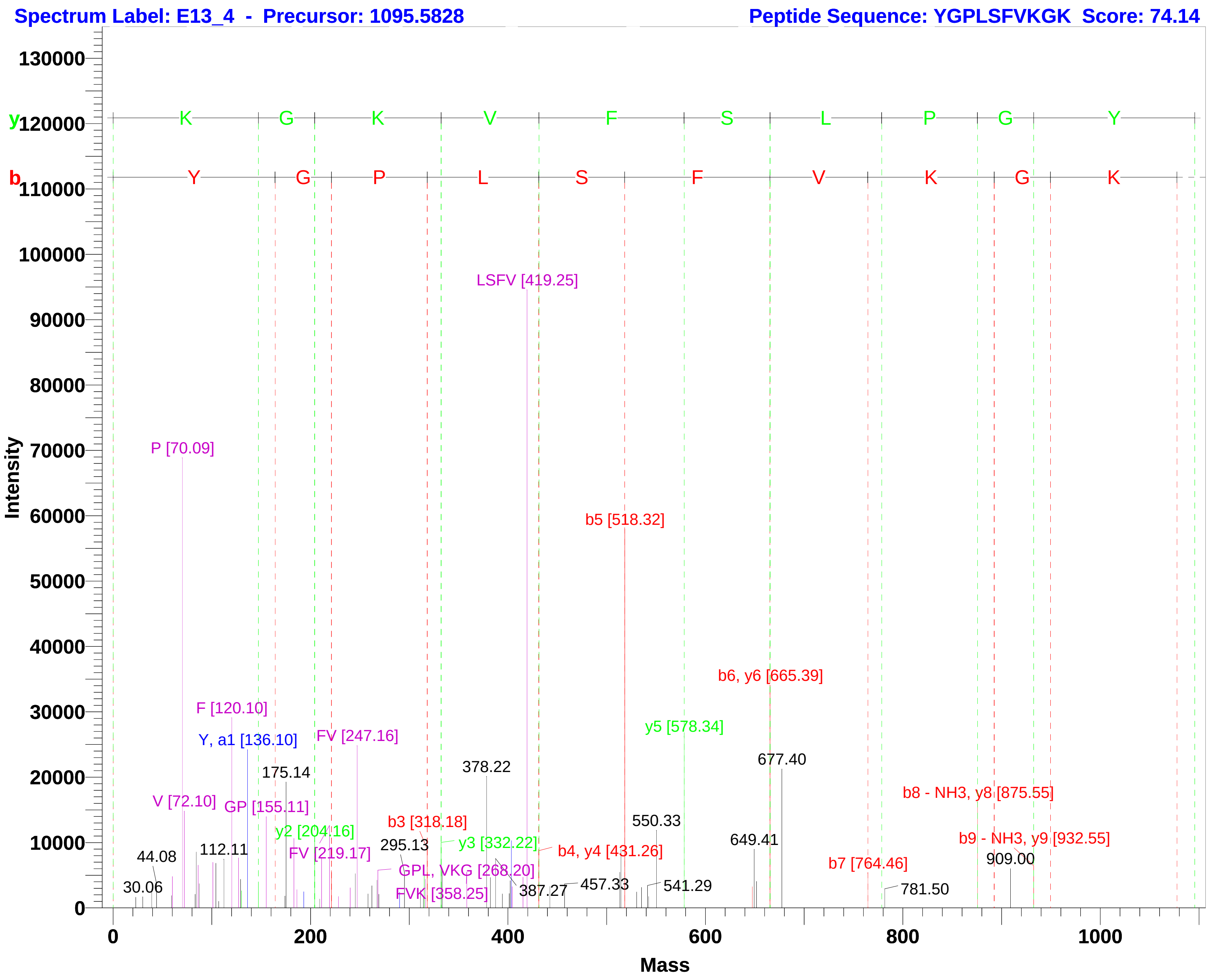

Spot S15 Superoxide dismutase

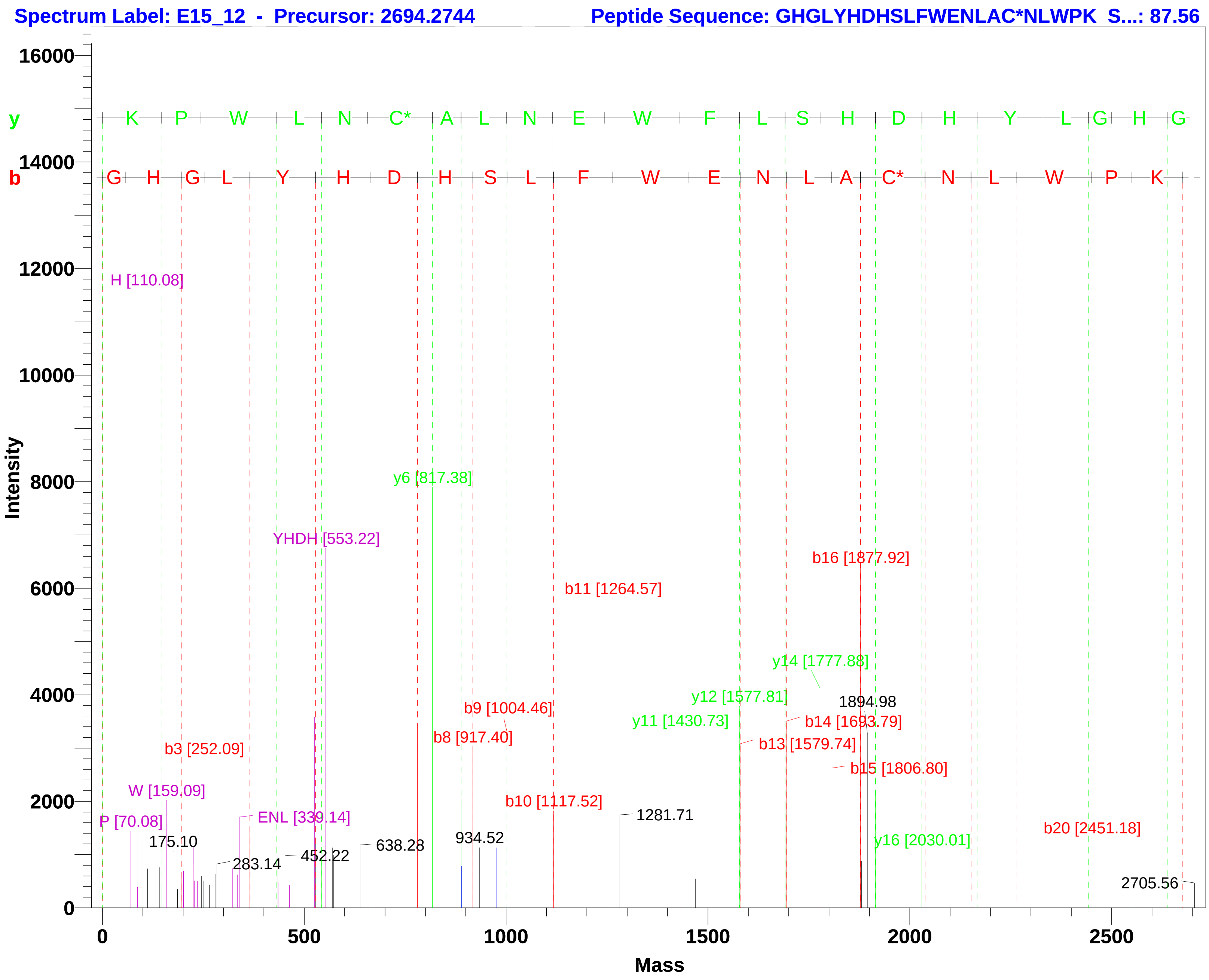

Spot S15 Superoxide dismutase

Spectrum Label: E15_3 - Precursor: 2136.1079 **Peptide Sequence: SYIPPLPYAYDAIEPAISR Score: 82.72**

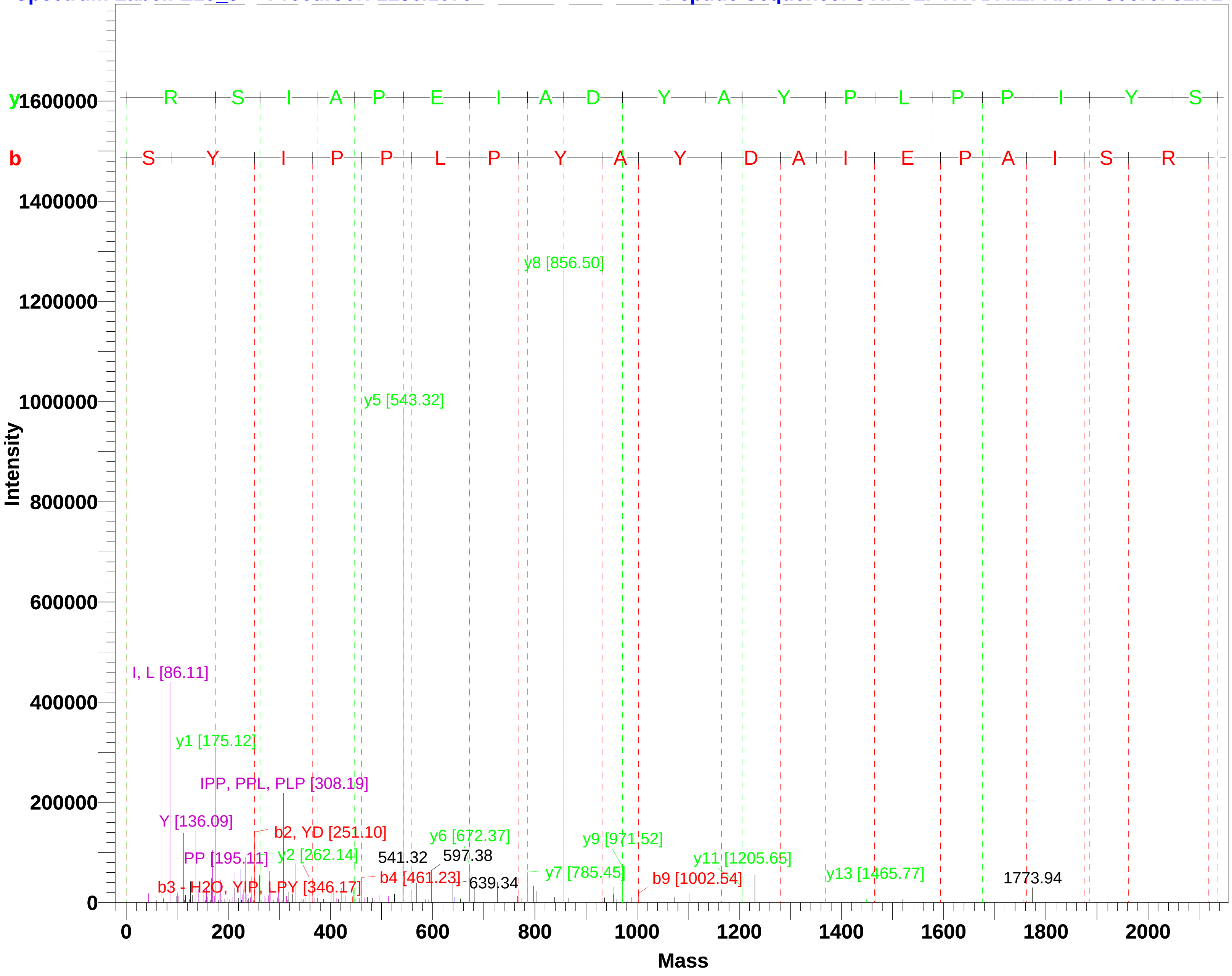

Spot S16 Glutathione reductase

Spectrum Label: E16_2 - Precursor: 1954.9436 **Peptide Sequence: SFDPIIKDTITKEYER Score: 91.51**

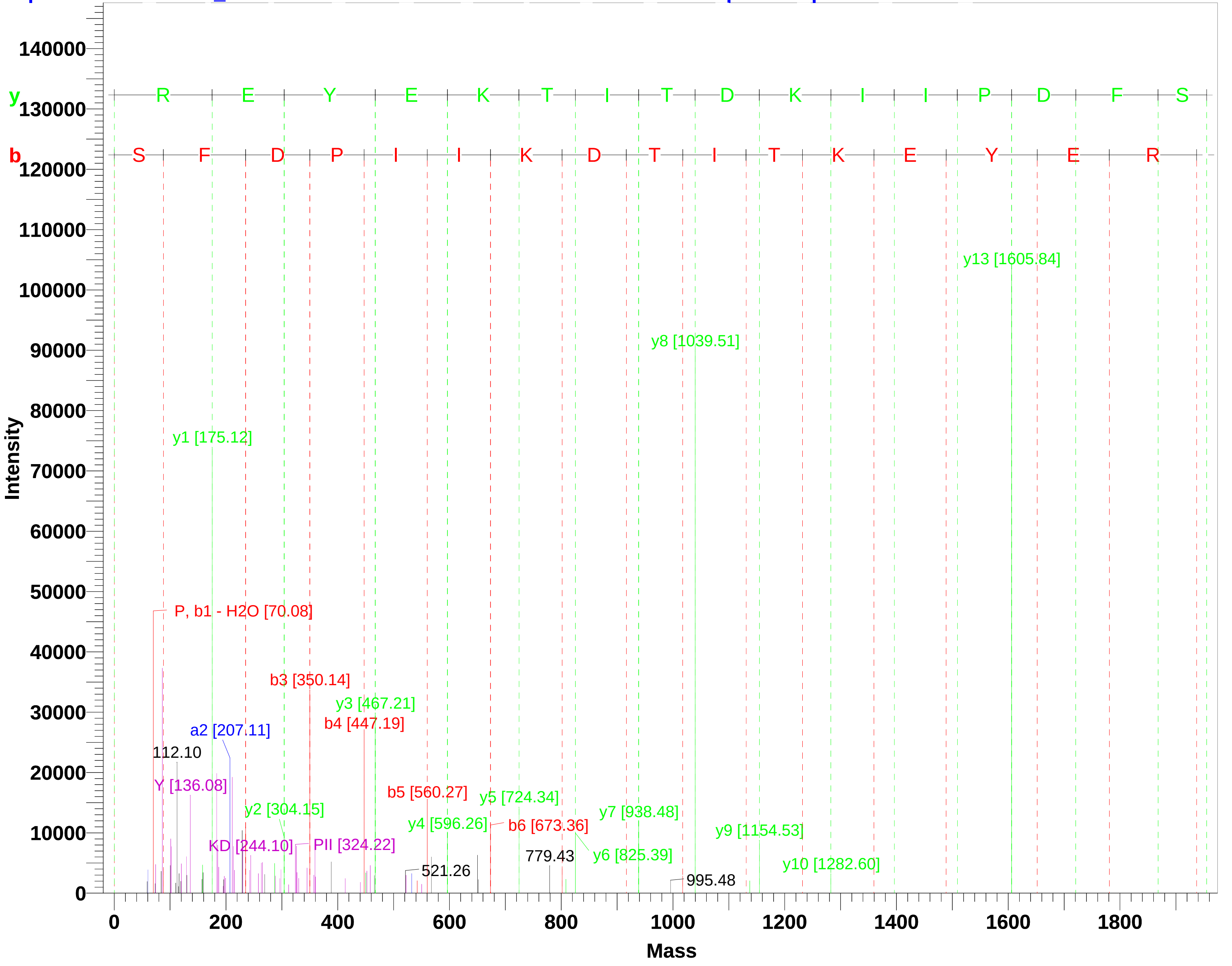

Spot S16 Glutathione reductase

Spectrum Label: E16_5 - Precursor: 1767.8699 **Peptide Sequence: VAPHYLVLNGSGGLASGR Score: 74.92**

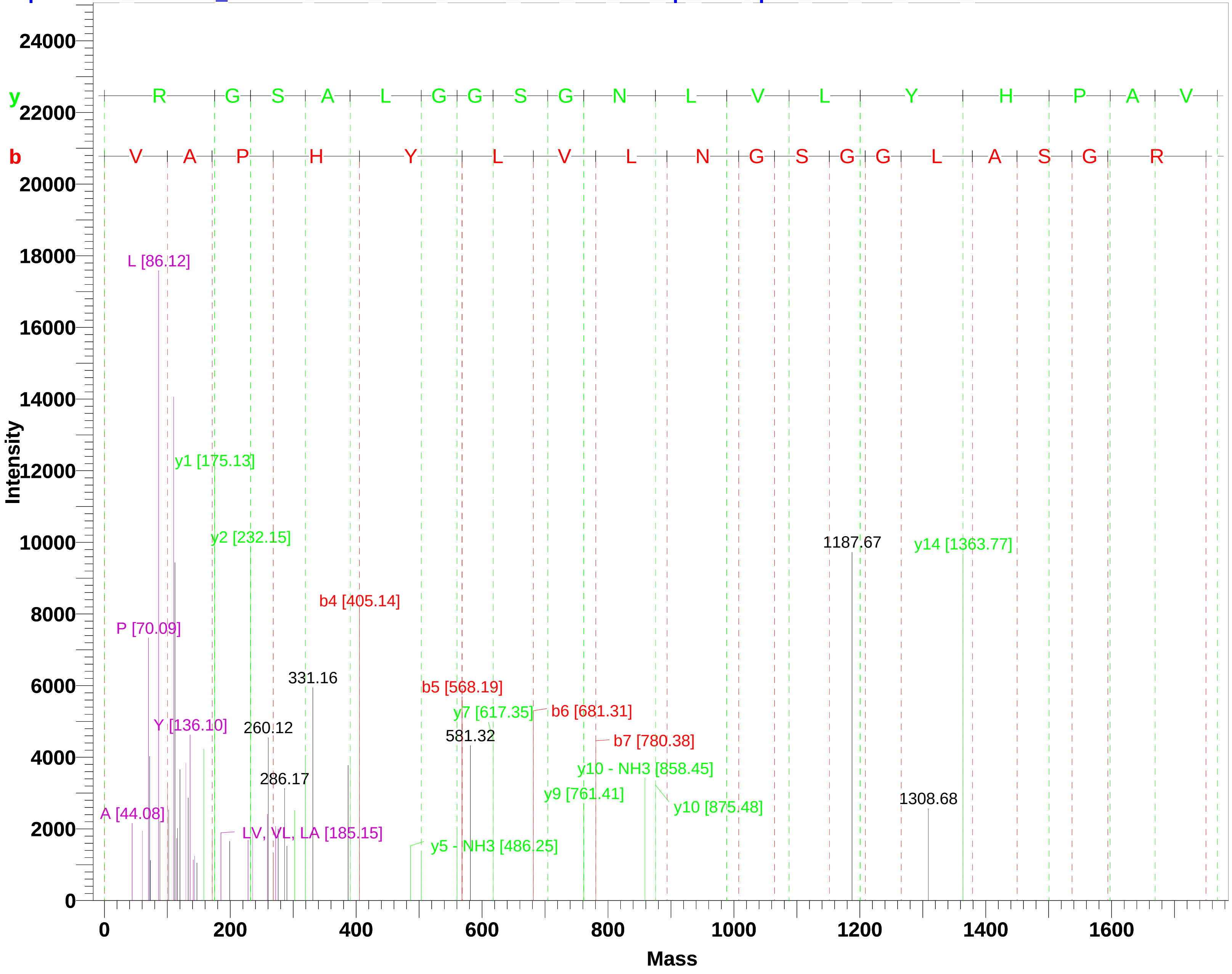

Supplement: Additional file 2 — Extracellular proteins identified by means of PRO-BLAST search. Fragmentation spectra obtained from secreted proteins after de novo sequencing by means of ProBLAST software and suitably identified by BLAST similarity searching are showed. [file 1477-5956-8-46-S2.PDF]
