# Supplementary material for: Cytoplasmic- and extracellular-proteome analysis of Diplodia seriata: a phytopathogenic fungus involved in grapevine decline
Source: Proteome Sci. 2010 Sep 9;8:46. doi: 10.1186/1477-5956-8-46 (PMC2944164; doi:10.1186/1477-5956-8-46)
Supplement: Additional file 3 — MASCOT Search Results. Data (observed, expected and calculated mases, ppm and misscleavages) corresponding to those proteins identified by combined PMF and MS/MS search using MASCOT are indicated. [file 1477-5956-8-46-S3.PDF]

Spot S1

Mascot Search Results

Protein View

Match to: **gi|256724272** Score: **83** Expect: **0.0032**
hypothetical protein NECHADRAFT_53490 [Nectria haematococca mpVI 77-13-4]

Nominal mass (M_r): **13527**; Calculated pI value: **9.60**
NCBI BLAST search of gi|256724272 against nr
Unformatted sequence string for pasting into other applications

Taxonomy: Nectria haematococca mpVI 77-13-4

Fixed modifications: Carbamidomethyl (C)
Variable modifications: Oxidation (M)
Cleavage by Trypsin: cuts C-term side of KR unless next residue is P
Sequence Coverage: **20%**

Matched peptides shown in **Bold Red**

```
  1 MAAPAASSDS VSMMAATPQW TIQGLSRSCD KPDTTCTWNF KINTGSGAAT
 51 ACKYVVKGKS ASKANGGPAK CGTFTITSGW SGQFGAGKGF TTVSVVSSKK
101 QIIYPAYTDK QLAGGKVVKP DQSYAPAALP
```

Show predicted peptides also

Sort Peptides By ◉ Residue Number ○ Increasing Mass ○ Decreasing Mass

Start - End	Observed	Mr(expt)	Mr(calc)	ppm	Miss	Sequence
42 - 57	1639.8523	1638.8450	1638.8399	3	1	K.INTGSGAATACKYVVK.G (No match)
101 - 110	1211.6337	1210.6264	1210.6234	3	0	K.QIIYPAYTDK.Q (Ions score 72)
101 - 110	1211.6337	1210.6264	1210.6234	3	0	K.QIIYPAYTDK.Q (No match)

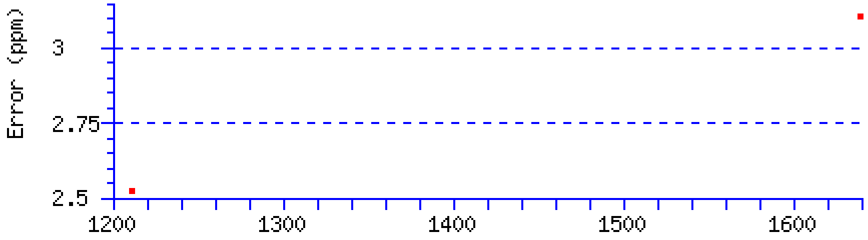

RMS error 2 ppm

```
LOCUS       EEU37643                 130 aa            linear   PLN 30-AUG-2009
DEFINITION  hypothetical protein NECHADRAFT_53490 [Nectria haematococca mpVI
            77-13-4].
ACCESSION   EEU37643
VERSION     EEU37643.1  GI:256724272
DBLINK      Project: 16586
DBSOURCE    accession GG698921.1
KEYWORDS    .
SOURCE      Nectria haematococca mpVI 77-13-4
  ORGANISM  Nectria haematococca mpVI 77-13-4
            Eukaryota; Fungi; Dikarya; Ascomycota; Saccharomyceta;
            Pezizomycotina; Leotiomyceta; Sordariomyceta; Sordariomycetes;
            Hypocreomycetidae; Hypocreales; Nectriaceae; Nectria; Nectria
            haematococca complex.
REFERENCE   1  (residues 1 to 130)
  AUTHORS   Coleman,J.J., Rounsley,S.D., Rodriguez-Carres,M., Kuo,A.,
            Wasmann,C.C., Grimwood,J., Schmutz,J., Taga,M., White,G.J.,
            Zhou,S., Schwartz,D.C., Freitag,M., Ma,L.J., Danchin,E.G.,
            Henrissat,B., Coutinho,P.M., Nelson,D.R., Straney,D., Napoli,C.A.,
            Barker,B.M., Gribskov,M., Rep,M., Kroken,S., Molnar,I., Rensing,C.,
            Kennell,J.C., Zamora,J., Farman,M.L., Selker,E.U., Salamov,A.,
            Shapiro,H., Pangilinan,J., Lindquist,E., Lamers,C., Grigoriev,I.V.,
            Geiser,D.M., Covert,S.F., Temporini,E. and Vanetten,H.D.
  TITLE     The genome of Nectria haematococca: contribution of supernumerary
            chromosomes to gene expansion
  JOURNAL   PLoS Genet. 5 (8), E1000618 (2009)
   PUBMED   19714214
REFERENCE   2  (residues 1 to 130)
  AUTHORS   Kuo,A., Salamov,A., Grimwood,J., Schmutz,J., Pangilinan,J.,
            Shapiro,H., Lindquist,E., Lucas,S., Pitluck,S., Henrissat,B.,
            Geiser,D.M., Coleman,J.J., Kroken,S., Napoli,C.A., Rep,M.,
            Covert,S.F., Temporini,E., VanEtten,H.D. and Grigoriev,I.
  CONSRTM   US DOE Joint Genome Institute (JGI-PGF)
  TITLE     Direct Submission
  JOURNAL   Submitted (14-JUL-2009) US DOE Joint Genome Institute, 2800
            Mitchell Drive, Walnut Creek, CA 94598-1698, USA
COMMENT     Method: conceptual translation.
FEATURES             Location/Qualifiers
     source          1..130
                     /organism="Nectria haematococca mpVI 77-13-4"
                     /isolate="77-13-4"
                     /culture_collection="FGSC:9596"
                     /db_xref="taxon:660122"
                     /chromosome="12"
                     /country="USA"
                     /note="mpVI; mating population VI"
     Protein         1..130
                     /product="hypothetical protein"
```

```
CDS             1..130
                /locus_tag="NECHADRAFT_53490"
                /coded_by="complement(GG698921.1:41444..41836)"
                /note="expressed hypothetical protein"
                /db_xref="JGIDB:Necha2_53490"
```

Mascot: http://www.matrixscience.com/

Spot S3

Mascot Search Results

Protein View

Match to: **gi|256724272** Score: **82** Expect: **0.0038**
hypothetical protein NECHADRAFT_53490 [Nectria haematococca mpVI 77-13-4]

Nominal mass (M_r): **13527**; Calculated pI value: **9.60**
NCBI BLAST search of gi|256724272 against nr
Unformatted sequence string for pasting into other applications

Taxonomy: Nectria haematococca mpVI 77-13-4

Fixed modifications: Carbamidomethyl (C)
Variable modifications: Oxidation (M)
Cleavage by Trypsin: cuts C-term side of KR unless next residue is P
Sequence Coverage: **20%**

Matched peptides shown in **Bold Red**

```
  1 MAAPAASSDS VSMMAATPQW TIQGLSRSCD KPDTTCTWNF KINTGSGAAT
 51 ACKYVVKGKS ASKANGGPAK CGTFTITSGW SGQFGAGKGF TTVSVVSSKK
101 QIIYPAYTDK QLAGGKVVKP DQSYAPAALP
```

Show predicted peptides also

Sort Peptides By ◉ Residue Number ○ Increasing Mass ○ Decreasing Mass

Start - End	Observed	Mr(expt)	Mr(calc)	ppm	Miss	Sequence
42 - 57	1639.8645	1638.8572	1638.8399	11	1	K.INTGSGAATACKYVVK.G (No match)
101 - 110	1211.6310	1210.6237	1210.6234	0	0	K.QIIYPAYTDK.Q (Ions score 71)
101 - 110	1211.6310	1210.6237	1210.6234	0	0	K.QIIYPAYTDK.Q (No match)

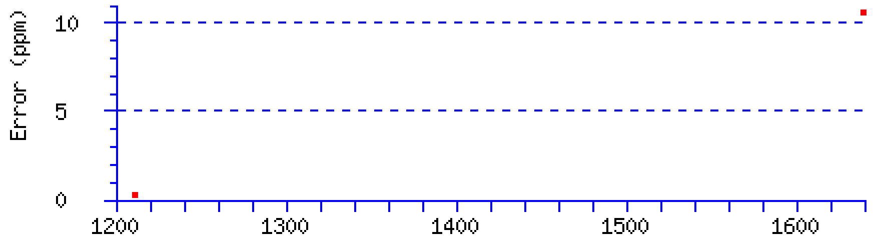

RMS error 6 ppm

```
LOCUS       EEU37643                 130 aa            linear   PLN 30-AUG-2009
DEFINITION  hypothetical protein NECHADRAFT_53490 [Nectria haematococca mpVI
            77-13-4].
ACCESSION   EEU37643
VERSION     EEU37643.1  GI:256724272
DBLINK      Project: 16586
DBSOURCE    accession GG698921.1
KEYWORDS    .
SOURCE      Nectria haematococca mpVI 77-13-4
  ORGANISM  Nectria haematococca mpVI 77-13-4
            Eukaryota; Fungi; Dikarya; Ascomycota; Saccharomyceta;
            Pezizomycotina; Leotiomyceta; Sordariomyceta; Sordariomycetes;
            Hypocreomycetidae; Hypocreales; Nectriaceae; Nectria; Nectria
            haematococca complex.
REFERENCE   1  (residues 1 to 130)
  AUTHORS   Coleman,J.J., Rounsley,S.D., Rodriguez-Carres,M., Kuo,A.,
            Wasmann,C.C., Grimwood,J., Schmutz,J., Taga,M., White,G.J.,
            Zhou,S., Schwartz,D.C., Freitag,M., Ma,L.J., Danchin,E.G.,
            Henrissat,B., Coutinho,P.M., Nelson,D.R., Straney,D., Napoli,C.A.,
            Barker,B.M., Gribskov,M., Rep,M., Kroken,S., Molnar,I., Rensing,C.,
            Kennell,J.C., Zamora,J., Farman,M.L., Selker,E.U., Salamov,A.,
            Shapiro,H., Pangilinan,J., Lindquist,E., Lamers,C., Grigoriev,I.V.,
            Geiser,D.M., Covert,S.F., Temporini,E. and Vanetten,H.D.
  TITLE     The genome of Nectria haematococca: contribution of supernumerary
            chromosomes to gene expansion
  JOURNAL   PLoS Genet. 5 (8), E1000618 (2009)
   PUBMED   19714214
REFERENCE   2  (residues 1 to 130)
  AUTHORS   Kuo,A., Salamov,A., Grimwood,J., Schmutz,J., Pangilinan,J.,
            Shapiro,H., Lindquist,E., Lucas,S., Pitluck,S., Henrissat,B.,
            Geiser,D.M., Coleman,J.J., Kroken,S., Napoli,C.A., Rep,M.,
            Covert,S.F., Temporini,E., VanEtten,H.D. and Grigoriev,I.
  CONSRTM   US DOE Joint Genome Institute (JGI-PGF)
  TITLE     Direct Submission
  JOURNAL   Submitted (14-JUL-2009) US DOE Joint Genome Institute, 2800
            Mitchell Drive, Walnut Creek, CA 94598-1698, USA
COMMENT     Method: conceptual translation.
FEATURES             Location/Qualifiers
     source          1..130
                     /organism="Nectria haematococca mpVI 77-13-4"
                     /isolate="77-13-4"
                     /culture_collection="FGSC:9596"
                     /db_xref="taxon:660122"
                     /chromosome="12"
                     /country="USA"
                     /note="mpVI; mating population VI"
     Protein         1..130
                     /product="hypothetical protein"
```

```
     CDS             1..130
                     /locus_tag="NECHADRAFT_53490"
                     /coded_by="complement(GG698921.1:41444..41836)"
                     /note="expressed hypothetical protein"
                     /db_xref="JGIDB:Necha2_53490"
```

Mascot: http://www.matrixscience.com/

Spot S7

Mascot Search Results

Protein View

Match to: **gi|189192036** Score: **94** Expect: **0.00026**
hypothetical protein PTRG_02024 [Pyrenophora tritici-repentis Pt-1C-BFP]

Nominal mass (M_r): **22518**; Calculated pI value: **7.63**
NCBI BLAST search of gi|189192036 against nr
Unformatted sequence string for pasting into other applications

Taxonomy: Pyrenophora tritici-repentis Pt-1C-BFP
Links to retrieve other entries containing this sequence from NCBI Entrez:
gi|187973963 from Pyrenophora tritici-repentis Pt-1C-BFP

Fixed modifications: Carbamidomethyl (C)
Variable modifications: Oxidation (M)
Cleavage by Trypsin: cuts C-term side of KR unless next residue is P
Sequence Coverage: **18%**

Matched peptides shown in **Bold Red**

```
  1 MKTSIFIAVA SAALVSAAPA PVSLEARAPP NIPTKATATT LLAGLTVAAQ
 51 GPQDGYSRDL FPHWITQSGT CNTRETVLKR DGTNVVTNDA CASTSGNWLS
101 PYDGGSWTAA SDVDIDHMVP LSNAWKSGAA AWTTARRQAF ANDLTNPQLL
151 AVTDNVNQAK GDKGPEDWKP PLTSYYCTYS KAWIKVKSVY SLTITSAEKS
201 ALTSMLATCS S
```

Show predicted peptides also

Sort Peptides By ◉ Residue Number ○ Increasing Mass ○ Decreasing Mass

Start - End	Observed	Mr(expt)	Mr(calc)	ppm	Miss	Sequence	
137 - 160	2641.2021	2640.1948	2640.3620	-63	1	R.RQAFANDLTNPQLLAVTDNVNQAK.G	(No match)
186 - 199	1525.8484	1524.8411	1524.8399	1	1	K.VKSVYSLTITSAEK.S	(No match)
188 - 199	1298.6881	1297.6808	1297.6765	3	0	K.SVYSLTITSAEK.S	(Ions score 78)
188 - 199	1298.6881	1297.6808	1297.6765	3	0	K.SVYSLTITSAEK.S	(No match)

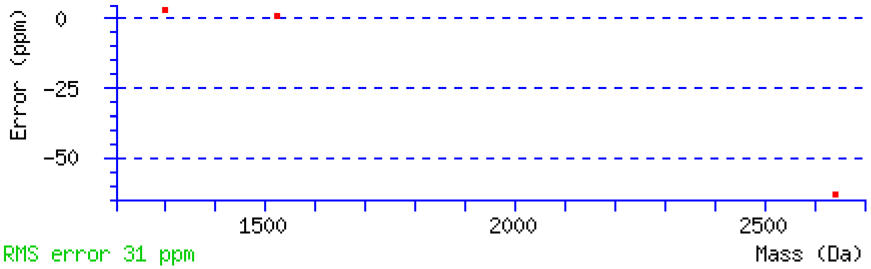


```
LOCUS       XP_001932357             211 aa            linear   PLN 30-MAY-2008
DEFINITION  hypothetical protein PTRG_02024 [Pyrenophora tritici-repentis
            Pt-1C-BFP].
ACCESSION   XP_001932357
VERSION     XP_001932357.1  GI:189192036
DBSOURCE    REFSEQ: accession XM_001932322.1
KEYWORDS    .
SOURCE      Pyrenophora tritici-repentis Pt-1C-BFP
  ORGANISM  Pyrenophora tritici-repentis Pt-1C-BFP
            Eukaryota; Fungi; Dikarya; Ascomycota; Saccharomyceta;
            Pezizomycotina; Leotiomyceta; Dothideomyceta; Dothideomycetes;
            Pleosporomycetidae; Pleosporales; Pleosporineae; Pleosporaceae;
            Pyrenophora.
REFERENCE   1  (residues 1 to 211)
  AUTHORS   Birren,B., Lander,E., Galagan,J., Nusbaum,C., Devon,K., Ma,L.-J.,
            Jaffe,D., Butler,J., Alvarez,P., Gnerre,S., Grabherr,M., Kleber,M.,
            Mauceli,E., Brockman,W., MacCallum,I.A., Young,S., LaButti,K.,
            DeCaprio,D., Crawford,M., Koehrsen,M., Engels,R., Montgomery,P.,
            Pearson,M., Howarth,C., Larson,L., White,J., Yandava,C., Kodira,C.,
            Guigo,R., Borodovsky,M., Zeng,Q., O'Leary,S., Alvarado,L.,
            Pandelova,I. and Ciuffetti,L.
  CONSRTM   The Broad Institute Genome Sequencing Platform
  TITLE     Genome Sequence of Pyrenophora tritici-repentis
  JOURNAL   Unpublished
REFERENCE   2  (residues 1 to 211)
  AUTHORS   Birren,B., Lander,E., Galagan,J., Nusbaum,C., Devon,K., Ma,L.-J.,
            Jaffe,D., Butler,J., Alvarez,P., Gnerre,S., Grabherr,M., Kleber,M.,
            Mauceli,E., Brockman,W., MacCallum,I.A., Young,S., LaButti,K.,
            DeCaprio,D., Crawford,M., Koehrsen,M., Engels,R., Montgomery,P.,
            Pearson,M., Howarth,C., Larson,L., White,J., Yandava,C., Kodira,C.,
            Zeng,Q., O'Leary,S., Alvarado,L., Ciuffetti,L. and Pandelova,I.
  CONSRTM   The Broad Institute Genome Sequencing Platform
  TITLE     Direct Submission
  JOURNAL   Submitted (16-MAR-2007) Broad Institute of MIT and Harvard, 7
            Cambridge Center, Cambridge, MA 02142, USA
```

```
COMMENT     PROVISIONAL REFSEQ: This record has not yet been subject to final
            NCBI review. The reference sequence was derived from EDU41462.
            Method: conceptual translation.
FEATURES             Location/Qualifiers
     source          1..211
                     /organism="Pyrenophora tritici-repentis Pt-1C-BFP"
                     /strain="Pt-1C-BFP"
                     /db_xref="taxon:426418"
     Protein         1..211
                     /product="hypothetical protein"
                     /calculated_mol_wt=22173
     Region          50..200
                     /region_name="DUF1994"
                     /note="Domain of unknown function (DUF1994); pfam09352"
                     /db_xref="CDD:150128"
     CDS             1..211
                     /locus_tag="PTRG_02024"
                     /coded_by="XM_001932322.1:1..636"
                     /db_xref="GeneID:6339539"
```

Mascot: http://www.matrixscience.com/

Spot S9

Mascot Search Results

Protein View

Match to: **gi|156061869** Score: **77** Expect: **0.012**
hypothetical protein SS1G_03080 [Sclerotinia sclerotiorum 1980]

Nominal mass (M_r): **26879**; Calculated pI value: **6.64**
NCBI BLAST search of gi|156061869 against nr
Unformatted sequence string for pasting into other applications

Taxonomy: Sclerotinia sclerotiorum 1980 UF-70
Links to retrieve other entries containing this sequence from NCBI Entrez:
gi|154700481 from Sclerotinia sclerotiorum 1980 UF-70

Fixed modifications: Carbamidomethyl (C)
Variable modifications: Oxidation (M)
Cleavage by Trypsin: cuts C-term side of KR unless next residue is P
Sequence Coverage: **21%**

Matched peptides shown in **Bold Red**

```
  1 MLFTNTKFLS VLAAAAAVKG APVEGNDIQA RAVVNHDSIN PWPENVPGGA
 51 LGNTLKRFEP YLHIAHGCQP YSAVDGYGNT SGGLQDTGNI SAGCRDQAKG
101 QTYVRGAWSG GKYGIMYAWY FPKDQPAAGN VVGGHRHDWE YVVIWTNNPE
151 VANPELLGGA ASSHSSNRKS TSIPTQGTRP KVEYFVEFPT NHELQFTNTL
201 GRDLPMMWYD FLPAVSKTAL DNTKFGDANC PFNNANFARK LAEAQI
```

Show predicted peptides also

Sort Peptides By ◉ Residue Number ○ Increasing Mass ○ Decreasing Mass

Start - End	Observed	Mr(expt)	Mr(calc)	ppm	Miss	Sequence
1 - 7	854.4020	853.3947	853.4368	-49	0	-.MLFTNTK.F (No match)
1 - 7	854.4020	853.3947	853.4368	-49	0	-.MLFTNTK.F (No match)
1 - 7	870.4010	869.3937	869.4317	-44	0	-.MLFTNTK.F Oxidation (M) (No match)
1 - 19	2011.9583	2010.9510	2011.1176	-83	1	-.MLFTNTKFLSVLAAAAAVK.G Oxidation (M) (No match)
96 - 105	1165.5872	1164.5799	1164.5887	-8	1	R.DQAKGQTYVR.G (No match)
113 - 123	1438.6901	1437.6828	1437.6791	3	0	K.YGIMYAWYFPK.D (Ions score 41)
113 - 123	1438.6901	1437.6828	1437.6791	3	0	K.YGIMYAWYFPK.D (No match)
113 - 123	1454.6866	1453.6793	1453.6740	4	0	K.YGIMYAWYFPK.D Oxidation (M) (No match)
113 - 136	2713.2756	2712.2683	2712.2907	-8	1	K.YGIMYAWYFPKDQPAAGNVVGGHR.H Oxidation (M) (No match)
124 - 136	1277.7162	1276.7089	1276.6273	64	0	K.DQPAAGNVVGGHR.H (No match)

Error: try setting browser cache to automatic.

```
LOCUS       XP_001596857             246 aa            linear   PLN 26-FEB-2008
DEFINITION  hypothetical protein SS1G_03080 [Sclerotinia sclerotiorum 1980].
ACCESSION   XP_001596857
VERSION     XP_001596857.1  GI:156061869
DBSOURCE    REFSEQ: accession XM_001596807.1
KEYWORDS    .
SOURCE      Sclerotinia sclerotiorum 1980 UF-70
  ORGANISM  Sclerotinia sclerotiorum 1980 UF-70
            Eukaryota; Fungi; Dikarya; Ascomycota; Saccharomyceta;
            Pezizomycotina; Leotiomyceta; Sordariomyceta; Leotiomycetes;
            Helotiales; Sclerotiniaceae; Sclerotinia.
REFERENCE   1  (residues 1 to 246)
  AUTHORS   Birren,B., Galagan,J., Lander,E., Devon,K., Nusbaum,C., Cuomo,C.,
            Jaffe,D., Butler,J., Alvarez,P., Gnerre,S., Grabherr,M., Kleber,M.,
            Mauceli,E., Brockman,W., Rounsley,S., Young,S., LaButti,K.,
            Pushparaj,V., DeCaprio,D., Crawford,M., Koehrsen,M., Engels,R.,
            Montgomery,P., Pearson,M., Howarth,C., Yandava,C., Kodira,C.,
            Zeng,Q., Alvarado,L., O'Leary,S., Dickman,M.B., Kohn,L. and
            Rollins,J.
  CONSRTM   The Broad Institute Genome Sequencing Platform
  TITLE     Annotation of the Sclerotinia sclerotiorum 1980 genome
  JOURNAL   Unpublished
REFERENCE   2  (residues 1 to 246)
  AUTHORS   Lander,E., Birren,B. and Cuomo,C.
  CONSRTM   The Genome Sequencing Platform, The Genome Assembly Team
  TITLE     Direct Submission
  JOURNAL   Submitted (10-JUN-2005) Broad Institute of MIT and Harvard, 320
            Charles Street, Cambridge, MA 02142, USA
COMMENT     PROVISIONAL REFSEQ: This record has not yet been subject to final
            NCBI review. The reference sequence was derived from EDO00220.
            Method: conceptual translation.
FEATURES             Location/Qualifiers
     source          1..246
                     /organism="Sclerotinia sclerotiorum 1980 UF-70"
                     /strain="1980"
```

```
                     /db_xref="taxon:665079"
     Protein         1..246
                     /product="hypothetical protein"
                     /calculated_mol_wt=26594
     Region          30..244
                     /region_name="NPP1"
                     /note="Necrosis inducing protein (NPP1); pfam05630"
                     /db_xref="CDD:147668"
     CDS             1..246
                     /locus_tag="SS1G_03080"
                     /coded_by="XM_001596807.1:1..741"
                     /db_xref="GeneID:5492677"
```

Mascot: http://www.matrixscience.com/

Spot S10

{MATRIX SCIENCE} Mascot Search Results

Protein View

Match to: **gi|189196154** Score: **302** Expect: **4.1e-025**
NADP-specific glutamate dehydrogenase [Pyrenophora tritici-repentis Pt-1C-BFP]

Nominal mass (M_r): **49094**; Calculated pI value: **5.88**
NCBI BLAST search of gi|189196154 against nr
Unformatted sequence string for pasting into other applications

Taxonomy: Pyrenophora tritici-repentis Pt-1C-BFP
Links to retrieve other entries containing this sequence from NCBI Entrez:
gi|187980294 from Pyrenophora tritici-repentis Pt-1C-BFP

Fixed modifications: Carbamidomethyl (C)
Variable modifications: Oxidation (M)
Cleavage by Trypsin: cuts C-term side of KR unless next residue is P
Sequence Coverage: **31%**

Matched peptides shown in **Bold Red**

```
  1 MSEPEFEQAR KELVSTLEAS SLFSKNPEYK KALEVVSVPE RIIQFRVVWE
 51 NDKGECQVQK GYRVQFNSAL GPYKGGLRFH PTVNLSILKF LGFEQIFKNA
101 LTGLNMGGGK GGCDFDPKGK SDNEIRKFCV AFMRELNKHI GADTDVPAGD
151 IGVGGREIGY LFGAYRAERN RWEGVLTGKG GSWGGSLIRP EATGYGLVYY
201 VEHMINYASG GKESFAGKRV ALSGSGNVAQ YAALKIIELG GTVISLSDSK
251 GALIAEDDKG FTPEIINQIA ALKLERKALT ALENHNFKYI EGARPWKEVN
301 KVDVALPSAT QNEVSEDEAK ALIESGAKYI AEGSNMGCTQ EAIEVFEAHR
351 REKKGDAIWY APGKAANAGG VAVSGLEMAQ NSQRLSWTAE QVDEKLKGIM
401 KDCFENCLST AKEYFTPAEG EFPSLVGGAN VAGFRKVAAA MHDQGDWW
```

Show predicted peptides also

Sort Peptides By ◉ Residue Number ○ Increasing Mass ○ Decreasing Mass

Start - End	Observed	Mr(expt)	Mr(calc)	ppm	Miss	Sequence
11 - 25	1638.8660	1637.8587	1637.8876	-18	1	R.KELVSTLEASSLFSK.N (No match)
64 - 74	1223.6444	1222.6371	1222.6346	2	0	R.VQFNSALGPYK.G (No match)
79 - 89	1268.7410	1267.7337	1267.7289	4	0	R.FHPTVNLSILK.F (Ions score 73)
79 - 89	1268.7410	1267.7337	1267.7289	4	0	R.FHPTVNLSILK.F (No match)
90 - 98	1128.6104	1127.6031	1127.6015	1	0	K.FLGFEQIFK.N (Ions score 34)
90 - 98	1128.6104	1127.6031	1127.6015	1	0	K.FLGFEQIFK.N (No match)
139 - 156	1706.8516	1705.8443	1705.8384	3	0	K.HIGADTDVPAGDIGVGGR.E (Ions score 114)

139 - 156	1706.8516	1705.8443	1705.8384	3	0	K.HIGADTDVPAGDIGVGGR.E (No match)
170 - 179	1159.6262	1158.6189	1158.6145	4	1	R.NRWEGVLTGK.G (Ions score 33)
170 - 179	1159.6262	1158.6189	1158.6145	4	1	R.NRWEGVLTGK.G (No match)
180 - 212	3505.6985	3504.6912	3504.6772	4	0	K.GGSWGGSLIRPEATGYGLVYYVEHMINYASGGK.E Oxidation (M) (No matc
220 - 235	1548.8401	1547.8328	1547.8307	1	0	R.VALSGSGNVAQYAALK.I (No match)
365 - 384	1930.9451	1929.9378	1929.9326	3	0	K.AANAGGVAVSGLEMAQNSQR.L (No match)
365 - 384	1946.9508	1945.9435	1945.9276	8	0	K.AANAGGVAVSGLEMAQNSQR.L Oxidation (M) (No match)

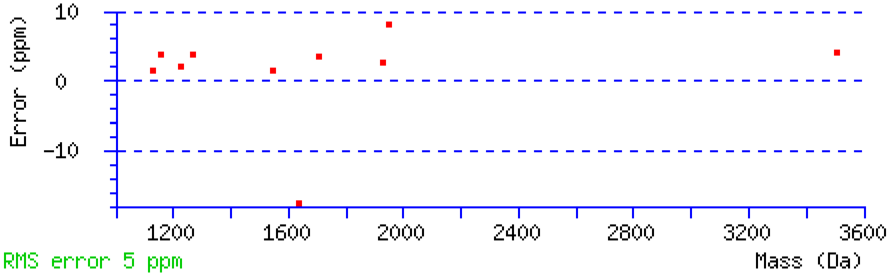

RMS error 5 ppm

```
LOCUS       XP_001934415             448 aa            linear   PLN 30-MAY-2008
DEFINITION  NADP-specific glutamate dehydrogenase [Pyrenophora tritici-repentis
            Pt-1C-BFP].
ACCESSION   XP_001934415
VERSION     XP_001934415.1  GI:189196154
DBSOURCE    REFSEQ: accession XM_001934380.1
KEYWORDS    .
SOURCE      Pyrenophora tritici-repentis Pt-1C-BFP
  ORGANISM  Pyrenophora tritici-repentis Pt-1C-BFP
            Eukaryota; Fungi; Dikarya; Ascomycota; Saccharomyceta;
            Pezizomycotina; Leotiomyceta; Dothideomyceta; Dothideomycetes;
            Pleosporomycetidae; Pleosporales; Pleosporineae; Pleosporaceae;
            Pyrenophora.
REFERENCE   1  (residues 1 to 448)
  AUTHORS   Birren,B., Lander,E., Galagan,J., Nusbaum,C., Devon,K., Ma,L.-J.,
            Jaffe,D., Butler,J., Alvarez,P., Gnerre,S., Grabherr,M., Kleber,M.,
            Mauceli,E., Brockman,W., MacCallum,I.A., Young,S., LaButti,K.,
            DeCaprio,D., Crawford,M., Koehrsen,M., Engels,R., Montgomery,P.,
            Pearson,M., Howarth,C., Larson,L., White,J., Yandava,C., Kodira,C.,
            Guigo,R., Borodovsky,M., Zeng,Q., O'Leary,S., Alvarado,L.,
            Pandelova,I. and Ciuffetti,L.
  CONSRTM   The Broad Institute Genome Sequencing Platform
  TITLE     Genome Sequence of Pyrenophora tritici-repentis
  JOURNAL   Unpublished
REFERENCE   2  (residues 1 to 448)
  AUTHORS   Birren,B., Lander,E., Galagan,J., Nusbaum,C., Devon,K., Ma,L.-J.,
            Jaffe,D., Butler,J., Alvarez,P., Gnerre,S., Grabherr,M., Kleber,M.,
            Mauceli,E., Brockman,W., MacCallum,I.A., Young,S., LaButti,K.,
```

```
            DeCaprio,D., Crawford,M., Koehrsen,M., Engels,R., Montgomery,P.,
            Pearson,M., Howarth,C., Larson,L., White,J., Yandava,C., Kodira,C.,
            Zeng,Q., O'Leary,S., Alvarado,L., Ciuffetti,L. and Pandelova,I.
  CONSRTM   The Broad Institute Genome Sequencing Platform
  TITLE     Direct Submission
  JOURNAL   Submitted (16-MAR-2007) Broad Institute of MIT and Harvard, 7
            Cambridge Center, Cambridge, MA 02142, USA
COMMENT     PROVISIONAL REFSEQ: This record has not yet been subject to final
            NCBI review. The reference sequence was derived from EDU46920.
            Method: conceptual translation.
FEATURES             Location/Qualifiers
     source          1..448
                     /organism="Pyrenophora tritici-repentis Pt-1C-BFP"
                     /strain="Pt-1C-BFP"
                     /db_xref="taxon:426418"
     Protein         1..448
                     /product="NADP-specific glutamate dehydrogenase"
                     /calculated_mol_wt=48651
     Region          3..445
                     /region_name="PRK09414"
                     /note="glutamate dehydrogenase; Provisional; PRK09414"
                     /db_xref="CDD:169848"
     Region          39..166
                     /region_name="ELFV_dehydrog_N"
                     /note="Glu/Leu/Phe/Val dehydrogenase, dimerization domain;
                     pfam02812"
                     /db_xref="CDD:145786"
     Region          177..446
                     /region_name="NAD_bind_2_Glu_DH"
                     /note="NAD(P) binding domain of glutamate dehydrogenase,
                     subgroup 2; cd05313"
                     /db_xref="CDD:133455"
     Site            order(226..228,248..249,309..310,333..335)
                     /site_type="other"
                     /note="NAD(P) binding site"
                     /db_xref="CDD:133455"
     CDS             1..448
                     /locus_tag="PTRG_04082"
                     /coded_by="XM_001934380.1:1..1347"
                     /db_xref="GeneID:6342318"
```

Mascot: http://www.matrixscience.com/

Spot S11

Mascot Search Results

Protein View

Match to: **gi|171677093** Score: **80** Expect: **0.0072**
unnamed protein product [Podospora anserina]

Nominal mass (M_r): **43049**; Calculated pI value: **8.26**
NCBI BLAST search of gi|171677093 against nr
Unformatted sequence string for pasting into other applications

Taxonomy: Podospora anserina DSM 980
Links to retrieve other entries containing this sequence from NCBI Entrez:
gi|170936614 from Podospora anserina

Fixed modifications: Carbamidomethyl (C)
Variable modifications: Oxidation (M)
Cleavage by Trypsin: cuts C-term side of KR unless next residue is P
Sequence Coverage: **19%**

Matched peptides shown in **Bold Red**

```
  1 MSLKVPQYVF GITEAFKADT FDKKINLGVG AYRDDAGKPY VLPSVRQAEE
 51 KVIASRLNKE YAGITGVPEF TKAAAVLAYG KDSPALDRVA ITQSISGTGA
101 LRIGGAFLAR FFPGAKTIYI PQPSWANHAA VFKDSGLAVE KYAYYNKETI
151 GLDFEGMIAD INKAPNGSIF LFHACAHNPT GVDPTPEQWK EIEAAVKAKG
201 HYSFFDMAYQ GFASGDIHKD AFAVRHFVAQ GHNVALSQSF AKNMGLYGER
251 IGAFSIVCEN AEEKKRVDSQ IKILVRPMYS NPPIHGARIA AEILNTPALY
301 DQWLVEVKEM ADRIITMRAL LKENLEKLGS KHDWSHITSQ IGMFAYTGLS
351 PEQMDALAKE HSVYATRDGR ISVAGITTGN VGRLAEAIFK VTG
```

Show predicted peptides also

Sort Peptides By ◉ Residue Number ○ Increasing Mass ○ Decreasing Mass

Start - End	Observed	Mr(expt)	Mr(calc)	ppm	Miss	Sequence
57 - 72	1766.7885	1765.7812	1765.9250	-81	1	R.LNKEYAGITGVPEFTK.A (No match)
198 - 219	2493.2739	2492.2666	2492.1219	58	1	K.AKGHYSFFDMAYQGFASGDIHK.D Oxidation (M) (No match)
226 - 250	2777.3384	2776.3311	2776.3504	-7	1	R.HFVAQGHNVALSQSFAKNMGLYGER.I Oxidation (M) (No match)
226 - 250	2777.3384	2776.3311	2776.3504	-7	1	R.HFVAQGHNVALSQSFAKNMGLYGER.I Oxidation (M) (No match)
243 - 250	955.4633	954.4560	954.4229	35	0	K.NMGLYGER.I Oxidation (M) (Ions score 54)
243 - 250	955.4633	954.4560	954.4229	35	0	K.NMGLYGER.I Oxidation (M) (No match)
251 - 264	1566.7706	1565.7633	1565.7395	15	0	R.IGAFSIVCENAEEK.K (No match)

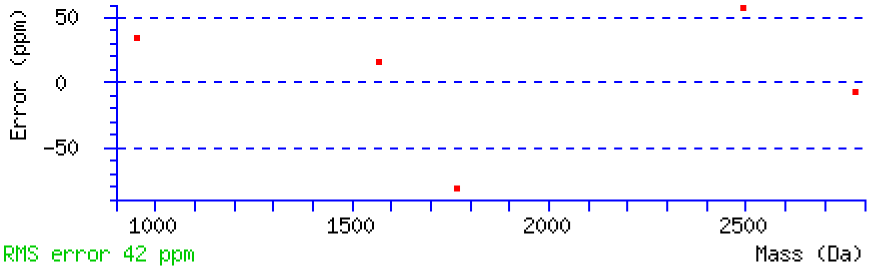

```
LOCUS       XP_001903498             393 aa            linear   PLN 05-MAY-2010
DEFINITION  hypothetical protein [Podospora anserina S mat+].
ACCESSION   XP_001903498
VERSION     XP_001903498.1  GI:171677093
DBSOURCE    REFSEQ: accession XM_001903463.1
KEYWORDS    .
SOURCE      Podospora anserina S mat+
  ORGANISM  Podospora anserina S mat+
            Eukaryota; Fungi; Dikarya; Ascomycota; Saccharomyceta;
            Pezizomycotina; Leotiomyceta; Sordariomyceta; Sordariomycetes;
            Sordariomycetidae; Sordariales; Lasiosphaeriaceae; Podospora.
REFERENCE   1  (residues 1 to 393)
  AUTHORS   Eric,Espagne., Olivier,Lespinet., Fabienne,Malagnac., Corinne
            Da,Silva., Olivier,Jaillon., Betina,M.Porcel., Arnaud,Couloux.,
            Beatrice,Segurens., Julie,Poulain., Veronique,Anthouard.,
            Sandrine,Grossetete., Hamid,Khalili., Evelyne,Coppin.,
            Michelle,Dequard.-Chablat., Marguerite,Picard.,
            Veronique,Contamine., Sylvie,Arnaise., Anne,Bourdais.,
            Veronique,Berteaux.-Lecellier., Daniel,Gautheret.,
            Ronald,Pde.Vries., Evy,Battaglia., Pedro,M.Coutinho.,
            Etienne,G.J.Danchin., Bernard,Henrissat., Riyad El,Khoury.,
            Annie,Sainsard.-Chanet., Antoine,Boivin.,
            Berangere,Pinan.-Lucarre., Carole,H.Sellem., Robert,Debuchy.,
            Patrick,Wincker., Jean,Weissenbach. and Philippe,Silar.
  TITLE     The Genome Sequence of the Model Ascomycete Fungus Podospora
            anserina
  JOURNAL   Unpublished
REFERENCE   2  (residues 1 to 393)
  AUTHORS   Genoscope -,C.E.A.
  TITLE     Direct Submission
  JOURNAL   Submitted (25-MAR-2008) Genoscope - Centre National de Sequencage :
            BP 191 91006 EVRY cedex - FRANCE (E-mail : seqref@genoscope.cns.fr
            - Web : www.genoscope.cns.fr)
COMMENT     PROVISIONAL REFSEQ: This record has not yet been subject to final
            NCBI review. The reference sequence was derived from CAP61273.
```

```
FEATURES             Location/Qualifiers
     source          1..393
                     /organism="Podospora anserina S mat+"
                     /db_xref="taxon:515849"
                     /chromosome="3"
     Protein         1..393
                     /product="hypothetical protein"
                     /calculated_mol_wt=42831
     Region          25..390
                     /region_name="AAT_like"
                     /note="Aspartate aminotransferase family. This family
                     belongs to pyridoxal phosphate (PLP)-dependent aspartate
                     aminotransferase superfamily (fold I). Pyridoxal phosphate
                     combines with an alpha-amino acid to form a compound
                     called a Schiff base or aldimine...; cd00609"
                     /db_xref="CDD:99734"
     Site            order(96..98,125,178,209,239,241..242,250)
                     /site_type="other"
                     /note="pyridoxal 5'-phosphate binding site"
                     /db_xref="CDD:99734"
     Site            order(99,135,202,248..250,286,289)
                     /site_type="other"
                     /note="homodimer interface"
                     /db_xref="CDD:99734"
     Site            242
                     /site_type="other"
                     /note="catalytic residue"
                     /db_xref="CDD:99734"
     CDS             1..393
                     /locus_tag="PODANSg513"
                     /coded_by="XM_001903463.1:1..1182"
                     /note="Predicted CDS Pa_3_11290"
                     /db_xref="GeneID:6187631"
```

Mascot: http://www.matrixscience.com/

Spot 1

Mascot Search Results

Protein View

Match to: **gi|238498522** Score: **251** Expect: **7.9e-019**
phosphoglycerate kinase PgkA, putative [Aspergillus flavus NRRL3357]

Nominal mass (M_r): **44399**; Calculated pI value: **7.66**
NCBI BLAST search of gi|238498522 against nr
Unformatted sequence string for pasting into other applications

Taxonomy: Aspergillus flavus NRRL3357
Links to retrieve other entries containing this sequence from NCBI Entrez:
gi|220693770 from Aspergillus flavus NRRL3357

Fixed modifications: Carbamidomethyl (C)
Variable modifications: Oxidation (M)
Cleavage by Trypsin: cuts C-term side of KR unless next residue is P
Sequence Coverage: **30%**

Matched peptides shown in **Bold Red**

```
  1 MSLSNKLAIT DVDLKDKRVL IRVDFNVPLD ADKKITNNQR IVGALPTIKY
 51 AIENGAKAVV LMSHLGRPDG KANPKYSLKP VATELEKLLS KSVIFAENCV
101 GKETEEIVNK ATGGQVILLE NLRFHAEEEG SSKDAEGKKV KADKEKVEEF
151 RKGLTALGDV YINDAFGTAH RAHSSMVGVD LPQKASGFLV KKELEYFAKA
201 LESPQRPFLA ILGGAKVSDK IQLIDNLLPK VNSLIITGAM AFTFKKTLEN
251 VKIGNSLFDE AGSKIVGDIV EKAKKNNVKI VLPVDYVTAD KFAADAKTGY
301 ATDADGIPDG YMGLDVGEKS VELYKKTIAE AKTILWNGPP GVFELEPFAN
351 ATKKTLDAAV AAAQSGSIVI IGGGDTATVA AKYGAEAKLS HVSTGGGASL
401 ELLEGKVLPG VDALSSK
```

Show predicted peptides also

Sort Peptides By ◉ Residue Number ○ Increasing Mass ○ Decreasing Mass

Start - End	Observed	Mr(expt)	Mr(calc)	ppm	Miss	Sequence
7 - 15	987.5745	986.5672	986.5648	2	0	K.LAITDVDLK.D (No match)
23 - 33	1232.6282	1231.6209	1231.6085	10	0	R.VDFNVPLDADK.K (No match)
23 - 33	1232.6282	1231.6209	1231.6085	10	0	R.VDFNVPLDADK.K (No match)
34 - 40	873.4912	872.4839	872.4828	1	1	K.KITNNQR.I (No match)
41 - 49	911.5909	910.5836	910.5851	-2	0	R.IVGALPTIK.Y (No match)

58 - 71	1479.8085	1478.8012	1478.8028	-1	0	K.AVVLMSHLGRPDGK.A	(Ions score 91)
58 - 71	1479.8085	1478.8012	1478.8028	-1	0	K.AVVLMSHLGRPDGK.A	(No match)
58 - 71	1495.8011	1494.7938	1494.7977	-3	0	K.AVVLMSHLGRPDGK.A	Oxidation (M) (Ions score 38)
58 - 71	1495.8011	1494.7938	1494.7977	-3	0	K.AVVLMSHLGRPDGK.A	Oxidation (M) (No match)
111 - 123	1383.8152	1382.8079	1382.7881	14	0	K.ATGGQVILLENLR.F	(Ions score 91)
111 - 123	1383.8152	1382.8079	1382.7881	14	0	K.ATGGQVILLENLR.F	(No match)
192 - 199	1027.5510	1026.5437	1026.5386	5	1	K.KELEYFAK.A	(No match)
193 - 199	899.4527	898.4454	898.4436	2	0	K.ELEYFAK.A	(No match)
231 - 246	1756.9224	1755.9151	1755.9593	-25	1	K.VNSLIITGAMAFTFKK.T	Oxidation (M) (No match)
265 - 274	1071.5603	1070.5530	1070.6335	-75	1	K.IVGDIVEKAK.K	(No match)
389 - 406	1754.9307	1753.9234	1753.9210	1	0	K.LSHVSTGGGASLELLEGK.V	(No match)
407 - 417	1085.5751	1084.5678	1084.6128	-41	0	K.VLPGVDALSSK.-	(No match)

Error: try setting browser cache to automatic.

```
LOCUS       XP_002380496             417 aa            linear   PLN 19-JAN-2010
DEFINITION  phosphoglycerate kinase PgkA, putative [Aspergillus flavus
            NRRL3357].
ACCESSION   XP_002380496
VERSION     XP_002380496.1  GI:238498522
DBSOURCE    REFSEQ: accession XM_002380455.1
KEYWORDS    .
SOURCE      Aspergillus flavus NRRL3357
  ORGANISM  Aspergillus flavus NRRL3357
            Eukaryota; Fungi; Dikarya; Ascomycota; Saccharomyceta;
            Pezizomycotina; Leotiomyceta; Eurotiomycetes; Eurotiomycetidae;
            Eurotiales; Trichocomaceae; mitosporic Trichocomaceae; Aspergillus.
REFERENCE   1  (residues 1 to 417)
  AUTHORS   Nierman,W.C.
  TITLE     Direct Submission
  JOURNAL   Submitted (16-AUG-2007) J. Craig Venter Institute, 9704 Medical
            Center Drive, Rockville, MD 20850, USA
REFERENCE   2  (residues 1 to 417)
  AUTHORS   Nierman,W.C.
  TITLE     Direct Submission
  JOURNAL   Submitted (16-JUN-2005) The Institute for Genomic Research, 9712
            Medical Center Drive, Rockville, MD 20850, USA
```

```
COMMENT     PROVISIONAL REFSEQ: This record has not yet been subject to final
            NCBI review. The reference sequence was derived from
            mrna.AFLA_069370A.
            Method: conceptual translation.
FEATURES             Location/Qualifiers
     source          1..417
                     /organism="Aspergillus flavus NRRL3357"
                     /strain="NRRL3357"
                     /db_xref="taxon:332952"
     Protein         1..417
                     /product="phosphoglycerate kinase PgkA, putative"
                     /EC_number="2.7.2.3"
                     /calculated_mol_wt=44238
     Region          9..416
                     /region_name="Phosphoglycerate_kinase"
                     /note="Phosphoglycerate kinase (PGK) is a monomeric enzyme
                     which catalyzes the transfer of the high-energy phosphate
                     group of 1,3-bisphosphoglycerate to ADP, forming ATP and
                     3-phosphoglycerate. This reaction represents the first of
                     the two substrate-level...; cd00318"
                     /db_xref="CDD:29400"
     Site            order(24,26,40,64,123)
                     /site_type="other"
                     /note="substrate binding site"
                     /db_xref="CDD:29400"
     Site            order(201..204,392..394)
                     /site_type="other"
                     /note="hinge regions"
                     /db_xref="CDD:29400"
     Site            order(239,313,337,339,341..344,374..376)
                     /site_type="other"
                     /note="ADP binding site"
                     /db_xref="CDD:29400"
     Site            375
                     /site_type="other"
                     /note="catalytic site"
                     /db_xref="CDD:29400"
     CDS             1..417
                     /locus_tag="AFLA_069370"
                     /coded_by="XM_002380455.1:1..1254"
                     /note="encoded by transcript AFLA_069370A"
                     /db_xref="GeneID:7910479"
```

Mascot: http://www.matrixscience.com/

Spot 2

Mascot Search Results

Protein View

Match to: **gi|67903236** Score: **219** Expect: **1.3e-015**
hypothetical protein AN8605.2 [Aspergillus nidulans FGSC A4]

Nominal mass (M_r): **17787**; Calculated pI value: **8.86**
NCBI BLAST search of gi|67903236 against nr
Unformatted sequence string for pasting into other applications

Taxonomy: Aspergillus nidulans FGSC A4
Links to retrieve other entries containing this sequence from NCBI Entrez:
gi|40741449 from Aspergillus nidulans FGSC A4
gi|259483180 from Aspergillus nidulans FGSC A4

Fixed modifications: Carbamidomethyl (C)
Variable modifications: Oxidation (M)
Cleavage by Trypsin: cuts C-term side of KR unless next residue is P
Sequence Coverage: **47%**

Matched peptides shown in **Bold Red**

```
  1 MSNVFFDITA NGEPLGRVEF KLFDDVVPKT ARNFRELATG QHGFGYKGSP
 51 FHRVIPQFML QGGDFTRQNG TGGKSIYGEK FEDENFTLKH DRPYLLSMAN
101 AGRNTNGSQF FITTVKTSWL DGAHVVFGEV VKGQEVVDAV EKLGSQSGAT
151 KKKVVISNSG TL
```

Show predicted peptides also

Sort Peptides By ◉ Residue Number ○ Increasing Mass ○ Decreasing Mass

Start - End	Observed	Mr(expt)	Mr(calc)	ppm	Miss	Sequence
1 - 21	2371.0571	2370.0498	2370.1678	-50	1	-.MSNVFFDITANGEPLGRVEFK.L (No match)
1 - 21	2387.0676	2386.0603	2386.1627	-43	1	-.MSNVFFDITANGEPLGRVEFK.L Oxidation (M) (No match)
18 - 29	1435.7466	1434.7393	1434.7759	-25	1	R.VEFKLFDDVVPK.T (Ions score 53)
18 - 29	1435.7466	1434.7393	1434.7759	-25	1	R.VEFKLFDDVVPK.T (No match)
54 - 67	1608.8413	1607.8340	1607.8130	13	0	R.VIPQFMLQGGDFTR.Q (Ions score 128)
54 - 67	1608.8413	1607.8340	1607.8130	13	0	R.VIPQFMLQGGDFTR.Q (No match)
54 - 67	1624.8225	1623.8152	1623.8079	4	0	R.VIPQFMLQGGDFTR.Q Oxidation (M) (Ions score 55)
54 - 67	1624.8225	1623.8152	1623.8079	4	0	R.VIPQFMLQGGDFTR.Q Oxidation (M) (No match)
75 - 89	1819.8466	1818.8393	1818.8676	-16	1	K.SIYGEKFEDENFTLK.H (No match)

133 - 151	1902.9520	1901.9447	1901.9694	-13	1	K.GQEVVDAVEKLGSQSGATK.K	(No match)

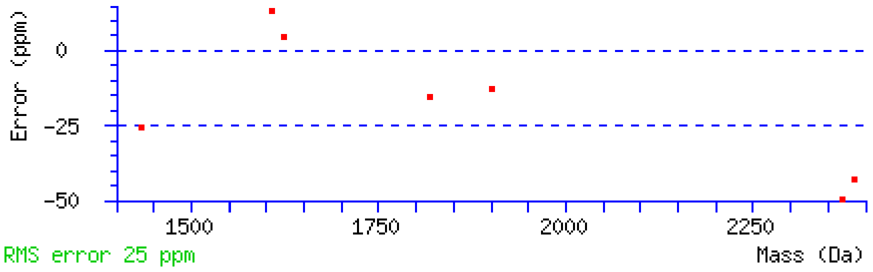

LOCUS   XP_681874   162 aa   linear   PLN 09-APR-2008
DEFINITION hypothetical protein AN8605.2 [Aspergillus nidulans FGSC A4].
ACCESSION   XP_681874
VERSION   XP_681874.1 GI:67903236
DBSOURCE   REFSEQ: accession XM_676782.1
KEYWORDS   .
SOURCE   Aspergillus nidulans FGSC A4
  ORGANISM Aspergillus nidulans FGSC A4
            Eukaryota; Fungi; Dikarya; Ascomycota; Saccharomyceta;
            Pezizomycotina; Leotiomyceta; Eurotiomycetes; Eurotiomycetidae;
            Eurotiales; Trichocomaceae; Emericella.
REFERENCE   1 (residues 1 to 162)
  AUTHORS   Galagan,J.E., Calvo,S.E., Cuomo,C., Ma,L.J., Wortman,J.R.,
            Batzoglou,S., Lee,S.I., Basturkmen,M., Spevak,C.C., Clutterbuck,J.,
            Kapitonov,V., Jurka,J., Scazzocchio,C., Farman,M., Butler,J.,
            Purcell,S., Harris,S., Braus,G.H., Draht,O., Busch,S., D'Enfert,C.,
            Bouchier,C., Goldman,G.H., Bell-Pedersen,D., Griffiths-Jones,S.,
            Doonan,J.H., Yu,J., Vienken,K., Pain,A., Freitag,M., Selker,E.U.,
            Archer,D.B., Penalva,M.A., Oakley,B.R., Momany,M., Tanaka,T.,
            Kumagai,T., Asai,K., Machida,M., Nierman,W.C., Denning,D.W.,
            Caddick,M., Hynes,M., Paoletti,M., Fischer,R., Miller,B., Dyer,P.,
            Sachs,M.S., Osmani,S.A. and Birren,B.W.
  TITLE   Sequencing of Aspergillus nidulans and comparative analysis with A.
            fumigatus and A. oryzae
  JOURNAL   Nature 438 (7071), 1105-1115 (2005)
   PUBMED   16372000
REFERENCE   2 (residues 1 to 162)
  AUTHORS   Birren,B., Nusbaum,C., Abebe,A., Abouelleil,A., Adekoya,E.,
            Ait-zahra,M., Allen,N., Allen,T., An,P., Anderson,M., Anderson,S.,
            Arachchi,H., Armbruster,J., Bachantsang,P., Baldwin,J., Barry,A.,
            Bayul,T., Blitshsteyn,B., Bloom,T., Blye,J., Boguslavskiy,L.,
            Borowsky,M., Boukhgalter,B., Brunache,A., Butler,J., Calixte,N.,
            Calvo,S., Camarata,J., Campo,K., Chang,J., Cheshatsang,Y.,

```
            Citroen,M., Collymore,A., Considine,T., Cook,A., Cooke,P.,
            Corum,B., Cuomo,C., David,R., Dawoe,T., Degray,S., Dodge,S.,
            Dooley,K., Dorje,P., Dorjee,K., Dorris,L., Duffey,N., Dupes,A.,
            Elkins,T., Engels,R., Erickson,J., Farina,A., Faro,S., Ferreira,P.,
            Fischer,H., Fitzgerald,M., Foley,K., Gage,D., Galagan,J.,
            Gearin,G., Gnerre,S., Gnirke,A., Goyette,A., Graham,J.,
            Grandbois,E., Gyaltsen,K., Hafez,N., Hagopian,D., Hagos,B.,
            Hall,J., Hatcher,B., Heller,A., Higgins,H., Honan,T., Horn,A.,
            Houde,N., Hughes,L., Hulme,W., Husby,E., Iliev,I., Jaffe,D.,
            Jones,C., Kamal,M., Kamat,A., Kamvysselis,M., Karlsson,E.,
            Kells,C., Kieu,A., Kisner,P., Kodira,C., Kulbokas,E., Labutti,K.,
            Lama,D., Landers,T., Leger,J., Levine,S., Lewis,D., Lewis,T.,
            Lindblad-toh,K., Liu,X., Lokyitsang,T., Lokyitsang,Y., Lucien,O.,
            Lui,A., Ma,L.J., Mabbitt,R., Macdonald,J., Maclean,C., Major,J.,
            Manning,J., Marabella,R., Maru,K., Matthews,C., Mauceli,E.,
            Mccarthy,M., Mcdonough,S., Mcghee,T., Meldrim,J., Meneus,L.,
            Mesirov,J., Mihalev,A., Mihova,T., Mikkelsen,T., Mlenga,V.,
            Moru,K., Mozes,J., Mulrain,L., Munson,G., Naylor,J., Newes,C.,
            Nguyen,C., Nguyen,N., Nguyen,T., Nicol,R., Nielsen,C., Nizzari,M.,
            Norbu,C., Norbu,N., O'donnell,P., Okoawo,O., O'leary,S.,
            Omotosho,B., O'neill,K., Osman,S., Parker,S., Perrin,D.,
            Phunkhang,P., Piqani,B., Purcell,S., Rachupka,T., Ramasamy,U.,
            Rameau,R., Ray,V., Raymond,C., Retta,R., Richardson,S., Rise,C.,
            Rodriguez,J., Rogers,J., Rogov,P., Rutman,M., Schupbach,R.,
            Seaman,C., Settipalli,S., Sharpe,T., Sheridan,J., Sherpa,N.,
            Shi,J., Smirnov,S., Smith,C., Sougnez,C., Spencer,B., Stalker,J.,
            Stange-thomann,N., Stavropoulos,S., Stetson,K., Stone,C., Stone,S.,
            Stubbs,M., Talamas,J., Tchuinga,P., Tenzing,P., Tesfaye,S.,
            Theodore,J., Thoulutsang,Y., Topham,K., Towey,S., Tsamla,T.,
            Tsomo,N., Vallee,D., Vassiliev,H., Venkataraman,V., Vinson,J.,
            Vo,A., Wade,C., Wang,S., Wangchuk,T., Wangdi,T., Whittaker,C.,
            Wilkinson,J., Wu,Y., Wyman,D., Yadav,S., Yang,S., Yang,X.,
            Yeager,S., Yee,E., Young,G., Zainoun,J., Zembeck,L., Zimmer,A.,
            Zody,M. and Lander,E.
  TITLE     Direct Submission
  JOURNAL   Submitted (26-APR-2004) Whitehead Institute/MIT Center for Genome
            Research, 320 Charles Street, Cambridge, MA 02142, USA
COMMENT     PROVISIONAL REFSEQ: This record has not yet been subject to final
            NCBI review. The reference sequence was derived from EAA60639.
            Method: conceptual translation.
FEATURES             Location/Qualifiers
     source          1..162
                     /organism="Aspergillus nidulans FGSC A4"
                     /strain="FGSC A4"
                     /db_xref="taxon:227321"
                     /chromosome="III"
     Protein         1..162
```

```
                     /product="hypothetical protein"
                     /calculated_mol_wt=17667
     Region          2..160
                     /region_name="cyclophilin_ABH_like"
                     /note="cyclophilin_ABH_like: Cyclophilin  A, B and H-like
                     cyclophilin-type peptidylprolyl cis- trans isomerase
                     (PPIase) domain. This family represents the archetypal
                     cystolic cyclophilin similar to human cyclophilins A, B
                     and H. PPIase is an enzyme which...; cd01926"
                     /db_xref="CDD:29397"
     Site            order(52..53,58,109,111,119)
                     /site_type="active"
                     /db_xref="CDD:29397"
     CDS             1..162
                     /locus_tag="AN8605.2"
                     /coded_by="XM_676782.1:1..489"
                     /db_xref="GeneID:2868486"
```

Mascot: http://www.matrixscience.com/

Spot 3

{MATRIX SCIENCE} Mascot Search Results

Protein View

Match to: **gi|169601412** Score: **595** Expect: **3.2e-053**
hypothetical protein SNOG_03571 [Phaeosphaeria nodorum SN15]

Nominal mass (M_r): **47405**; Calculated pI value: **5.20**
NCBI BLAST search of gi|169601412 against nr
Unformatted sequence string for pasting into other applications

Taxonomy: Phaeosphaeria nodorum SN15
Links to retrieve other entries containing this sequence from NCBI Entrez:
gi|111067656 from Phaeosphaeria nodorum SN15

Fixed modifications: Carbamidomethyl (C)
Variable modifications: Oxidation (M)
Cleavage by Trypsin: cuts C-term side of KR unless next residue is P
Sequence Coverage: **30%**

Matched peptides shown in **Bold Red**

```
  1 MAITKIHARS VYDSRGNPTV EVDIVTETGL HRAIVPSGAS TGSHEACELR
 51 DGDKTKWGGK GVTKAVANVN DTIAPALIKE AIDVKDQSAV DAFLNKLDGT
101 KNKEKLGANA ILGVSMAIAK AAAAEKGVPL YAHISDLAGT KKPYVLPVPF
151 QNVLNGGSHA GGRLAFQEFM IVPSEAPTFS EAMRQGAEVY QKLKSLAKKR
201 YGQSAGNVGD EGGVAPDIQT PEEALDLITD AIEEAGYTGK IKIAMDVASS
251 EFYKTEEKKY DLDFKNPDSD KSKWLSYEQL AELYKSLAQK YPIVSIEDPF
301 AEDDWEAWSY FFKTSDFQIV GDDLTVTNPE FIKKAIELKS CNALLLKVNQ
351 IGTITEAIQA AKDAFGAGWG VMVSHRSGET EDVTIADIVV GLRAGQIKTG
401 APARSERLAK LNQILRIEEE LGDNAIYAGQ NFRTAINL
```

Show predicted peptides also

Sort Peptides By ◉ Residue Number ○ Increasing Mass ○ Decreasing Mass

Start - End	Observed	Mr(expt)	Mr(calc)	ppm	Miss	Sequence
16 - 32	1836.9496	1835.9423	1835.9378	2	0	R.GNPTVEVDIVTETGLHR.A (Ions score 124)
16 - 32	1836.9496	1835.9423	1835.9378	2	0	R.GNPTVEVDIVTETGLHR.A (No match)
33 - 50	1841.8832	1840.8759	1840.8737	1	0	R.AIVPSGASTGSHEACELR.D (No match)
33 - 54	2257.0601	2256.0528	2256.0441	4	1	R.AIVPSGASTGSHEACELRDGDK.T (No match)
127 - 141	1541.8315	1540.8242	1540.8249	-0	0	K.GVPLYAHISDLAGTK.K (Ions score 90)

127 - 141	1541.8315	1540.8242	1540.8249	-0	0	K.GVPLYAHISDLAGTK.K	(No match)
127 - 163	3830.0461	3829.0388	3829.0428	-1	1	K.GVPLYAHISDLAGTKKPYVLPVPFQNVLNGGSHAGGR.L	(No match)
142 - 163	2307.2405	2306.2332	2306.2284	2	0	K.KPYVLPVPFQNVLNGGSHAGGR.L	(Ions score 145)
142 - 163	2307.2405	2306.2332	2306.2284	2	0	K.KPYVLPVPFQNVLNGGSHAGGR.L	(No match)
243 - 254	1360.6470	1359.6397	1359.6380	1	0	K.IAMDVASSEFYK.T	(No match)
243 - 254	1376.6459	1375.6386	1375.6329	4	0	K.IAMDVASSEFYK.T	Oxidation (M) (No match)
259 - 265	928.4814	927.4741	927.4702	4	1	K.KYDLDFK.N	(No match)
314 - 333	2239.0540	2238.0467	2238.1056	-26	0	K.TSDFQIVGDDLTVTNPEFIK.K	(No match)
314 - 334	2367.2034	2366.1961	2366.2006	-2	1	K.TSDFQIVGDDLTVTNPEFIKK.A	(No match)
377 - 393	1773.9287	1772.9214	1772.9156	3	0	R.SGETEDVTIADIVVGLR.A	(Ions score 159)
377 - 393	1773.9287	1772.9214	1772.9156	3	0	R.SGETEDVTIADIVVGLR.A	(No match)

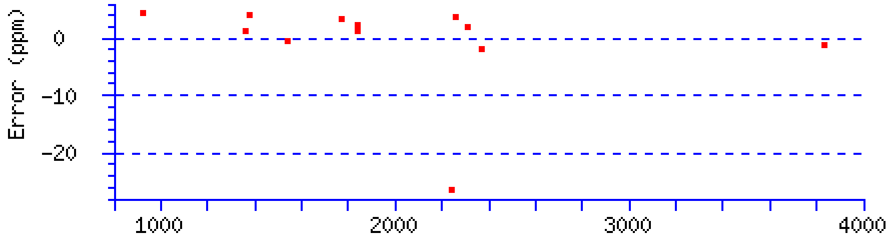

RMS error 7 ppm

```
LOCUS       XP_001794128             438 aa            linear   PLN 02-APR-2008
DEFINITION  hypothetical protein SNOG_03571 [Phaeosphaeria nodorum SN15].
ACCESSION   XP_001794128
VERSION     XP_001794128.1  GI:169601412
DBSOURCE    REFSEQ: accession XM_001794076.1
KEYWORDS    .
SOURCE      Phaeosphaeria nodorum SN15
  ORGANISM  Phaeosphaeria nodorum SN15
            Eukaryota; Fungi; Dikarya; Ascomycota; Saccharomyceta;
            Pezizomycotina; Leotiomyceta; Dothideomyceta; Dothideomycetes;
            Pleosporomycetidae; Pleosporales; Pleosporineae; Phaeosphaeriaceae;
            Phaeosphaeria.
REFERENCE   1  (residues 1 to 438)
  AUTHORS   Birren,B., Lander,E., Galagan,J., Devon,K., Nusbaum,C., Jaffe,D.,
            Butler,J., Alvarez,P., Gnerre,S., Grabherr,M., Kleber,M.,
            Mauceli,E., Brockman,W., Rounsley,S., Young,S., LaButti,K.,
            Pushparaj,V., DeCaprio,D., Crawford,M., Koehrsen,M., Engels,R.,
            Montgomery,P., Pearson,M., Howarth,C., Kodira,C., Zeng,Q.,
            Yandava,C., Alvarado,L., Oleary,S., Oliver,R.O. and Solomon,P.
  CONSRTM   The Broad Institute Genome Sequencing Platform
  TITLE     Annotation of the Phaeosphaeria nodorum SN15 genome
  JOURNAL   Unpublished
REFERENCE   2  (residues 1 to 438)
```

```
  AUTHORS   Lander,E. and Birren,B.
  CONSRTM   The Genome Sequencing Platform, The Genome Assembly Team
  TITLE     Direct Submission
  JOURNAL   Submitted (05-MAR-2008) Broad Institute of MIT and Harvard, 320
            Charles Street, Cambridge, MA 02141, USA
  REMARK    Direct Submission
REFERENCE   3  (residues 1 to 438)
  AUTHORS   Oliver,R. and Solomon,P.
  TITLE     Direct Submission
  JOURNAL   Submitted (05-MAR-2008) Murdoch University, South Street, Perth, WA
            6150, Australia
  REMARK    Direct Submission
COMMENT     PROVISIONAL REFSEQ: This record has not yet been subject to final
            NCBI review. The reference sequence was derived from EAT88776.
            Method: conceptual translation.
FEATURES             Location/Qualifiers
     source          1..438
                     /organism="Phaeosphaeria nodorum SN15"
                     /db_xref="taxon:321614"
     Protein         1..438
                     /product="hypothetical protein"
                     /calculated_mol_wt=47190
     Region          1..431
                     /region_name="PTZ00081"
                     /note="enolase (2-phospho-D-glycerate hydrolase);
                     Provisional; PTZ00081"
                     /db_xref="CDD:173375"
     Region          5..420
                     /region_name="enolase"
                     /note="Enolase: Enolases are homodimeric enzymes that
                     catalyse the reversible dehydration of
                     2-phospho-D-glycerate to phosphoenolpyruvate as part of
                     the glycolytic and gluconeogenesis pathways. The reaction
                     is facilitated by the presence of metal ions; cd03313"
                     /db_xref="CDD:48188"
     Site            order(7,9..17,21,32,160..162,183..184,187..188,191..192,
                     207..208,215,217,379..381,404..406,408..409,412,415..416,
                     419)
                     /site_type="other"
                     /note="dimer interface"
                     /db_xref="CDD:48188"
     Site            order(40,246,297,322)
                     /site_type="metal-binding"
                     /db_xref="CDD:48188"
     Site            order(159,211,347,375..377,398)
                     /site_type="other"
                     /note="substrate binding pocket"
```

```
                     /db_xref="CDD:48188"
     CDS             1..438
                     /locus_tag="SNOG_03571"
                     /coded_by="XM_001794076.1:36..1352"
                     /inference="ab initio prediction:Unveil:1.0"
                     /inference="similar to RNA sequence, EST (same
                     species):INSD:DR045144.1"
                     /inference="similar to RNA sequence, EST (same
                     species):INSD:DR045387.1"
                     /inference="similar to RNA sequence, EST (same
                     species):INSD:EH386538.1"
                     /inference="similar to RNA sequence, EST (same
                     species):INSD:EH387222.1"
                     /inference="similar to RNA sequence, EST (same
                     species):INSD:EH387450.1"
                     /inference="similar to RNA sequence, EST (same
                     species):INSD:EH387515.1"
                     /inference="similar to RNA sequence, EST (same
                     species):INSD:EH388229.1"
                     /inference="similar to RNA sequence, EST (same
                     species):INSD:EH388420.1"
                     /inference="similar to RNA sequence, EST (same
                     species):INSD:EH388466.1"
                     /inference="similar to RNA sequence, EST (same
                     species):INSD:EH388511.1"
                     /inference="similar to RNA sequence, EST (same
                     species):INSD:EH392894.1"
                     /inference="similar to RNA sequence, EST (same
                     species):INSD:EH393160.1"
                     /inference="similar to RNA sequence, EST (same
                     species):INSD:EH394998.1"
                     /inference="similar to RNA sequence, EST (same
                     species):INSD:EH395735.1"
                     /inference="similar to RNA sequence, EST (same
                     species):INSD:EH397990.1"
                     /inference="similar to RNA sequence, EST (same
                     species):INSD:EH398184.1"
                     /inference="similar to RNA sequence, EST (same
                     species):INSD:EH400659.1"
                     /inference="similar to RNA sequence, EST (same
                     species):INSD:EH400989.1"
                     /note="gene prediction version 2"
                     /db_xref="GeneID:5970991"
```

Mascot: http://www.matrixscience.com/

Spot 4

Mascot Search Results

Protein View

Match to: **gi|198425409** Score: **88** Expect: **0.015**
PREDICTED: Zn-finger (U1-like)-10 isoform 1 [Ciona intestinalis]

Nominal mass (M_r): **22597**; Calculated pI value: **9.48**
NCBI BLAST search of gi|198425409 against nr
Unformatted sequence string for pasting into other applications

Taxonomy: Ciona intestinalis

Fixed modifications: Carbamidomethyl (C)
Variable modifications: Oxidation (M)
Cleavage by Trypsin: cuts C-term side of KR unless next residue is P
Sequence Coverage: **54%**

Matched peptides shown in **Bold Red**

```
  1 MAGRGRGRGW LSKFESQSKE MKPFEQKTNP VEQKSNNSNG FMEKNSTLSK
 51 LMAKYNYKAN PNSPAGFKEL SIRQSEELFL LSEHKEQPHW WLVRNKAGEE
101 GYSPKSYLMK METKVASLPW LENKKTVEIN TKVVPTHVAP KKYVSAYKEN
151 QGKSDIQWHC DVCNKSFNGP HPYNSHMVSK AHREEVEVAQ LYGRM
```

Show predicted peptides also

Sort Peptides By ◉ Residue Number ○ Increasing Mass ○ Decreasing Mass

Start - End	Observed	Mr(expt)	Mr(calc)	ppm	Miss	Sequence
14 - 27	1758.9020	1757.8947	1757.8294	37	1	K.FESQSKEMKPFEQK.T Oxidation (M) (No match)
20 - 34	1832.9186	1831.9113	1831.9138	-1	1	K.EMKPFEQKTNPVEQK.S (No match)
45 - 54	1108.5361	1107.5288	1107.5958	-60	1	K.NSTLSKLMAK.Y Oxidation (M) (No match)
51 - 58	1030.5126	1029.5053	1029.5317	-26	1	K.LMAKYNYK.A (No match)
51 - 58	1030.5126	1029.5053	1029.5317	-26	1	K.LMAKYNYK.A (No match)
55 - 68	1570.7637	1569.7564	1569.7576	-1	1	K.YNYKANPNSPAGFK.E (No match)
59 - 68	1002.4763	1001.4690	1001.4930	-24	0	K.ANPNSPAGFK.E (No match)
106 - 114	1130.5592	1129.5519	1129.5511	1	1	K.SYLMKMETK.V (No match)
111 - 124	1645.8542	1644.8469	1644.8545	-5	1	K.METKVASLPWLENK.K (No match)
154 - 180	3260.3545	3259.3472	3259.4022	-17	1	K.SDIQWHCDVCNKSFNGPHPYNSHMVSK.A Oxidation (M) (No match)
166 - 183	2066.0923	2065.0850	2064.9700	56	1	K.SFNGPHPYNSHMVSKAHR.E (No match)
184 - 194	1292.6592	1291.6519	1291.6408	9	0	R.EEVEVAQLYGR.M (No match)
184 - 195	1439.7808	1438.7735	1438.6762	68	1	R.EEVEVAQLYGRM.- Oxidation (M) (No match)

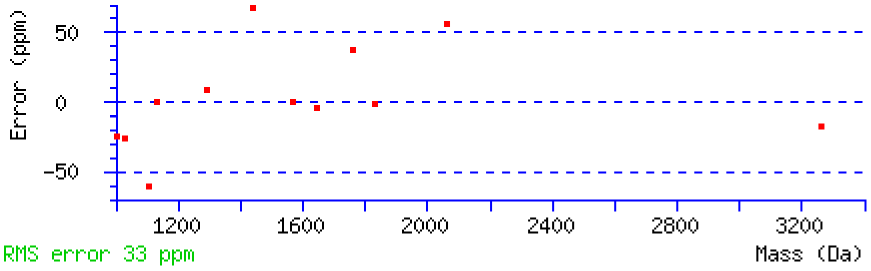


```
LOCUS       XP_002130942             195 aa            linear   INV 09-SEP-2008
DEFINITION  PREDICTED: Zn-finger (U1-like)-10 isoform 1 [Ciona intestinalis].
ACCESSION   XP_002130942
VERSION     XP_002130942.1  GI:198425409
DBSOURCE    REFSEQ: accession XM_002130906.1
KEYWORDS    .
SOURCE      Ciona intestinalis
  ORGANISM  Ciona intestinalis
            Eukaryota; Metazoa; Chordata; Tunicata; Ascidiacea; Enterogona;
            Phlebobranchia; Cionidae; Ciona.
COMMENT     MODEL REFSEQ:  This record is predicted by automated computational
            analysis. This record is derived from a genomic sequence
            (NW_001955313) annotated using gene prediction method: GNOMON,
            supported by mRNA and EST evidence.
            Also see:
                Documentation of NCBI's Annotation Process

            COMPLETENESS: full length.
FEATURES             Location/Qualifiers
     source          1..195
                     /organism="Ciona intestinalis"
                     /db_xref="taxon:7719"
     Protein         1..195
                     /product="Zn-finger (U1-like)-10 isoform 1"
                     /calculated_mol_wt=22366
     Region          53..108
                     /region_name="SH3"
                     /note="Src homology 3 domains; SH3 domains bind to
                     proline-rich ligands with moderate affinity and
                     selectivity, preferentially to PxxP motifs; they play a
                     role in the regulation of enzymes by intramolecular
                     interactions, changing the subcellular localization...;
                     cl09950"
                     /db_xref="CDD:158800"
     Region          157..187
```

```
                     /region_name="zf-C2H2_jaz"
                     /note="Zinc-finger double-stranded RNA-binding; cl09952"
                     /db_xref="CDD:158802"
     CDS             1..195
                     /gene="zf(u1like)-10"
                     /coded_by="XM_002130906.1:213..800"
                     /db_xref="GeneID:100170048"
```

Mascot: http://www.matrixscience.com/

Spot 5

Mascot Search Results

Protein View

Match to: **gi|156053161** Score: **83** Expect: **0.0036**
hypothetical protein SS1G_06748 [Sclerotinia sclerotiorum 1980]

Nominal mass (M_r): **37910**; Calculated pI value: **8.75**
NCBI BLAST search of gi|156053161 against nr
Unformatted sequence string for pasting into other applications

Taxonomy: Sclerotinia sclerotiorum 1980 UF-70
Links to retrieve other entries containing this sequence from NCBI Entrez:
gi|154704526 from Sclerotinia sclerotiorum 1980 UF-70

Fixed modifications: Carbamidomethyl (C)
Variable modifications: Oxidation (M)
Cleavage by Trypsin: cuts C-term side of KR unless next residue is P
Sequence Coverage: **5%**

Matched peptides shown in **Bold Red**

```
  1 MFTRTLPSAL RTSSRLSQLS SQIQRRSMAS ITTHKLNTGA TIPAIGFGTW
 51 QDKDSQEEAV TEALKAGYRH IDTARVYGTE SACGAAIRAS GIPRSELFIT
101 TKLWNNKHKP EDVEPALNES LKDLGLDYVD LYLMHWPSAF KPGDDLFPKV
151 DGKTQTADIS YVNTYKAMEK LIETGKTKAI GISNFSRGEL ENLLKEASIV
201 PAVHQLELHP WLQQTEFCEF NKRKGIHITQ YSPFGNQNEV YDSGKGIGKL
251 MDDPTIVEIG KKYSKSGAQV ALAWGIAHGH SVIPKSKTPS RIKSNLEGDF
301 KLEAEDLKKL DGLDKKLRFN DASASFGYNF YSDLDGKRKN
```

Show predicted peptides also

Sort Peptides By ◉ Residue Number ○ Increasing Mass ○ Decreasing Mass

Start - End	Observed	Mr(expt)	Mr(calc)	ppm	Miss	Sequence	
26 - 35	1131.5928	1130.5855	1130.5866	-1	1	R.RSMASITTHK.L	(No match)
179 - 187	964.5257	963.5184	963.5138	5	0	K.AIGISNFSR.G	(Ions score 77)
179 - 187	964.5257	963.5184	963.5138	5	0	K.AIGISNFSR.G	(No match)

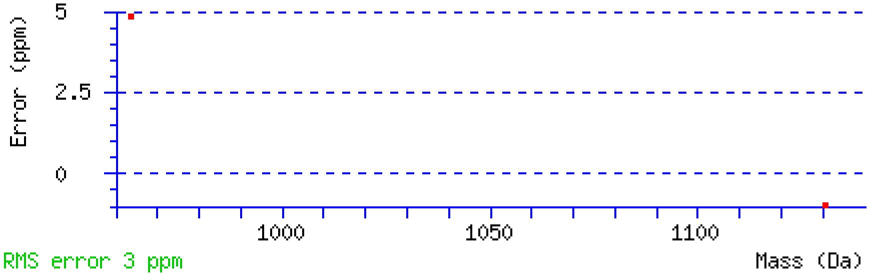


```
LOCUS       XP_001592507             340 aa            linear   PLN 26-FEB-2008
DEFINITION  hypothetical protein SS1G_06748 [Sclerotinia sclerotiorum 1980].
ACCESSION   XP_001592507
VERSION     XP_001592507.1  GI:156053161
DBSOURCE    REFSEQ: accession XM_001592457.1
KEYWORDS    .
SOURCE      Sclerotinia sclerotiorum 1980 UF-70
  ORGANISM  Sclerotinia sclerotiorum 1980 UF-70
            Eukaryota; Fungi; Dikarya; Ascomycota; Saccharomyceta;
            Pezizomycotina; Leotiomyceta; Sordariomyceta; Leotiomycetes;
            Helotiales; Sclerotiniaceae; Sclerotinia.
REFERENCE   1  (residues 1 to 340)
  AUTHORS   Birren,B., Galagan,J., Lander,E., Devon,K., Nusbaum,C., Cuomo,C.,
            Jaffe,D., Butler,J., Alvarez,P., Gnerre,S., Grabherr,M., Kleber,M.,
            Mauceli,E., Brockman,W., Rounsley,S., Young,S., LaButti,K.,
            Pushparaj,V., DeCaprio,D., Crawford,M., Koehrsen,M., Engels,R.,
            Montgomery,P., Pearson,M., Howarth,C., Yandava,C., Kodira,C.,
            Zeng,Q., Alvarado,L., O'Leary,S., Dickman,M.B., Kohn,L. and
            Rollins,J.
  CONSRTM   The Broad Institute Genome Sequencing Platform
  TITLE     Annotation of the Sclerotinia sclerotiorum 1980 genome
  JOURNAL   Unpublished
REFERENCE   2  (residues 1 to 340)
  AUTHORS   Lander,E., Birren,B. and Cuomo,C.
  CONSRTM   The Genome Sequencing Platform, The Genome Assembly Team
  TITLE     Direct Submission
  JOURNAL   Submitted (10-JUN-2005) Broad Institute of MIT and Harvard, 320
            Charles Street, Cambridge, MA 02142, USA
COMMENT     PROVISIONAL REFSEQ: This record has not yet been subject to final
            NCBI review. The reference sequence was derived from EDO04265.
            Method: conceptual translation.
FEATURES             Location/Qualifiers
     source          1..340
                     /organism="Sclerotinia sclerotiorum 1980 UF-70"
                     /strain="1980"
```

```
                     /db_xref="taxon:665079"
Protein              1..340
                     /product="hypothetical protein"
                     /calculated_mol_wt=37689
Region               33..297
                     /region_name="Aldo_ket_red"
                     /note="Aldo-keto reductases (AKRs) are a superfamily of
                     soluble NAD(P)(H) oxidoreductases whose chief purpose is
                     to reduce aldehydes and ketones to primary and secondary
                     alcohols. AKRs are present in all phyla and are of
                     importance to both health and...; cd06660"
                     /db_xref="CDD:119408"
Site                 order(48..50,72,77,102,135..136,183..184,205,231..236,268,
                     283..286,291,294..295)
                     /site_type="active"
                     /db_xref="CDD:119408"
Site                 order(72,77,102,135)
                     /site_type="other"
                     /note="catalytic tetrad"
                     /db_xref="CDD:119408"
CDS                  1..340
                     /locus_tag="SS1G_06748"
                     /coded_by="XM_001592457.1:1..1023"
                     /db_xref="GeneID:5488691"
```

Mascot: http://www.matrixscience.com/

Spot 6

{MATRIX SCIENCE} Mascot Search Results

Protein View

Match to: **gi|169625443** Score: **257** Expect: **2e-019**
hypothetical protein SNOG_15994 [Phaeosphaeria nodorum SN15]

Nominal mass (M_r): **27112**; Calculated pI value: **6.14**
NCBI BLAST search of gi|169625443 against nr
Unformatted sequence string for pasting into other applications

Taxonomy: Phaeosphaeria nodorum SN15
Links to retrieve other entries containing this sequence from NCBI Entrez:
gi|111055453 from Phaeosphaeria nodorum SN15

Fixed modifications: Carbamidomethyl (C)
Variable modifications: Oxidation (M)
Cleavage by Trypsin: cuts C-term side of KR unless next residue is P
Sequence Coverage: **23%**

Matched peptides shown in **Bold Red**

```
  1 MARQFFVGGN FKMNGTIKSI KEILGHLSQA KLDPNTEVVV APPALYLLLA
 51 REHLRPGLEV AAQNIFDKPS GAFTGEISAD QLKDSGITWT ILGHSERRTI
101 LNEDDAFVAS KTKAALDCGL GVILCCGESL EQREANKTIE VVTKQLKAVA
151 DKVKDWSKIV VAYEPIWAIG TGKVATTEQA QEVHKAIREW LQKEVSAEAA
201 EKTRILYGGS VSEKNCNELA KQPDIDGFLV GGASLKPAFV DIINAKQA
```

Show predicted peptides also

Sort Peptides By ◉ Residue Number ○ Increasing Mass ○ Decreasing Mass

Start - End	Observed	Mr(expt)	Mr(calc)	ppm	Miss	Sequence
2 - 12	1270.5781	1269.5708	1269.6619	-72	1	M.ARQFFVGGNFK.M (No match)
4 - 12	1043.5726	1042.5653	1042.5236	40	0	R.QFFVGGNFK.M (Ions score 76)
84 - 97	1571.8567	1570.8494	1570.7740	48	0	K.DSGITWTILGHSER.R (Ions score 113)
84 - 97	1571.8567	1570.8494	1570.7740	48	0	K.DSGITWTILGHSER.R (No match)
84 - 98	1727.9580	1726.9507	1726.8751	44	1	K.DSGITWTILGHSERR.T (No match)
159 - 173	1616.9719	1615.9646	1615.8974	42	0	K.IVVAYEPIWAIGTGK.V (Ions score 26)
159 - 173	1616.9719	1615.9646	1615.8974	42	0	K.IVVAYEPIWAIGTGK.V (No match)
186 - 193	1043.5726	1042.5653	1042.5923	-26	1	K.AIREWLQK.E (No match)
205 - 214	1052.6038	1051.5965	1051.5550	40	0	R.ILYGGSVSEK.N (No match)

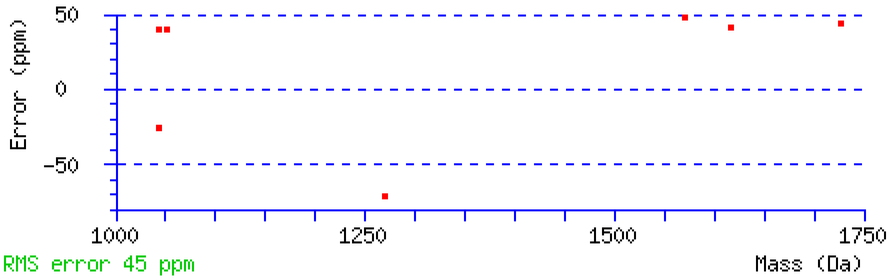


```
LOCUS       XP_001806125             248 aa            linear   PLN 02-APR-2008
DEFINITION  hypothetical protein SNOG_15994 [Phaeosphaeria nodorum SN15].
ACCESSION   XP_001806125
VERSION     XP_001806125.1  GI:169625443
DBSOURCE    REFSEQ: accession XM_001806073.1
KEYWORDS    .
SOURCE      Phaeosphaeria nodorum SN15
  ORGANISM  Phaeosphaeria nodorum SN15
            Eukaryota; Fungi; Dikarya; Ascomycota; Saccharomyceta;
            Pezizomycotina; Leotiomyceta; Dothideomyceta; Dothideomycetes;
            Pleosporomycetidae; Pleosporales; Pleosporineae; Phaeosphaeriaceae;
            Phaeosphaeria.
REFERENCE   1  (residues 1 to 248)
  AUTHORS   Birren,B., Lander,E., Galagan,J., Devon,K., Nusbaum,C., Jaffe,D.,
            Butler,J., Alvarez,P., Gnerre,S., Grabherr,M., Kleber,M.,
            Mauceli,E., Brockman,W., Rounsley,S., Young,S., LaButti,K.,
            Pushparaj,V., DeCaprio,D., Crawford,M., Koehrsen,M., Engels,R.,
            Montgomery,P., Pearson,M., Howarth,C., Kodira,C., Zeng,Q.,
            Yandava,C., Alvarado,L., Oleary,S., Oliver,R.O. and Solomon,P.
  CONSRTM   The Broad Institute Genome Sequencing Platform
  TITLE     Annotation of the Phaeosphaeria nodorum SN15 genome
  JOURNAL   Unpublished
REFERENCE   2  (residues 1 to 248)
  AUTHORS   Lander,E. and Birren,B.
  CONSRTM   The Genome Sequencing Platform, The Genome Assembly Team
  TITLE     Direct Submission
  JOURNAL   Submitted (05-MAR-2008) Broad Institute of MIT and Harvard, 320
            Charles Street, Cambridge, MA 02141, USA
  REMARK    Direct Submission
REFERENCE   3  (residues 1 to 248)
  AUTHORS   Oliver,R. and Solomon,P.
  TITLE     Direct Submission
  JOURNAL   Submitted (05-MAR-2008) Murdoch University, South Street, Perth, WA
            6150, Australia
  REMARK    Direct Submission
```

```
COMMENT     PROVISIONAL REFSEQ: This record has not yet been subject to final
            NCBI review. The reference sequence was derived from EAT76573.
            Method: conceptual translation.
FEATURES             Location/Qualifiers
     source          1..248
                     /organism="Phaeosphaeria nodorum SN15"
                     /db_xref="taxon:321614"
     Protein         1..248
                     /product="hypothetical protein"
                     /calculated_mol_wt=26770
     Region          5..244
                     /region_name="TIM"
                     /note="Triosephosphate isomerase (TIM) is a glycolytic
                     enzyme that catalyzes the interconversion of
                     dihydroxyacetone phosphate and
                     D-glyceraldehyde-3-phosphate. The reaction is very
                     efficient and requires neither cofactors nor metal ions.
                     TIM, usually...; cd00311"
                     /db_xref="CDD:73362"
     Site            order(10,12,94,164,170,210,229,231..232)
                     /site_type="other"
                     /note="substrate binding site"
                     /db_xref="CDD:73362"
     Site            order(10,13,44..46,48,51,63,81,84..85,96..97)
                     /site_type="other"
                     /note="dimer interface"
                     /db_xref="CDD:73362"
     Site            order(12,94,164)
                     /site_type="other"
                     /note="catalytic triad"
                     /db_xref="CDD:73362"
     CDS             1..248
                     /locus_tag="SNOG_15994"
                     /coded_by="XM_001806073.1:64..810"
                     /inference="ab initio prediction:Unveil:1.0"
                     /inference="similar to RNA sequence, EST (same
                     species):INSD:DR045396.1"
                     /inference="similar to RNA sequence, EST (same
                     species):INSD:DR045883.1"
                     /inference="similar to RNA sequence, EST (same
                     species):INSD:EH391712.1"
                     /inference="similar to RNA sequence, EST (same
                     species):INSD:EH395067.1"
                     /inference="similar to RNA sequence, EST (same
                     species):INSD:EH395816.1"
                     /inference="similar to RNA sequence, EST (same
                     species):INSD:EH396096.1"
                     /inference="similar to RNA sequence, EST (same
                     species):INSD:EH396292.1"
```

```
/inference="similar to RNA sequence, EST (same
species):INSD:EH396491.1"
/inference="similar to RNA sequence, EST (same
species):INSD:EH396642.1"
/inference="similar to RNA sequence, EST (same
species):INSD:EH397193.1"
/inference="similar to RNA sequence, EST (same
species):INSD:EH397293.1"
/inference="similar to RNA sequence, EST (same
species):INSD:EH397591.1"
/inference="similar to RNA sequence, EST (same
species):INSD:EH397667.1"
/inference="similar to RNA sequence, EST (same
species):INSD:EH399506.1"
/inference="similar to RNA sequence, EST (same
species):INSD:EH399520.1"
/inference="similar to RNA sequence, EST (same
species):INSD:EH399782.1"
/inference="similar to RNA sequence, EST (same
species):INSD:EH399798.1"
/inference="similar to RNA sequence, EST (same
species):INSD:EH400091.1"
/inference="similar to RNA sequence, EST (same
species):INSD:EH400187.1"
/inference="similar to RNA sequence, EST (same
species):INSD:EH400270.1"
/inference="similar to RNA sequence, EST (same
species):INSD:EH401081.1"
/note="gene prediction version 2"
/db_xref="GeneID:5983055"
```

Mascot: http://www.matrixscience.com/

Spot 7

MATRIX SCIENCE Mascot Search Results

Protein View

Match to: **gi|46581334** Score: **112** Expect: **6.3e-005**
hypothetical protein DVU2930 [Desulfovibrio vulgaris subsp. vulgaris str. Hildenborough]

Nominal mass (M_r): **16788**; Calculated pI value: **6.30**
NCBI BLAST search of gi|46581334 against nr
Unformatted sequence string for pasting into other applications

Taxonomy: Desulfovibrio vulgaris str. Hildenborough
Links to retrieve other entries containing this sequence from NCBI Entrez:
gi|46450756 from Desulfovibrio vulgaris str. Hildenborough

Fixed modifications: Carbamidomethyl (C)
Variable modifications: Oxidation (M)
Cleavage by Trypsin: cuts C-term side of KR unless next residue is P
Sequence Coverage: **23%**

Matched peptides shown in **Bold Red**

```
  1 MVLRVAVDID DREALRNLLR DGAQLKLTLA ATLKRSRTLM PAEEVGVVVA
 51 EILMRHDPLT REFEFSTSPD LPHRRDRNFN RLMDATLERL RLPLAPAASL
101 AQGESYTVSL TCGLRHTQVP PWLEKTLFFW SWDVVPESTY TQSFTF
```

Show predicted peptides also

Sort Peptides By ◉ Residue Number ○ Increasing Mass ○ Decreasing Mass

Start - End	Observed	Mr(expt)	Mr(calc)	ppm	Miss	Sequence
2 - 12	1270.7087	1269.7014	1269.7041	-2	1	M.VLRVAVDIDDR.E (No match)
2 - 12	1270.7087	1269.7014	1269.7041	-2	1	M.VLRVAVDIDDR.E (No match)
5 - 12	902.4884	901.4811	901.4505	34	0	R.VAVDIDDR.E (No match)
13 - 20	984.5458	983.5385	983.5876	-50	1	R.EALRNLLR.D (No match)
21 - 34	1442.7502	1441.7429	1441.8504	-75	1	R.DGAQLKLTLAATLK.R (No match)
27 - 34	830.4565	829.4492	829.5273	-94	0	K.LTLAATLK.R (Ions score 38)
27 - 35	986.5621	985.5548	985.6284	-75	1	K.LTLAATLKR.S (Ions score 39)
27 - 35	986.5621	985.5548	985.6284	-75	1	K.LTLAATLKR.S (No match)

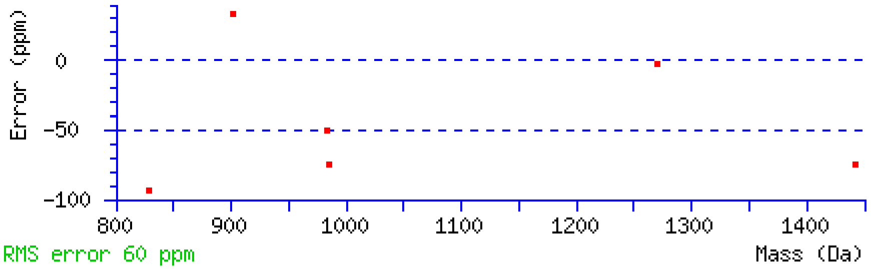


```
LOCUS       YP_012142                146 aa            linear   BCT 29-MAR-2010
DEFINITION  hypothetical protein DVU2930 [Desulfovibrio vulgaris subsp.
            vulgaris str. Hildenborough].
ACCESSION   YP_012142
VERSION     YP_012142.1  GI:46581334
DBLINK      Project: 51
DBSOURCE    REFSEQ: accession NC_002937.3
KEYWORDS    .
SOURCE      Desulfovibrio vulgaris str. Hildenborough
  ORGANISM  Desulfovibrio vulgaris str. Hildenborough
            Bacteria; Proteobacteria; Deltaproteobacteria; Desulfovibrionales;
            Desulfovibrionaceae; Desulfovibrio.
REFERENCE   1  (residues 1 to 146)
  AUTHORS   Heidelberg,J.F., Seshadri,R., Haveman,S.A., Hemme,C.L.,
            Paulsen,I.T., Kolonay,J.F., Eisen,J.A., Ward,N., Methe,B.,
            Brinkac,L.M., Daugherty,S.C., Deboy,R.T., Dodson,R.J., Durkin,A.S.,
            Madupu,R., Nelson,W.C., Sullivan,S.A., Fouts,D., Haft,D.H.,
            Selengut,J., Peterson,J.D., Davidsen,T.M., Zafar,N., Zhou,L.,
            Radune,D., Dimitrov,G., Hance,M., Tran,K., Khouri,H., Gill,J.,
            Utterback,T.R., Feldblyum,T.V., Wall,J.D., Voordouw,G. and
            Fraser,C.M.
  TITLE     The genome sequence of the anaerobic, sulfate-reducing bacterium
            Desulfovibrio vulgaris Hildenborough
  JOURNAL   Nat. Biotechnol. 22 (5), 554-559 (2004)
   PUBMED   15077118
REFERENCE   2  (residues 1 to 146)
  AUTHORS   Heidelberg,J.F., Seshadri,R., Haveman,S.A., Hemme,C.L.,
            Paulsen,I.T., Kolonay,J.F., Eisen,J.A., Ward,N., Methe,B.,
            Brinkac,L.M., Daugherty,S.C., DeBoy,R.T., Dodson,R.J., Durkin,A.S.,
            Madupu,R., Nelson,W.C., Sullivan,S.A., Fouts,D.E., Haft,D.H.,
            Selengut,J., Peterson,J.D., Davidsen,T.M., Zafar,N., Zhou,L.,
            Radune,D., Dimitrov,G., Hance,M., Tran,K., Khouri,H.M., Gill,J.,
            Utterback,T.R., Feldblyum,T.V., Wall,J.D., Voordouw,G. and
            Fraser,C.M.
  TITLE     Direct Submission
```

```
  JOURNAL   Submitted (18-MAR-2004) The Institute for Genomic Research, 9712
            Medical Center Dr, Rockville, MD 20850, USA
REFERENCE   3  (residues 1 to 146)
  CONSRTM   NCBI Genome Project
  TITLE     Direct Submission
  JOURNAL   Submitted (15-MAR-2004) National Center for Biotechnology
            Information, NIH, Bethesda, MD 20894, USA
COMMENT     PROVISIONAL REFSEQ: This record has not yet been subject to final
            NCBI review. The reference sequence was derived from AAS97402.
            Method: conceptual translation.
FEATURES             Location/Qualifiers
     source          1..146
                     /organism="Desulfovibrio vulgaris str. Hildenborough"
                     /strain="Hildenborough"
                     /sub_species="vulgaris"
                     /db_xref="taxon:882"
     Protein         1..146
                     /product="hypothetical protein"
                     /calculated_mol_wt=16610
     CDS             1..146
                     /locus_tag="DVU2930"
                     /coded_by="NC_002937.3:3033771..3034211"
                     /inference="non-experimental evidence, no additional
                     details recorded"
                     /transl_table=11
                     /db_xref="GeneID:2793968"
```

Mascot: http://www.matrixscience.com/

Spot 8

Mascot Search Results

Protein View

Match to: **gi|189210148** Score: **117** Expect: **2e-005**
signal transduction protein [Pyrenophora tritici-repentis Pt-1C-BFP]

Nominal mass (M_r): **14872**; Calculated pI value: **4.85**
NCBI BLAST search of gi|189210148 against nr
Unformatted sequence string for pasting into other applications

Taxonomy: Pyrenophora tritici-repentis Pt-1C-BFP
Links to retrieve other entries containing this sequence from NCBI Entrez:
gi|187977499 from Pyrenophora tritici-repentis Pt-1C-BFP

Fixed modifications: Carbamidomethyl (C)
Variable modifications: Oxidation (M)
Cleavage by Trypsin: cuts C-term side of KR unless next residue is P
Sequence Coverage: **19%**

Matched peptides shown in **Bold Red**

1 MGFWDNKGEN YDQVYNNDNF EENKSSLGHE VIVGGAAFAG FK**AFEDHQR**N
51 EGKPVSHAFA K**ELLAGFAAA EVDKLAETK**G EDWFDREKAK RDAKKHAEQM
101 YDDHYVDNHG ADQYDPNQYS GPQQFDNRSW

Show predicted peptides also

Sort Peptides By ◉ Residue Number ○ Increasing Mass ○ Decreasing Mass

Start - End	Observed	Mr(expt)	Mr(calc)	ppm	Miss	Sequence
43 - 49	902.4146	901.4073	901.4042	3	0	K.AFEDHQR.N (Ions score 36)
43 - 49	902.4146	901.4073	901.4042	3	0	K.AFEDHQR.N (No match)
62 - 74	1333.6974	1332.6901	1332.6925	-2	0	K.ELLAGFAAAEVDK.L (No match)
62 - 79	1876.0060	1874.9987	1874.9989	-0	1	K.ELLAGFAAAEVDKLAETK.G (Ions score 60)
62 - 79	1876.0060	1874.9987	1874.9989	-0	1	K.ELLAGFAAAEVDKLAETK.G (No match)

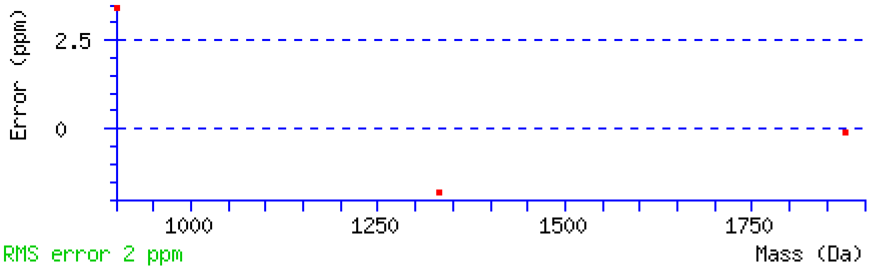

```
LOCUS       XP_001941406             130 aa            linear   PLN 30-MAY-2008
DEFINITION  signal transduction protein [Pyrenophora tritici-repentis
            Pt-1C-BFP].
ACCESSION   XP_001941406
VERSION     XP_001941406.1  GI:189210148
DBSOURCE    REFSEQ: accession XM_001941371.1
KEYWORDS    .
SOURCE      Pyrenophora tritici-repentis Pt-1C-BFP
  ORGANISM  Pyrenophora tritici-repentis Pt-1C-BFP
            Eukaryota; Fungi; Dikarya; Ascomycota; Saccharomyceta;
            Pezizomycotina; Leotiomyceta; Dothideomyceta; Dothideomycetes;
            Pleosporomycetidae; Pleosporales; Pleosporineae; Pleosporaceae;
            Pyrenophora.
REFERENCE   1  (residues 1 to 130)
  AUTHORS   Birren,B., Lander,E., Galagan,J., Nusbaum,C., Devon,K., Ma,L.-J.,
            Jaffe,D., Butler,J., Alvarez,P., Gnerre,S., Grabherr,M., Kleber,M.,
            Mauceli,E., Brockman,W., MacCallum,I.A., Young,S., LaButti,K.,
            DeCaprio,D., Crawford,M., Koehrsen,M., Engels,R., Montgomery,P.,
            Pearson,M., Howarth,C., Larson,L., White,J., Yandava,C., Kodira,C.,
            Guigo,R., Borodovsky,M., Zeng,Q., O'Leary,S., Alvarado,L.,
            Pandelova,I. and Ciuffetti,L.
  CONSRTM   The Broad Institute Genome Sequencing Platform
  TITLE     Genome Sequence of Pyrenophora tritici-repentis
  JOURNAL   Unpublished
REFERENCE   2  (residues 1 to 130)
  AUTHORS   Birren,B., Lander,E., Galagan,J., Nusbaum,C., Devon,K., Ma,L.-J.,
            Jaffe,D., Butler,J., Alvarez,P., Gnerre,S., Grabherr,M., Kleber,M.,
            Mauceli,E., Brockman,W., MacCallum,I.A., Young,S., LaButti,K.,
            DeCaprio,D., Crawford,M., Koehrsen,M., Engels,R., Montgomery,P.,
            Pearson,M., Howarth,C., Larson,L., White,J., Yandava,C., Kodira,C.,
            Zeng,Q., O'Leary,S., Alvarado,L., Ciuffetti,L. and Pandelova,I.
  CONSRTM   The Broad Institute Genome Sequencing Platform
  TITLE     Direct Submission
  JOURNAL   Submitted (16-MAR-2007) Broad Institute of MIT and Harvard, 7
            Cambridge Center, Cambridge, MA 02142, USA
```

```
COMMENT     PROVISIONAL REFSEQ: This record has not yet been subject to final
            NCBI review. The reference sequence was derived from EDU44125.
            Method: conceptual translation.
FEATURES             Location/Qualifiers
     source          1..130
                     /organism="Pyrenophora tritici-repentis Pt-1C-BFP"
                     /strain="Pt-1C-BFP"
                     /db_xref="taxon:426418"
     Protein         1..130
                     /product="signal transduction protein"
                     /calculated_mol_wt=14750
     Region          9..105
                     /region_name="DUF3759"
                     /note="Protein of unknown function (DUF3759); pfam12585"
                     /db_xref="CDD:153019"
     CDS             1..130
                     /locus_tag="PTRG_11075"
                     /coded_by="XM_001941371.1:72..464"
                     /db_xref="GeneID:6349387"
```

Mascot: http://www.matrixscience.com/

Spot 9

Mascot Search Results

Protein View

Match to: **gi|115400267** Score: **165** Expect: **3.2e-010**
transaldolase [Aspergillus terreus NIH2624]

Nominal mass (M_r): **35206**; Calculated pI value: **6.16**
NCBI BLAST search of gi|115400267 against nr
Unformatted sequence string for pasting into other applications

Taxonomy: Aspergillus terreus NIH2624
Links to retrieve other entries containing this sequence from NCBI Entrez:
gi|114191388 from Aspergillus terreus NIH2624

Fixed modifications: Carbamidomethyl (C)
Variable modifications: Oxidation (M)
Cleavage by Trypsin: cuts C-term side of KR unless next residue is P
Sequence Coverage: **18%**

Matched peptides shown in **Bold Red**

```
  1 MSSSLEQLKA TGTVVVCDSA IGKYKPQDAT TNPSLILAAS KKPEYAALID
 51 AAVAYGKQHG KTVDEQVDAT LDRLLVEFGK EILKIIPGKV STEVDARFSF
101 DTQASIDKAL HIIKLYEEIG IPKDRILIKI ASTWEGIKAA QVLQSQHGIN
151 CNLTLMFSTV QAIAAAEAGA YLISPFVGRI LDWYKAAHKR DYTAQEDPGV
201 KSVQAIFNYY KKYGYKTIVM GASFRNTGEI TELAGCDYLT ISPNLLEDLY
251 NSTAAVPKKL DAAAAASQDI PKRSYINDEA LFRFDFNEEA MAVEKLREGI
301 SKFAADAVTL KDLLKQKIQA
```

Show predicted peptides also

Sort Peptides By ◉ Residue Number ○ Increasing Mass ○ Decreasing Mass

Start - End	Observed	Mr(expt)	Mr(calc)	ppm	Miss	Sequence
42 - 57	1679.8977	1678.8904	1678.8930	-2	0	K.KPEYAALIDAAVAYGK.Q (Ions score 135)
42 - 57	1679.8977	1678.8904	1678.8930	-2	0	K.KPEYAALIDAAVAYGK.Q (No match)
74 - 80	805.5040	804.4967	804.4745	28	0	R.LLVEFGK.E (No match)
85 - 97	1384.7795	1383.7722	1383.7722	0	1	K.IIPGKVSTEVDAR.F (No match)
90 - 97	876.4454	875.4381	875.4349	4	0	K.VSTEVDAR.F (No match)
130 - 138	1004.5442	1003.5369	1003.5338	3	0	K.IASTWEGIK.A (No match)
303 - 315	1404.8094	1403.8021	1403.8024	-0	1	K.FAADAVTLKDLLK.Q (No match)

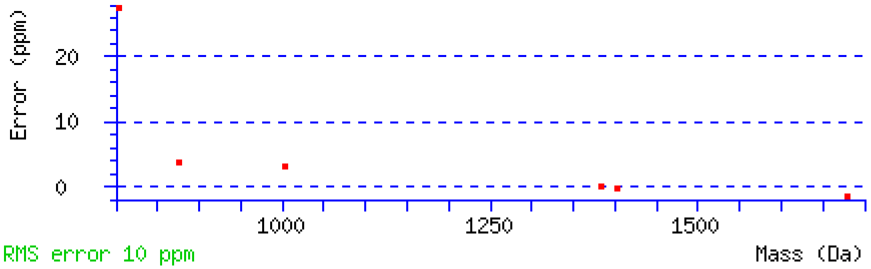


```
LOCUS       XP_001215722             320 aa            linear   PLN 23-APR-2008
DEFINITION  transaldolase [Aspergillus terreus NIH2624].
ACCESSION   XP_001215722
VERSION     XP_001215722.1  GI:115400267
DBSOURCE    REFSEQ: accession XM_001215722.1
KEYWORDS    .
SOURCE      Aspergillus terreus NIH2624
  ORGANISM  Aspergillus terreus NIH2624
            Eukaryota; Fungi; Dikarya; Ascomycota; Saccharomyceta;
            Pezizomycotina; Leotiomyceta; Eurotiomycetes; Eurotiomycetidae;
            Eurotiales; Trichocomaceae; mitosporic Trichocomaceae; Aspergillus.
REFERENCE   1  (residues 1 to 320)
  AUTHORS   Birren,B., Lander,E., Galagan,J., Nusbaum,C., Devon,K., Henn,M.,
            Ma,L.-J., Jaffe,D., Butler,J., Alvarez,P., Gnerre,S., Grabherr,M.,
            Kleber,M., Mauceli,E., Brockman,W., Rounsley,S., Young,S.,
            LaButti,K., Pushparaj,V., DeCaprio,D., Crawford,M., Koehrsen,M.,
            Engels,R., Montgomery,P., Pearson,M., Howarth,C., Larson,L.,
            Luoma,S., White,J., Alvarado,L., Kodira,C., Zeng,Q., Oleary,S.,
            Yandava,C., Denning,D., Nierman,B., Milne,T. and Madden,K.
  CONSRTM   The Broad Institute Genome Sequencing Platform
  TITLE     Annotation of the Aspergillus terreus NIH2624 genome
  JOURNAL   Unpublished
REFERENCE   2  (residues 1 to 320)
  AUTHORS   Birren,B., Lander,E., Galagan,J., Devon,K., Nusbaum,C., Henn,M.,
            Borowsky,M., Jaffe,D., Butler,J., Alvarez,P., Gnerre,S.,
            Grabherr,M., Kleber,M., Mauceli,E., Brockman,W., Rounsley,S.,
            Young,S., LaButti,K., Pushparaj,V., DeCaprio,D., Crawford,M.,
            Koehrsen,M., Engels,R., Montgomery,P., Pearson,M., Howarth,C.,
            Kodira,C., Zeng,Q., Yandava,C., Oleary,S. and Alvarado,L.
  TITLE     Direct Submission
  JOURNAL   Submitted (02-SEP-2005) Broad Institute of MIT and Harvard, 320
            Charles Street, Cambridge, MA 02141, USA
REFERENCE   3  (residues 1 to 320)
  AUTHORS   Denning,D. and Anderson,M.
  TITLE     Direct Submission
```

```
  JOURNAL   Submitted (02-SEP-2005) The University of Manchester, Oxford Road,
            Manchester M13 9PT, UK
REFERENCE   4  (residues 1 to 320)
  AUTHORS   Nierman,W.C.
  TITLE     Direct Submission
  JOURNAL   Submitted (02-SEP-2005) The Institute for Genomic Research, 9712
            Medical Center Drive, Rockville, MD 20850, USA
COMMENT     PROVISIONAL REFSEQ: This record has not yet been subject to final
            NCBI review. The reference sequence was derived from EAU33088.
            Method: conceptual translation.
FEATURES             Location/Qualifiers
     source          1..320
                     /organism="Aspergillus terreus NIH2624"
                     /strain="NIH2624"
                     /db_xref="taxon:341663"
     Protein         1..320
                     /product="transaldolase"
                     /calculated_mol_wt=34926
     Region          3..314
                     /region_name="Transaldolase_TalAB"
                     /note="Transaldolases including both TalA and TalB. The
                     enzyme catalyses the reversible transfer of a
                     dyhydroxyacetone moiety, derived from fructose-6-phosphate
                     to erythrose-4-phosphate yielding
                     sedoheptulose-7-phosphate and glyceraldehyde-3-phosphate.
                     The...; cd00957"
                     /db_xref="CDD:29950"
     Site            order(18,30,32..33,91,93,129,152,154,174,179)
                     /site_type="active"
                     /db_xref="CDD:29950"
     Site            order(101,280,283,287..288,294,297,301)
                     /site_type="other"
                     /note="dimer interface"
                     /db_xref="CDD:29950"
     Site            129
                     /site_type="other"
                     /note="catalytic residue"
                     /db_xref="CDD:29950"
     CDS             1..320
                     /locus_tag="ATEG_06544"
                     /coded_by="XM_001215722.1:1..963"
                     /db_xref="GeneID:4322187"
```

Mascot: http://www.matrixscience.com/

Spot 10

Mascot Search Results

Protein View

Match to: **gi|85107722** Score: **217** Expect: **2e-015**
hypothetical protein NCU07914 [Neurospora crassa OR74A]

Nominal mass (M_r): **45257**; Calculated pI value: **6.17**
NCBI BLAST search of gi|85107722 against nr
Unformatted sequence string for pasting into other applications

Taxonomy: Neurospora crassa OR74A
Links to retrieve other entries containing this sequence from NCBI Entrez:
gi|52788211 from Neurospora crassa
gi|28924036 from Neurospora crassa OR74A

Fixed modifications: Carbamidomethyl (C)
Variable modifications: Oxidation (M)
Cleavage by Trypsin: cuts C-term side of KR unless next residue is P
Sequence Coverage: **22%**

Matched peptides shown in **Bold Red**

```
  1 MSLSNKLSIE DVDLKGKRVL IRVDFNVPLD AEKKVTNPQR IAGAIPTIKY
 51 ALDHGAKAVV LMSHLGRPDG KPNPKYSLKP VVPELEKLLG KKVTFAPDCV
101 GPEVEEIVNK ADNGEVILLE NLRFHIEEEG KGTDAEGNKV KADKAKVEEF
151 RKNLTKLGDV YINDAFGTAH RAHSSMVGID LPVKAAGFLM KKELQYFAKV
201 LESPQRPFLS ILGGAKVSDK IQLIDNLLDK VNTLIVCGGM AFTFKKTLQN
251 MPIGNSLFDE AGAKIVPDLV KKAEKNNVKL VLPVDFTIAD KFDKDANTGY
301 ATDKDGIPDG WMGLDCGEES VKLFTQAINE SQTILWNGPA GVFEFDKFAK
351 GTKATLDACV KAAEEGRTVI IGGGDTATVA AKYGVEDKLS HVSTGGGASL
401 ELLEGKALPG VVALSERQ
```

Show predicted peptides also

Sort Peptides By ◉ Residue Number ○ Increasing Mass ○ Decreasing Mass

Start - End	Observed	Mr(expt)	Mr(calc)	ppm	Miss	Sequence
1 - 15	1707.8717	1706.8644	1706.8760	-7	1	-.MSLSNKLSIEDVDLK.G Oxidation (M) (No match)
19 - 33	1727.9666	1726.9593	1726.9618	-1	1	R.VLIRVDFNVPLDAEK.K (No match)
23 - 33	1246.6361	1245.6288	1245.6241	4	0	R.VDFNVPLDAEK.K (No match)
23 - 34	1374.7245	1373.7172	1373.7191	-1	1	R.VDFNVPLDAEKK.V (No match)
50 - 57	874.4529	873.4456	873.4344	13	0	K.YALDHGAK.A (No match)
157 - 171	1648.8069	1647.7996	1647.8005	-1	0	K.LGDVYINDAFGTAHR.A (Ions score 163)

157 - 171	1648.8069	1647.7996	1647.8005	-1	0	K.LGDVYINDAFGTAHR.A	(No match)
185 - 192	881.4696	880.4623	880.4840	-25	1	K.AAGFLMKK.E	Oxidation (M) (No match)
265 - 272	911.5916	910.5843	910.5851	-1	1	K.IVPDLVKK.A	(No match)
383 - 406	2446.2434	2445.2361	2445.2387	-1	1	K.YGVEDKLSHVSTGGGASLELLEGK.A	(No match)
389 - 406	1754.9246	1753.9173	1753.9210	-2	0	K.LSHVSTGGGASLELLEGK.A	(No match)

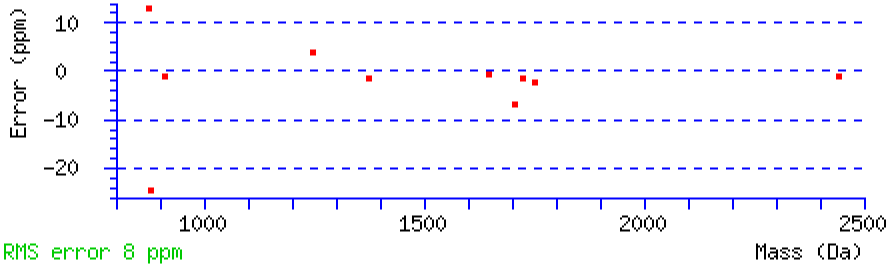

RMS error 8 ppm

```
LOCUS       XP_962430                418 aa            linear   PLN 10-APR-2008
DEFINITION  hypothetical protein NCU07914 [Neurospora crassa OR74A].
ACCESSION   XP_962430
VERSION     XP_962430.1  GI:85107722
DBSOURCE    REFSEQ: accession XM_957337.2
KEYWORDS    .
SOURCE      Neurospora crassa OR74A
  ORGANISM  Neurospora crassa OR74A
            Eukaryota; Fungi; Dikarya; Ascomycota; Saccharomyceta;
            Pezizomycotina; Leotiomyceta; Sordariomyceta; Sordariomycetes;
            Sordariomycetidae; Sordariales; Sordariaceae; Neurospora.
REFERENCE   1  (residues 1 to 418)
  AUTHORS   Galagan,J.E., Calvo,S.E., Borkovich,K.A., Selker,E.U., Read,N.D.,
            Jaffe,D., FitzHugh,W., Ma,L.J., Smirnov,S., Purcell,S., Rehman,B.,
            Elkins,T., Engels,R., Wang,S., Nielsen,C.B., Butler,J.,
            Endrizzi,M., Qui,D., Ianakiev,P., Bell-Pedersen,D., Nelson,M.A.,
            Werner-Washburne,M., Selitrennikoff,C.P., Kinsey,J.A., Braun,E.L.,
            Zelter,A., Schulte,U., Kothe,G.O., Jedd,G., Mewes,W., Staben,C.,
            Marcotte,E., Greenberg,D., Roy,A., Foley,K., Naylor,J.,
            Stange-Thomann,N., Barrett,R., Gnerre,S., Kamal,M., Kamvysselis,M.,
            Mauceli,E., Bielke,C., Rudd,S., Frishman,D., Krystofova,S.,
            Rasmussen,C., Metzenberg,R.L., Perkins,D.D., Kroken,S., Cogoni,C.,
            Macino,G., Catcheside,D., Li,W., Pratt,R.J., Osmani,S.A.,
            DeSouza,C.P., Glass,L., Orbach,M.J., Berglund,J.A., Voelker,R.,
            Yarden,O., Plamann,M., Seiler,S., Dunlap,J., Radford,A.,
            Aramayo,R., Natvig,D.O., Alex,L.A., Mannhaupt,G., Ebbole,D.J.,
            Freitag,M., Paulsen,I., Sachs,M.S., Lander,E.S., Nusbaum,C. and
            Birren,B.
  TITLE     The genome sequence of the filamentous fungus Neurospora crassa
  JOURNAL   Nature 422 (6934), 859-868 (2003)
```

```
   PUBMED   12712197
REFERENCE   2  (residues 1 to 418)
  AUTHORS   Galagan,J., Henn,M.R., Hood,H., Radford,A., Collins,R.,
            DeCaprio,D., Crawford,M., Koehrsen,M., Engels,R., Montgomery,P.,
            Pearson,M., Howarth,C., Larson,L., White,J., Ledlie,T., Kodira,C.,
            Zeng,Q., Yandava,C., Alvarado,L., O'Leary,S., Bowman,B., Colot,H.,
            Ebbole,D., Rasmussen,C., Baker,C., Kalkman,E., Chen,C.-H., Shi,M.,
            Mathur,R., Lambreghts,R., mehra,A., Collopy,P., Mehra,A.,
            Schweredtfeger,C., Hong,C., Belden,W., Glass,N.L., Borkovich,K.,
            Dunlap,J., Lander,E., Nusbaum,C., Sachs,M. and Birren,B.
  TITLE     Version 3 gene predictions for the Neurospora crassa assembly 7
  JOURNAL   Unpublished
REFERENCE   3  (residues 1 to 418)
  AUTHORS   Birren,B., Galagan,J. and Henn,M.R.
  TITLE     Direct Submission
  JOURNAL   Submitted (06-JUL-2007) Broad Institute of MIT and Harvard, 7
            Cambridge Center, Cambridge, MA 02142, USA
REFERENCE   4  (residues 1 to 418)
  AUTHORS   Birren,B.
  TITLE     Direct Submission
  JOURNAL   Submitted (11-MAR-2003) Whitehead Institute/MIT Center for Genome
            Research, 320 Charles Street, Cambridge, MA 02142, USA
COMMENT     PROVISIONAL REFSEQ: This record has not yet been subject to final
            NCBI review. The reference sequence was derived from EAA33194.
            Method: conceptual translation.
FEATURES             Location/Qualifiers
     source          1..418
                     /organism="Neurospora crassa OR74A"
                     /strain="OR74A"
                     /db_xref="taxon:367110"
                     /chromosome="IV"
     Protein         1..418
                     /product="hypothetical protein"
                     /name="similar to phosphoglycerate kinase 1"
                     /calculated_mol_wt=44926
     Region          9..416
                     /region_name="Phosphoglycerate_kinase"
                     /note="Phosphoglycerate kinase (PGK) is a monomeric enzyme
                     which catalyzes the transfer of the high-energy phosphate
                     group of 1,3-bisphosphoglycerate to ADP, forming ATP and
                     3-phosphoglycerate. This reaction represents the first of
                     the two substrate-level...; cd00318"
                     /db_xref="CDD:29400"
     Site            order(24,26,40,64,123)
                     /site_type="other"
                     /note="substrate binding site"
                     /db_xref="CDD:29400"
     Site            order(201..204,392..394)
                     /site_type="other"
```

```
                     /note="hinge regions"
                     /db_xref="CDD:29400"
     Site            order(239,313,337,339,341..344,374..376)
                     /site_type="other"
                     /note="ADP binding site"
                     /db_xref="CDD:29400"
     Site            375
                     /site_type="other"
                     /note="catalytic site"
                     /db_xref="CDD:29400"
     CDS             1..418
                     /locus_tag="NCU07914"
                     /old_locus_tag="NCU07914.1"
                     /coded_by="XM_957337.2:107..1363"
                     /db_xref="GeneID:3878602"
```

**Mascot:** http://www.matrixscience.com/

Spot 11

Mascot Search Results

Protein View

Match to: **gi|120691** Score: **334** Expect: **4e-027**
RecName: Full=Glyceraldehyde-3-phosphate dehydrogenase; Short=GAPDH

Nominal mass (M_r): **36641**; Calculated pI value: **6.72**
NCBI BLAST search of gi|120691 against nr
Unformatted sequence string for pasting into other applications

Taxonomy: Cochliobolus lunatus
Links to retrieve other entries containing this sequence from NCBI Entrez:
gi|2601 from Cochliobolus lunatus

Fixed modifications: Carbamidomethyl (C)
Variable modifications: Oxidation (M)
Cleavage by Trypsin: cuts C-term side of KR unless next residue is P
Sequence Coverage: **23%**

Matched peptides shown in **Bold Red**

```
  1 MVVKVGINGF GRIGRIVFRN AIEHNDVEIV AVNDPFIEPH YAAYMLKYDS
 51 THGQFKGDIK VDGNNLTVNG KTVRFHMEKD PANIPWSETG AYYVVESTGV
101 FTTTEKAKAH LKGGAKKVVI SAPSADAPMF VMGVNHETYK SDIEVLSNSS
151 CTTNCLAPLA KVIHDKYTII EGLMTTIHSY TATQKVVDGP SAKDWRGGRT
201 AAQNIIPSST GAAKAVGKVI PELNGKLTGM AMRVPTANVS VVDLTVRIEK
251 GASYDEIKQA VKEASEGPLS GILGYTEDDI VTTDLNGDNR SSIFDAKAGI
301 SLNKNFVKLV SWYDNEWGYS RRVLDLLVYI AKIDGNA
```

Show predicted peptides also

Sort Peptides By Residue Number Increasing Mass Decreasing Mass

Start - End	Observed	Mr(expt)	Mr(calc)	ppm	Miss	Sequence
48 - 56	1082.4883	1081.4810	1081.4829	-2	0	K.YDSTHGQFK.G (No match)
48 - 60	1495.7140	1494.7067	1494.7103	-2	1	K.YDSTHGQFKGDIK.V (Ions score 75)
48 - 60	1495.7140	1494.7067	1494.7103	-2	1	K.YDSTHGQFKGDIK.V (No match)
72 - 79	1047.5978	1046.5905	1046.5331	55	1	K.TVRFHMEK.D (No match)
72 - 79	1047.5978	1046.5905	1046.5331	55	1	K.TVRFHMEK.D (No match)
263 - 297	3699.7375	3698.7302	3698.7435	-4	1	K.EASEGPLSGILGYTEDDIVTTDLNGDNRSSIFDAK.A (No match)
309 - 321	1674.7506	1673.7433	1673.7474	-2	0	K.LVSWYDNEWGYSR.R (Ions score 108)
309 - 321	1674.7506	1673.7433	1673.7474	-2	0	K.LVSWYDNEWGYSR.R (No match)
309 - 322	1830.8538	1829.8465	1829.8485	-1	1	K.LVSWYDNEWGYSRR.V (Ions score 43)

309 - 322	1830.8538	1829.8465	1829.8485	-1	1	K.LVSWYDNEWGYSRR.V	(No match)
322 - 332	1302.8107	1301.8034	1301.8071	-3	1	R.RVLDLLVYIAK.I	(Ions score 61)
322 - 332	1302.8107	1301.8034	1301.8071	-3	1	R.RVLDLLVYIAK.I	(No match)
323 - 332	1146.7112	1145.7039	1145.7060	-2	0	R.VLDLLVYIAK.I	(No match)

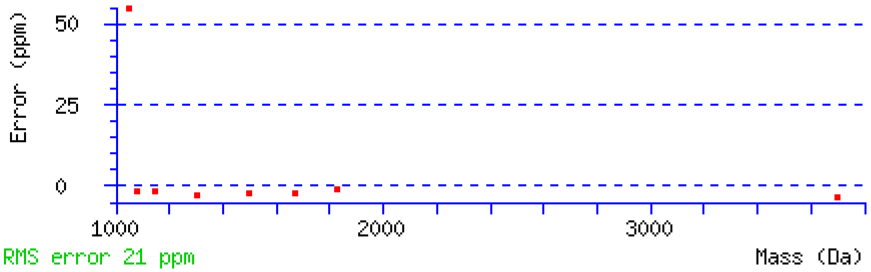


```
LOCUS       G3P_CURLU                337 aa            linear   PLN 13-JUL-2010
DEFINITION  RecName: Full=Glyceraldehyde-3-phosphate dehydrogenase;
            Short=GAPDH.
ACCESSION   P28844
VERSION     P28844.1  GI:120691
DBSOURCE    UniProtKB: locus G3P_CURLU, accession P28844;
            class: standard.
            created: Dec 1, 1992.
            sequence updated: Dec 1, 1992.
            annotation updated: Jul 13, 2010.
            xrefs: X58718.1, CAA41554.1, DEYDGC
            xrefs (non-sequence databases): SMR:P28844, BRENDA:1.2.1.12,
            GO:0005737, GO:0004365, GO:0051287, GO:0006096, GO:0055114,
            InterPro:IPR020830, InterPro:IPR020829, InterPro:IPR020832,
            InterPro:IPR020831, InterPro:IPR020828, InterPro:IPR000173,
            InterPro:IPR006424, PANTHER:PTHR10836, Pfam:PF02800, Pfam:PF00044,
            PIRSF:PIRSF000149, PRINTS:PR00078, SMART:SM00846,
            TIGRFAMs:TIGR01534, PROSITE:PS00071
KEYWORDS    Cytoplasm; Glycolysis; NAD; Oxidoreductase.
SOURCE      Cochliobolus lunatus (anamorph: Curvularia lunata)
  ORGANISM  Cochliobolus lunatus
            Eukaryota; Fungi; Dikarya; Ascomycota; Saccharomyceta;
            Pezizomycotina; Leotiomyceta; Dothideomyceta; Dothideomycetes;
            Pleosporomycetidae; Pleosporales; Pleosporineae; Pleosporaceae;
            Cochliobolus.
REFERENCE   1  (residues 1 to 337)
  AUTHORS   Osiewacz,H.D. and Ridder,R.
  TITLE     Genome analysis of imperfect fungi: electrophoretic karyotyping and
            characterization of the nuclear gene coding for
            glyceraldehyde-3-phosphate dehydrogenase (gpd) of Curvularia lunata
  JOURNAL   Curr. Genet. 20 (1-2), 151-155 (1991)
```

```
   PUBMED   1934112
  REMARK    NUCLEOTIDE SEQUENCE [GENOMIC DNA].
            STRAIN=AT46
COMMENT     On Oct 11, 2005 this sequence version replaced gi:66000.
            [CATALYTIC ACTIVITY] D-glyceraldehyde 3-phosphate + phosphate +
            NAD(+) = 3-phospho-D-glyceroyl phosphate + NADH.
            [PATHWAY] Carbohydrate degradation; glycolysis; pyruvate from
            D-glyceraldehyde 3-phosphate: step 1/5.
            [SUBUNIT] Homotetramer.
            [SUBCELLULAR LOCATION] Cytoplasm.
            [SIMILARITY] Belongs to the glyceraldehyde-3-phosphate
            dehydrogenase family.
FEATURES             Location/Qualifiers
     source          1..337
                     /organism="Cochliobolus lunatus"
                     /db_xref="taxon:5503"
     gene            1..337
                     /gene="GPD"
     Protein         1..337
                     /gene="GPD"
                     /product="Glyceraldehyde-3-phosphate dehydrogenase"
                     /EC_number="1.2.1.12"
                     /note="GAPDH"
                     /UniProtKB_evidence="Inferred from homology"
     Region          1..337
                     /gene="GPD"
                     /region_name="Mature chain"
                     /experiment="experimental evidence, no additional details
                     recorded"
                     /note="Glyceraldehyde-3-phosphate dehydrogenase.
                     /FTId=PRO_0000145552."
     Region          1..334
                     /gene="GPD"
                     /region_name="PTZ00023"
                     /note="glyceraldehyde-3-phosphate dehydrogenase;
                     Provisional; PTZ00023"
                     /db_xref="CDD:173322"
     Region          3..151
                     /gene="GPD"
                     /region_name="Gp_dh_N"
                     /note="Glyceraldehyde 3-phosphate dehydrogenase, NAD
                     binding domain; cl12058"
                     /db_xref="CDD:159718"
     Site            12..13
                     /gene="GPD"
                     /site_type="np-binding"
                     /inference="non-experimental evidence, no additional
                     details recorded"
                     /note="NAD (By similarity)."
```

```
Site            34
                /gene="GPD"
                /site_type="binding"
                /inference="non-experimental evidence, no additional
                details recorded"
                /note="NAD (By similarity)."
Site            79
                /gene="GPD"
                /site_type="binding"
                /inference="non-experimental evidence, no additional
                details recorded"
                /note="NAD; via carbonyl oxygen (By similarity)."
Region          150..152
                /gene="GPD"
                /region_name="Region of interest in the sequence"
                /inference="non-experimental evidence, no additional
                details recorded"
                /note="Glyceraldehyde 3-phosphate binding (By
                similarity)."
Site            151
                /gene="GPD"
                /site_type="active"
                /inference="non-experimental evidence, no additional
                details recorded"
                /note="Nucleophile (By similarity)."
Region          156..313
                /gene="GPD"
                /region_name="Gp_dh_C"
                /note="Glyceraldehyde 3-phosphate dehydrogenase,
                C-terminal domain; pfam02800"
                /db_xref="CDD:145777"
Site            178
                /gene="GPD"
                /site_type="other"
                /inference="non-experimental evidence, no additional
                details recorded"
                /note="Activates thiol group during catalysis (By
                similarity)."
Site            181
                /gene="GPD"
                /site_type="binding"
                /inference="non-experimental evidence, no additional
                details recorded"
                /note="Glyceraldehyde 3-phosphate (By similarity)."
Region          210..211
                /gene="GPD"
                /region_name="Region of interest in the sequence"
                /inference="non-experimental evidence, no additional
                details recorded"
```

```
                   /note="Glyceraldehyde 3-phosphate binding (By
                   similarity)."
     Site          233
                   /gene="GPD"
                   /site_type="binding"
                   /inference="non-experimental evidence, no additional
                   details recorded"
                   /note="Glyceraldehyde 3-phosphate (By similarity)."
     Site          315
                   /gene="GPD"
                   /site_type="binding"
                   /inference="non-experimental evidence, no additional
                   details recorded"
                   /note="NAD (By similarity)."
```

Mascot: http://www.matrixscience.com/

Spot 12

Mascot Search Results

Protein View

Match to: **gi|189204622** Score: **156** Expect: **2.5e-009**
fructose-bisphosphate aldolase [Pyrenophora tritici-repentis Pt-1C-BFP]

Nominal mass (M_r): **40217**; Calculated pI value: **5.47**
NCBI BLAST search of gi|189204622 against nr
Unformatted sequence string for pasting into other applications

Taxonomy: Pyrenophora tritici-repentis Pt-1C-BFP
Links to retrieve other entries containing this sequence from NCBI Entrez:
gi|187985745 from Pyrenophora tritici-repentis Pt-1C-BFP

Fixed modifications: Carbamidomethyl (C)
Variable modifications: Oxidation (M)
Cleavage by Trypsin: cuts C-term side of KR unless next residue is P
Sequence Coverage: **9%**

Matched peptides shown in **Bold Red**

```
  1 MVQASEVLSR KTGVIVGDDV YALFKHAQSE GYAIPAINVT SSSTVVASLE
 51 AARDSKSPIM LGGAAYFAGK GVDNKDQSAS IQGAIAGAHY IRAIAPAYGI
101 PVILHTDHCA KKLLPWLDGM MDADEAYFKE KGEPLFSSHM IDLSEEPKEW
151 NIATTKKYLQ RAAPMKQLIE MEIGITGGEE DGVNNESVDN NSLYTQPEDI
201 FEIYKELSAV SPLFSIAAGF GNDHFHTHST ALLYITDALS TVHGVYKPGN
251 HQQYVKDQTK SKDDKPVFLV FHGGSGSSVD EFRQAISYGV VKVNLDTDMQ
301 WAYLSGIRDY IQSKSGYLQT QVGNPDGEDK PNKKYYDPRV WVREGEKTMS
351 QRIKTALEDF HAAGKA
```

Show predicted peptides also

Sort Peptides By ◉ Residue Number ○ Increasing Mass ○ Decreasing Mass

Start - End	Observed	Mr(expt)	Mr(calc)	ppm	Miss	Sequence
93 - 111	2047.0778	2046.0705	2046.0721	-1	0	R.AIAPAYGIPVILHTDHCAK.K (Ions score 142)
93 - 111	2047.0778	2046.0705	2046.0721	-1	0	R.AIAPAYGIPVILHTDHCAK.K (No match)
93 - 112	2175.1726	2174.1653	2174.1670	-1	1	R.AIAPAYGIPVILHTDHCAKK.L (No match)
335 - 343	1253.6385	1252.6312	1252.6353	-3	1	K.YYDPRVWVR.E (No match)
340 - 347	1002.5409	1001.5336	1001.5294	4	1	R.VWVREGEK.T (No match)

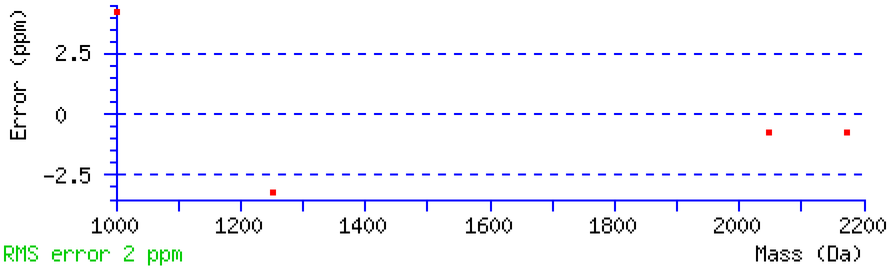

```
LOCUS       XP_001938646             366 aa            linear   PLN 30-MAY-2008
DEFINITION  fructose-bisphosphate aldolase [Pyrenophora tritici-repentis
            Pt-1C-BFP].
ACCESSION   XP_001938646
VERSION     XP_001938646.1  GI:189204622
DBSOURCE    REFSEQ: accession XM_001938611.1
KEYWORDS    .
SOURCE      Pyrenophora tritici-repentis Pt-1C-BFP
  ORGANISM  Pyrenophora tritici-repentis Pt-1C-BFP
            Eukaryota; Fungi; Dikarya; Ascomycota; Saccharomyceta;
            Pezizomycotina; Leotiomyceta; Dothideomyceta; Dothideomycetes;
            Pleosporomycetidae; Pleosporales; Pleosporineae; Pleosporaceae;
            Pyrenophora.
REFERENCE   1  (residues 1 to 366)
  AUTHORS   Birren,B., Lander,E., Galagan,J., Nusbaum,C., Devon,K., Ma,L.-J.,
            Jaffe,D., Butler,J., Alvarez,P., Gnerre,S., Grabherr,M., Kleber,M.,
            Mauceli,E., Brockman,W., MacCallum,I.A., Young,S., LaButti,K.,
            DeCaprio,D., Crawford,M., Koehrsen,M., Engels,R., Montgomery,P.,
            Pearson,M., Howarth,C., Larson,L., White,J., Yandava,C., Kodira,C.,
            Guigo,R., Borodovsky,M., Zeng,Q., O'Leary,S., Alvarado,L.,
            Pandelova,I. and Ciuffetti,L.
  CONSRTM   The Broad Institute Genome Sequencing Platform
  TITLE     Genome Sequence of Pyrenophora tritici-repentis
  JOURNAL   Unpublished
REFERENCE   2  (residues 1 to 366)
  AUTHORS   Birren,B., Lander,E., Galagan,J., Nusbaum,C., Devon,K., Ma,L.-J.,
            Jaffe,D., Butler,J., Alvarez,P., Gnerre,S., Grabherr,M., Kleber,M.,
            Mauceli,E., Brockman,W., MacCallum,I.A., Young,S., LaButti,K.,
            DeCaprio,D., Crawford,M., Koehrsen,M., Engels,R., Montgomery,P.,
            Pearson,M., Howarth,C., Larson,L., White,J., Yandava,C., Kodira,C.,
            Zeng,Q., O'Leary,S., Alvarado,L., Ciuffetti,L. and Pandelova,I.
  CONSRTM   The Broad Institute Genome Sequencing Platform
  TITLE     Direct Submission
  JOURNAL   Submitted (16-MAR-2007) Broad Institute of MIT and Harvard, 7
            Cambridge Center, Cambridge, MA 02142, USA
```

```
COMMENT     PROVISIONAL REFSEQ: This record has not yet been subject to final
            NCBI review. The reference sequence was derived from EDU51233.
            Method: conceptual translation.
FEATURES             Location/Qualifiers
     source          1..366
                     /organism="Pyrenophora tritici-repentis Pt-1C-BFP"
                     /strain="Pt-1C-BFP"
                     /db_xref="taxon:426418"
     Protein         1..366
                     /product="fructose-bisphosphate aldolase"
                     /calculated_mol_wt=40054
     Region          17..365
                     /region_name="FBP_aldolase_IIA"
                     /note="Class II Type A, Fructose-1,6-bisphosphate (FBP)
                     aldolases. The enzyme catalyses the zinc-dependent,
                     reversible aldol condensation of dihydroxyacetone
                     phosphate with glyceraldehyde-3-phosphate to form
                     fructose-1,6-bisphosphate. FBP aldolase is...; cd00946"
                     /db_xref="CDD:29572"
     Region          23..366
                     /region_name="gatY"
                     /note="tagatose-bisphosphate aldolase; Reviewed; PRK09195"
                     /db_xref="CDD:169707"
     Site            order(38,107..108,142,172,174,177..180,242..244,246,
                     272..273,275,294,296..297)
                     /site_type="active"
                     /db_xref="CDD:29572"
     Site            order(41..43,46,63,66..68,86,90,93..94,180,297..298,
                     300..301,304,308,311)
                     /site_type="other"
                     /note="intersubunit interface"
                     /db_xref="CDD:29572"
     Site            order(108,142,172,179,243,272)
                     /site_type="other"
                     /note="zinc binding site"
                     /db_xref="CDD:29572"
     Site            order(242,244,246,273,275)
                     /site_type="other"
                     /note="Na+ binding site"
                     /db_xref="CDD:29572"
     CDS             1..366
                     /locus_tag="PTRG_08314"
                     /coded_by="XM_001938611.1:1..1101"
                     /db_xref="GeneID:6346595"
```

Mascot: http://www.matrixscience.com/

Spot 13

Mascot Search Results

Protein View

Match to: **gi|189197705** Score: **98** Expect: **0.0017**
glycolipid transfer protein HET-C2 [Pyrenophora tritici-repentis Pt-1C-BFP]

Nominal mass (M_r): **24033**; Calculated pI value: **5.78**
NCBI BLAST search of gi|189197705 against nr
Unformatted sequence string for pasting into other applications

Taxonomy: Pyrenophora tritici-repentis Pt-1C-BFP
Links to retrieve other entries containing this sequence from NCBI Entrez:
gi|187981138 from Pyrenophora tritici-repentis Pt-1C-BFP

Fixed modifications: Carbamidomethyl (C)
Variable modifications: Oxidation (M)
Cleavage by Trypsin: cuts C-term side of KR unless next residue is P
Sequence Coverage: **25%**

Matched peptides shown in **Bold Red**

```
  1 MTAEIPPGGT FFDTLKKSFV DVPIDESKDS AIPTTEFLEA AESLLTLFDV
 51 LGSAAFKPVK SDMSGNIKKI RDRQLEAPAQ SETLQDLVLN ELAEKKHTAT
101 EGLVWLNRGL DFTAQALRHN ISNNEKELAD SFRDAYGNTL KPHHSFIVKP
151 IFSAAMSATP YRRDFYNKLG QDDAKVQAEL VKWLSALEKD VAVLNTFLAK
201 KEAKWVDWMG RHCF
```

Show predicted peptides also

Sort Peptides By ◉ Residue Number ○ Increasing Mass ○ Decreasing Mass

Start - End	Observed	Mr(expt)	Mr(calc)	ppm	Miss	Sequence
18 - 28	1235.5391	1234.5318	1234.6081	-62	0	K.SFVDVPIDESK.D (No match)
74 - 95	2439.2832	2438.2759	2438.2540	9	0	R.QLEAPAQSETLQDLVLNELAEK.K (No match)
74 - 96	2567.3911	2566.3838	2566.3490	14	1	R.QLEAPAQSETLQDLVLNELAEKK.H (No match)
109 - 118	1091.5867	1090.5794	1090.5771	2	0	R.GLDFTAQALR.H (Ions score 65)
109 - 118	1091.5867	1090.5794	1090.5771	2	0	R.GLDFTAQALR.H (No match)
202 - 211	1277.6161	1276.6088	1276.6023	5	1	K.EAKWVDWMGR.H (No match)
202 - 211	1277.6161	1276.6088	1276.6023	5	1	K.EAKWVDWMGR.H (No match)

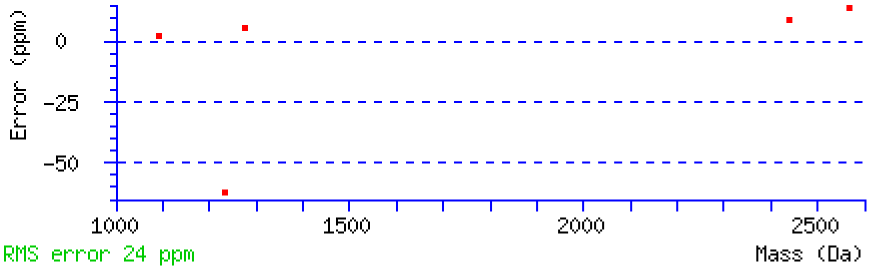


```
LOCUS       XP_001935190             214 aa            linear   PLN 30-MAY-2008
DEFINITION  glycolipid transfer protein HET-C2 [Pyrenophora tritici-repentis
            Pt-1C-BFP].
ACCESSION   XP_001935190
VERSION     XP_001935190.1  GI:189197705
DBSOURCE    REFSEQ: accession XM_001935155.1
KEYWORDS    .
SOURCE      Pyrenophora tritici-repentis Pt-1C-BFP
  ORGANISM  Pyrenophora tritici-repentis Pt-1C-BFP
            Eukaryota; Fungi; Dikarya; Ascomycota; Saccharomyceta;
            Pezizomycotina; Leotiomyceta; Dothideomyceta; Dothideomycetes;
            Pleosporomycetidae; Pleosporales; Pleosporineae; Pleosporaceae;
            Pyrenophora.
REFERENCE   1  (residues 1 to 214)
  AUTHORS   Birren,B., Lander,E., Galagan,J., Nusbaum,C., Devon,K., Ma,L.-J.,
            Jaffe,D., Butler,J., Alvarez,P., Gnerre,S., Grabherr,M., Kleber,M.,
            Mauceli,E., Brockman,W., MacCallum,I.A., Young,S., LaButti,K.,
            DeCaprio,D., Crawford,M., Koehrsen,M., Engels,R., Montgomery,P.,
            Pearson,M., Howarth,C., Larson,L., White,J., Yandava,C., Kodira,C.,
            Guigo,R., Borodovsky,M., Zeng,Q., O'Leary,S., Alvarado,L.,
            Pandelova,I. and Ciuffetti,L.
  CONSRTM   The Broad Institute Genome Sequencing Platform
  TITLE     Genome Sequence of Pyrenophora tritici-repentis
  JOURNAL   Unpublished
REFERENCE   2  (residues 1 to 214)
  AUTHORS   Birren,B., Lander,E., Galagan,J., Nusbaum,C., Devon,K., Ma,L.-J.,
            Jaffe,D., Butler,J., Alvarez,P., Gnerre,S., Grabherr,M., Kleber,M.,
            Mauceli,E., Brockman,W., MacCallum,I.A., Young,S., LaButti,K.,
            DeCaprio,D., Crawford,M., Koehrsen,M., Engels,R., Montgomery,P.,
            Pearson,M., Howarth,C., Larson,L., White,J., Yandava,C., Kodira,C.,
            Zeng,Q., O'Leary,S., Alvarado,L., Ciuffetti,L. and Pandelova,I.
  CONSRTM   The Broad Institute Genome Sequencing Platform
  TITLE     Direct Submission
  JOURNAL   Submitted (16-MAR-2007) Broad Institute of MIT and Harvard, 7
            Cambridge Center, Cambridge, MA 02142, USA
```

```
COMMENT     PROVISIONAL REFSEQ: This record has not yet been subject to final
            NCBI review. The reference sequence was derived from EDU47764.
            Method: conceptual translation.
FEATURES             Location/Qualifiers
     source          1..214
                     /organism="Pyrenophora tritici-repentis Pt-1C-BFP"
                     /strain="Pt-1C-BFP"
                     /db_xref="taxon:426418"
     Protein         1..214
                     /product="glycolipid transfer protein HET-C2"
                     /calculated_mol_wt=23860
     Region          31..173
                     /region_name="GLTP"
                     /note="Glycolipid transfer protein (GLTP); pfam08718"
                     /db_xref="CDD:149696"
     CDS             1..214
                     /locus_tag="PTRG_04857"
                     /coded_by="XM_001935155.1:73..717"
                     /db_xref="GeneID:6343099"
```

Mascot: http://www.matrixscience.com/

Spot 14

{MATRIX SCIENCE} Mascot Search Results

Protein View

Match to: **gi|126139808** Score: **124** Expect: **4e-006**
hypothetical protein PICST_50942 [Pichia stipitis CBS 6054]

Nominal mass (M_r): **38084**; Calculated pI value: **5.44**
NCBI BLAST search of gi|126139808 against nr
Unformatted sequence string for pasting into other applications

Taxonomy: Pichia stipitis CBS 6054
Links to retrieve other entries containing this sequence from NCBI Entrez:
gi|126093710 from Pichia stipitis CBS 6054

Fixed modifications: Carbamidomethyl (C)
Variable modifications: Oxidation (M)
Cleavage by Trypsin: cuts C-term side of KR unless next residue is P
Sequence Coverage: **20%**

Matched peptides shown in **Bold Red**

```
  1 MAPPTRIETT TSVVEVNLAK VSKKSIKLES QADNAEVTFA DWENFKFAPI
 51 RESTVSRAMT KRYFADLDKY TESDVVIVGA GSAGLSAAYV LAKNRPNLKI
101 AIIEASVSPG GGCWLGGQLF SAMVLRKPAH LFLDELEIQY DDEGDYVVVK
151 HAALFMSTLL SKVLQFPNVK LFNATAVEDL ITRRDENTGE LRIAGVVTNW
201 TLVALNHDTQ SCMDPNTINC NIVLSTTGHD GPFGAFSAKR LEELGKAPKD
251 ITQGFRPQER AQPVAASADG FQLGGMRGLD MNKAEDAIVK GTREVVPGLV
301 IAGMELAEVD GSNRMGPTFG AMALSGVKAA ESVLNAFDLR KKQNETCYGA
351 Q
```

Show predicted peptides also

Sort Peptides By ◉ Residue Number ○ Increasing Mass ○ Decreasing Mass

Start - End	Observed	Mr(expt)	Mr(calc)	ppm	Miss	Sequence
70 - 93	2341.2615	2340.2542	2340.2213	14	0	K.YTESDVVIVGAGSAGLSAAYVLAK.N (No match)
70 - 93	2341.2615	2340.2542	2340.2213	14	0	K.YTESDVVIVGAGSAGLSAAYVLAK.N (No match)
151 - 162	1318.6838	1317.6765	1317.7115	-27	0	K.HAALFMSTLLSK.V (No match)
151 - 162	1334.6796	1333.6723	1333.7064	-26	0	K.HAALFMSTLLSK.V Oxidation (M) (No match)
163 - 170	944.5566	943.5493	943.5491	0	0	K.VLQFPNVK.L (No match)
315 - 328	1366.6854	1365.6781	1365.6785	-0	0	R.MGPTFGAMALSGVK.A (Ions score 86)
315 - 328	1366.6854	1365.6781	1365.6785	-0	0	R.MGPTFGAMALSGVK.A (No match)
315 - 328	1382.6804	1381.6731	1381.6734	-0	0	R.MGPTFGAMALSGVK.A Oxidation (M) (Ions score 49)

315 - 328	1382.6804	1381.6731	1381.6734	-0	0	R.MGPTFGAMALSGVK.A	Oxidation (M) (No match)
315 - 328	1398.6754	1397.6681	1397.6683	-0	0	R.MGPTFGAMALSGVK.A	2 Oxidation (M) (No match)
329 - 341	1433.7393	1432.7320	1432.7674	-25	1	K.AAESVLNAFDLRK.K	(Ions score 14)
329 - 341	1433.7393	1432.7320	1432.7674	-25	1	K.AAESVLNAFDLRK.K	(No match)

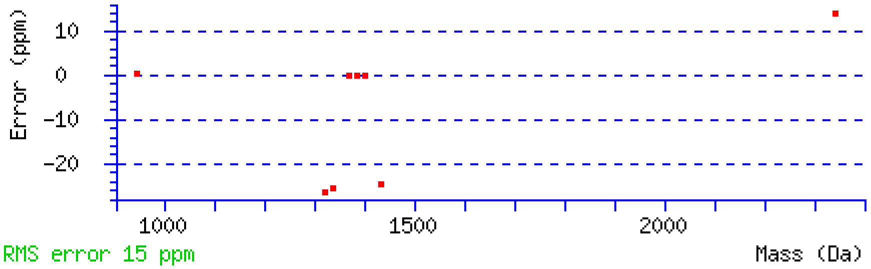


```
LOCUS       XP_001386426             351 aa            linear   PLN 06-JUL-2010
DEFINITION  hypothetical protein PICST_50942 [Scheffersomyces stipitis CBS
            6054].
ACCESSION   XP_001386426
VERSION     XP_001386426.1  GI:126139808
DBSOURCE    REFSEQ: accession XM_001386389.1
KEYWORDS    .
SOURCE      Scheffersomyces stipitis CBS 6054 (Pichia stipitis CBS 6054)
  ORGANISM  Scheffersomyces stipitis CBS 6054
            Eukaryota; Fungi; Dikarya; Ascomycota; Saccharomyceta;
            Saccharomycotina; Saccharomycetes; Saccharomycetales;
            Debaryomycetaceae; Scheffersomyces.
REFERENCE   1  (residues 1 to 351)
  AUTHORS   Jeffries,T.W., Grigoriev,I.V., Grimwood,J., Laplaza,J.M., Aerts,A.,
            Salamov,A., Schmutz,J., Lindquist,E., Dehal,P., Shapiro,H.,
            Jin,Y.S., Passoth,V. and Richardson,P.M.
  TITLE     Genome sequence of the lignocellulose-bioconverting and
            xylose-fermenting yeast Pichia stipitis
  JOURNAL   Nat. Biotechnol. 25 (3), 319-326 (2007)
   PUBMED   17334359
REFERENCE   2  (residues 1 to 351)
  AUTHORS   Grigoriev,I., Grimwood,J., Aerts,A., Salamov,A., Zhou,K., Lou,Y.,
            Pitluck,S., Schmutz,J., Myers,R.M., Lucas,S., Detter,J.C., Glavina
            del Rio,T., Jin,Y.-S., Passoth,V., Laplaza,J.M., Jeffries,T.W. and
            Richardson,P.
  CONSRTM   US DOE Joint Genome Institute
  TITLE     Direct Submission
  JOURNAL   Submitted (12-DEC-2006) US DOE Joint Genome Institute, 2800
            Mitchell Drive B100, Walnut Creek, CA 94598-1698, USA
COMMENT     PROVISIONAL REFSEQ: This record has not yet been subject to final
            NCBI review. The reference sequence was derived from ABN68397.
```

```
            Method: conceptual translation.
FEATURES             Location/Qualifiers
     source          1..351
                     /organism="Scheffersomyces stipitis CBS 6054"
                     /strain="CBS 6054"
                     /db_xref="taxon:322104"
                     /chromosome="8"
     Protein         1..351
                     /product="hypothetical protein"
                     /calculated_mol_wt=37749
     Region          46..342
                     /region_name="NADB_Rossmann"
                     /note="Rossmann-fold NAD(P)(+)-binding proteins; cl09931"
                     /db_xref="CDD:176428"
     Region          56..319
                     /region_name="Thi4"
                     /note="Thi4 family; pfam01946"
                     /db_xref="CDD:145231"
     CDS             1..351
                     /locus_tag="PICST_50942"
                     /coded_by="XM_001386389.1:1..1056"
                     /transl_table=12
                     /db_xref="InterPro:IPR002922"
                     /db_xref="GeneID:4840840"
```

Mascot: http://www.matrixscience.com/

Spot 15

MATRIX SCIENCE Mascot Search Results

Protein View

Match to: **gi|212542889** Score: **306** Expect: **2.5e-024**
glycerol dehydrogenase Gcy1, putative [Penicillium marneffei ATCC 18224]

Nominal mass (M_r): **33667**; Calculated pI value: **6.02**
NCBI BLAST search of gi|212542889 against nr
Unformatted sequence string for pasting into other applications

Taxonomy: Penicillium marneffei ATCC 18224
Links to retrieve other entries containing this sequence from NCBI Entrez:
gi|210066506 from Penicillium marneffei ATCC 18224

Fixed modifications: Carbamidomethyl (C)
Variable modifications: Oxidation (M)
Cleavage by Trypsin: cuts C-term side of KR unless next residue is P
Sequence Coverage: **17%**

Matched peptides shown in **Bold Red**

```
  1 MSALEHTKKT FTLNTGDKIP AVGLGTWQSK PNEVREAVKT ALLAGYRHID
 51 TALAYGNEHE VGQGIKDSGI PREQIWITTK LDNAWHHRVQ DGINSSLSSL
101 GVDYVDLYLV HWPSSTDPND DMKHLPDWDF IKTWEEIQKL PATGKVRNIG
151 VSNFGIKNLE RLLNAPTTKI VPAVNQIELH PNNPSPKLVE YNVSKGIHCT
201 GYSCLGSTNS PLYKDSTLLK MAEKKGKTPQ QLLLAWGVQK GWSVIPKSVS
251 KERIEKNFQL DEWELSAEEL EELSSLKDRF KVCGDDWLPV RVFFGDDE
```

Show predicted peptides also

Sort Peptides By ◉ Residue Number ○ Increasing Mass ○ Decreasing Mass

Start - End	Observed	Mr(expt)	Mr(calc)	ppm	Miss	Sequence
19 - 35	1852.0052	1850.9979	1851.0003	-1	0	K.IPAVGLGTWQSKPNEVR.E (Ions score 155)
19 - 35	1852.0052	1850.9979	1851.0003	-1	0	K.IPAVGLGTWQSKPNEVR.E (No match)
19 - 39	2279.2395	2278.2322	2278.2433	-5	1	K.IPAVGLGTWQSKPNEVREAVK.T (No match)
158 - 169	1369.8114	1368.8041	1368.7725	23	1	K.NLERLLNAPTTK.I (No match)
170 - 187	1967.0675	1966.0602	1966.0636	-2	0	K.IVPAVNQIELHPNNPSPK.L (Ions score 131)
170 - 187	1967.0675	1966.0602	1966.0636	-2	0	K.IVPAVNQIELHPNNPSPK.L (No match)

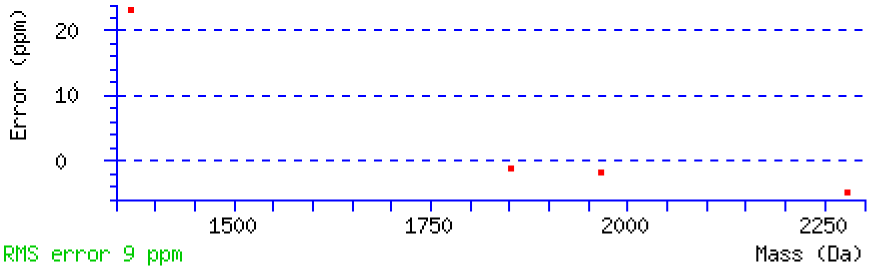


```
LOCUS       XP_002151599             298 aa            linear   PLN 13-NOV-2008
DEFINITION  glycerol dehydrogenase Gcy1, putative [Penicillium marneffei ATCC
            18224].
ACCESSION   XP_002151599
VERSION     XP_002151599.1  GI:212542889
DBSOURCE    REFSEQ: accession XM_002151563.1
KEYWORDS    .
SOURCE      Penicillium marneffei ATCC 18224
  ORGANISM  Penicillium marneffei ATCC 18224
            Eukaryota; Fungi; Dikarya; Ascomycota; Saccharomyceta;
            Pezizomycotina; Leotiomyceta; Eurotiomycetes; Eurotiomycetidae;
            Eurotiales; Trichocomaceae; mitosporic Trichocomaceae; Penicillium.
REFERENCE   1  (residues 1 to 298)
  AUTHORS   Fedorova,N.D., Joardar,V., Maiti,R., Schobel,S., Amedeo,P.,
            Galens,K., Inman,J.M., White,O.R., Whitty,B.R., Wortman,J.R. and
            Nierman,W.
  TITLE     Direct Submission
  JOURNAL   Submitted (03-OCT-2007) J. Craig Ventor Institute, 9704 Medical
            Center Drive, Rockville, MD 20850, USA
COMMENT     PROVISIONAL REFSEQ: This record has not yet been subject to final
            NCBI review. The reference sequence was derived from
            mrna.PMAA_044320A.
            Method: conceptual translation.
FEATURES             Location/Qualifiers
     source          1..298
                     /organism="Penicillium marneffei ATCC 18224"
                     /strain="ATCC 18224"
                     /culture_collection="ATCC:18224"
                     /db_xref="taxon:441960"
     Protein         1..298
                     /product="glycerol dehydrogenase Gcy1, putative"
                     /calculated_mol_wt=33386
     Region          10..259
                     /region_name="Aldo_ket_red"
                     /note="Aldo-keto reductases (AKRs) are a superfamily of
```

```
                     soluble NAD(P)(H) oxidoreductases whose chief purpose is
                     to reduce aldehydes and ketones to primary and secondary
                     alcohols. AKRs are present in all phyla and are of
                     importance to both health and...; cd06660"
                     /db_xref="CDD:119408"
     Site            order(25..27,50,55,80,111..112,152..153,176,202..207,230,
                     245..248,253,256..257)
                     /site_type="active"
                     /db_xref="CDD:119408"
     Site            order(50,55,80,111)
                     /site_type="other"
                     /note="catalytic tetrad"
                     /db_xref="CDD:119408"
     CDS             1..298
                     /locus_tag="PMAA_044320"
                     /coded_by="XM_002151563.1:417..1313"
                     /note="encoded by transcript PMAA_044320A"
                     /db_xref="GeneID:7029053"
```

Mascot: http://www.matrixscience.com/

Spot 16

MATRIX SCIENCE Mascot Search Results

Protein View

Match to: **gi|67903236** Score: **214** Expect: **4e-015**
hypothetical protein AN8605.2 [Aspergillus nidulans FGSC A4]

Nominal mass (M_r): **17787**; Calculated pI value: **8.86**
NCBI BLAST search of gi|67903236 against nr
Unformatted sequence string for pasting into other applications

Taxonomy: Aspergillus nidulans FGSC A4
Links to retrieve other entries containing this sequence from NCBI Entrez:
gi|40741449 from Aspergillus nidulans FGSC A4
gi|259483180 from Aspergillus nidulans FGSC A4

Fixed modifications: Carbamidomethyl (C)
Variable modifications: Oxidation (M)
Cleavage by Trypsin: cuts C-term side of KR unless next residue is P
Sequence Coverage: **50%**

Matched peptides shown in **Bold Red**

```
  1 MSNVFFDITA NGEPLGRVEF KLFDDVVPKT ARNFRELATG QHGFGYKGSP
 51 FHRVIPQFML QGGDFTRQNG TGGKSIYGEK FEDENFTLKH DRPYLLSMAN
101 AGRNTNGSQF FITTVKTSWL DGAHVVFGEV VKGQEVVDAV EKLGSQSGAT
151 KKKVVISNSG TL
```

Show predicted peptides also

Sort Peptides By ◉ Residue Number ○ Increasing Mass ○ Decreasing Mass

Start - End	Observed	Mr(expt)	Mr(calc)	ppm	Miss	Sequence
1 - 21	2371.0574	2370.0501	2370.1678	-50	1	-.MSNVFFDITANGEPLGRVEFK.L (No match)
18 - 29	1435.7458	1434.7385	1434.7759	-26	1	R.VEFKLFDDVVPK.T (Ions score 59)
18 - 29	1435.7458	1434.7385	1434.7759	-26	1	R.VEFKLFDDVVPK.T (No match)
36 - 47	1307.6772	1306.6699	1306.6306	30	0	R.ELATGQHGFGYK.G (No match)
54 - 67	1608.8229	1607.8156	1607.8130	2	0	R.VIPQFMLQGGDFTR.Q (Ions score 107)
54 - 67	1608.8229	1607.8156	1607.8130	2	0	R.VIPQFMLQGGDFTR.Q (No match)
54 - 67	1624.8175	1623.8102	1623.8079	1	0	R.VIPQFMLQGGDFTR.Q Oxidation (M) (Ions score 65)
54 - 67	1624.8175	1623.8102	1623.8079	1	0	R.VIPQFMLQGGDFTR.Q Oxidation (M) (No match)
68 - 80	1338.6637	1337.6564	1337.6575	-1	1	R.QNGTGGKSIYGEK.F (No match)
90 - 103	1600.7590	1599.7517	1599.7940	-26	0	K.HDRPYLLSMANAGR.N (No match)

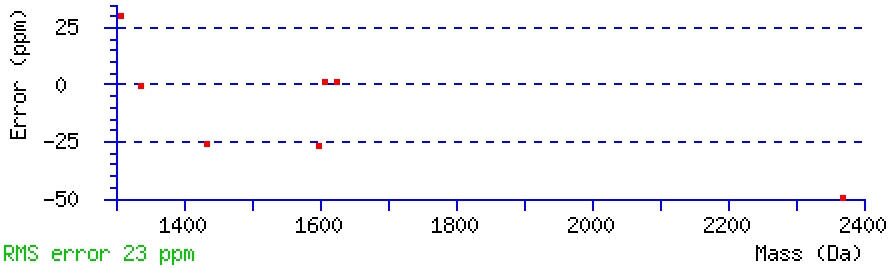


```
LOCUS       XP_681874                162 aa            linear   PLN 09-APR-2008
DEFINITION  hypothetical protein AN8605.2 [Aspergillus nidulans FGSC A4].
ACCESSION   XP_681874
VERSION     XP_681874.1  GI:67903236
DBSOURCE    REFSEQ: accession XM_676782.1
KEYWORDS    .
SOURCE      Aspergillus nidulans FGSC A4
  ORGANISM  Aspergillus nidulans FGSC A4
            Eukaryota; Fungi; Dikarya; Ascomycota; Saccharomyceta;
            Pezizomycotina; Leotiomyceta; Eurotiomycetes; Eurotiomycetidae;
            Eurotiales; Trichocomaceae; Emericella.
REFERENCE   1  (residues 1 to 162)
  AUTHORS   Galagan,J.E., Calvo,S.E., Cuomo,C., Ma,L.J., Wortman,J.R.,
            Batzoglou,S., Lee,S.I., Basturkmen,M., Spevak,C.C., Clutterbuck,J.,
            Kapitonov,V., Jurka,J., Scazzocchio,C., Farman,M., Butler,J.,
            Purcell,S., Harris,S., Braus,G.H., Draht,O., Busch,S., D'Enfert,C.,
            Bouchier,C., Goldman,G.H., Bell-Pedersen,D., Griffiths-Jones,S.,
            Doonan,J.H., Yu,J., Vienken,K., Pain,A., Freitag,M., Selker,E.U.,
            Archer,D.B., Penalva,M.A., Oakley,B.R., Momany,M., Tanaka,T.,
            Kumagai,T., Asai,K., Machida,M., Nierman,W.C., Denning,D.W.,
            Caddick,M., Hynes,M., Paoletti,M., Fischer,R., Miller,B., Dyer,P.,
            Sachs,M.S., Osmani,S.A. and Birren,B.W.
  TITLE     Sequencing of Aspergillus nidulans and comparative analysis with A.
            fumigatus and A. oryzae
  JOURNAL   Nature 438 (7071), 1105-1115 (2005)
   PUBMED   16372000
REFERENCE   2  (residues 1 to 162)
  AUTHORS   Birren,B., Nusbaum,C., Abebe,A., Abouelleil,A., Adekoya,E.,
            Ait-zahra,M., Allen,N., Allen,T., An,P., Anderson,M., Anderson,S.,
            Arachchi,H., Armbruster,J., Bachantsang,P., Baldwin,J., Barry,A.,
            Bayul,T., Blitshsteyn,B., Bloom,T., Blye,J., Boguslavskiy,L.,
            Borowsky,M., Boukhgalter,B., Brunache,A., Butler,J., Calixte,N.,
            Calvo,S., Camarata,J., Campo,K., Chang,J., Cheshatsang,Y.,
            Citroen,M., Collymore,A., Considine,T., Cook,A., Cooke,P.,
            Corum,B., Cuomo,C., David,R., Dawoe,T., Degray,S., Dodge,S.,
```

```
            Dooley,K., Dorje,P., Dorjee,K., Dorris,L., Duffey,N., Dupes,A.,
            Elkins,T., Engels,R., Erickson,J., Farina,A., Faro,S., Ferreira,P.,
            Fischer,H., Fitzgerald,M., Foley,K., Gage,D., Galagan,J.,
            Gearin,G., Gnerre,S., Gnirke,A., Goyette,A., Graham,J.,
            Grandbois,E., Gyaltsen,K., Hafez,N., Hagopian,D., Hagos,B.,
            Hall,J., Hatcher,B., Heller,A., Higgins,H., Honan,T., Horn,A.,
            Houde,N., Hughes,L., Hulme,W., Husby,E., Iliev,I., Jaffe,D.,
            Jones,C., Kamal,M., Kamat,A., Kamvysselis,M., Karlsson,E.,
            Kells,C., Kieu,A., Kisner,P., Kodira,C., Kulbokas,E., Labutti,K.,
            Lama,D., Landers,T., Leger,J., Levine,S., Lewis,D., Lewis,T.,
            Lindblad-toh,K., Liu,X., Lokyitsang,T., Lokyitsang,Y., Lucien,O.,
            Lui,A., Ma,L.J., Mabbitt,R., Macdonald,J., Maclean,C., Major,J.,
            Manning,J., Marabella,R., Maru,K., Matthews,C., Mauceli,E.,
            Mccarthy,M., Mcdonough,S., Mcghee,T., Meldrim,J., Meneus,L.,
            Mesirov,J., Mihalev,A., Mihova,T., Mikkelsen,T., Mlenga,V.,
            Moru,K., Mozes,J., Mulrain,L., Munson,G., Naylor,J., Newes,C.,
            Nguyen,C., Nguyen,N., Nguyen,T., Nicol,R., Nielsen,C., Nizzari,M.,
            Norbu,C., Norbu,N., O'donnell,P., Okoawo,O., O'leary,S.,
            Omotosho,B., O'neill,K., Osman,S., Parker,S., Perrin,D.,
            Phunkhang,P., Piqani,B., Purcell,S., Rachupka,T., Ramasamy,U.,
            Rameau,R., Ray,V., Raymond,C., Retta,R., Richardson,S., Rise,C.,
            Rodriguez,J., Rogers,J., Rogov,P., Rutman,M., Schupbach,R.,
            Seaman,C., Settipalli,S., Sharpe,T., Sheridan,J., Sherpa,N.,
            Shi,J., Smirnov,S., Smith,C., Sougnez,C., Spencer,B., Stalker,J.,
            Stange-thomann,N., Stavropoulos,S., Stetson,K., Stone,C., Stone,S.,
            Stubbs,M., Talamas,J., Tchuinga,P., Tenzing,P., Tesfaye,S.,
            Theodore,J., Thoulutsang,Y., Topham,K., Towey,S., Tsamla,T.,
            Tsomo,N., Vallee,D., Vassiliev,H., Venkataraman,V., Vinson,J.,
            Vo,A., Wade,C., Wang,S., Wangchuk,T., Wangdi,T., Whittaker,C.,
            Wilkinson,J., Wu,Y., Wyman,D., Yadav,S., Yang,S., Yang,X.,
            Yeager,S., Yee,E., Young,G., Zainoun,J., Zembeck,L., Zimmer,A.,
            Zody,M. and Lander,E.
  TITLE     Direct Submission
  JOURNAL   Submitted (26-APR-2004) Whitehead Institute/MIT Center for Genome
            Research, 320 Charles Street, Cambridge, MA 02142, USA
COMMENT     PROVISIONAL REFSEQ: This record has not yet been subject to final
            NCBI review. The reference sequence was derived from EAA60639.
            Method: conceptual translation.
FEATURES             Location/Qualifiers
     source          1..162
                     /organism="Aspergillus nidulans FGSC A4"
                     /strain="FGSC A4"
                     /db_xref="taxon:227321"
                     /chromosome="III"
     Protein         1..162
                     /product="hypothetical protein"
                     /calculated_mol_wt=17667
     Region          2..160
                     /region_name="cyclophilin_ABH_like"
```

```
                     /note="cyclophilin_ABH_like: Cyclophilin  A, B and H-like
                     cyclophilin-type peptidylprolyl cis- trans isomerase
                     (PPIase) domain. This family represents the archetypal
                     cystolic cyclophilin similar to human cyclophilins A, B
                     and H. PPIase is an enzyme which...; cd01926"
                     /db_xref="CDD:29397"
     Site            order(52..53,58,109,111,119)
                     /site_type="active"
                     /db_xref="CDD:29397"
     CDS             1..162
                     /locus_tag="AN8605.2"
                     /coded_by="XM_676782.1:1..489"
                     /db_xref="GeneID:2868486"
```

Mascot: http://www.matrixscience.com/

Spot 17

{MATRIX SCIENCE} Mascot Search Results

Protein View

Match to: **gi|70985178** Score: **325** Expect: **3.2e-026**
thiamine biosynthesis protein (Nmt1) [Aspergillus fumigatus Af293]

Nominal mass (M_r): **38583**; Calculated pI value: **6.04**
NCBI BLAST search of gi|70985178 against nr
Unformatted sequence string for pasting into other applications

Taxonomy: Aspergillus fumigatus Af293
Links to retrieve other entries containing this sequence from NCBI Entrez:
gi|55743873 from Aspergillus fumigatus
gi|66845723 from Aspergillus fumigatus Af293
gi|159125982 from Aspergillus fumigatus A1163

Fixed modifications: Carbamidomethyl (C)
Variable modifications: Oxidation (M)
Cleavage by Trypsin: cuts C-term side of KR unless next residue is P
Sequence Coverage: **25%**

Matched peptides shown in **Bold Red**

```
  1 MSTDKITFLT NWHATPYHAP LYLAHSKGFF KEEGLKVAIL EPNDPSDVTE
 51 IIGSGKVDMG FKAMIHTLAA KARNFPVTSI GSLLDEPFTG VVYLKDSGIT
101 EDFRSLKGKR IGYVGEFGKI QIDELTKYYG MTADDYTAVR CGMNVTKAII
151 RGDIDAGIGL ENVQMVELAE WLASQNRPRD DVQMLRIDQL AELGCCCFCS
201 ILYIANDAFL AANPDKVQKF MRAVKRATDY VLAEPAKAYE EYIDVKPIMG
251 TPVNRKIFER SFAYFSRDLK NVQRDWAKVT NYGKRLGILD ANFEPNYTNK
301 YLSWDLDADS TDPLGDQKRM AELQKQVAAE GGFKRLHVSA SA
```

Show predicted peptides also

Sort Peptides By ◉ Residue Number ○ Increasing Mass ○ Decreasing Mass

Start - End	Observed	Mr(expt)	Mr(calc)	ppm	Miss	Sequence
28 - 36	1054.5637	1053.5564	1053.5495	7	1	K.GFFKEEGLK.V (Ions score 42)
28 - 36	1054.5637	1053.5564	1053.5495	7	1	K.GFFKEEGLK.V (No match)
37 - 56	2054.0669	2053.0596	2053.0579	1	0	K.VAILEPNDPSDVTEIIGSGK.V (Ions score 35)
37 - 56	2054.0669	2053.0596	2053.0579	1	0	K.VAILEPNDPSDVTEIIGSGK.V (No match)
74 - 95	2396.2756	2395.2683	2395.2675	0	0	R.NFPVTSIGSLLDEPFTGVVYLK.D (Ions score 27)
74 - 95	2396.2756	2395.2683	2395.2675	0	0	R.NFPVTSIGSLLDEPFTGVVYLK.D (No match)
110 - 119	1125.6115	1124.6042	1124.5978	6	1	K.RIGYVGEFGK.I (Ions score 23)

110 - 119	1125.6115	1124.6042	1124.5978	6	1	K.RIGYVGEFGK.I	(No match)
111 - 119	969.5135	968.5062	968.4967	10	0	R.IGYVGEFGK.I	(Ions score 70)
111 - 119	969.5135	968.5062	968.4967	10	0	R.IGYVGEFGK.I	(No match)
128 - 140	1525.6808	1524.6735	1524.6555	12	0	K.YYGMTADDYTAVR.C	(Ions score 30)
128 - 140	1525.6808	1524.6735	1524.6555	12	0	K.YYGMTADDYTAVR.C	(No match)
128 - 140	1541.6741	1540.6668	1540.6504	11	0	K.YYGMTADDYTAVR.C	Oxidation (M) (Ions score 34)
128 - 140	1541.6741	1540.6668	1540.6504	11	0	K.YYGMTADDYTAVR.C	Oxidation (M) (No match)
180 - 186	876.4326	875.4253	875.4171	9	0	R.DDVQMLR.I	(No match)
180 - 186	892.4239	891.4166	891.4120	5	0	R.DDVQMLR.I	Oxidation (M) (No match)
261 - 267	877.4264	876.4191	876.4130	7	0	R.SFAYFSR.D	(Ions score 52)
261 - 267	877.4264	876.4191	876.4130	7	0	R.SFAYFSR.D	(No match)

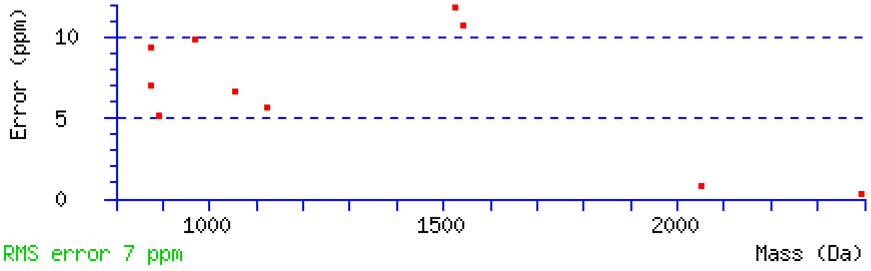

Mascot: http://www.matrixscience.com/

Spot 18

{MATRIX SCIENCE} Mascot Search Results

Protein View

Match to: **gi|255943905** Score: **471** Expect: **7.9e-041**
Pc20g01610 [Penicillium chrysogenum Wisconsin 54-1255]

Nominal mass (M_r): **35821**; Calculated pI value: **8.44**
NCBI BLAST search of gi|255943905 against nr
Unformatted sequence string for pasting into other applications

Taxonomy: Penicillium chrysogenum Wisconsin 54-1255
Links to retrieve other entries containing this sequence from NCBI Entrez:
gi|211587455 from Penicillium chrysogenum Wisconsin 54-1255

Fixed modifications: Carbamidomethyl (C)
Variable modifications: Oxidation (M)
Cleavage by Trypsin: cuts C-term side of KR unless next residue is P
Sequence Coverage: **26%**

Matched peptides shown in **Bold Red**

1 MFAARRTVNL FQKR**AFSASA INASK**VSVLG AAGGIGQPLS LLLKLNPR**VS**
51 **ELALYDIR**GG PGVAADLSHI NTNSTVTGYN PDASGLR**DCL EGSEIILIPA**
101 **GVPR**KPGMTR **DDLFNTNASI VR**DLAKAAAE AAPKAHVLVI ANPVNSTVPI
151 VAEVYKARNV YDPK**RLFGVT TLDVVR**ASRF ISQVQNTNPA GEAVPVVGGH
201 SGVTIVPLLS QSNHSSIAGQ ARDALVNRIQ FGGDEVVKAK DGAGSATLSM
251 AMAGAR**FAES LLRAAQGEKG VIEPTFVDSP LYK**DQGIDFF ASRVELGPNG
301 VEKINSVGEV NEYEQGLLDA CLTDLKKNIQ KGVDFVKANP

Show predicted peptides also

Sort Peptides By ◉ Residue Number ○ Increasing Mass ○ Decreasing Mass

Start - End	Observed	Mr(expt)	Mr(calc)	ppm	Miss	Sequence	
15 - 25	1066.6061	1065.5988	1065.5454	50	0	R.AFSASAINASK.V	(No match)
15 - 25	1066.6061	1065.5988	1065.5454	50	0	R.AFSASAINASK.V	(No match)
49 - 58	1178.6432	1177.6359	1177.6343	1	0	R.VSELALYDIR.G	(Ions score 78)
49 - 58	1178.6432	1177.6359	1177.6343	1	0	R.VSELALYDIR.G	(No match)
88 - 104	1838.9681	1837.9608	1837.9608	0	0	R.DCLEGSEIILIPAGVPR.K	(Ions score 48)
88 - 104	1838.9681	1837.9608	1837.9608	0	0	R.DCLEGSEIILIPAGVPR.K	(No match)
111 - 122	1364.6836	1363.6763	1363.6732	2	0	R.DDLFNTNASIVR.D	(Ions score 103)
111 - 122	1364.6836	1363.6763	1363.6732	2	0	R.DDLFNTNASIVR.D	(No match)
165 - 176	1375.8073	1374.8000	1374.7984	1	1	K.RLFGVTTLDVVR.A	(Ions score 81)

165 - 176	1375.8073	1374.8000	1374.7984	1	1	K.RLFGVTTLDVVR.A	(No match)
166 - 176	1219.7064	1218.6991	1218.6972	2	0	R.LFGVTTLDVVR.A	(Ions score 71)
166 - 176	1219.7064	1218.6991	1218.6972	2	0	R.LFGVTTLDVVR.A	(No match)
257 - 263	835.4628	834.4555	834.4599	-5	0	R.FAESLLR.A	(Ions score 37)
257 - 269	1419.7958	1418.7885	1418.7517	26	1	R.FAESLLRAAQGEK.G	(No match)
270 - 283	1564.8185	1563.8112	1563.8185	-5	0	K.GVIEPTFVDSPLYK.D	(No match)

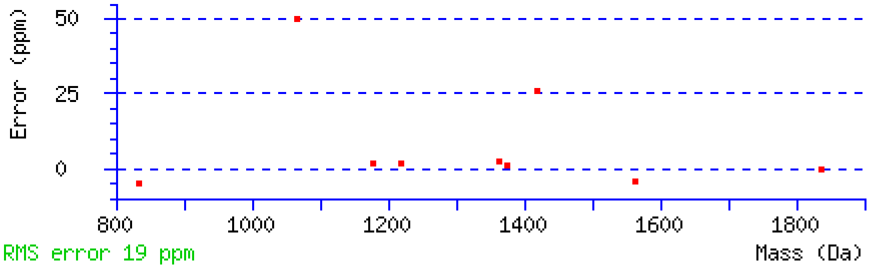

RMS error 19 ppm

```
LOCUS       XP_002562720             340 aa            linear   PLN 14-AUG-2009
DEFINITION  Pc20g01610 [Penicillium chrysogenum Wisconsin 54-1255].
ACCESSION   XP_002562720
VERSION     XP_002562720.1  GI:255943905
DBSOURCE    REFSEQ: accession XM_002562674.1
KEYWORDS    .
SOURCE      Penicillium chrysogenum Wisconsin 54-1255
  ORGANISM  Penicillium chrysogenum Wisconsin 54-1255
            Eukaryota; Fungi; Dikarya; Ascomycota; Saccharomyceta;
            Pezizomycotina; Leotiomyceta; Eurotiomycetes; Eurotiomycetidae;
            Eurotiales; Trichocomaceae; mitosporic Trichocomaceae; Penicillium;
            Penicillium chrysogenum complex.
REFERENCE   1  (residues 1 to 340)
  AUTHORS   van den Berg,M.A., Albang,R., Albermann,K., Badger,J.H.,
            Daran,J.M., Driessen,A.J., Garcia-Estrada,C., Fedorova,N.D.,
            Harris,D.M., Heijne,W.H., Joardar,V., Kiel,J.A., Kovalchuk,A.,
            Martin,J.F., Nierman,W.C., Nijland,J.G., Pronk,J.T., Roubos,J.A.,
            van der Klei,I.J., van Peij,N.N., Veenhuis,M., von Dohren,H.,
            Wagner,C., Wortman,J. and Bovenberg,R.A.
  TITLE     Genome sequencing and analysis of the filamentous fungus
            Penicillium chrysogenum
  JOURNAL   Nat. Biotechnol. 26 (10), 1161-1168 (2008)
   PUBMED   18820685
REFERENCE   2  (residues 1 to 340)
  AUTHORS   van den Berg,M.A.
  TITLE     Direct Submission
  JOURNAL   Submitted (22-NOV-2007) van den Berg M.A., DAI/INNO (624-0270), DSM
            Anti-Infectives, Alexander Fleminglaan 1, Delftn, 2613 AX,
            NETHERLANDS
```

```
COMMENT     PROVISIONAL REFSEQ: This record has not yet been subject to final
            NCBI review. The reference sequence is identical to CAP85490.
FEATURES             Location/Qualifiers
     source          1..340
                     /organism="Penicillium chrysogenum Wisconsin 54-1255"
                     /strain="Wisconsin 54-1255"
                     /db_xref="taxon:500485"
                     /clone="Pc00c20"
     Protein         1..340
                     /product="hypothetical protein"
                     /name="Pc20g01610"
                     /calculated_mol_wt=35598
     Region          25..337
                     /region_name="MDH_euk_gproteo"
                     /note="malate dehydrogenase, NAD-dependent; TIGR01772"
                     /db_xref="CDD:130833"
     Region          25..336
                     /region_name="MDH_glyoxysomal_mitochondrial"
                     /note="Glyoxysomal and mitochondrial malate
                     dehydrogenases; cd01337"
                     /db_xref="CDD:133422"
     Site            order(32..35,56,99,101..102,113,117,120,140,142,169,172,
                     200,246,250)
                     /site_type="other"
                     /note="NAD binding site"
                     /db_xref="CDD:133422"
     Site            order(37,65..66,68..70,175..176,179,232,235,239..240,
                     246..249,252)
                     /site_type="other"
                     /note="dimerization interface"
                     /db_xref="CDD:133422"
     Site            order(104,110,142,176,200,233)
                     /site_type="other"
                     /note="Substrate binding site"
                     /db_xref="CDD:133422"
     CDS             1..340
                     /locus_tag="Pc20g01610"
                     /coded_by="XM_002562674.1:1..1023"
                     /inference="protein motif:COGS:COG0039"
                     /inference="protein motif:PFAM:PF00056"
                     /inference="protein motif:PFAM:PF02866"
                     /inference="similar to AA sequence:PIR:DEBYMM"
                     /db_xref="GeneID:8312298"
```

Mascot: http://www.matrixscience.com/

Spot 19

{MATRIX SCIENCE} Mascot Search Results

Protein View

Match to: **gi|254583736** Score: **161** Expect: **7.9e-010**
ZYRO0F05522p [Zygosaccharomyces rouxii]

Nominal mass (M_r): **53989**; Calculated pI value: **8.64**
NCBI BLAST search of gi|254583736 against nr
Unformatted sequence string for pasting into other applications

Taxonomy: Zygosaccharomyces rouxii CBS 732
Links to retrieve other entries containing this sequence from NCBI Entrez:
gi|238940329 from Zygosaccharomyces rouxii

Fixed modifications: Carbamidomethyl (C)
Variable modifications: Oxidation (M)
Cleavage by Trypsin: cuts C-term side of KR unless next residue is P
Sequence Coverage: **9%**

Matched peptides shown in **Bold Red**

```
  1 MFSIRKRQTV VWVLLFSLVI YWKHLLMATT RPVVIVGTGL AGLSAGNQLV
 51 KHKIPVILLD KASSIGGNSI KASSGINGAW TETQKRLNVQ DSPELFLQDT
101 IRSAKGKGVE PLMEKLTTDA ASAIHWLQDE FKLKLDLLMQ IGGQSAPRTH
151 RSSGKLPPGF EIIQSMSKAL QAQAEKDPSL VKILLESKVV DVSVDGRGKI
201 SGVAYEDSKG NTQKIETDNV IFCSGGFSRS KEMLEEYVPQ YTKIPTTNGE
251 ATTGDGQRIL SKLGADLIDM DQVQLHPTGF IDPNNRDSAW KFLAAEGLRG
301 LGGILINPAT GRRFVDELQT RDYVTNVIRS ECPKDDNKAY LVMSEATYQE
351 FKNNMDFYMS KNLLRKVTIE QLVKENNLPV TVAEFVQELK EYSTAKQDKF
401 GRSLVINTFG DQVNPSTEVY LGEVTPVVHF TMGGAKINQQ AQVVGKDNKP
451 LAQGLYAAGE VSGGVHGGNR LGGSSLLECV VYGRAAAKDI AGK
```

Show predicted peptides also

Sort Peptides By ◉ Residue Number ○ Increasing Mass ○ Decreasing Mass

Start - End	Observed	Mr(expt)	Mr(calc)	ppm	Miss	Sequence
8 - 23	1993.9766	1992.9693	1993.1441	-88	0	R.QTVVWVLLFSLVIYWK.H (No match)
108 - 115	918.5399	917.5326	917.4528	87	0	K.GVEPLMEK.L Oxidation (M) (No match)
108 - 115	918.5399	917.5326	917.4528	87	0	K.GVEPLMEK.L Oxidation (M) (No match)
313 - 321	1163.6173	1162.6100	1162.6095	0	1	R.RFVDELQTR.D (Ions score 5)
313 - 321	1163.6173	1162.6100	1162.6095	0	1	R.RFVDELQTR.D (No match)
314 - 321	1007.5192	1006.5119	1006.5084	4	0	R.FVDELQTR.D (Ions score 43)

```
314 - 321    1007.5192  1006.5119  1006.5084        4     0  R.FVDELQTR.D  (No match)
471 - 484    1509.7740  1508.7667  1508.7657        1     0  R.LGGSSLLECVVYGR.A  (Ions score 100)
471 - 484    1509.7740  1508.7667  1508.7657        1     0  R.LGGSSLLECVVYGR.A  (No match)
```

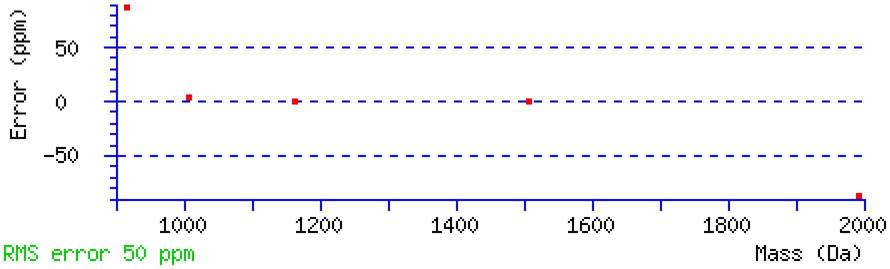

RMS error 50 ppm

```
LOCUS       XP_002497436              493 aa            linear   PLN 22-JUL-2009
DEFINITION  ZYRO0F05522p [Zygosaccharomyces rouxii].
ACCESSION   XP_002497436
VERSION     XP_002497436.1  GI:254583736
DBSOURCE    REFSEQ: accession XM_002497391.1
KEYWORDS    .
SOURCE      Zygosaccharomyces rouxii CBS 732
  ORGANISM  Zygosaccharomyces rouxii CBS 732
            Eukaryota; Fungi; Dikarya; Ascomycota; Saccharomyceta;
            Saccharomycotina; Saccharomycetes; Saccharomycetales;
            Saccharomycetaceae; Zygosaccharomyces.
REFERENCE   1  (residues 1 to 493)
  CONSRTM   The Genolevures Consortium
  TITLE     Comparative genomics of protoploid Saccharomycetaceae
  JOURNAL   Unpublished
REFERENCE   2  (residues 1 to 493)
  AUTHORS   Genoscope -,C.E.A.
  TITLE     Direct Submission
  JOURNAL   Submitted (04-JUN-2009) Genoscope - Centre National de Sequencage :
            BP 191 91006 EVRY cedex - FRANCE (E-mail : seqref@genoscope.cns.fr
            - Web : www.genoscope.cns.fr)
COMMENT     PROVISIONAL REFSEQ: This record has not yet been subject to final
            NCBI review. The reference sequence was derived from CAR28503.
FEATURES             Location/Qualifiers
     source          1..493
                     /organism="Zygosaccharomyces rouxii CBS 732"
                     /strain="CBS 732"
                     /db_xref="taxon:559307"
                     /chromosome="F"
     Protein         1..493
                     /product="hypothetical protein"
                     /calculated_mol_wt=53721
```

```
     Region          32..487
                     /region_name="flavo_cyto_c"
                     /note="flavocytochrome c; TIGR01813"
                     /db_xref="CDD:162543"
     Region          33..490
                     /region_name="NADB_Rossmann"
                     /note="Rossmann-fold NAD(P)(+)-binding proteins; cl09931"
                     /db_xref="CDD:176428"
     CDS             1..493
                     /locus_tag="ZYRO0F05522g"
                     /old_locus_tag="ZYRO-ORF2942"
                     /coded_by="XM_002497391.1:1..1482"
                     /db_xref="GeneID:8205197"
```

Mascot: http://www.matrixscience.com/

Spot 20

Mascot Search Results

Protein View

Match to: **gi|154285406** Score: **234** Expect: **4e-017**
nucleoside-diphosphate kinase [Ajellomyces capsulatus NAm1]

Nominal mass (M_r): **16862**; Calculated pI value: **7.77**
NCBI BLAST search of gi|154285406 against nr
Unformatted sequence string for pasting into other applications

Taxonomy: Ajellomyces capsulatus NAm1
Links to retrieve other entries containing this sequence from NCBI Entrez:
gi|150407139 from Ajellomyces capsulatus NAm1

Fixed modifications: Carbamidomethyl (C)
Variable modifications: Oxidation (M)
Cleavage by Trypsin: cuts C-term side of KR unless next residue is P
Sequence Coverage: **38%**

Matched peptides shown in **Bold Red**

```
  1 MSASEQTFIA IKPDGVQRGL VGPIISRFES RGYKLAAIKL VTPSKEHLEK
 51 HYEDLSSKPF FKGLVTYMLS GPICAMVWEG RDAVKTGRAI LGATNPLASA
101 PGTIRGDFAI DVGRNVCHGS DSVENAKKEI ALWFKPEELV QYQQSQANWV
151 YE
```

Show predicted peptides also

Sort Peptides By ◉ Residue Number ○ Increasing Mass ○ Decreasing Mass

Start - End	Observed	Mr(expt)	Mr(calc)	ppm	Miss	Sequence
1 - 18	1993.9775	1992.9702	1992.9939	-12	0	-.MSASEQTFIAIKPDGVQR.G Oxidation (M) (No match)
89 - 105	1622.9312	1621.9239	1621.9151	5	0	R.AILGATNPLASAPGTIR.G (Ions score 115)
89 - 105	1622.9312	1621.9239	1621.9151	5	0	R.AILGATNPLASAPGTIR.G (No match)
89 - 114	2553.3689	2552.3616	2552.3711	-4	1	R.AILGATNPLASAPGTIRGDFAIDVGR.N (No match)
106 - 114	949.4771	948.4698	948.4665	3	0	R.GDFAIDVGR.N (Ions score 69)
106 - 114	949.4771	948.4698	948.4665	3	0	R.GDFAIDVGR.N (No match)
115 - 127	1416.6149	1415.6076	1415.6099	-2	0	R.NVCHGSDSVENAK.K (No match)
115 - 128	1544.7195	1543.7122	1543.7049	5	1	R.NVCHGSDSVENAKK.E (No match)

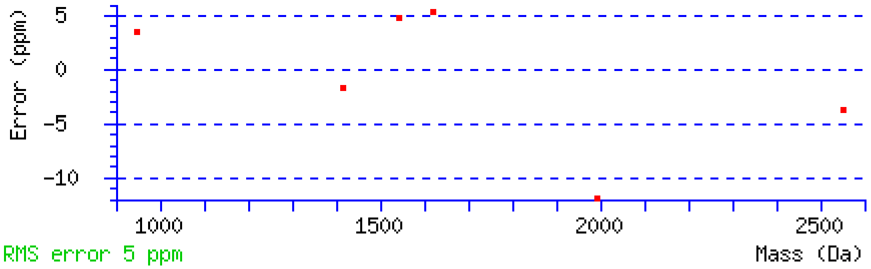


```
LOCUS       XP_001543498             152 aa            linear   PLN 26-FEB-2008
DEFINITION  nucleoside-diphosphate kinase [Ajellomyces capsulatus NAm1].
ACCESSION   XP_001543498
VERSION     XP_001543498.1  GI:154285406
DBSOURCE    REFSEQ: accession XM_001543448.1
KEYWORDS    .
SOURCE      Ajellomyces capsulatus NAm1
  ORGANISM  Ajellomyces capsulatus NAm1
            Eukaryota; Fungi; Dikarya; Ascomycota; Saccharomyceta;
            Pezizomycotina; Leotiomyceta; Eurotiomycetes; Eurotiomycetidae;
            Onygenales; Ajellomycetaceae; Ajellomyces.
REFERENCE   1  (residues 1 to 152)
  AUTHORS   Birren,B., Lander,E., Galagan,J., Nusbaum,C., Devon,K., Ma,L.-J.,
            Henn,M., Jaffe,D., Butler,J., Alvarez,P., Gnerre,S., Grabherr,M.,
            Kleber,M., Mauceli,E., Brockman,W., Rounsley,S., Young,S.,
            LaButti,K., Pushparaj,V., DeCaprio,D., Crawford,M., Koehrsen,M.,
            Engels,R., Montgomery,P., Pearson,M., Howarth,C., Larson,L.,
            Luoma,S., White,J., Yandava,C., Kodira,C., Zeng,Q., Oleary,S.,
            Alvarado,L., Taylor,J., Sil,A. and Goldman,B.
  CONSRTM   The Broad Institute Genome Sequencing Platform
  TITLE     Annotation of the Ajellomyces capsulatus (Histoplasma capsulatum)
            genome
  JOURNAL   Unpublished (2004)
REFERENCE   2  (residues 1 to 152)
  AUTHORS   Birren,B., Ma,L.J., LaButti,K., Lander,E., Taylor,J.,
            Osguthorpe,R., Magrini,V. and Goldman,W.E.
  CONSRTM   The Genome Sequencing Platform, The Genome Assembly Team, The
            Assembly Analysis Team
  TITLE     Direct Submission
  JOURNAL   Submitted (19-AUG-2005) Broad Institute of MIT and Harvard, 320
            Charles Street, Cambridge, MA 02141, USA
COMMENT     PROVISIONAL REFSEQ: This record has not yet been subject to final
            NCBI review. The reference sequence was derived from EDN02680.
            Method: conceptual translation.
FEATURES             Location/Qualifiers
```

```
source          1..152
                /organism="Ajellomyces capsulatus NAm1"
                /strain="NAm1"
                /db_xref="taxon:339724"
Protein         1..152
                /product="nucleoside-diphosphate kinase"
                /calculated_mol_wt=16627
Region          5..134
                /region_name="NDPk_I"
                /note="Nucleoside diphosphate kinase Group I
                (NDPk_I)-like: NDP kinase domains are present in a large
                family of structurally and functionally conserved proteins
                from bacteria to humans that generally catalyze the
                transfer of gamma-phosphates of a nucleoside...; cd04413"
                /db_xref="CDD:58528"
Site            order(12,52,60,88,94,105,115,118,120..121,129)
                /site_type="active"
                /db_xref="CDD:58528"
Site            order(16,21..23,26,29,38..40)
                /site_type="other"
                /note="multimer interface"
                /db_xref="CDD:58528"
CDS             1..152
                /locus_tag="HCAG_00544"
                /coded_by="XM_001543448.1:1..459"
                /db_xref="GeneID:5449951"
```

Mascot: http://www.matrixscience.com/

Spot 21

Mascot Search Results

Protein View

Match to: **gi|164429080** Score: **252** Expect: **6.3e-019**
adenosine kinase [Neurospora crassa OR74A]

Nominal mass (M_r): **49357**; Calculated pI value: **6.02**
NCBI BLAST search of gi|164429080 against nr
Unformatted sequence string for pasting into other applications

Taxonomy: Neurospora crassa OR74A
Links to retrieve other entries containing this sequence from NCBI Entrez:
gi|157072401 from Neurospora crassa OR74A

Fixed modifications: Carbamidomethyl (C)
Variable modifications: Oxidation (M)
Cleavage by Trypsin: cuts C-term side of KR unless next residue is P
Sequence Coverage: **12%**

Matched peptides shown in **Bold Red**

```
  1 MLLTHPITRP QRLIAGKLES LIGVSYAVGA RRHGQPSAAG ATRCFYSTAP
 51 VNPFPAANPS TSNLVRGCDP AVGCFSLSAS NLIPVNNSFH TTTPSRFSIR
101 SNPFSKMAAT KDYRLLCLEN PLLDIQAFGD EALLEKYGLK ANDAILAEEK
151 HQGLFEDLLQ NYDAKLIAGG AAQNTARGAQ YLLPPNSVVY LGGVGDDKYA
201 AILHDAVKQA GLRVEYRVDP KISTGRCGVV ITGHNRSMCT ELGAANHYDL
251 EHLKKPEVWS LVENAEVYYV GGYHFTVCPP AIMELAKQAA SGNKPFILSL
301 SAPFICQFFK EPLDASAPYW DYVIGNEGEA AAYAESHGLN TTDVKEIAKA
351 LANLPKENTQ RKRVAIITQG TEPTIVAIQG EDEVKEYPVH SIDPAKINDT
401 NGAGDAFAGG FAAGVVEGKS IEESIHMGQW LAKLSIQELG PSYPFPKQAY
451 PGHN
```

Show predicted peptides also

Sort Peptides By ● Residue Number ○ Increasing Mass ○ Decreasing Mass

Start - End	Observed	Mr(expt)	Mr(calc)	ppm	Miss	Sequence
141 - 150	1073.5552	1072.5479	1072.5400	7	0	K.ANDAILAEEK.H (No match)
166 - 177	1142.6313	1141.6240	1141.6203	3	0	K.LIAGGAAQNTAR.G (Ions score 80)
166 - 177	1142.6313	1141.6240	1141.6203	3	0	K.LIAGGAAQNTAR.G (No match)
227 - 236	1112.5679	1111.5606	1111.5557	4	0	R.CGVVITGHNR.S (Ions score 52)
227 - 236	1112.5679	1111.5606	1111.5557	4	0	R.CGVVITGHNR.S (No match)
346 - 356	1167.6261	1166.6188	1166.7023	-72	1	K.EIAKALANLPK.E (No match)

434 - 447	1575.8433	1574.8360	1574.8344	1	0	K.LSIQELGPSYPFPK.Q	(Ions score 105)
434 - 447	1575.8433	1574.8360	1574.8344	1	0	K.LSIQELGPSYPFPK.Q	(No match)

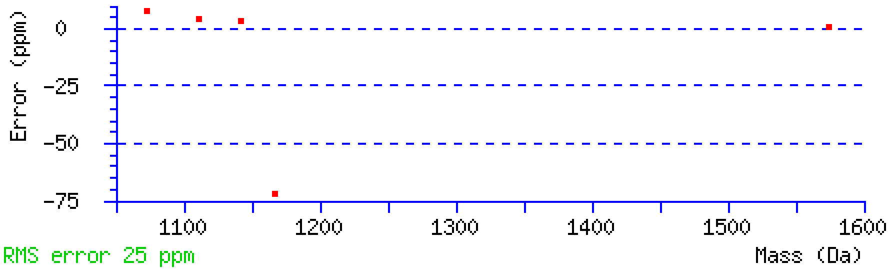


```
LOCUS       XP_957300                454 aa            linear   PLN 10-APR-2008
DEFINITION  adenosine kinase [Neurospora crassa OR74A].
ACCESSION   XP_957300
VERSION     XP_957300.2  GI:164429080
DBSOURCE    REFSEQ: accession XM_952207.2
KEYWORDS    .
SOURCE      Neurospora crassa OR74A
  ORGANISM  Neurospora crassa OR74A
            Eukaryota; Fungi; Dikarya; Ascomycota; Saccharomyceta;
            Pezizomycotina; Leotiomyceta; Sordariomyceta; Sordariomycetes;
            Sordariomycetidae; Sordariales; Sordariaceae; Neurospora.
REFERENCE   1  (residues 1 to 454)
  AUTHORS   Galagan,J.E., Calvo,S.E., Borkovich,K.A., Selker,E.U., Read,N.D.,
            Jaffe,D., FitzHugh,W., Ma,L.J., Smirnov,S., Purcell,S., Rehman,B.,
            Elkins,T., Engels,R., Wang,S., Nielsen,C.B., Butler,J.,
            Endrizzi,M., Qui,D., Ianakiev,P., Bell-Pedersen,D., Nelson,M.A.,
            Werner-Washburne,M., Selitrennikoff,C.P., Kinsey,J.A., Braun,E.L.,
            Zelter,A., Schulte,U., Kothe,G.O., Jedd,G., Mewes,W., Staben,C.,
            Marcotte,E., Greenberg,D., Roy,A., Foley,K., Naylor,J.,
            Stange-Thomann,N., Barrett,R., Gnerre,S., Kamal,M., Kamvysselis,M.,
            Mauceli,E., Bielke,C., Rudd,S., Frishman,D., Krystofova,S.,
            Rasmussen,C., Metzenberg,R.L., Perkins,D.D., Kroken,S., Cogoni,C.,
            Macino,G., Catcheside,D., Li,W., Pratt,R.J., Osmani,S.A.,
            DeSouza,C.P., Glass,L., Orbach,M.J., Berglund,J.A., Voelker,R.,
            Yarden,O., Plamann,M., Seiler,S., Dunlap,J., Radford,A.,
            Aramayo,R., Natvig,D.O., Alex,L.A., Mannhaupt,G., Ebbole,D.J.,
            Freitag,M., Paulsen,I., Sachs,M.S., Lander,E.S., Nusbaum,C. and
            Birren,B.
  TITLE     The genome sequence of the filamentous fungus Neurospora crassa
  JOURNAL   Nature 422 (6934), 859-868 (2003)
   PUBMED   12712197
REFERENCE   2  (residues 1 to 454)
  AUTHORS   Galagan,J., Henn,M.R., Hood,H., Radford,A., Collins,R.,
```

```
            DeCaprio,D., Crawford,M., Koehrsen,M., Engels,R., Montgomery,P.,
            Pearson,M., Howarth,C., Larson,L., White,J., Ledlie,T., Kodira,C.,
            Zeng,Q., Yandava,C., Alvarado,L., O'Leary,S., Bowman,B., Colot,H.,
            Ebbole,D., Rasmussen,C., Baker,C., Kalkman,E., Chen,C.-H., Shi,M.,
            Mathur,R., Lambreghts,R., Mehra,A., Collopy,P., Mehra,A.,
            Schweredtfeger,C., Hong,C., Belden,W., Glass,N.L., Borkovich,K.,
            Dunlap,J., Lander,E., Nusbaum,C., Sachs,M. and Birren,B.
  TITLE     Version 3 gene predictions for the Neurospora crassa assembly 7
  JOURNAL   Unpublished
REFERENCE   3  (residues 1 to 454)
  AUTHORS   Galagan,J., Henn,M.R., Hood,H., Radford,A., Collins,R.,
            DeCaprio,D., Crawford,M., Koehrsen,M., Engels,R., Montgomery,P.,
            Pearson,M., Howarth,C., Larson,L., White,J., Ledlie,T., Kodira,C.,
            Zeng,Q., Yandava,C., Alvarado,L., O'Leary,S., Bowman,B., Colot,H.,
            Ebbole,D., Rasmussen,C., Baker,C., Kalkman,E., Chen,C.-H., Shi,M.,
            Mathur,R., Lambreghts,R., mehra,A., Collopy,P., Mehra,A.,
            Schweredtfeger,C., Hong,C., Belden,W., Glass,N.L., Borkovich,K.,
            Dunlap,J., Lander,E., Nusbaum,C., Sachs,M. and Birren,B.
  TITLE     Version 3 gene predictions for the Neurospora crassa assembly 7
  JOURNAL   Unpublished
REFERENCE   4  (residues 1 to 454)
  AUTHORS   Birren,B., Galagan,J. and Henn,M.R.
  TITLE     Direct Submission
  JOURNAL   Submitted (06-JUL-2007) Broad Institute of MIT and Harvard, 7
            Cambridge Center, Cambridge, MA 02142, USA
REFERENCE   5  (residues 1 to 454)
  AUTHORS   Birren,B.
  TITLE     Direct Submission
  JOURNAL   Submitted (11-MAR-2003) Whitehead Institute/MIT Center for Genome
            Research, 320 Charles Street, Cambridge, MA 02142, USA
COMMENT     PROVISIONAL REFSEQ: This record has not yet been subject to final
            NCBI review. The reference sequence was derived from EAA28064.
            On Jan 3, 2008 this sequence version replaced gi:85084340.
            Method: conceptual translation.
FEATURES             Location/Qualifiers
     source          1..454
                     /organism="Neurospora crassa OR74A"
                     /strain="OR74A"
                     /db_xref="taxon:367110"
                     /chromosome="III"
     Protein         1..454
                     /product="adenosine kinase"
                     /calculated_mol_wt=48800
     Region          112..444
                     /region_name="adenosine_kinase"
                     /note="Adenosine kinase (AK) catalyzes the phosphorylation
                     of ribofuranosyl-containing nucleoside analogues at the
                     5'-hydroxyl using ATP or GTP as the phosphate donor.The
                     physiological function of AK is associated with the
```

```
                     regulation of extracellular...; cd01168"
                     /db_xref="CDD:29352"
     Site            order(120,122,124,144,169..171,174,227,238,240,242,273,
                     304,401..402,405)
                     /site_type="other"
                     /note="substrate binding site"
                     /db_xref="CDD:29352"
     Site            order(368,370..371,374,389,391,397,400,403..404,407,432,
                     436)
                     /site_type="other"
                     /note="ATP binding site"
                     /db_xref="CDD:29352"
     CDS             1..454
                     /locus_tag="NCU00414"
                     /old_locus_tag="NCU00414.1"
                     /coded_by="XM_952207.2:1..1365"
                     /db_xref="GeneID:3873447"
```

Mascot: http://www.matrixscience.com/

Spot 22

Mascot Search Results

Protein View

Match to: **gi|242777912** Score: **157** Expect: **2e-009**
conserved hypothetical protein [Talaromyces stipitatus ATCC 10500]

Nominal mass (M_r): **42847**; Calculated pI value: **5.79**
NCBI BLAST search of gi|242777912 against nr
Unformatted sequence string for pasting into other applications

Taxonomy: Talaromyces stipitatus ATCC 10500
Links to retrieve other entries containing this sequence from NCBI Entrez:
gi|218722748 from Talaromyces stipitatus ATCC 10500

Fixed modifications: Carbamidomethyl (C)
Variable modifications: Oxidation (M)
Cleavage by Trypsin: cuts C-term side of KR unless next residue is P
Sequence Coverage: **19%**

Matched peptides shown in **Bold Red**

```
  1 MSAKPIFVAT HPRACSTAFE RVFMTRRDSL QCIHEPFGDA FYFGPERLSA
 51 RYENDEKARL DSGFSTSTYK TIFDRIESET TEGKRIFIKD IIHYLVPPDG
101 KPASIAPSLF KVKRGIGTNG EINGLTNGHA EVNGVTTNGG TPVKAEVPPY
151 PYPTEAEPGN PTVVPLELLS KFHFTFLIRD PHYSIPSYFR CTIPPLDDVT
201 GFHEFSPSEA GYDEVRRVFD YLRSVGLIGP RVATTTHAED TANKQTNGTT
251 HRHDNGSMGV EICVIDADDL LDNPSLMIES YCKSVGIPFE PEMLRWDTEE
301 DHAYARAAFE KWKGFHEDAI ASKELKARTH SKSFKSEEEF DAEWREKFGE
351 KGAKIIRETV NRNMADYHYM KHFALKV
```

Show predicted peptides also

Sort Peptides By ◉ Residue Number ○ Increasing Mass ○ Decreasing Mass

Start - End	Observed	Mr(expt)	Mr(calc)	ppm	Miss	Sequence
14 - 21	941.4161	940.4088	940.4073	2	0	R.ACSTAFER.V (Ions score 26)
14 - 21	941.4161	940.4088	940.4073	2	0	R.ACSTAFER.V (No match)
28 - 47	2385.0583	2384.0510	2384.0532	-1	0	R.DSLQCIHEPFGDAFYFGPER.L (Ions score 114)
28 - 47	2385.0583	2384.0510	2384.0532	-1	0	R.DSLQCIHEPFGDAFYFGPER.L (No match)
60 - 75	1837.9657	1836.9584	1836.8894	38	1	R.LDSGFSTSTYKTIFDR.I (No match)
60 - 75	1837.9657	1836.9584	1836.8894	38	1	R.LDSGFSTSTYKTIFDR.I (No match)
114 - 144	3034.4233	3033.4160	3033.5228	-35	1	K.RGIGTNGEINGLTNGHAEVNGVTTNGGTPVK.A (No match)

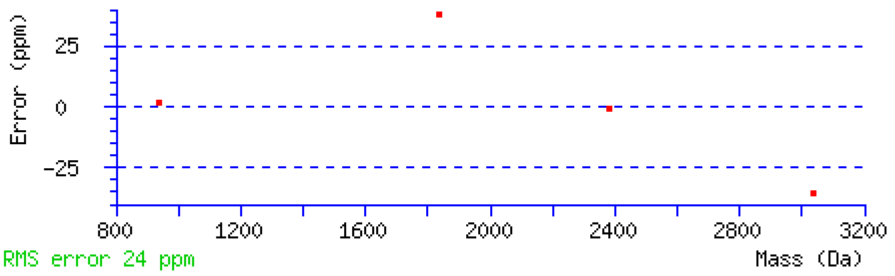


```
LOCUS       XP_002479129             377 aa            linear   PLN 02-JUL-2009
DEFINITION  conserved hypothetical protein [Talaromyces stipitatus ATCC 10500].
ACCESSION   XP_002479129
VERSION     XP_002479129.1  GI:242777912
DBSOURCE    REFSEQ: accession XM_002479084.1
KEYWORDS    .
SOURCE      Talaromyces stipitatus ATCC 10500
  ORGANISM  Talaromyces stipitatus ATCC 10500
            Eukaryota; Fungi; Dikarya; Ascomycota; Saccharomyceta;
            Pezizomycotina; Leotiomyceta; Eurotiomycetes; Eurotiomycetidae;
            Eurotiales; Trichocomaceae; Talaromyces.
REFERENCE   1  (residues 1 to 377)
  AUTHORS   Fedorova,N.D., Joardar,V., Maiti,R., Schobel,S., Amedeo,P.,
            Galens,K., Inman,J.M., Galinsky,K.J., White,O.R., Whitty,B.R.,
            Wortman,J.R. and Nierman,W.C.
  TITLE     Direct Submission
  JOURNAL   Submitted (01-OCT-2007) J. Craig Ventor Institue, 9704 Medical
            Center Drive, Rockville, MD 20850, USA
REFERENCE   2  (residues 1 to 377)
  AUTHORS   Nierman,W.C.
  TITLE     Direct Submission
  JOURNAL   Submitted (02-MAY-2007) The Institute for Genomic Research, 9712
            Medical Center Drive, Rockville, MD 20850, USA
COMMENT     PROVISIONAL REFSEQ: This record has not yet been subject to final
            NCBI review. The reference sequence was derived from
            mrna.TSTA_094120A.
            Method: conceptual translation.
FEATURES             Location/Qualifiers
     source          1..377
                     /organism="Talaromyces stipitatus ATCC 10500"
                     /strain="ATCC 10500"
                     /culture_collection="ATCC:10500"
                     /db_xref="taxon:441959"
     Protein         1..377
                     /product="hypothetical protein"
```

```
                     /calculated_mol_wt=42458
     CDS             1..377
                     /locus_tag="TSTA_094120"
                     /coded_by="XM_002479084.1:1..1134"
                     /note="encoded by transcript TSTA_094120A"
                     /db_xref="GeneID:8103740"
```

**Mascot:** http://www.matrixscience.com/

Spot 23

Mascot Search Results

Protein View

Match to: **gi|189196154** Score: **504** Expect: **4e-044**
NADP-specific glutamate dehydrogenase [Pyrenophora tritici-repentis Pt-1C-BFP]

Nominal mass (M_r): **49094**; Calculated pI value: **5.88**
NCBI BLAST search of gi|189196154 against nr
Unformatted sequence string for pasting into other applications

Taxonomy: Pyrenophora tritici-repentis Pt-1C-BFP
Links to retrieve other entries containing this sequence from NCBI Entrez:
gi|187980294 from Pyrenophora tritici-repentis Pt-1C-BFP

Fixed modifications: Carbamidomethyl (C)
Variable modifications: Oxidation (M)
Cleavage by Trypsin: cuts C-term side of KR unless next residue is P
Sequence Coverage: **22%**

Matched peptides shown in **Bold Red**

```
  1 MSEPEFEQAR KELVSTLEAS SLFSKNPEYK KALEVVSVPE RIIQFRVVWE
 51 NDKGECQVQK GYRVQFNSAL GPYKGGLRFH PTVNLSILKF LGFEQIFKNA
101 LTGLNMGGGK GGCDFDPKGK SDNEIRKFCV AFMRELNKHI GADTDVPAGD
151 IGVGGREIGY LFGAYRAERN RWEGVLTGKG GSWGGSLIRP EATGYGLVYY
201 VEHMINYASG GKESFAGKRV ALSGSGNVAQ YAALKIIELG GTVISLSDSK
251 GALIAEDDKG FTPEIINQIA ALKLERKALT ALENHNFKYI EGARPWKEVN
301 KVDVALPSAT QNEVSEDEAK ALIESGAKYI AEGSNMGCTQ EAIEVFEAHR
351 REKKGDAIWY APGKAANAGG VAVSGLEMAQ NSQRLSWTAE QVDEKLKGIM
401 KDCFENCLST AKEYFTPAEG EFPSLVGGAN VAGFRKVAAA MHDQGDWW
```

Show predicted peptides also

Sort Peptides By ◉ Residue Number ○ Increasing Mass ○ Decreasing Mass

Start - End	Observed	Mr(expt)	Mr(calc)	ppm	Miss	Sequence
64 - 74	1223.6378	1222.6305	1222.6346	-3	0	R.VQFNSALGPYK.G (No match)
64 - 78	1606.8733	1605.8660	1605.8627	2	1	R.VQFNSALGPYKGGLR.F (Ions score 51)
64 - 78	1606.8733	1605.8660	1605.8627	2	1	R.VQFNSALGPYKGGLR.F (No match)
75 - 89	1651.9641	1650.9568	1650.9570	-0	1	K.GGLRFHPTVNLSILK.F (No match)
79 - 89	1268.7380	1267.7307	1267.7289	1	0	R.FHPTVNLSILK.F (Ions score 69)
79 - 89	1268.7380	1267.7307	1267.7289	1	0	R.FHPTVNLSILK.F (No match)
90 - 98	1128.6123	1127.6050	1127.6015	3	0	K.FLGFEQIFK.N (Ions score 69)

90 - 98	1128.6123	1127.6050	1127.6015	3	0	K.FLGFEQIFK.N	(No match)
139 - 156	1706.8507	1705.8434	1705.8384	3	0	K.HIGADTDVPAGDIGVGGR.E	(Ions score 116)
139 - 156	1706.8507	1705.8434	1705.8384	3	0	K.HIGADTDVPAGDIGVGGR.E	(No match)
170 - 179	1159.6238	1158.6165	1158.6145	2	1	R.NRWEGVLTGK.G	(Ions score 36)
170 - 179	1159.6238	1158.6165	1158.6145	2	1	R.NRWEGVLTGK.G	(No match)
219 - 235	1704.9283	1703.9210	1703.9318	-6	1	K.RVALSGSGNVAQYAALK.I	(No match)
220 - 235	1548.8413	1547.8340	1547.8307	2	0	R.VALSGSGNVAQYAALK.I	(No match)
365 - 384	1930.9442	1929.9369	1929.9326	2	0	K.AANAGGVAVSGLEMAQNSQR.L	(Ions score 108)
365 - 384	1930.9442	1929.9369	1929.9326	2	0	K.AANAGGVAVSGLEMAQNSQR.L	(No match)
365 - 384	1946.9397	1945.9324	1945.9276	2	0	K.AANAGGVAVSGLEMAQNSQR.L	Oxidation (M) (No match)
365 - 384	1946.9397	1945.9324	1945.9276	2	0	K.AANAGGVAVSGLEMAQNSQR.L	Oxidation (M) (No match)

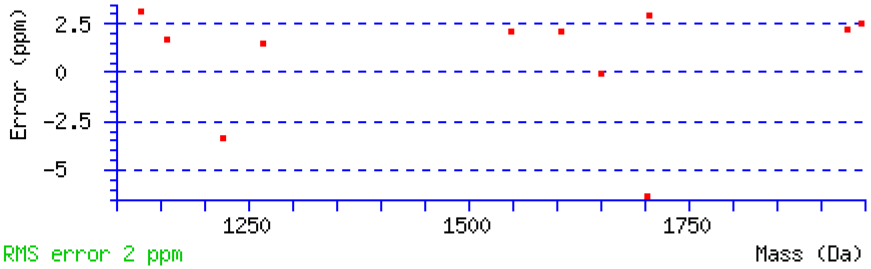


```
LOCUS       XP_001934415             448 aa            linear   PLN 30-MAY-2008
DEFINITION  NADP-specific glutamate dehydrogenase [Pyrenophora tritici-repentis
            Pt-1C-BFP].
ACCESSION   XP_001934415
VERSION     XP_001934415.1  GI:189196154
DBSOURCE    REFSEQ: accession XM_001934380.1
KEYWORDS    .
SOURCE      Pyrenophora tritici-repentis Pt-1C-BFP
  ORGANISM  Pyrenophora tritici-repentis Pt-1C-BFP
            Eukaryota; Fungi; Dikarya; Ascomycota; Saccharomyceta;
            Pezizomycotina; Leotiomyceta; Dothideomyceta; Dothideomycetes;
            Pleosporomycetidae; Pleosporales; Pleosporineae; Pleosporaceae;
            Pyrenophora.
REFERENCE   1  (residues 1 to 448)
  AUTHORS   Birren,B., Lander,E., Galagan,J., Nusbaum,C., Devon,K., Ma,L.-J.,
            Jaffe,D., Butler,J., Alvarez,P., Gnerre,S., Grabherr,M., Kleber,M.,
            Mauceli,E., Brockman,W., MacCallum,I.A., Young,S., LaButti,K.,
            DeCaprio,D., Crawford,M., Koehrsen,M., Engels,R., Montgomery,P.,
            Pearson,M., Howarth,C., Larson,L., White,J., Yandava,C., Kodira,C.,
            Guigo,R., Borodovsky,M., Zeng,Q., O'Leary,S., Alvarado,L.,
            Pandelova,I. and Ciuffetti,L.
  CONSRTM   The Broad Institute Genome Sequencing Platform
  TITLE     Genome Sequence of Pyrenophora tritici-repentis
  JOURNAL   Unpublished
```

```
REFERENCE   2  (residues 1 to 448)
  AUTHORS   Birren,B., Lander,E., Galagan,J., Nusbaum,C., Devon,K., Ma,L.-J.,
            Jaffe,D., Butler,J., Alvarez,P., Gnerre,S., Grabherr,M., Kleber,M.,
            Mauceli,E., Brockman,W., MacCallum,I.A., Young,S., LaButti,K.,
            DeCaprio,D., Crawford,M., Koehrsen,M., Engels,R., Montgomery,P.,
            Pearson,M., Howarth,C., Larson,L., White,J., Yandava,C., Kodira,C.,
            Zeng,Q., O'Leary,S., Alvarado,L., Ciuffetti,L. and Pandelova,I.
  CONSRTM   The Broad Institute Genome Sequencing Platform
  TITLE     Direct Submission
  JOURNAL   Submitted (16-MAR-2007) Broad Institute of MIT and Harvard, 7
            Cambridge Center, Cambridge, MA 02142, USA
COMMENT     PROVISIONAL REFSEQ: This record has not yet been subject to final
            NCBI review. The reference sequence was derived from EDU46920.
            Method: conceptual translation.
FEATURES             Location/Qualifiers
     source          1..448
                     /organism="Pyrenophora tritici-repentis Pt-1C-BFP"
                     /strain="Pt-1C-BFP"
                     /db_xref="taxon:426418"
     Protein         1..448
                     /product="NADP-specific glutamate dehydrogenase"
                     /calculated_mol_wt=48651
     Region          3..445
                     /region_name="PRK09414"
                     /note="glutamate dehydrogenase; Provisional; PRK09414"
                     /db_xref="CDD:169848"
     Region          39..166
                     /region_name="ELFV_dehydrog_N"
                     /note="Glu/Leu/Phe/Val dehydrogenase, dimerization domain;
                     pfam02812"
                     /db_xref="CDD:145786"
     Region          177..446
                     /region_name="NAD_bind_2_Glu_DH"
                     /note="NAD(P) binding domain of glutamate dehydrogenase,
                     subgroup 2; cd05313"
                     /db_xref="CDD:133455"
     Site            order(226..228,248..249,309..310,333..335)
                     /site_type="other"
                     /note="NAD(P) binding site"
                     /db_xref="CDD:133455"
     CDS             1..448
                     /locus_tag="PTRG_04082"
                     /coded_by="XM_001934380.1:1..1347"
                     /db_xref="GeneID:6342318"
```

Mascot: http://www.matrixscience.com/

Spot 24

Mascot Search Results

Protein View

Match to: **gi|238840987** Score: **391** Expect: **7.9e-033**
eukaryotic translation initiation factor 5A-1 [Microsporum canis CBS 113480]

Nominal mass (M_r): **17884**; Calculated pI value: **5.33**
NCBI BLAST search of gi|238840987 against nr
Unformatted sequence string for pasting into other applications

Taxonomy: Microsporum canis CBS 113480

Fixed modifications: Carbamidomethyl (C)
Variable modifications: Oxidation (M)
Cleavage by Trypsin: cuts C-term side of KR unless next residue is P
Sequence Coverage: **30%**

Matched peptides shown in **Bold Red**

```
  1 MADDEQHNVT FENADAGAST TYPMQCSALR KNGHVVIKGR PCKIVEMSTS
 51 KTGKHGHAKV HLVAIDIFTG KKLEDLSPST HNMDVPNVRR QEYQLVDITD
101 DGFLNLMKED GTPKDDVKLP DNEVGEKITK LFKVEEKDVN VVVLTAMGEE
151 CAMDAKEMAH
```

Show predicted peptides also

Sort Peptides By ◉ Residue Number ○ Increasing Mass ○ Decreasing Mass

Start - End	Observed	Mr(expt)	Mr(calc)	ppm	Miss	Sequence
60 - 71	1312.7632	1311.7559	1311.7551	1	0	K.VHLVAIDIFTGK.K (Ions score 109)
60 - 71	1312.7632	1311.7559	1311.7551	1	0	K.VHLVAIDIFTGK.K (No match)
60 - 72	1440.8619	1439.8546	1439.8500	3	1	K.VHLVAIDIFTGKK.L (Ions score 102)
60 - 72	1440.8619	1439.8546	1439.8500	3	1	K.VHLVAIDIFTGKK.L (No match)
72 - 89	2052.0205	2051.0132	2051.0106	1	1	K.KLEDLSPSTHNMDVPNVR.R (Ions score 112)
72 - 89	2052.0205	2051.0132	2051.0106	1	1	K.KLEDLSPSTHNMDVPNVR.R (No match)
72 - 89	2068.0151	2067.0078	2067.0055	1	1	K.KLEDLSPSTHNMDVPNVR.R Oxidation (M) (Ions score 50)
72 - 89	2068.0151	2067.0078	2067.0055	1	1	K.KLEDLSPSTHNMDVPNVR.R Oxidation (M) (No match)
73 - 89	1923.9226	1922.9153	1922.9156	-0	0	K.LEDLSPSTHNMDVPNVR.R (No match)
73 - 89	1923.9226	1922.9153	1922.9156	-0	0	K.LEDLSPSTHNMDVPNVR.R (No match)
73 - 89	1939.9261	1938.9188	1938.9105	4	0	K.LEDLSPSTHNMDVPNVR.R Oxidation (M) (Ions score 36)
73 - 89	1939.9261	1938.9188	1938.9105	4	0	K.LEDLSPSTHNMDVPNVR.R Oxidation (M) (No match)
138 - 156	2084.0149	2083.0076	2082.9272	39	0	K.DVNVVVLTAMGEECAMDAK.E 2 Oxidation (M) (No match)

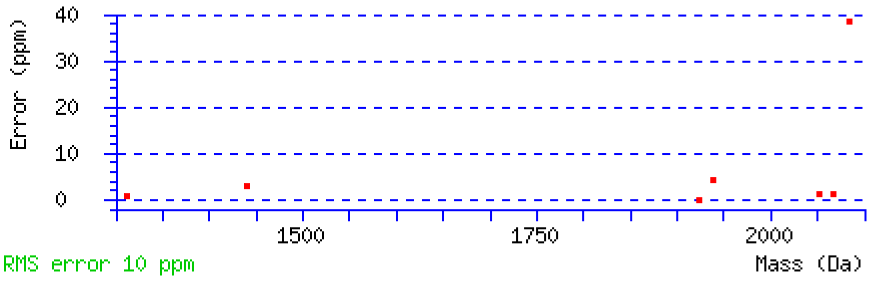

```
LOCUS       EEQ30649                 160 aa            linear   PLN 03-JUN-2009
DEFINITION  eukaryotic translation initiation factor 5A-1 [Microsporum canis
            CBS 113480].
ACCESSION   EEQ30649
VERSION     EEQ30649.1  GI:238840987
DBSOURCE    accession DS995703.1
KEYWORDS    .
SOURCE      Arthroderma otae CBS 113480 (anamorph: Microsporum canis CBS
            113480)
  ORGANISM  Arthroderma otae CBS 113480
            Eukaryota; Fungi; Dikarya; Ascomycota; Saccharomyceta;
            Pezizomycotina; Leotiomyceta; Eurotiomycetes; Eurotiomycetidae;
            Onygenales; Arthrodermataceae; Arthroderma.
REFERENCE   1  (residues 1 to 160)
  AUTHORS   Cuomo,C., Henn,M.R., Young,S.K., Kodira,C.D., Zeng,Q., Koehrsen,M.,
            Alvarado,L., Berlin,A., Borenstein,D., Chen,Z., Engels,R.,
            Freedman,E., Gellesch,M., Goldberg,J., Griggs,A., Gujja,S.,
            Heiman,D., Hepburn,T., Howarth,C., Jen,D., Larson,L., Lewis,B.,
            Mehta,T., Park,D., Pearson,M., Roberts,A., Saif,S., Shea,T.,
            Shenoy,N., Sisk,P., Stolte,C., Sykes,S., Walk,T., White,J.,
            Yandava,C., Guigo,R., Borodovsky,M., White,T.C., Oliver,B.G.,
            Graser,Y., Abdel-Rahman,S., Gurr,S.J., Martinez-Rossi,N.,
            Summerbell,R., Lander,E., Nusbaum,C., Galagan,J. and Birren,B.
  CONSRTM   The Broad Institute Genome Sequencing Platform
  TITLE     Annotation of Microsporum canis strain CBS 113480
  JOURNAL   Unpublished
REFERENCE   2  (residues 1 to 160)
  AUTHORS   Cuomo,C., Henn,M.R., Jaffe,D., Young,S., Gnerre,S., Berlin,A.,
            Heiman,D., Hepburn,T., Sykes,S., Alvarado,L., Kodira,C.D.,
            White,T.C., Oliver,B.G., Graser,Y., Abdel-Rahman,S., Gurr,S.J.,
            Martinez-Rossi,N., Summerbell,R., Lander,E., Nusbaum,C., Galagan,J.
            and Birren,B.
  CONSRTM   The Broad Institute Genome Sequencing Platform
  TITLE     Direct Submission
  JOURNAL   Submitted (07-OCT-2008) Broad Institute of MIT and Harvard, 7
```

```
            Cambridge Center, Cambridge, MA 02142, USA
COMMENT     Method: conceptual translation.
FEATURES             Location/Qualifiers
     source          1..160
                     /organism="Arthroderma otae CBS 113480"
                     /strain="CBS 113480"
                     /db_xref="taxon:554155"
     Protein         1..160
                     /product="eukaryotic translation initiation factor 5A-1"
     Region          18..156
                     /region_name="eIF_5A"
                     /note="translation initiation factor eIF-5A; TIGR00037"
                     /db_xref="CDD:129148"
     Region          88..158
                     /region_name="S1_eIF5A"
                     /note="S1_eIF5A: Eukaryotic translation Initiation Factor
                     5A (eIF5A), S1-like RNA-binding domain. eIF5A is an
                     evolutionarily conserved protein found in eukaryotes.
                     eIF5A is the only protein known to have the unusual amino
                     acid hypusine. Hypusine is essential...; cd04468"
                     /db_xref="CDD:88433"
     Site            order(114,142,149..150)
                     /site_type="other"
                     /note="RNA binding site"
                     /db_xref="CDD:88433"
     CDS             1..160
                     /locus_tag="MCYG_03468"
                     /coded_by="join(DS995703.1:1657332..1657349,
                     DS995703.1:1657493..1657759,DS995703.1:1657827..1657959,
                     DS995703.1:1658049..1658113)"
```

Mascot: http://www.matrixscience.com/

Spot 25

Mascot Search Results

Protein View

Match to: **gi|145613040** Score: **306** Expect: **2.5e-024**
conserved hypothetical protein [Magnaporthe grisea 70-15]

Nominal mass (M_r): **25180**; Calculated pI value: **5.73**
NCBI BLAST search of gi|145613040 against nr
Unformatted sequence string for pasting into other applications

Taxonomy: Magnaporthe grisea 70-15
Links to retrieve other entries containing this sequence from NCBI Entrez:
gi|145020105 from Magnaporthe grisea 70-15

Fixed modifications: Carbamidomethyl (C)
Variable modifications: Oxidation (M)
Cleavage by Trypsin: cuts C-term side of KR unless next residue is P
Sequence Coverage: **21%**

Matched peptides shown in **Bold Red**

```
  1 MAEEQRPAPL RLGTEAPNFK AETTKGPIDF HEFIGSNWVI LFSHPEDFTP
 51 VCTTELGEFA RLEPEFTKRG VKLIGLSANT VGSHDGWIKD INDVTGSHVA
101 FPIIADKERK VAYLYDMLDY QDTTNVDEKG IAFTIRSVFI IDPAKKIRTI
151 LSYPASTGRN SAEVLRIVDS LQTGDKHKVT TPINWVPGDD VIVHPSIKDE
201 QAKDLFPNFR AVKPYLRFTP LPKE
```

Show predicted peptides also

Sort Peptides By ◉ Residue Number ○ Increasing Mass ○ Decreasing Mass

Start - End	Observed	Mr(expt)	Mr(calc)	ppm	Miss	Sequence
62 - 68	863.4529	862.4456	862.4436	2	0	R.LEPEFTK.R (No match)
62 - 69	1019.5555	1018.5482	1018.5447	3	1	R.LEPEFTKR.G (Ions score 68)
62 - 69	1019.5555	1018.5482	1018.5447	3	1	R.LEPEFTKR.G (No match)
149 - 159	1165.6234	1164.6161	1164.6139	2	0	R.TILSYPASTGR.N (Ions score 103)
149 - 159	1165.6234	1164.6161	1164.6139	2	0	R.TILSYPASTGR.N (No match)
167 - 176	1075.5903	1074.5830	1074.5557	25	0	R.IVDSLQTGDK.H (No match)
179 - 198	2187.1755	2186.1682	2186.1736	-2	0	K.VTTPINWVPGDDVIVHPSIK.D (Ions score 107)
179 - 198	2187.1755	2186.1682	2186.1736	-2	0	K.VTTPINWVPGDDVIVHPSIK.D (No match)

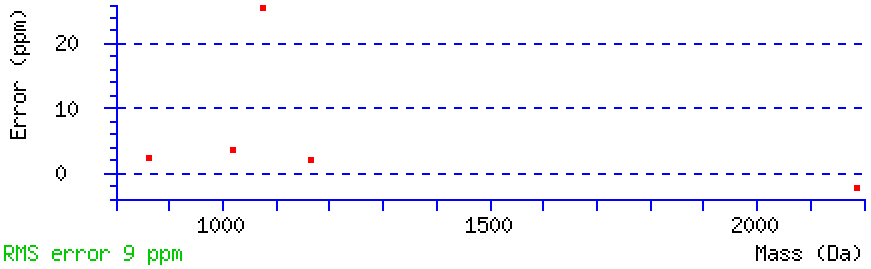

Mascot: http://www.matrixscience.com/

Spot 26

Mascot Search Results

Protein View

Match to: **gi|225877962** Score: **146** Expect: **2.5e-008**
putative saccharopine dehydrogenase (NAD+,L-lysine-forming) [Gibberella fujikuroi]

Nominal mass (M_r): **43543**; Calculated pI value: **5.17**
NCBI BLAST search of gi|225877962 against nr
Unformatted sequence string for pasting into other applications

Taxonomy: Gibberella fujikuroi

Fixed modifications: Carbamidomethyl (C)
Variable modifications: Oxidation (M)
Cleavage by Trypsin: cuts C-term side of KR unless next residue is P
Sequence Coverage: **11%**

Matched peptides shown in **Bold Red**

```
  1 MSDYPHILLR AEEKPLEHRS FSPAIIKTLV DAGYPISVER SSTDPKFKRI
 51 FEDSEYEAAG ARLVDTGVWP NAEPGTIILG LKELPSEDFP LKNDHITFAH
101 CYKNQGGWEQ VLGRWARGGS RLYDLEFLVD EQGRRVSAFG YHAGFAGAAL
151 GIKTLAHQLQ GSSSKLPSVE TFTDGRGYYL NEDELVNQIR EDLAKAEKAL
201 GRKPTALVLG ALGRCGKGAV DLFLKAGMPD DNITRWDLNE TKDRDGPYEE
251 IAKADVFLNA IYLSKPIPPF INQELLAKQG RNLAVVIDVS CDTTNPHNPI
301 PIYSINTTFE DPTVPVEIKD DQNNLPLSVI SIDHLPSMLP REASEAFSEG
351 LKESLLTLKD RETSRVWTDA EKLFHEKVAL LPEELRTKSV
```

Show predicted peptides also

Sort Peptides By ◉ Residue Number ○ Increasing Mass ○ Decreasing Mass

Start - End	Observed	Mr(expt)	Mr(calc)	ppm	Miss	Sequence	
28 - 46	2035.0815	2034.0742	2034.0269	23	1	K.TLVDAGYPISVERSSTDPK.F	(No match)
135 - 153	1893.0167	1892.0094	1892.0057	2	1	R.RVSAFGYHAGFAGAALGIK.T	(No match)
136 - 153	1736.9139	1735.9066	1735.9046	1	0	R.VSAFGYHAGFAGAALGIK.T	(Ions score 131)
136 - 153	1736.9139	1735.9066	1735.9046	1	0	R.VSAFGYHAGFAGAALGIK.T	(No match)
218 - 225	862.5034	861.4961	861.4960	0	0	K.GAVDLFLK.A	(No match)

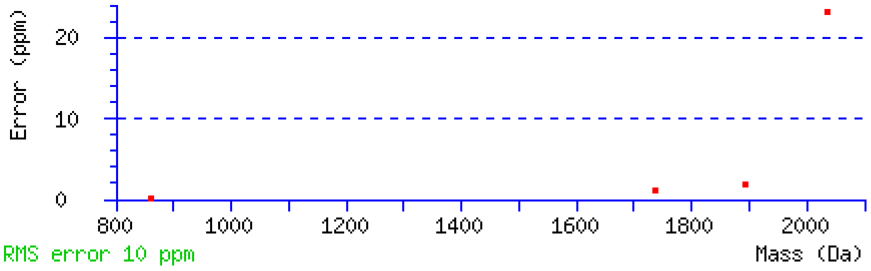

**Mascot:** http://www.matrixscience.com/

Spot 27

Mascot Search Results

Protein View

```
Match to: gi|194669270 Score: 88 Expect: 0.017
PREDICTED: similar to endonuclease reverse transcriptase [Bos taurus]

Nominal mass (Mr): 150298; Calculated pI value: 9.68
NCBI BLAST search of gi|194669270 against nr
Unformatted sequence string for pasting into other applications

Taxonomy: Bos taurus

Fixed modifications: Carbamidomethyl (C)
Variable modifications: Oxidation (M)
Cleavage by Trypsin: cuts C-term side of KR unless next residue is P
Sequence Coverage: 24%

Matched peptides shown in Bold Red

   1 MAMGTYLSVI TLNVNGLNAP TKRQRLAEWI QKQDPYICCL QETHLKTGDT
  51 YRLKVKGWKK IFHANRDQKK AGVAILISDK IDFKTKAVKR DKEGHYIMIT
 101 GSIQEEDITI INIYAPNTGA PQYVRQLLTS MKGEINNNTI IVGYLNTPLT
 151 PMDRSTKQKI NKETQTLNDT IDQLDLIDIY RSFHPKTMNF TFFSSAHGTF
 201 SRIDHILGHK ASLGKFKKIE IIPSIFSDHN AVRLDLNYRR KTIKNSNIWR
 251 LNNTLLNNQQ ITEEIKKEIK ICIETNENEN TTTQNLWDTV KAVLRGKFIA
 301 IQAHLKKQEK SQINNLTLHL KQLEKEEMKN PRVSRRKEIL KIRAEINAKE
 351 TKETIAKINK TKSWFFERIN KIDKPLARLI KKQREKNQIH KVRNQNGEIT
 401 RDNTEIQRII RDYYQQLYAN KMDNVEEMDK FLEKYNFPKL DQEEIENLNR
 451 PITSTEIETV IKNLPANKSP GPDGFTAEFY QKFREELTPV LLKLFQKIAE
 501 EGKLPNSFYE ATITLIPKPD KDPTKKENYR PISLMNIDAK ILNKILAIRI
 551 QQHIKKIIHH DQVGFIPGMQ GFFNIRKSVN VIHHINKLKN KNHMIISIDA
 601 EKAFDKIQHP FMIKTLQKAG IEGTYLNIIK AIYDKPTANI ILNGEKLKAF
 651 PLKSGTRQGC PLSPLLFNIV LEVLATAIRA EKEIKGIQIG KEEVKLSLFA
 701 DDMILYIENP KESTRKLLEI INDYSKVAGY KINTQKSLAF LYTNNEKTER
 751 EIKETIPFTI ATERIKYLGI YLPKETKDLY IENYKTLVKE IKEDTNRWRN
 801 IPCSWIGRIN IVKMSILPKA IYRFNAIPIK LPTVFFTELE QIISQFVWKY
 851 KKPRIAKAIL KKKNGTGGIN LPDFRLYYKA TVIKTVWYWH KDRNIDQWNK
 901 IESPEINPHT YGHLIFDKGG KNIQWIKDNL FNKWCWENWS TTCKRMKLEH
 951 FLTPYTKINS KWIKDLNVRP ETIKLLEENI GKTLSDIHHS RILYDPPPRI
1001 LEIKAKINKW DLINLKSFCT SKETISKVKR QPSEWEKIIA NEATDKQLIS
1051 KIYKQLLQLN SRKINDPIKK WAKELNRHFS KKDIQMANKH MKRCSTSLII
1101 REMQIKTTMR YHFTPVRMAA IQKSTNNKCW RGCGEKGTLL HCWWECKLVQ
1151 PLWRTVWRFL KKLEIELPYD PAIPLLGIHT EETRRERDTC TPVFITALFI
1201 IARTWKQPRC PSADEWIRKL WYIYTMEYYS AIKKNTFESV LMRWMKLEPI
1251 IQSEVSQKEK HQYSILTHIY GI
```

Show predicted peptides also

Sort Peptides By ◉ Residue Number ○ Increasing Mass ○ Decreasing Mass

Start - End	Observed	Mr(expt)	Mr(calc)	ppm	Miss	Sequence
2 - 22	2177.1362	2176.1289	2176.1562	-13	0	M.AMGTYLSVITLNVNGLNAPTK.R (No match)
61 - 69	1128.5367	1127.5294	1127.5836	-48	1	K.IFHANRDQK.K (No match)
71 - 84	1489.7719	1488.7646	1488.8552	-61	1	K.AGVAILISDKIDFK.T (No match)
133 - 157	2777.4697	2776.4624	2776.4065	20	1	K.GEINNNTIIVGYLNTPLTPMDRSTK.Q Oxidation (M) (No match)
187 - 202	1837.9470	1836.9397	1836.8254	62	0	K.TMNFTFFSSAHGTFSR.I (No match)
251 - 266	1884.9423	1883.9350	1883.9952	-32	0	R.LNNTLLNNQQITEEIK.K (No match)
251 - 266	1884.9423	1883.9350	1883.9952	-32	0	R.LNNTLLNNQQITEEIK.K (No match)
251 - 267	2013.0337	2012.0264	2012.0902	-32	1	R.LNNTLLNNQQITEEIKK.E (No match)
463 - 482	2181.1655	2180.1582	2180.0538	48	1	K.NLPANKSPGPDGFTAEFYQK.F (No match)
504 - 521	2047.0862	2046.0789	2046.1037	-12	0	K.LPNSFYEATITLIPKPDK.D (No match)
527 - 540	1663.7385	1662.7312	1662.8399	-65	0	K.ENYRPISLMNIDAK.I (No match)
557 - 576	2326.1904	2325.1831	2325.1841	-0	0	K.IIHHDQVGFIPGMQGFFNIR.K (No match)
607 - 614	1013.5328	1012.5255	1012.5528	-27	0	K.IQHPFMIK.T (No match)
631 - 646	1759.8478	1758.8405	1758.9516	-63	0	K.AIYDKPTANIILNGEK.L (No match)
631 - 646	1759.8478	1758.8405	1758.9516	-63	0	K.AIYDKPTANIILNGEK.L (No match)
683 - 691	985.5443	984.5370	984.5968	-61	1	K.EIKGIQIGK.E (No match)
683 - 691	985.5443	984.5370	984.5968	-61	1	K.EIKGIQIGK.E (No match)
692 - 711	2383.2380	2382.2307	2382.2028	12	1	K.EEVKLSLFADDMILYIENPK.E Oxidation (M) (No match)
751 - 764	1647.7527	1646.7454	1646.8879	-87	1	R.EIKETIPFTIATER.I (No match)
767 - 777	1324.6663	1323.6590	1323.7438	-64	1	K.YLGIYLPKETK.D (No match)
892 - 900	1188.6536	1187.6463	1187.5683	66	1	K.DRNIDQWNK.I (No match)
1052 - 1062	1375.6859	1374.6786	1374.7983	-87	1	K.IYKQLLQLNSR.K (No match)
1082 - 1089	947.5059	946.4986	946.4906	9	1	K.KDIQMANK.H (No match)
1102 - 1110	1137.5371	1136.5298	1136.5682	-34	1	R.EMQIKTTMR.Y (No match)
1102 - 1110	1137.5371	1136.5298	1136.5682	-34	1	R.EMQIKTTMR.Y (No match)
1188 - 1203	1837.9470	1836.9397	1836.9808	-22	0	R.DTCTPVFITALFIIAR.T (No match)
1235 - 1243	1096.5143	1095.5070	1095.5383	-29	0	K.NTFESVLMR.W (No match)
1235 - 1243	1112.5077	1111.5004	1111.5332	-29	0	K.NTFESVLMR.W Oxidation (M) (No match)

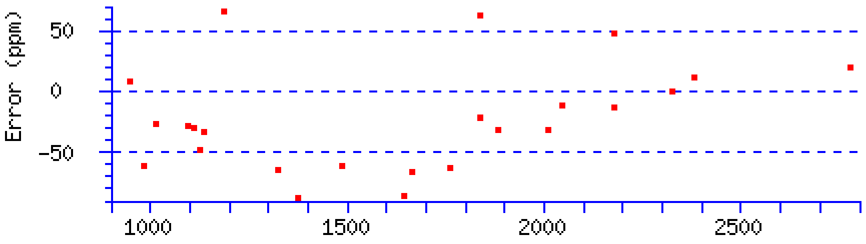

RMS error 47 ppm

```
LOCUS       XP_001790010            1272 aa            linear   MAM 29-JUL-2008
DEFINITION  PREDICTED: similar to endonuclease reverse transcriptase [Bos
            taurus].
ACCESSION   XP_001790010
VERSION     XP_001790010.1  GI:194669270
DBSOURCE    REFSEQ: accession XM_001789958.1
KEYWORDS    .
SOURCE      Bos taurus (cattle)
  ORGANISM  Bos taurus
            Eukaryota; Metazoa; Chordata; Craniata; Vertebrata; Euteleostomi;
            Mammalia; Eutheria; Laurasiatheria; Cetartiodactyla; Ruminantia;
            Pecora; Bovidae; Bovinae; Bos.
COMMENT     MODEL REFSEQ:  This record is predicted by automated computational
            analysis. This record is derived from a genomic sequence
            (NW_001495409) annotated using gene prediction method: GNOMON,
            supported by mRNA evidence.
            Also see:
                Documentation of NCBI's Annotation Process

            COMPLETENESS: full length.
FEATURES             Location/Qualifiers
     source          1..1272
                     /organism="Bos taurus"
                     /db_xref="taxon:9913"
                     /chromosome="8"
                     /breed="Hereford"
     Protein         1..1272
                     /product="similar to endonuclease reverse transcriptase"
                     /calculated_mol_wt=149407
     CDS             1..1272
                     /gene="LOC100140546"
                     /coded_by="XM_001789958.1:1..3819"
                     /db_xref="GeneID:100140546"
```

Mascot: http://www.matrixscience.com/

Spot 28

{MATRIX SCIENCE} Mascot Search Results

Protein View

Match to: **gi|169625443** Score: **178** Expect: **1.6e-011**
hypothetical protein SNOG_15994 [Phaeosphaeria nodorum SN15]

Nominal mass (M_r): **27112**; Calculated pI value: **6.14**
NCBI BLAST search of gi|169625443 against nr
Unformatted sequence string for pasting into other applications

Taxonomy: Phaeosphaeria nodorum SN15
Links to retrieve other entries containing this sequence from NCBI Entrez:
gi|111055453 from Phaeosphaeria nodorum SN15

Fixed modifications: Carbamidomethyl (C)
Variable modifications: Oxidation (M)
Cleavage by Trypsin: cuts C-term side of KR unless next residue is P
Sequence Coverage: **19%**

Matched peptides shown in **Bold Red**

```
  1 MARQFFVGGN FKMNGTIKSI KEILGHLSQA KLDPNTEVVV APPALYLLLA
 51 REHLRPGLEV AAQNIFDKPS GAFTGEISAD QLKDSGITWT ILGHSERRTI
101 LNEDDAFVAS KTKAALDCGL GVILCCGESL EQREANKTIE VVTKQLKAVA
151 DKVKDWSKIV VAYEPIWAIG TGKVATTEQA QEVHKAIREW LQKEVSAEAA
201 EKTRILYGGS VSEKNCNELA KQPDIDGFLV GGASLKPAFV DIINAKQA
```

Show predicted peptides also

Sort Peptides By ◉ Residue Number ○ Increasing Mass ○ Decreasing Mass

Start - End	Observed	Mr(expt)	Mr(calc)	ppm	Miss	Sequence
4 - 12	1043.5372	1042.5299	1042.5236	6	0	R.QFFVGGNFK.M (Ions score 74)
4 - 12	1043.5372	1042.5299	1042.5236	6	0	R.QFFVGGNFK.M (No match)
84 - 97	1571.8010	1570.7937	1570.7740	13	0	K.DSGITWTILGHSER.R (Ions score 79)
84 - 97	1571.8010	1570.7937	1570.7740	13	0	K.DSGITWTILGHSER.R (No match)
84 - 98	1727.8900	1726.8827	1726.8751	4	1	K.DSGITWTILGHSERR.T (No match)
159 - 173	1616.9110	1615.9037	1615.8974	4	0	K.IVVAYEPIWAIGTGK.V (No match)
205 - 214	1052.5699	1051.5626	1051.5550	7	0	R.ILYGGSVSEK.N (No match)

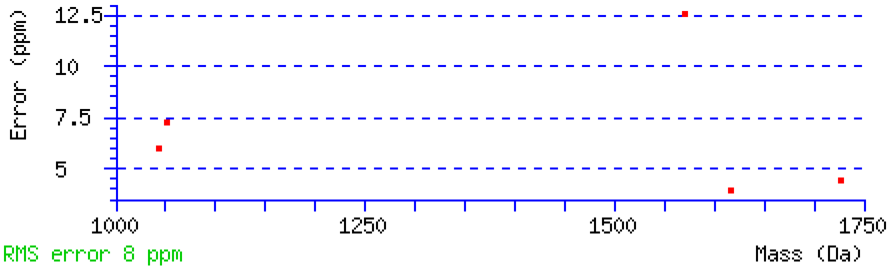


```
LOCUS       XP_001806125             248 aa            linear   PLN 02-APR-2008
DEFINITION  hypothetical protein SNOG_15994 [Phaeosphaeria nodorum SN15].
ACCESSION   XP_001806125
VERSION     XP_001806125.1  GI:169625443
DBSOURCE    REFSEQ: accession XM_001806073.1
KEYWORDS    .
SOURCE      Phaeosphaeria nodorum SN15
  ORGANISM  Phaeosphaeria nodorum SN15
            Eukaryota; Fungi; Dikarya; Ascomycota; Saccharomyceta;
            Pezizomycotina; Leotiomyceta; Dothideomyceta; Dothideomycetes;
            Pleosporomycetidae; Pleosporales; Pleosporineae; Phaeosphaeriaceae;
            Phaeosphaeria.
REFERENCE   1  (residues 1 to 248)
  AUTHORS   Birren,B., Lander,E., Galagan,J., Devon,K., Nusbaum,C., Jaffe,D.,
            Butler,J., Alvarez,P., Gnerre,S., Grabherr,M., Kleber,M.,
            Mauceli,E., Brockman,W., Rounsley,S., Young,S., LaButti,K.,
            Pushparaj,V., DeCaprio,D., Crawford,M., Koehrsen,M., Engels,R.,
            Montgomery,P., Pearson,M., Howarth,C., Kodira,C., Zeng,Q.,
            Yandava,C., Alvarado,L., Oleary,S., Oliver,R.O. and Solomon,P.
  CONSRTM   The Broad Institute Genome Sequencing Platform
  TITLE     Annotation of the Phaeosphaeria nodorum SN15 genome
  JOURNAL   Unpublished
REFERENCE   2  (residues 1 to 248)
  AUTHORS   Lander,E. and Birren,B.
  CONSRTM   The Genome Sequencing Platform, The Genome Assembly Team
  TITLE     Direct Submission
  JOURNAL   Submitted (05-MAR-2008) Broad Institute of MIT and Harvard, 320
            Charles Street, Cambridge, MA 02141, USA
  REMARK    Direct Submission
REFERENCE   3  (residues 1 to 248)
  AUTHORS   Oliver,R. and Solomon,P.
  TITLE     Direct Submission
  JOURNAL   Submitted (05-MAR-2008) Murdoch University, South Street, Perth, WA
            6150, Australia
  REMARK    Direct Submission
```

```
COMMENT     PROVISIONAL REFSEQ: This record has not yet been subject to final
            NCBI review. The reference sequence was derived from EAT76573.
            Method: conceptual translation.
FEATURES             Location/Qualifiers
     source          1..248
                     /organism="Phaeosphaeria nodorum SN15"
                     /db_xref="taxon:321614"
     Protein         1..248
                     /product="hypothetical protein"
                     /calculated_mol_wt=26770
     Region          5..244
                     /region_name="TIM"
                     /note="Triosephosphate isomerase (TIM) is a glycolytic
                     enzyme that catalyzes the interconversion of
                     dihydroxyacetone phosphate and
                     D-glyceraldehyde-3-phosphate. The reaction is very
                     efficient and requires neither cofactors nor metal ions.
                     TIM, usually...; cd00311"
                     /db_xref="CDD:73362"
     Site            order(10,12,94,164,170,210,229,231..232)
                     /site_type="other"
                     /note="substrate binding site"
                     /db_xref="CDD:73362"
     Site            order(10,13,44..46,48,51,63,81,84..85,96..97)
                     /site_type="other"
                     /note="dimer interface"
                     /db_xref="CDD:73362"
     Site            order(12,94,164)
                     /site_type="other"
                     /note="catalytic triad"
                     /db_xref="CDD:73362"
     CDS             1..248
                     /locus_tag="SNOG_15994"
                     /coded_by="XM_001806073.1:64..810"
                     /inference="ab initio prediction:Unveil:1.0"
                     /inference="similar to RNA sequence, EST (same
                     species):INSD:DR045396.1"
                     /inference="similar to RNA sequence, EST (same
                     species):INSD:DR045883.1"
                     /inference="similar to RNA sequence, EST (same
                     species):INSD:EH391712.1"
                     /inference="similar to RNA sequence, EST (same
                     species):INSD:EH395067.1"
                     /inference="similar to RNA sequence, EST (same
                     species):INSD:EH395816.1"
                     /inference="similar to RNA sequence, EST (same
                     species):INSD:EH396096.1"
                     /inference="similar to RNA sequence, EST (same
                     species):INSD:EH396292.1"
```

```
/inference="similar to RNA sequence, EST (same
species):INSD:EH396491.1"
/inference="similar to RNA sequence, EST (same
species):INSD:EH396642.1"
/inference="similar to RNA sequence, EST (same
species):INSD:EH397193.1"
/inference="similar to RNA sequence, EST (same
species):INSD:EH397293.1"
/inference="similar to RNA sequence, EST (same
species):INSD:EH397591.1"
/inference="similar to RNA sequence, EST (same
species):INSD:EH397667.1"
/inference="similar to RNA sequence, EST (same
species):INSD:EH399506.1"
/inference="similar to RNA sequence, EST (same
species):INSD:EH399520.1"
/inference="similar to RNA sequence, EST (same
species):INSD:EH399782.1"
/inference="similar to RNA sequence, EST (same
species):INSD:EH399798.1"
/inference="similar to RNA sequence, EST (same
species):INSD:EH400091.1"
/inference="similar to RNA sequence, EST (same
species):INSD:EH400187.1"
/inference="similar to RNA sequence, EST (same
species):INSD:EH400270.1"
/inference="similar to RNA sequence, EST (same
species):INSD:EH401081.1"
/note="gene prediction version 2"
/db_xref="GeneID:5983055"
```

Mascot: http://www.matrixscience.com/

Spot 29

Mascot Search Results

Protein View

Match to: **gi|255711238** Score: **73** Expect: **0.035**
KLTH0B02596p [Lachancea thermotolerans]

Nominal mass (M_r): **16303**; Calculated pI value: **5.43**
NCBI BLAST search of gi|255711238 against nr
Unformatted sequence string for pasting into other applications

Taxonomy: Lachancea thermotolerans CBS 6340
Links to retrieve other entries containing this sequence from NCBI Entrez:
gi|238933280 from Lachancea thermotolerans

Fixed modifications: Carbamidomethyl (C)
Variable modifications: Oxidation (M)
Cleavage by Trypsin: cuts C-term side of KR unless next residue is P
Sequence Coverage: **70%**

Matched peptides shown in **Bold Red**

```
  1 MSILVVDMDE VRSLAQRHSI EMGLERLRVV GQYVGSWWDA GRLRLEFGNV
 51 SGLASAGGRA VLDASESALE RVRRARAGLV EGAVCDVRCC VMGRDVEVME
101 LRVLTLREVQ ALGEFLAGDA GREFMSLSSG ALQHAGHDRR DDEDRAR
```

Show predicted peptides also

Sort Peptides By ● Residue Number ○ Increasing Mass ○ Decreasing Mass

Start - End	Observed	Mr(expt)	Mr(calc)	ppm	Miss	Sequence
1 - 12	1438.7418	1437.7345	1437.6844	35	0	-.MSILVVDMDEVR.S 2 Oxidation (M) (No match)
1 - 17	1993.9860	1992.9787	1992.9972	-9	1	-.MSILVVDMDEVRSLAQR.H 2 Oxidation (M) (No match)
13 - 26	1626.8844	1625.8771	1625.8307	29	1	R.SLAQRHSIEMGLER.L (No match)
18 - 28	1340.6871	1339.6798	1339.7030	-17	1	R.HSIEMGLERLR.V (No match)
29 - 44	1848.8961	1847.8888	1847.9431	-29	1	R.VVGQYVGSWWDAGRLR.L (No match)
45 - 59	1434.7738	1433.7665	1433.7263	28	0	R.LEFGNVSGLASAGGR.A (No match)
45 - 59	1434.7738	1433.7665	1433.7263	28	0	R.LEFGNVSGLASAGGR.A (No match)
95 - 102	1006.4774	1005.4701	1005.4801	-10	0	R.DVEVMELR.V Oxidation (M) (No match)
103 - 122	2115.0432	2114.0359	2114.1484	-53	1	R.VLTLREVQALGEFLAGDAGR.E (No match)
103 - 122	2115.0432	2114.0359	2114.1484	-53	1	R.VLTLREVQALGEFLAGDAGR.E (No match)
123 - 139	1858.9049	1857.8976	1857.8428	30	0	R.EFMSLSSGALQHAGHDR.R Oxidation (M) (No match)

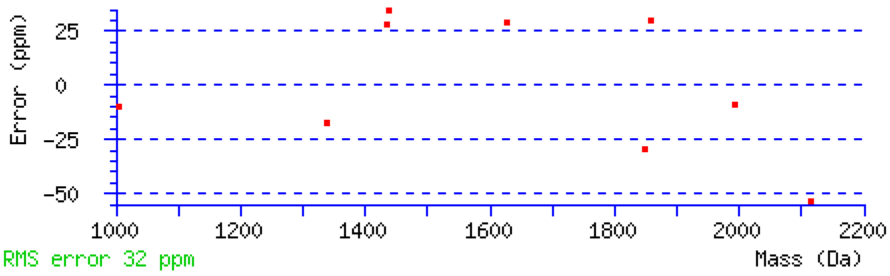

```
LOCUS       XP_002551902             147 aa            linear   PLN 11-AUG-2009
DEFINITION  KLTH0B02596p [Lachancea thermotolerans].
ACCESSION   XP_002551902
VERSION     XP_002551902.1  GI:255711238
DBSOURCE    REFSEQ: accession XM_002551856.1
KEYWORDS    .
SOURCE      Lachancea thermotolerans CBS 6340 (Kluyveromyces thermotolerans CBS
            6340)
  ORGANISM  Lachancea thermotolerans CBS 6340
            Eukaryota; Fungi; Dikarya; Ascomycota; Saccharomyceta;
            Saccharomycotina; Saccharomycetes; Saccharomycetales;
            Saccharomycetaceae; Lachancea.
REFERENCE   1  (residues 1 to 147)
  CONSRTM   The Genolevures Consortium
  TITLE     Comparative genomics of protoploid Saccharomycetaceae
  JOURNAL   Unpublished
REFERENCE   2  (residues 1 to 147)
  AUTHORS   Genoscope -,C.E.A.
  TITLE     Direct Submission
  JOURNAL   Submitted (04-JUN-2009) Genoscope - Centre National de Sequencage :
            BP 191 91006 EVRY cedex - FRANCE (E-mail : seqref@genoscope.cns.fr
            - Web : www.genoscope.cns.fr)
COMMENT     PROVISIONAL REFSEQ: This record has not yet been subject to final
            NCBI review. The reference sequence is identical to CAR21464.
FEATURES             Location/Qualifiers
     source          1..147
                     /organism="Lachancea thermotolerans CBS 6340"
                     /strain="CBS 6340"
                     /db_xref="taxon:559295"
                     /chromosome="B"
     Protein         1..147
                     /product="KLTH0B02596p"
                     /calculated_mol_wt=16011
     CDS             1..147
                     /locus_tag="KLTH0B02596g"
```

```
/old_locus_tag="KLTH-ORF15654"
/coded_by="XM_002551856.1:1..444"
/note="conserved hypothetical protein"
/db_xref="GeneID:8290733"
```

Mascot: http://www.matrixscience.com/

Spot 31

Mascot Search Results

Protein View

Match to: **gi|67903236** Score: **168** Expect: **1.6e-010**
hypothetical protein AN8605.2 [Aspergillus nidulans FGSC A4]

Nominal mass (M_r): **17787**; Calculated pI value: **8.86**
NCBI BLAST search of gi|67903236 against nr
Unformatted sequence string for pasting into other applications

Taxonomy: Aspergillus nidulans FGSC A4
Links to retrieve other entries containing this sequence from NCBI Entrez:
gi|40741449 from Aspergillus nidulans FGSC A4
gi|259483180 from Aspergillus nidulans FGSC A4

Fixed modifications: Carbamidomethyl (C)
Variable modifications: Oxidation (M)
Cleavage by Trypsin: cuts C-term side of KR unless next residue is P
Sequence Coverage: **35%**

Matched peptides shown in **Bold Red**

```
  1 MSNVFFDITA NGEPLGRVEF KLFDDVVPKT ARNFRELATG QHGFGYKGSP
 51 FHRVIPQFML QGGDFTRQNG TGGKSIYGEK FEDENFTLKH DRPYLLSMAN
101 AGRNTNGSQF FITTVKTSWL DGAHVVFGEV VKGQEVVDAV EKLGSQSGAT
151 KKKVVISNSG TL
```

Show predicted peptides also

Sort Peptides By ( ) Residue Number ( ) Increasing Mass ( ) Decreasing Mass

Start - End	Observed	Mr(expt)	Mr(calc)	ppm	Miss	Sequence
1 - 21	2371.0581	2370.0508	2370.1678	-49	1	-.MSNVFFDITANGEPLGRVEFK.L (No match)
18 - 29	1435.7448	1434.7375	1434.7759	-27	1	R.VEFKLFDDVVPK.T (Ions score 74)
18 - 29	1435.7448	1434.7375	1434.7759	-27	1	R.VEFKLFDDVVPK.T (No match)
54 - 67	1608.8221	1607.8148	1607.8130	1	0	R.VIPQFMLQGGDFTR.Q (Ions score 58)
54 - 67	1608.8221	1607.8148	1607.8130	1	0	R.VIPQFMLQGGDFTR.Q (No match)
54 - 67	1624.8145	1623.8072	1623.8079	-0	0	R.VIPQFMLQGGDFTR.Q Oxidation (M) (Ions score 58)
54 - 67	1624.8145	1623.8072	1623.8079	-0	0	R.VIPQFMLQGGDFTR.Q Oxidation (M) (No match)
75 - 89	1819.8599	1818.8526	1818.8676	-8	1	K.SIYGEKFEDENFTLK.H (No match)
81 - 89	1142.5856	1141.5783	1141.5291	43	0	K.FEDENFTLK.H (No match)

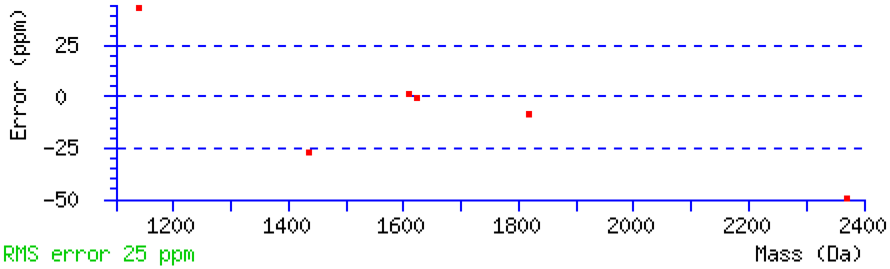


```
LOCUS       XP_681874                162 aa            linear   PLN 09-APR-2008
DEFINITION  hypothetical protein AN8605.2 [Aspergillus nidulans FGSC A4].
ACCESSION   XP_681874
VERSION     XP_681874.1  GI:67903236
DBSOURCE    REFSEQ: accession XM_676782.1
KEYWORDS    .
SOURCE      Aspergillus nidulans FGSC A4
  ORGANISM  Aspergillus nidulans FGSC A4
            Eukaryota; Fungi; Dikarya; Ascomycota; Saccharomyceta;
            Pezizomycotina; Leotiomyceta; Eurotiomycetes; Eurotiomycetidae;
            Eurotiales; Trichocomaceae; Emericella.
REFERENCE   1  (residues 1 to 162)
  AUTHORS   Galagan,J.E., Calvo,S.E., Cuomo,C., Ma,L.J., Wortman,J.R.,
            Batzoglou,S., Lee,S.I., Basturkmen,M., Spevak,C.C., Clutterbuck,J.,
            Kapitonov,V., Jurka,J., Scazzocchio,C., Farman,M., Butler,J.,
            Purcell,S., Harris,S., Braus,G.H., Draht,O., Busch,S., D'Enfert,C.,
            Bouchier,C., Goldman,G.H., Bell-Pedersen,D., Griffiths-Jones,S.,
            Doonan,J.H., Yu,J., Vienken,K., Pain,A., Freitag,M., Selker,E.U.,
            Archer,D.B., Penalva,M.A., Oakley,B.R., Momany,M., Tanaka,T.,
            Kumagai,T., Asai,K., Machida,M., Nierman,W.C., Denning,D.W.,
            Caddick,M., Hynes,M., Paoletti,M., Fischer,R., Miller,B., Dyer,P.,
            Sachs,M.S., Osmani,S.A. and Birren,B.W.
  TITLE     Sequencing of Aspergillus nidulans and comparative analysis with A.
            fumigatus and A. oryzae
  JOURNAL   Nature 438 (7071), 1105-1115 (2005)
   PUBMED   16372000
REFERENCE   2  (residues 1 to 162)
  AUTHORS   Birren,B., Nusbaum,C., Abebe,A., Abouelleil,A., Adekoya,E.,
            Ait-zahra,M., Allen,N., Allen,T., An,P., Anderson,M., Anderson,S.,
            Arachchi,H., Armbruster,J., Bachantsang,P., Baldwin,J., Barry,A.,
            Bayul,T., Blitshsteyn,B., Bloom,T., Blye,J., Boguslavskiy,L.,
            Borowsky,M., Boukhgalter,B., Brunache,A., Butler,J., Calixte,N.,
            Calvo,S., Camarata,J., Campo,K., Chang,J., Cheshatsang,Y.,
            Citroen,M., Collymore,A., Considine,T., Cook,A., Cooke,P.,
            Corum,B., Cuomo,C., David,R., Dawoe,T., Degray,S., Dodge,S.,
```

```
            Dooley,K., Dorje,P., Dorjee,K., Dorris,L., Duffey,N., Dupes,A.,
            Elkins,T., Engels,R., Erickson,J., Farina,A., Faro,S., Ferreira,P.,
            Fischer,H., Fitzgerald,M., Foley,K., Gage,D., Galagan,J.,
            Gearin,G., Gnerre,S., Gnirke,A., Goyette,A., Graham,J.,
            Grandbois,E., Gyaltsen,K., Hafez,N., Hagopian,D., Hagos,B.,
            Hall,J., Hatcher,B., Heller,A., Higgins,H., Honan,T., Horn,A.,
            Houde,N., Hughes,L., Hulme,W., Husby,E., Iliev,I., Jaffe,D.,
            Jones,C., Kamal,M., Kamat,A., Kamvysselis,M., Karlsson,E.,
            Kells,C., Kieu,A., Kisner,P., Kodira,C., Kulbokas,E., Labutti,K.,
            Lama,D., Landers,T., Leger,J., Levine,S., Lewis,D., Lewis,T.,
            Lindblad-toh,K., Liu,X., Lokyitsang,T., Lokyitsang,Y., Lucien,O.,
            Lui,A., Ma,L.J., Mabbitt,R., Macdonald,J., Maclean,C., Major,J.,
            Manning,J., Marabella,R., Maru,K., Matthews,C., Mauceli,E.,
            Mccarthy,M., Mcdonough,S., Mcghee,T., Meldrim,J., Meneus,L.,
            Mesirov,J., Mihalev,A., Mihova,T., Mikkelsen,T., Mlenga,V.,
            Moru,K., Mozes,J., Mulrain,L., Munson,G., Naylor,J., Newes,C.,
            Nguyen,C., Nguyen,N., Nguyen,T., Nicol,R., Nielsen,C., Nizzari,M.,
            Norbu,C., Norbu,N., O'donnell,P., Okoawo,O., O'leary,S.,
            Omotosho,B., O'neill,K., Osman,S., Parker,S., Perrin,D.,
            Phunkhang,P., Piqani,B., Purcell,S., Rachupka,T., Ramasamy,U.,
            Rameau,R., Ray,V., Raymond,C., Retta,R., Richardson,S., Rise,C.,
            Rodriguez,J., Rogers,J., Rogov,P., Rutman,M., Schupbach,R.,
            Seaman,C., Settipalli,S., Sharpe,T., Sheridan,J., Sherpa,N.,
            Shi,J., Smirnov,S., Smith,C., Sougnez,C., Spencer,B., Stalker,J.,
            Stange-thomann,N., Stavropoulos,S., Stetson,K., Stone,C., Stone,S.,
            Stubbs,M., Talamas,J., Tchuinga,P., Tenzing,P., Tesfaye,S.,
            Theodore,J., Thoulutsang,Y., Topham,K., Towey,S., Tsamla,T.,
            Tsomo,N., Vallee,D., Vassiliev,H., Venkataraman,V., Vinson,J.,
            Vo,A., Wade,C., Wang,S., Wangchuk,T., Wangdi,T., Whittaker,C.,
            Wilkinson,J., Wu,Y., Wyman,D., Yadav,S., Yang,S., Yang,X.,
            Yeager,S., Yee,E., Young,G., Zainoun,J., Zembeck,L., Zimmer,A.,
            Zody,M. and Lander,E.
  TITLE     Direct Submission
  JOURNAL   Submitted (26-APR-2004) Whitehead Institute/MIT Center for Genome
            Research, 320 Charles Street, Cambridge, MA 02142, USA
COMMENT     PROVISIONAL REFSEQ: This record has not yet been subject to final
            NCBI review. The reference sequence was derived from EAA60639.
            Method: conceptual translation.
FEATURES             Location/Qualifiers
     source          1..162
                     /organism="Aspergillus nidulans FGSC A4"
                     /strain="FGSC A4"
                     /db_xref="taxon:227321"
                     /chromosome="III"
     Protein         1..162
                     /product="hypothetical protein"
                     /calculated_mol_wt=17667
     Region          2..160
                     /region_name="cyclophilin_ABH_like"
```

```
                     /note="cyclophilin_ABH_like: Cyclophilin  A, B and H-like
                     cyclophilin-type peptidylprolyl cis- trans isomerase
                     (PPIase) domain. This family represents the archetypal
                     cystolic cyclophilin similar to human cyclophilins A, B
                     and H. PPIase is an enzyme which...; cd01926"
                     /db_xref="CDD:29397"
     Site            order(52..53,58,109,111,119)
                     /site_type="active"
                     /db_xref="CDD:29397"
     CDS             1..162
                     /locus_tag="AN8605.2"
                     /coded_by="XM_676782.1:1..489"
                     /db_xref="GeneID:2868486"
```

Mascot: http://www.matrixscience.com/

Spot 32

Mascot Search Results

Protein View

Match to: **gi|189196154** Score: **541** Expect: **7.9e-048**
NADP-specific glutamate dehydrogenase [Pyrenophora tritici-repentis Pt-1C-BFP]

Nominal mass (M_r): **49094**; Calculated pI value: **5.88**
NCBI BLAST search of gi|189196154 against nr
Unformatted sequence string for pasting into other applications

Taxonomy: Pyrenophora tritici-repentis Pt-1C-BFP
Links to retrieve other entries containing this sequence from NCBI Entrez:
gi|187980294 from Pyrenophora tritici-repentis Pt-1C-BFP

Fixed modifications: Carbamidomethyl (C)
Variable modifications: Oxidation (M)
Cleavage by Trypsin: cuts C-term side of KR unless next residue is P
Sequence Coverage: **29%**

Matched peptides shown in **Bold Red**

```
  1 MSEPEFEQAR KELVSTLEAS SLFSKNPEYK KALEVVSVPE RIIQFRVVWE
 51 NDKGECQVQK GYRVQFNSAL GPYKGGLRFH PTVNLSILKF LGFEQIFKNA
101 LTGLNMGGGK GGCDFDPKGK SDNEIRKFCV AFMRELNKHI GADTDVPAGD
151 IGVGGREIGY LFGAYRAERN RWEGVLTGKG GSWGGSLIRP EATGYGLVYY
201 VEHMINYASG GKESFAGKRV ALSGSGNVAQ YAALKIIELG GTVISLSDSK
251 GALIAEDDKG FTPEIINQIA ALKLERKALT ALENHNFKYI EGARPWKEVN
301 KVDVALPSAT QNEVSEDEAK ALIESGAKYI AEGSNMGCTQ EAIEVFEAHR
351 REKKGDAIWY APGKAANAGG VAVSGLEMAQ NSQRLSWTAE QVDEKLKGIM
401 KDCFENCLST AKEYFTPAEG EFPSLVGGAN VAGFRKVAAA MHDQGDWW
```

Show predicted peptides also

Sort Peptides By ● Residue Number ○ Increasing Mass ○ Decreasing Mass

Start - End	Observed	Mr(expt)	Mr(calc)	ppm	Miss	Sequence
64 - 74	1223.6421	1222.6348	1222.6346	0	0	R.VQFNSALGPYK.G (Ions score 54)
64 - 74	1223.6421	1222.6348	1222.6346	0	0	R.VQFNSALGPYK.G (No match)
64 - 78	1606.8735	1605.8662	1605.8627	2	1	R.VQFNSALGPYKGGLR.F (No match)
75 - 89	1651.9634	1650.9561	1650.9570	-1	1	K.GGLRFHPTVNLSILK.F (No match)
79 - 89	1268.7367	1267.7294	1267.7289	0	0	R.FHPTVNLSILK.F (Ions score 83)
79 - 89	1268.7367	1267.7294	1267.7289	0	0	R.FHPTVNLSILK.F (No match)
90 - 98	1128.6107	1127.6034	1127.6015	2	0	K.FLGFEQIFK.N (Ions score 60)

90 - 98	1128.6107	1127.6034	1127.6015	2	0	K.FLGFEQIFK.N	(No match)
139 - 156	1706.8507	1705.8434	1705.8384	3	0	K.HIGADTDVPAGDIGVGGR.E	(Ions score 120)
139 - 156	1706.8507	1705.8434	1705.8384	3	0	K.HIGADTDVPAGDIGVGGR.E	(No match)
170 - 179	1159.6249	1158.6176	1158.6145	3	1	R.NRWEGVLTGK.G	(Ions score 58)
170 - 179	1159.6249	1158.6176	1158.6145	3	1	R.NRWEGVLTGK.G	(No match)
180 - 212	3489.6873	3488.6800	3488.6823	-1	0	K.GGSWGGSLIRPEATGYGLVYYVEHMINYASGGK.E	(No match)
180 - 212	3505.6711	3504.6638	3504.6772	-4	0	K.GGSWGGSLIRPEATGYGLVYYVEHMINYASGGK.E	Oxidation (M) (No matc
219 - 235	1704.9366	1703.9293	1703.9318	-1	1	K.RVALSGSGNVAQYAALK.I	(No match)
220 - 235	1548.8418	1547.8345	1547.8307	2	0	R.VALSGSGNVAQYAALK.I	(No match)
365 - 384	1930.9451	1929.9378	1929.9326	3	0	K.AANAGGVAVSGLEMAQNSQR.L	(Ions score 98)
365 - 384	1930.9451	1929.9378	1929.9326	3	0	K.AANAGGVAVSGLEMAQNSQR.L	(No match)
365 - 384	1946.9423	1945.9350	1945.9276	4	0	K.AANAGGVAVSGLEMAQNSQR.L	Oxidation (M) (Ions score 59)
365 - 384	1946.9423	1945.9350	1945.9276	4	0	K.AANAGGVAVSGLEMAQNSQR.L	Oxidation (M) (No match)

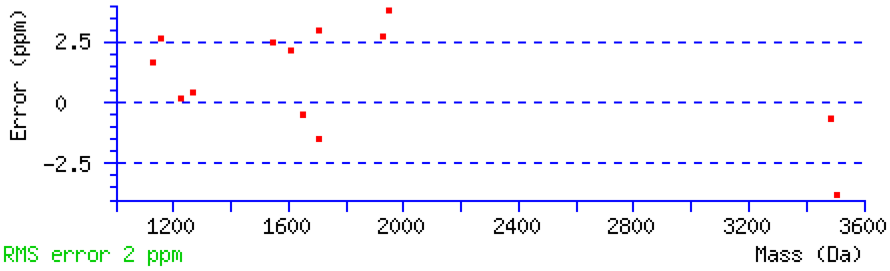


```
LOCUS       XP_001934415             448 aa            linear   PLN 30-MAY-2008
DEFINITION  NADP-specific glutamate dehydrogenase [Pyrenophora tritici-repentis
            Pt-1C-BFP].
ACCESSION   XP_001934415
VERSION     XP_001934415.1  GI:189196154
DBSOURCE    REFSEQ: accession XM_001934380.1
KEYWORDS    .
SOURCE      Pyrenophora tritici-repentis Pt-1C-BFP
  ORGANISM  Pyrenophora tritici-repentis Pt-1C-BFP
            Eukaryota; Fungi; Dikarya; Ascomycota; Saccharomyceta;
            Pezizomycotina; Leotiomyceta; Dothideomyceta; Dothideomycetes;
            Pleosporomycetidae; Pleosporales; Pleosporineae; Pleosporaceae;
            Pyrenophora.
REFERENCE   1  (residues 1 to 448)
  AUTHORS   Birren,B., Lander,E., Galagan,J., Nusbaum,C., Devon,K., Ma,L.-J.,
            Jaffe,D., Butler,J., Alvarez,P., Gnerre,S., Grabherr,M., Kleber,M.,
            Mauceli,E., Brockman,W., MacCallum,I.A., Young,S., LaButti,K.,
            DeCaprio,D., Crawford,M., Koehrsen,M., Engels,R., Montgomery,P.,
            Pearson,M., Howarth,C., Larson,L., White,J., Yandava,C., Kodira,C.,
            Guigo,R., Borodovsky,M., Zeng,Q., O'Leary,S., Alvarado,L.,
            Pandelova,I. and Ciuffetti,L.
  CONSRTM   The Broad Institute Genome Sequencing Platform
```

```
  TITLE     Genome Sequence of Pyrenophora tritici-repentis
  JOURNAL   Unpublished
REFERENCE   2  (residues 1 to 448)
  AUTHORS   Birren,B., Lander,E., Galagan,J., Nusbaum,C., Devon,K., Ma,L.-J.,
            Jaffe,D., Butler,J., Alvarez,P., Gnerre,S., Grabherr,M., Kleber,M.,
            Mauceli,E., Brockman,W., MacCallum,I.A., Young,S., LaButti,K.,
            DeCaprio,D., Crawford,M., Koehrsen,M., Engels,R., Montgomery,P.,
            Pearson,M., Howarth,C., Larson,L., White,J., Yandava,C., Kodira,C.,
            Zeng,Q., O'Leary,S., Alvarado,L., Ciuffetti,L. and Pandelova,I.
  CONSRTM   The Broad Institute Genome Sequencing Platform
  TITLE     Direct Submission
  JOURNAL   Submitted (16-MAR-2007) Broad Institute of MIT and Harvard, 7
            Cambridge Center, Cambridge, MA 02142, USA
COMMENT     PROVISIONAL REFSEQ: This record has not yet been subject to final
            NCBI review. The reference sequence was derived from EDU46920.
            Method: conceptual translation.
FEATURES             Location/Qualifiers
     source          1..448
                     /organism="Pyrenophora tritici-repentis Pt-1C-BFP"
                     /strain="Pt-1C-BFP"
                     /db_xref="taxon:426418"
     Protein         1..448
                     /product="NADP-specific glutamate dehydrogenase"
                     /calculated_mol_wt=48651
     Region          3..445
                     /region_name="PRK09414"
                     /note="glutamate dehydrogenase; Provisional; PRK09414"
                     /db_xref="CDD:169848"
     Region          39..166
                     /region_name="ELFV_dehydrog_N"
                     /note="Glu/Leu/Phe/Val dehydrogenase, dimerization domain;
                     pfam02812"
                     /db_xref="CDD:145786"
     Region          177..446
                     /region_name="NAD_bind_2_Glu_DH"
                     /note="NAD(P) binding domain of glutamate dehydrogenase,
                     subgroup 2; cd05313"
                     /db_xref="CDD:133455"
     Site            order(226..228,248..249,309..310,333..335)
                     /site_type="other"
                     /note="NAD(P) binding site"
                     /db_xref="CDD:133455"
     CDS             1..448
                     /locus_tag="PTRG_04082"
                     /coded_by="XM_001934380.1:1..1347"
                     /db_xref="GeneID:6342318"
```

Mascot: http://www.matrixscience.com/

Spot 33

Mascot Search Results

Protein View

Match to: **gi|145613040** Score: **305** Expect: **3.2e-024**
conserved hypothetical protein [Magnaporthe grisea 70-15]

Nominal mass (M_r): **25180**; Calculated pI value: **5.73**
NCBI BLAST search of gi|145613040 against nr
Unformatted sequence string for pasting into other applications

Taxonomy: Magnaporthe grisea 70-15
Links to retrieve other entries containing this sequence from NCBI Entrez:
gi|145020105 from Magnaporthe grisea 70-15

Fixed modifications: Carbamidomethyl (C)
Variable modifications: Oxidation (M)
Cleavage by Trypsin: cuts C-term side of KR unless next residue is P
Sequence Coverage: **21%**

Matched peptides shown in **Bold Red**

```
  1 MAEEQRPAPL RLGTEAPNFK AETTKGPIDF HEFIGSNWVI LFSHPEDFTP
 51 VCTTELGEFA RLEPEFTKRG VKLIGLSANT VGSHDGWIKD INDVTGSHVA
101 FPIIADKERK VAYLYDMLDY QDTTNVDEKG IAFTIRSVFI IDPAKKIRTI
151 LSYPASTGRN SAEVLRIVDS LQTGDKHKVT TPINWVPGDD VIVHPSIKDE
201 QAKDLFPNFR AVKPYLRFTP LPKE
```

Show predicted peptides also

Sort Peptides By ◉ Residue Number ○ Increasing Mass ○ Decreasing Mass

Start - End	Observed	Mr(expt)	Mr(calc)	ppm	Miss	Sequence
62 - 69	1019.5524	1018.5451	1018.5447	0	1	R.LEPEFTKR.G (Ions score 56)
62 - 69	1019.5524	1018.5451	1018.5447	0	1	R.LEPEFTKR.G (No match)
137 - 145	989.5738	988.5665	988.5593	7	0	R.SVFIIDPAK.K (No match)
149 - 159	1165.6189	1164.6116	1164.6139	-2	0	R.TILSYPASTGR.N (Ions score 95)
149 - 159	1165.6189	1164.6116	1164.6139	-2	0	R.TILSYPASTGR.N (No match)
179 - 198	2187.1760	2186.1687	2186.1736	-2	0	K.VTTPINWVPGDDVIVHPSIK.D (Ions score 133)
179 - 198	2187.1760	2186.1687	2186.1736	-2	0	K.VTTPINWVPGDDVIVHPSIK.D (No match)

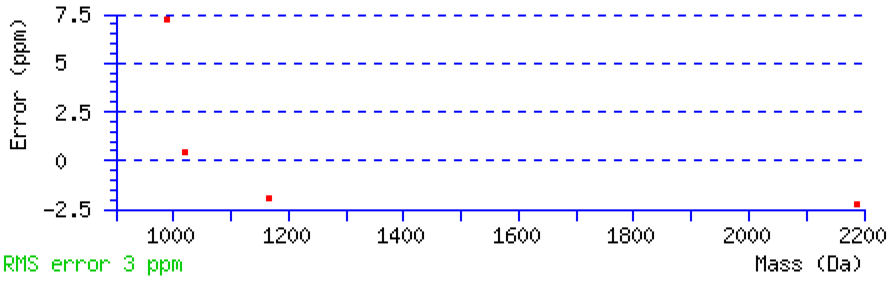

```
LOCUS       XP_362792                224 aa            linear   PLN 17-MAY-2010
DEFINITION  conserved hypothetical protein [Magnaporthe oryzae 70-15].
ACCESSION   XP_362792
VERSION     XP_362792.2  GI:145613040
DBSOURCE    REFSEQ: accession XM_362792.2
KEYWORDS    .
SOURCE      Magnaporthe oryzae 70-15
  ORGANISM  Magnaporthe oryzae 70-15
            Eukaryota; Fungi; Dikarya; Ascomycota; Saccharomyceta;
            Pezizomycotina; Leotiomyceta; Sordariomyceta; Sordariomycetes;
            Sordariomycetidae; Magnaporthales; Magnaporthaceae; Magnaporthe.
REFERENCE   1  (residues 1 to 224)
  AUTHORS   Dean,R.A., Talbot,N.J., Ebbole,D.J., Farman,M.L., Mitchell,T.K.,
            Orbach,M.J., Thon,M., Kulkarni,R., Xu,J.R., Pan,H., Read,N.D.,
            Lee,Y.H., Carbone,I., Brown,D., Oh,Y.Y., Donofrio,N., Jeong,J.S.,
            Soanes,D.M., Djonovic,S., Kolomiets,E., Rehmeyer,C., Li,W.,
            Harding,M., Kim,S., Lebrun,M.H., Bohnert,H., Coughlan,S.,
            Butler,J., Calvo,S., Ma,L.J., Nicol,R., Purcell,S., Nusbaum,C.,
            Galagan,J.E. and Birren,B.W.
  TITLE     The genome sequence of the rice blast fungus Magnaporthe grisea
  JOURNAL   Nature 434 (7036), 980-986 (2005)
   PUBMED   15846337
REFERENCE   2  (residues 1 to 224)
  AUTHORS   Birren,B., Lander,E., Galagan,J., Nusbaum,C., Devon,K., Jaffe,D.,
            Butler,J., Alvarez,P., Gnerre,S., Grabherr,M., Kleber,M.,
            Mauceli,E., Brockman,W., Rounsley,S., Young,S., LaButti,K.,
            Pushparaj,V., DeCaprio,D., Crawford,M., Koehrsen,M., Engels,R.,
            Montgomery,P., Pearson,M., Howarth,C., Kodira,C., Yandava,C.,
            Zeng,Q., Alvarado,L., Oleary,S., Dean,R., Mitchell,T., Brown,D.,
            Pan,H., Thon,M., Zhu,H. and Blackmon,B.
  CONSRTM   The Broad Institute Genome Sequencing Platform
  TITLE     Direct Submission
  JOURNAL   Submitted (26-SEP-2005) Broad Institute of MIT and Harvard, 320
            Charles Street, Cambridge, MA 02142, USA
COMMENT     PROVISIONAL REFSEQ: This record has not yet been subject to final
```

```
            NCBI review. The reference sequence was derived from EDK04333.
            On Apr 23, 2007 this sequence version replaced gi:39946510.
            Method: conceptual translation.
FEATURES             Location/Qualifiers
     source          1..224
                     /organism="Magnaporthe oryzae 70-15"
                     /strain="70-15"
                     /db_xref="taxon:242507"
                     /chromosome="I"
     Protein         1..224
                     /product="hypothetical protein"
                     /calculated_mol_wt=25008
     Region          9..203
                     /region_name="AhpC"
                     /note="Peroxiredoxin [Posttranslational modification,
                     protein turnover, chaperones]; COG0450"
                     /db_xref="CDD:30799"
     Region          12..220
                     /region_name="PRX_1cys"
                     /note="Peroxiredoxin (PRX) family, 1-cys PRX subfamily;
                     composed of PRXs containing only one conserved cysteine,
                     which serves as the peroxidatic cysteine. They are
                     homodimeric thiol-specific antioxidant (TSA) proteins that
                     confer a protective role in cells by...; cd03016"
                     /db_xref="CDD:48565"
     Site            order(12,50,53..55,58,90,93,116,134,148..150,152..154,
                     156..161,163,166,170..171,180..181,183..184,215,218)
                     /site_type="other"
                     /note="dimer interface"
                     /db_xref="CDD:48565"
     Site            order(24,78..80,82..83,85..86,88..89,98,107..108,118,
                     132..133,196,213)
                     /site_type="other"
                     /note="decamer (pentamer of dimers) interface"
                     /db_xref="CDD:48565"
     Site            order(49,52,136)
                     /site_type="other"
                     /note="catalytic triad"
                     /db_xref="CDD:48565"
     CDS             1..224
                     /locus_tag="MGG_08256"
                     /old_locus_tag="MG08256.4"
                     /coded_by="XM_362792.2:1..675"
                     /db_xref="GeneID:2678602"
```

Mascot: http://www.matrixscience.com/

Spot 34

Mascot Search Results

Protein View

Match to: **gi|156057169** Score: **98** Expect: **0.0016**
ATP sulfurylase [Sclerotinia sclerotiorum 1980]

Nominal mass (M_r): **64159**; Calculated pI value: **6.55**
NCBI BLAST search of gi|156057169 against nr
Unformatted sequence string for pasting into other applications

Taxonomy: Sclerotinia sclerotiorum 1980 UF-70
Links to retrieve other entries containing this sequence from NCBI Entrez:
gi|154702101 from Sclerotinia sclerotiorum 1980 UF-70

Fixed modifications: Carbamidomethyl (C)
Variable modifications: Oxidation (M)
Cleavage by Trypsin: cuts C-term side of KR unless next residue is P
Sequence Coverage: **22%**

Matched peptides shown in **Bold Red**

```
  1 MANSPHGGVL KDLLARDLPR HNELSAEAET LPAIVLTERQ LCDLELILSG
 51 GFSPLEGFMN EKDYNGVVEN NRLADGNVFS MPITLDVSKE QIEQLGIKEG
101 VRITIRDFRD DRNLAIINVE DVYKPNKEKE AKEVFGGDVD HPAVKYLYNT
151 AAEFYVGGKI DAINRLEHYD YVALRYTPAE MRLHFDKLGW SKVVAFQTRN
201 PMHRAHRELT VRAARARQAN VLIHPVVGLT KPGDIDHFTR VRVYQALLPR
251 YPNGMAVLGL LPLAMRMGGP REAVWHAIIR KNYGATHFIV GRDHAGPGKN
301 SKGEEFYGPY DAQYAVEKFK DELGIEVVPF QMMTYLPDSD EYRPKDEVPQ
351 GTRTLDISGT ELRSRLRSGR EIPEWFSYPE VVRVLRESHP PRSAQGFTVF
401 LTGYHSSGKD AIARALQTTL NQQGGRSVSL LLGETVRAEL SSELGFSRAD
451 RTRNIGRIAF VASELTRSGA AVIAAPIAPY EDARKHAREM VEKYGDFYLV
501 HVATSLEHSE KIDKKGVYAK ARNGEIKGFT GVDDPYEVPA KADFTVDIEK
551 TSVRNAVHSI ILMLESAGLL DRL
```

Show predicted peptides also

Sort Peptides By ◉ Residue Number ○ Increasing Mass ○ Decreasing Mass

Start - End	Observed	Mr(expt)	Mr(calc)	ppm	Miss	Sequence
2 - 16	1547.7948	1546.7875	1546.8580	-46	1	M.ANSPHGGVLKDLLAR.D (No match)
40 - 62	2597.1714	2596.1641	2596.2553	-35	0	R.QLCDLELILSGGFSPLEGFMNEK.D (No match)
218 - 240	2527.3840	2526.3767	2526.3707	2	0	R.QANVLIHPVVGLTKPGDIDHFTR.V (No match)
241 - 250	1214.7379	1213.7306	1213.7295	1	1	R.VRVYQALLPR.Y (No match)

243 - 250	959.5714	958.5641	958.5600	4	0	R.VYQALLPR.Y	(Ions score 48)
243 - 250	959.5714	958.5641	958.5600	4	0	R.VYQALLPR.Y	(No match)
251 - 266	1731.8242	1730.8169	1730.9211	-60	0	R.YPNGMAVLGLLPLAMR.M	Oxidation (M) (No match)
251 - 266	1747.9231	1746.9158	1746.9161	-0	0	R.YPNGMAVLGLLPLAMR.M	2 Oxidation (M) (No match)
251 - 266	1747.9231	1746.9158	1746.9161	-0	0	R.YPNGMAVLGLLPLAMR.M	2 Oxidation (M) (No match)
272 - 280	1094.6117	1093.6044	1093.6032	1	0	R.EAVWHAIIR.K	(No match)
272 - 281	1222.7073	1221.7000	1221.6982	1	1	R.EAVWHAIIRK.N	(No match)
354 - 363	1104.5975	1103.5902	1103.5822	7	0	R.TLDISGTELR.S	(No match)
427 - 437	1173.6858	1172.6785	1172.6765	2	0	R.SVSLLLGETVR.A	(No match)
458 - 467	1106.5741	1105.5668	1105.6131	-42	0	R.IAFVASELTR.S	(No match)

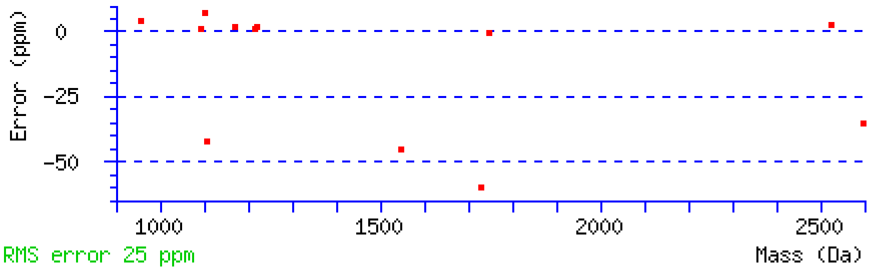


```
LOCUS       XP_001594508             573 aa            linear   PLN 26-FEB-2008
DEFINITION  ATP sulfurylase [Sclerotinia sclerotiorum 1980].
ACCESSION   XP_001594508
VERSION     XP_001594508.1  GI:156057169
DBSOURCE    REFSEQ: accession XM_001594458.1
KEYWORDS    .
SOURCE      Sclerotinia sclerotiorum 1980 UF-70
  ORGANISM  Sclerotinia sclerotiorum 1980 UF-70
            Eukaryota; Fungi; Dikarya; Ascomycota; Saccharomyceta;
            Pezizomycotina; Leotiomyceta; Sordariomyceta; Leotiomycetes;
            Helotiales; Sclerotiniaceae; Sclerotinia.
REFERENCE   1  (residues 1 to 573)
  AUTHORS   Birren,B., Galagan,J., Lander,E., Devon,K., Nusbaum,C., Cuomo,C.,
            Jaffe,D., Butler,J., Alvarez,P., Gnerre,S., Grabherr,M., Kleber,M.,
            Mauceli,E., Brockman,W., Rounsley,S., Young,S., LaButti,K.,
            Pushparaj,V., DeCaprio,D., Crawford,M., Koehrsen,M., Engels,R.,
            Montgomery,P., Pearson,M., Howarth,C., Yandava,C., Kodira,C.,
            Zeng,Q., Alvarado,L., O'Leary,S., Dickman,M.B., Kohn,L. and
            Rollins,J.
  CONSRTM   The Broad Institute Genome Sequencing Platform
  TITLE     Annotation of the Sclerotinia sclerotiorum 1980 genome
  JOURNAL   Unpublished
REFERENCE   2  (residues 1 to 573)
  AUTHORS   Lander,E., Birren,B. and Cuomo,C.
  CONSRTM   The Genome Sequencing Platform, The Genome Assembly Team
```

```
  TITLE     Direct Submission
  JOURNAL   Submitted (10-JUN-2005) Broad Institute of MIT and Harvard, 320
            Charles Street, Cambridge, MA 02142, USA
COMMENT     PROVISIONAL REFSEQ: This record has not yet been subject to final
            NCBI review. The reference sequence was derived from EDO01840.
            Method: conceptual translation.
FEATURES             Location/Qualifiers
     source          1..573
                     /organism="Sclerotinia sclerotiorum 1980 UF-70"
                     /strain="1980"
                     /db_xref="taxon:665079"
     Protein         1..573
                     /product="ATP sulfurylase"
                     /calculated_mol_wt=64011
     Region          4..572
                     /region_name="PRK05537"
                     /note="bifunctional sulfate adenylyltransferase subunit
                     1/adenylylsulfate kinase protein; Validated; PRK05537"
                     /db_xref="CDD:168090"
     Region          31..388
                     /region_name="ATPS"
                     /note="ATP-sulfurylase; cd00517"
                     /db_xref="CDD:173895"
     Site            order(196..200,206,291..292,294..295,332..333)
                     /site_type="active"
                     /db_xref="CDD:173895"
     Site            228..238
                     /site_type="other"
                     /note="flexible loop"
                     /db_xref="CDD:173895"
     Region          398..547
                     /region_name="APSK"
                     /note="Adenosine 5'-phosphosulfate kinase (APSK) catalyzes
                     the phosphorylation of adenosine 5'-phosphosulfate to form
                     3'-phosphoadenosine 5'-phosphosulfate (PAPS). The
                     end-product PAPS is a biologically 'activated' sulfate
                     form important for the assimilation...; cd02027"
                     /db_xref="CDD:30200"
     Site            order(405..406,408..411,437,446,451,454,477..479,512,
                     529..530)
                     /site_type="other"
                     /note="ligand-binding site"
                     /db_xref="CDD:30200"
     CDS             1..573
                     /locus_tag="SS1G_04315"
                     /coded_by="XM_001594458.1:1..1722"
                     /db_xref="GeneID:5490580"
```

**Mascot:** http://www.matrixscience.com/

Spot 35

Mascot Search Results

Protein View

Match to: **gi|169596136** Score: **271** Expect: **7.9e-021**
hypothetical protein SNOG_00819 [Phaeosphaeria nodorum SN15]

Nominal mass (M_r): **58359**; Calculated pI value: **5.71**
NCBI BLAST search of gi|169596136 against nr
Unformatted sequence string for pasting into other applications

Taxonomy: Phaeosphaeria nodorum SN15
Links to retrieve other entries containing this sequence from NCBI Entrez:
gi|111071194 from Phaeosphaeria nodorum SN15

Fixed modifications: Carbamidomethyl (C)
Variable modifications: Oxidation (M)
Cleavage by Trypsin: cuts C-term side of KR unless next residue is P
Sequence Coverage: **28%**

Matched peptides shown in **Bold Red**

```
  1 MANALDHLNA GRTRIEWLSQ LNTEYHPAKE YRRTSIIGTI GPKTNSAEKM
 51 NALRRAGLNV VRMNFSHGSY EYHQSVIDNA REAEKQQSGR PLAIALDTKG
101 PEIRTGNTVG DADIPIKAGT ELNITTDDAY ATKCDDKNMY VDYKNITKVI
151 EVGRTIYVDD GVLSFEVLEV VDDQTLRVKC VNNGKISSKK GVNLPKTDID
201 LPPLSEKDKA DLKFGVKNKV DMVFASFIRR GSDITAIREV LGEEGKDIQI
251 IAKVENQQGV NNFDDILKET DGVMVARGDL GIEIPPSQVF IAQKMMITKC
301 NIAGKPVICA TQMLESMTYN PRPTRAEVSD VGNAVLDGAD CVMLSGETAK
351 GDYPVEAVTM MHETCLLAEV AIPYVNAFDE LRKLAPVPCP TTETCAMAAV
401 SASLEQNAGA ILVLTTSGTT ARLVSKYRPV CPIIMVTRNA SASRYSHLYR
451 GVYPFYFAEE KPDFKAAPWQ EDVDRRLKWG IMNAIKLGVL EKGAPVICVQ
501 GWRGGMGHTN TLRIVPATED LGLDQEA
```

Show predicted peptides also

Sort Peptides By ◉ Residue Number ○ Increasing Mass ○ Decreasing Mass

Start - End	Observed	Mr(expt)	Mr(calc)	ppm	Miss	Sequence	
33 - 43	1142.6462	1141.6389	1141.6819	-38	1	R.RTSIIGTIGPK.T	(Ions score 7)
33 - 43	1142.6462	1141.6389	1141.6819	-38	1	R.RTSIIGTIGPK.T	(No match)
34 - 43	986.5948	985.5875	985.5808	7	0	R.TSIIGTIGPK.T	(Ions score 28)
34 - 43	986.5948	985.5875	985.5808	7	0	R.TSIIGTIGPK.T	(No match)
105 - 133	2965.4426	2964.4353	2964.4564	-7	1	R.TGNTVGDADIPIKAGTELNITTDDAYATK.C	(No match)

105 - 133	2965.4426	2964.4353	2964.4564	-7	1	R.TGNTVGDADIPIKAGTELNITTDDAYATK.C	(No match)
191 - 207	1835.9216	1834.9143	1835.0040	-49	1	K.GVNLPKTDIDLPPLSEK.D	(No match)
191 - 207	1835.9216	1834.9143	1835.0040	-49	1	K.GVNLPKTDIDLPPLSEK.D	(No match)
220 - 230	1356.6699	1355.6626	1355.7020	-29	1	K.VDMVFASFIRR.G	Oxidation (M) (No match)
220 - 230	1356.6699	1355.6626	1355.7020	-29	1	K.VDMVFASFIRR.G	Oxidation (M) (No match)
278 - 294	1811.9882	1810.9809	1810.9829	-1	0	R.GDLGIEIPPSQVFIAQK.M	(Ions score 62)
278 - 294	1811.9882	1810.9809	1810.9829	-1	0	R.GDLGIEIPPSQVFIAQK.M	(No match)
326 - 350	2508.1599	2507.1526	2507.1520	0	0	R.AEVSDVGNAVLDGADCVMLSGETAK.G	(No match)
326 - 350	2508.1599	2507.1526	2507.1520	0	0	R.AEVSDVGNAVLDGADCVMLSGETAK.G	(No match)
427 - 438	1504.8096	1503.8023	1503.8054	-2	0	K.YRPVCPIIMVTR.N	(Ions score 40)
427 - 438	1504.8096	1503.8023	1503.8054	-2	0	K.YRPVCPIIMVTR.N	(No match)
427 - 438	1520.8043	1519.7970	1519.8003	-2	0	K.YRPVCPIIMVTR.N	Oxidation (M) (Ions score 12)
427 - 438	1520.8043	1519.7970	1519.8003	-2	0	K.YRPVCPIIMVTR.N	Oxidation (M) (No match)
427 - 444	2107.0947	2106.0874	2106.0826	2	1	K.YRPVCPIIMVTRNASASR.Y	Oxidation (M) (No match)
427 - 444	2107.0947	2106.0874	2106.0826	2	1	K.YRPVCPIIMVTRNASASR.Y	Oxidation (M) (No match)
477 - 486	1173.6812	1172.6739	1172.6740	-0	1	R.LKWGIMNAIK.L	(No match)
477 - 486	1173.6812	1172.6739	1172.6740	-0	1	R.LKWGIMNAIK.L	(No match)
479 - 486	932.5044	931.4971	931.4949	2	0	K.WGIMNAIK.L	(Ions score 45)
479 - 486	932.5044	931.4971	931.4949	2	0	K.WGIMNAIK.L	(No match)
479 - 486	948.4987	947.4914	947.4899	2	0	K.WGIMNAIK.L	Oxidation (M) (Ions score 28)
479 - 486	948.4987	947.4914	947.4899	2	0	K.WGIMNAIK.L	Oxidation (M) (No match)
504 - 513	1043.5105	1042.5032	1042.4978	5	0	R.GGMGHTNTLR.I	(Ions score 11)
504 - 513	1043.5105	1042.5032	1042.4978	5	0	R.GGMGHTNTLR.I	(No match)
504 - 513	1059.5015	1058.4942	1058.4927	1	0	R.GGMGHTNTLR.I	Oxidation (M) (Ions score 21)
504 - 513	1059.5015	1058.4942	1058.4927	1	0	R.GGMGHTNTLR.I	Oxidation (M) (No match)

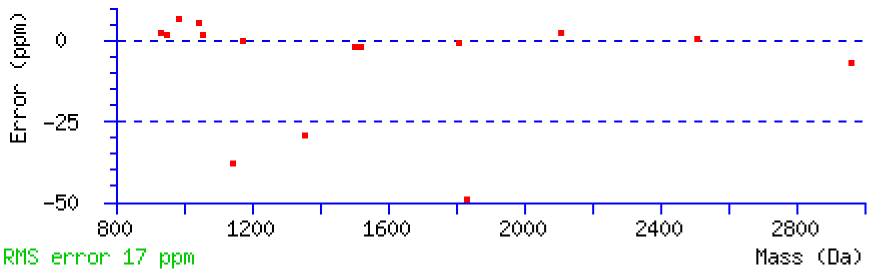


```
LOCUS       XP_001791492             527 aa            linear   PLN 02-APR-2008
DEFINITION  hypothetical protein SNOG_00819 [Phaeosphaeria nodorum SN15].
ACCESSION   XP_001791492
VERSION     XP_001791492.1  GI:169596136
DBSOURCE    REFSEQ: accession XM_001791440.1
KEYWORDS    .
SOURCE      Phaeosphaeria nodorum SN15
  ORGANISM  Phaeosphaeria nodorum SN15
            Eukaryota; Fungi; Dikarya; Ascomycota; Saccharomyceta;
            Pezizomycotina; Leotiomyceta; Dothideomyceta; Dothideomycetes;
```

```
            Pleosporomycetidae; Pleosporales; Pleosporineae; Phaeosphaeriaceae;
            Phaeosphaeria.
REFERENCE   1  (residues 1 to 527)
  AUTHORS   Birren,B., Lander,E., Galagan,J., Devon,K., Nusbaum,C., Jaffe,D.,
            Butler,J., Alvarez,P., Gnerre,S., Grabherr,M., Kleber,M.,
            Mauceli,E., Brockman,W., Rounsley,S., Young,S., LaButti,K.,
            Pushparaj,V., DeCaprio,D., Crawford,M., Koehrsen,M., Engels,R.,
            Montgomery,P., Pearson,M., Howarth,C., Kodira,C., Zeng,Q.,
            Yandava,C., Alvarado,L., Oleary,S., Oliver,R.O. and Solomon,P.
  CONSRTM   The Broad Institute Genome Sequencing Platform
  TITLE     Annotation of the Phaeosphaeria nodorum SN15 genome
  JOURNAL   Unpublished
REFERENCE   2  (residues 1 to 527)
  AUTHORS   Lander,E. and Birren,B.
  CONSRTM   The Genome Sequencing Platform, The Genome Assembly Team
  TITLE     Direct Submission
  JOURNAL   Submitted (05-MAR-2008) Broad Institute of MIT and Harvard, 320
            Charles Street, Cambridge, MA 02141, USA
  REMARK    Direct Submission
REFERENCE   3  (residues 1 to 527)
  AUTHORS   Oliver,R. and Solomon,P.
  TITLE     Direct Submission
  JOURNAL   Submitted (05-MAR-2008) Murdoch University, South Street, Perth, WA
            6150, Australia
  REMARK    Direct Submission
COMMENT     PROVISIONAL REFSEQ: This record has not yet been subject to final
            NCBI review. The reference sequence was derived from EAT92314.
            Method: conceptual translation.
FEATURES             Location/Qualifiers
     source          1..527
                     /organism="Phaeosphaeria nodorum SN15"
                     /db_xref="taxon:321614"
     Protein         1..527
                     /product="hypothetical protein"
                     /calculated_mol_wt=57694
     Region          31..517
                     /region_name="Pyruvate_Kinase"
                     /note="Pyruvate kinase (PK):  Large allosteric enzyme that
                     regulates glycolysis through binding of the substrate,
                     phosphoenolpyruvate, and one or more allosteric effectors.
                     Like other allosteric enzymes, PK has a high substrate
                     affinity R state and a low...; cd00288"
                     /db_xref="CDD:29370"
     Region          32..514
                     /region_name="pyruv_kin"
                     /note="pyruvate kinase; TIGR01064"
                     /db_xref="CDD:162184"
     Site            order(33..34,91,93,101..104,192..193,199,202..206,221,229,
                     236,247,283,301..302,306,338,340,369,371..372,426,428,430,
```

```
                     441,444..445,447,449..451)
                     /site_type="other"
                     /note="domain interfaces"
                     /db_xref="CDD:29370"
     Site            order(62,64,97,227,253,255,279,311)
                     /site_type="active"
                     /db_xref="CDD:29370"
     CDS             1..527
                     /locus_tag="SNOG_00819"
                     /coded_by="XM_001791440.1:1..1584"
                     /inference="ab initio prediction:Unveil:1.0"
                     /inference="similar to RNA sequence, EST (same
                     species):INSD:EH389286.1"
                     /inference="similar to RNA sequence, EST (same
                     species):INSD:EH389713.1"
                     /inference="similar to RNA sequence, EST (same
                     species):INSD:EH390034.1"
                     /inference="similar to RNA sequence, EST (same
                     species):INSD:EH390088.1"
                     /note="gene prediction version 2"
                     /db_xref="GeneID:5967717"
```

Mascot: http://www.matrixscience.com/

Spot 36

{MATRIX SCIENCE} Mascot Search Results

Protein View

Match to: **gi|145239481** Score: **136** Expect: **2.5e-007**
hypothetical protein An08g02700 [Aspergillus niger]

Nominal mass (M_r): **42273**; Calculated pI value: **5.70**
NCBI BLAST search of gi|145239481 against nr
Unformatted sequence string for pasting into other applications

Taxonomy: Aspergillus niger CBS 513.88
Links to retrieve other entries containing this sequence from NCBI Entrez:
gi|134076898 from Aspergillus niger

Fixed modifications: Carbamidomethyl (C)
Variable modifications: Oxidation (M)
Cleavage by Trypsin: cuts C-term side of KR unless next residue is P
Sequence Coverage: **18%**

Matched peptides shown in **Bold Red**

```
  1 MGSVANGSTF LFTSESVGEG HPDKIADQVS DAILDACLAE DPLSKVACET
 51 ATKTGMVMVF GEITTQAKLD YQKVIRGAIQ DIGYDASEKG FDYKTCNVLV
101 AIEQQSPDIA QGLHYEEALE KLGAGDQGIM FGYATDETPE LLPLTVVLSH
151 KLNKAMTDAR KNGTIPWLRP DTKTQVTIEY AHDNGAVKPL RVDTIVISAQ
201 HSDDVTTEEL RKVLKEQIIK SVIPANLLDD RTVYHLQPSG RFVIGGPQGD
251 AGLTGRKIIV DTYGGWGAHG GGAFSGKDYS KVDRSAAYVA RWIAKSLINA
301 GLARRALVQL SYAIGVAEPL SIFVETYGTS SKTSDELVQI IRNNFDLRPG
351 VIVKELDLAK PIYFKTAKNG HFTNQEFSWE KPKALKF
```

Show predicted peptides also

Sort Peptides By ◉ Residue Number ○ Increasing Mass ○ Decreasing Mass

Start - End	Observed	Mr(expt)	Mr(calc)	ppm	Miss	Sequence
161 - 173	1525.7782	1524.7709	1524.8412	-46	1	R.KNGTIPWLRPDTK.T (Ions score 50)
161 - 173	1525.7782	1524.7709	1524.8412	-46	1	R.KNGTIPWLRPDTK.T (No match)
221 - 231	1212.6659	1211.6586	1211.6510	6	0	K.SVIPANLLDDR.T (No match)
257 - 277	2078.0459	2077.0386	2077.0381	0	1	R.KIIVDTYGGWGAHGGGAFSGK.D (Ions score 50)
257 - 277	2078.0459	2077.0386	2077.0381	0	1	R.KIIVDTYGGWGAHGGGAFSGK.D (No match)
258 - 277	1949.9519	1948.9446	1948.9432	1	0	K.IIVDTYGGWGAHGGGAFSGK.D (No match)
258 - 277	1949.9519	1948.9446	1948.9432	1	0	K.IIVDTYGGWGAHGGGAFSGK.D (No match)
258 - 281	2443.1670	2442.1597	2442.1604	-0	1	K.IIVDTYGGWGAHGGGAFSGKDYSK.V (No match)

333 - 354 2526.3167 2525.3094 2525.3966 -35 1 K.TSDELVQIIRNNFDLRPGVIVK.E (No match)

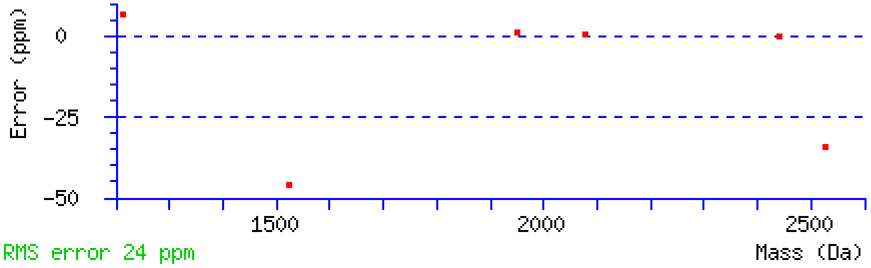

RMS error 24 ppm

```
LOCUS       XP_001392387             387 aa            linear   PLN 28-MAR-2008
DEFINITION  hypothetical protein An08g02700 [Aspergillus niger].
ACCESSION   XP_001392387
VERSION     XP_001392387.1  GI:145239481
DBSOURCE    REFSEQ: accession XM_001392350.1
KEYWORDS    .
SOURCE      Aspergillus niger CBS 513.88
  ORGANISM  Aspergillus niger CBS 513.88
            Eukaryota; Fungi; Dikarya; Ascomycota; Saccharomyceta;
            Pezizomycotina; Leotiomyceta; Eurotiomycetes; Eurotiomycetidae;
            Eurotiales; Trichocomaceae; mitosporic Trichocomaceae; Aspergillus.
REFERENCE   1  (residues 1 to 387)
  AUTHORS   Pel,H.J., de Winde,J.H., Archer,D.B., Dyer,P.S., Hofmann,G.,
            Schaap,P.J., Turner,G., de Vries,R.P., Albang,R., Albermann,K.,
            Andersen,M.R., Bendtsen,J.D., Benen,J.A., van den Berg,M.,
            Breestraat,S., Caddick,M.X., Contreras,R., Cornell,M.,
            Coutinho,P.M., Danchin,E.G., Debets,A.J., Dekker,P., van
            Dijck,P.W., van Dijk,A., Dijkhuizen,L., Driessen,A.J., d'Enfert,C.,
            Geysens,S., Goosen,C., Groot,G.S., de Groot,P.W., Guillemette,T.,
            Henrissat,B., Herweijer,M., van den Hombergh,J.P., van den
            Hondel,C.A., van der Heijden,R.T., van der Kaaij,R.M., Klis,F.M.,
            Kools,H.J., Kubicek,C.P., van Kuyk,P.A., Lauber,J., Lu,X., van der
            Maarel,M.J., Meulenberg,R., Menke,H., Mortimer,M.A., Nielsen,J.,
            Oliver,S.G., Olsthoorn,M., Pal,K., van Peij,N.N., Ram,A.F.,
            Rinas,U., Roubos,J.A., Sagt,C.M., Schmoll,M., Sun,J., Ussery,D.,
            Varga,J., Vervecken,W., van de Vondervoort,P.J., Wedler,H.,
            Wosten,H.A., Zeng,A.P., van Ooyen,A.J., Visser,J. and Stam,H.
  TITLE     Genome sequencing and analysis of the versatile cell factory
            Aspergillus niger CBS 513.88
  JOURNAL   Nat. Biotechnol. 25 (2), 221-231 (2007)
   PUBMED   17259976
COMMENT     PROVISIONAL REFSEQ: This record has not yet been subject to final
            NCBI review. The reference sequence was derived from CAK45307.
FEATURES             Location/Qualifiers
```

```
source          1..387
                /organism="Aspergillus niger CBS 513.88"
                /db_xref="taxon:425011"
                /clone="An08"
Protein         1..387
                /product="hypothetical protein"
                /calculated_mol_wt=41997
Region          9..386
                /region_name="PTZ00104"
                /note="S-adenosylmethionine synthetase; Provisional;
                PTZ00104"
                /db_xref="CDD:173396"
Region          10..107
                /region_name="S-AdoMet_synt_N"
                /note="S-adenosylmethionine synthetase, N-terminal domain;
                pfam00438"
                /db_xref="CDD:144142"
Region          120..242
                /region_name="S-AdoMet_synt_M"
                /note="S-adenosylmethionine synthetase, central domain;
                pfam02772"
                /db_xref="CDD:145757"
Region          244..381
                /region_name="S-AdoMet_synt_C"
                /note="S-adenosylmethionine synthetase, C-terminal domain;
                pfam02773"
                /db_xref="CDD:111646"
CDS             1..387
                /locus_tag="An08g02700"
                /coded_by="XM_001392350.1:1..1164"
                /inference="profile:COGS:COG0192"
                /inference="profile:PFAM:PF00438"
                /inference="profile:PFAM:PF02772"
                /inference="profile:PFAM:PF02773"
                /inference="similar to AA sequence:PIR:S65800"
                /note="Catalytic activity: methionine adenosyltransferases
                convert ATP and L-methionine and H(2)O to phosphate and
                diphosphate and S-adenosyl-L-methionine.;
                Function: adenosylmethionine is an important methyl donor
                in most transmethylation reactions and is also the
                propylamino donor in the biosynthesis of polyamines.;
                Phenotype: muations of the human adenosylmethionine can
                result in an autosomal recessive disorder, the
                hypermethioninemia.;
                Title: strong similarity to S-adenosylmethionine synthase
                eth-1 - Neurospora crassa"
                /citation=[PUBMED 8849888]
                /db_xref="GOA:A2QQJ1"
                /db_xref="GeneID:4982584"
```

Mascot: http://www.matrixscience.com/

Spot 37

Mascot Search Results

Protein View

Match to: **gi|148361511** Score: **115** Expect: **3.2e-005**
vacuolar serine protease [Cladosporium cladosporioides]

Nominal mass (M_r): **41006**; Calculated pI value: **5.80**
NCBI BLAST search of gi|148361511 against nr
Unformatted sequence string for pasting into other applications

Taxonomy: Cladosporium cladosporioides

Fixed modifications: Carbamidomethyl (C)
Variable modifications: Oxidation (M)
Cleavage by Trypsin: cuts C-term side of KR unless next residue is P
Sequence Coverage: **20%**

Matched peptides shown in **Bold Red**

```
  1 DQEVHVLKSE SEVEKNAPWG LARISHRDSL SFGTFNKYLY TEDGGEGVDV
 51 YVVDTGTNVD HVDFEGRASW GKTIPQGDAD EDGNGHGTHC SGTVAGKKYG
101 VAKKAHVKAV KVLRSNGSGS MSDVVKGVEY AAESHLEQVS IAKKGKRKGF
151 KGSTANMSLG GGKSPILDKA VNAAVDAGIH FAVAAGNDNA DSCNYSPAAA
201 ENAVTVGAST LSDERAYFSN YGKCNDIFAP GLNIQSTWIG SKYAVNTISG
251 TSMASPHVAG LLAYLLSLQP AKDSAFAVAD ISPKKLKANL ISIATVGALT
301 DVPSNTANIL AWNGGGESNY SAIVEKGGYK AAVRPTMLEE IESEAKVASK
351 KVYSEGDELA KKVAELTEKI EDLISGDLKD MFRDLKRE
```

Show predicted peptides also

Sort Peptides By ◉ Residue Number ○ Increasing Mass ○ Decreasing Mass

Start - End	Observed	Mr(expt)	Mr(calc)	ppm	Miss	Sequence
16 - 23	884.4792	883.4719	883.4664	6	0	K.NAPWGLAR.I (Ions score 57)
16 - 23	884.4792	883.4719	883.4664	6	0	K.NAPWGLAR.I (No match)
73 - 97	2481.0664	2480.0591	2480.0623	-1	0	K.TIPQGDADEDGNGHGTHCSGTVAGK.K (Ions score 23)
73 - 97	2481.0664	2480.0591	2480.0623	-1	0	K.TIPQGDADEDGNGHGTHCSGTVAGK.K (No match)
73 - 98	2609.1775	2608.1702	2608.1572	5	1	K.TIPQGDADEDGNGHGTHCSGTVAGKK.Y (No match)
273 - 285	1348.7119	1347.7046	1347.7034	1	1	K.DSAFAVADISPKK.L (No match)
331 - 346	1789.8601	1788.8528	1788.8927	-22	0	K.AAVRPTMLEEIESEAK.V Oxidation (M) (No match)
370 - 379	1102.5500	1101.5427	1101.5917	-44	0	K.IEDLISGDLK.D (No match)
370 - 379	1102.5500	1101.5427	1101.5917	-44	0	K.IEDLISGDLK.D (No match)
380 - 386	940.5173	939.5100	939.4484	66	1	K.DMFRDLK.R Oxidation (M) (No match)

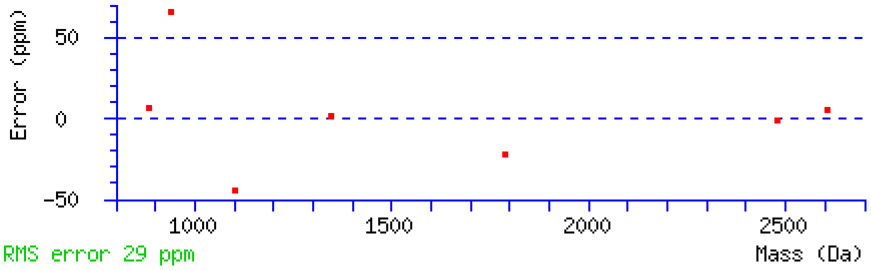


```
LOCUS       ABQ59329                 388 aa            linear   PLN 17-JUL-2009
DEFINITION  vacuolar serine protease [Cladosporium cladosporioides].
ACCESSION   ABQ59329
VERSION     ABQ59329.1  GI:148361511
DBSOURCE    accession EF407520.1
KEYWORDS    .
SOURCE      Cladosporium cladosporioides
  ORGANISM  Cladosporium cladosporioides
            Eukaryota; Fungi; Dikarya; Ascomycota; Saccharomyceta;
            Pezizomycotina; Leotiomyceta; Dothideomyceta; Dothideomycetes;
            Dothideomycetidae; Capnodiales; Davidiellaceae; mitosporic
            Davidiellaceae; Cladosporium.
REFERENCE   1  (residues 1 to 388)
  AUTHORS   Chou,H., Tam,M.F., Lee,L.H., Chiang,C.H., Tai,H.Y., Panzani,R.C.
            and Shen,H.D.
  TITLE     Vacuolar serine protease is a major allergen of Cladosporium
            cladosporioides
  JOURNAL   Int. Arch. Allergy Immunol. 146 (4), 277-286 (2008)
   PUBMED   18362473
REFERENCE   2  (residues 1 to 388)
  AUTHORS   Shen,H.-D. and Lee,L.-H.
  TITLE     Direct Submission
  JOURNAL   Submitted (29-JAN-2007) Medical Research and Education, Taipei
            Veterans General Hospital, No. 201, Shih-pai Road, Section 2,
            Taipei, Taiwan 11217, R.O.C.
FEATURES             Location/Qualifiers
     source          1..388
                     /organism="Cladosporium cladosporioides"
                     /db_xref="taxon:29917"
     Protein         <1..388
                     /product="vacuolar serine protease"
     Region          19..296
                     /region_name="Peptidases_S8_PCSK9_ProteinaseK_like"
                     /note="Peptidase S8 family domain in ProteinaseK-like
                     proteins; cd04077"
```

```
                     /db_xref="CDD:173790"
     Site            order(22,25..26)
                     /site_type="other"
                     /note="calcium binding site 2"
                     /db_xref="CDD:173790"
     Site            order(54,86,159..160,187,249,252)
                     /site_type="active"
                     /db_xref="CDD:173790"
     Site            order(54,86,252)
                     /site_type="other"
                     /note="catalytic triad"
                     /db_xref="CDD:173790"
     Site            order(201,203,226)
                     /site_type="other"
                     /note="calcium binding site 1"
                     /db_xref="CDD:173790"
     CDS             1..388
                     /coded_by="EF407520.1:<1..1167"
```

Mascot: http://www.matrixscience.com/

Spot 38

Mascot Search Results

Protein View

Match to: **gi|85094513** Score: **222** Expect: **6.3e-016**
hypothetical protein NCU02252 [Neurospora crassa OR74A]

Nominal mass (M_r): **57010**; Calculated pI value: **5.36**
NCBI BLAST search of gi|85094513 against nr
Unformatted sequence string for pasting into other applications

Taxonomy: Neurospora crassa OR74A
Links to retrieve other entries containing this sequence from NCBI Entrez:
gi|28921353 from Neurospora crassa OR74A
gi|40804637 from Neurospora crassa

Fixed modifications: Carbamidomethyl (C)
Variable modifications: Oxidation (M)
Cleavage by Trypsin: cuts C-term side of KR unless next residue is P
Sequence Coverage: **17%**

Matched peptides shown in **Bold Red**

```
  1 MAPEHKACLI VIDGWGIPSE ESPKNGDAIA AAETPVMDEL SKSATGFSEL
 51 EASSLAVGLP EGLMGNSEVG HLNIGAGRVV WQDVVRIDQT IKKGELSQNE
101 VIKATFERAK NGNGRLHLCG LVSHGGVHSK QTHLYALLKA AKEAGVPKVF
151 IHFFGDGRDT DPKSGAGYMQ ELLDTIKEIG IGELATVVGR YYAMDRDKRW
201 ERVEVALKGM ILGEGEESTD PVKTIKERYE KGENDEFLKP IVVGGDERRI
251 KEDDTVFFFN YRSDRVRQIT QLMGGVDRSP LPDFPFPNIK LVTMTQYKLD
301 YPFDVAFKPQ QMDNVLAEWL GKQGVKQVHI AETEKYAHVT FFFNGGVEKV
351 FPLETRDESQ DLVPSNKSVA TYDKAPEMSA DGVANQVVKR LGEQEFPFVM
401 NNFAPPDMVG HTGVYEAAIV GCAATDKAIG KILEGCKKEG YILFITSDHG
451 NAEEMKFPDG KPKTSHTTNK VPFIMANAPE GWSLKKEGGV LGDVAPTILA
501 AMGLPQPAEM TGQNLLVKA
```

Show predicted peptides also

Sort Peptides By ◉ Residue Number ○ Increasing Mass ○ Decreasing Mass

Start - End	Observed	Mr(expt)	Mr(calc)	ppm	Miss	Sequence
79 - 86	1000.5567	999.5494	999.5502	-1	0	R.VVWQDVVR.I (Ions score 51)
79 - 86	1000.5567	999.5494	999.5502	-1	0	R.VVWQDVVR.I (No match)
178 - 190	1313.7440	1312.7367	1312.7351	1	0	K.EIGIGELATVVGR.Y (Ions score 76)
178 - 190	1313.7440	1312.7367	1312.7351	1	0	K.EIGIGELATVVGR.Y (No match)

191 - 198	1061.4958	1060.4885	1060.4648	22	1	R.YYAMDRDK.R	(No match)
200 - 208	1129.6368	1128.6295	1128.6291	0	1	R.WERVEVALK.G	(Ions score 17)
200 - 208	1129.6368	1128.6295	1128.6291	0	1	R.WERVEVALK.G	(No match)
250 - 262	1693.8256	1692.8183	1692.8148	2	1	R.IKEDDTVFFFNYR.S	(Ions score 37)
250 - 262	1693.8256	1692.8183	1692.8148	2	1	R.IKEDDTVFFFNYR.S	(No match)
252 - 262	1452.6869	1451.6796	1451.6358	30	0	K.EDDTVFFFNYR.S	(No match)
268 - 278	1217.6276	1216.6203	1216.6234	-3	0	R.QITQLMGGVDR.S	(No match)
268 - 278	1233.6219	1232.6146	1232.6183	-3	0	R.QITQLMGGVDR.S	Oxidation (M) (Ions score 9)
268 - 278	1233.6219	1232.6146	1232.6183	-3	0	R.QITQLMGGVDR.S	Oxidation (M) (No match)
336 - 349	1615.7952	1614.7879	1614.7831	3	0	K.YAHVTFFFNGGVEK.V	(No match)
471 - 486	1803.9244	1802.9171	1802.9389	-12	1	K.VPFIMANAPEGWSLKK.E	Oxidation (M) (No match)
471 - 486	1803.9244	1802.9171	1802.9389	-12	1	K.VPFIMANAPEGWSLKK.E	Oxidation (M) (No match)

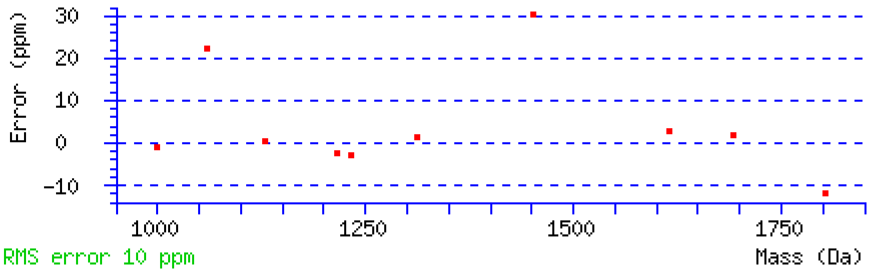


```
LOCUS       XP_959896                519 aa            linear   PLN 10-APR-2008
DEFINITION  hypothetical protein NCU02252 [Neurospora crassa OR74A].
ACCESSION   XP_959896
VERSION     XP_959896.1  GI:85094513
DBSOURCE    REFSEQ: accession XM_954803.2
KEYWORDS    .
SOURCE      Neurospora crassa OR74A
  ORGANISM  Neurospora crassa OR74A
            Eukaryota; Fungi; Dikarya; Ascomycota; Saccharomyceta;
            Pezizomycotina; Leotiomyceta; Sordariomyceta; Sordariomycetes;
            Sordariomycetidae; Sordariales; Sordariaceae; Neurospora.
REFERENCE   1  (residues 1 to 519)
  AUTHORS   Galagan,J.E., Calvo,S.E., Borkovich,K.A., Selker,E.U., Read,N.D.,
            Jaffe,D., FitzHugh,W., Ma,L.J., Smirnov,S., Purcell,S., Rehman,B.,
            Elkins,T., Engels,R., Wang,S., Nielsen,C.B., Butler,J.,
            Endrizzi,M., Qui,D., Ianakiev,P., Bell-Pedersen,D., Nelson,M.A.,
            Werner-Washburne,M., Selitrennikoff,C.P., Kinsey,J.A., Braun,E.L.,
            Zelter,A., Schulte,U., Kothe,G.O., Jedd,G., Mewes,W., Staben,C.,
            Marcotte,E., Greenberg,D., Roy,A., Foley,K., Naylor,J.,
            Stange-Thomann,N., Barrett,R., Gnerre,S., Kamal,M., Kamvysselis,M.,
            Mauceli,E., Bielke,C., Rudd,S., Frishman,D., Krystofova,S.,
            Rasmussen,C., Metzenberg,R.L., Perkins,D.D., Kroken,S., Cogoni,C.,
            Macino,G., Catcheside,D., Li,W., Pratt,R.J., Osmani,S.A.,
```

```
            DeSouza,C.P., Glass,L., Orbach,M.J., Berglund,J.A., Voelker,R.,
            Yarden,O., Plamann,M., Seiler,S., Dunlap,J., Radford,A.,
            Aramayo,R., Natvig,D.O., Alex,L.A., Mannhaupt,G., Ebbole,D.J.,
            Freitag,M., Paulsen,I., Sachs,M.S., Lander,E.S., Nusbaum,C. and
            Birren,B.
  TITLE     The genome sequence of the filamentous fungus Neurospora crassa
  JOURNAL   Nature 422 (6934), 859-868 (2003)
   PUBMED   12712197
REFERENCE   2  (residues 1 to 519)
  AUTHORS   Galagan,J., Henn,M.R., Hood,H., Radford,A., Collins,R.,
            DeCaprio,D., Crawford,M., Koehrsen,M., Engels,R., Montgomery,P.,
            Pearson,M., Howarth,C., Larson,L., White,J., Ledlie,T., Kodira,C.,
            Zeng,Q., Yandava,C., Alvarado,L., O'Leary,S., Bowman,B., Colot,H.,
            Ebbole,D., Rasmussen,C., Baker,C., Kalkman,E., Chen,C.-H., Shi,M.,
            Mathur,R., Lambreghts,R., mehra,A., Collopy,P., Mehra,A.,
            Schweredtfeger,C., Hong,C., Belden,W., Glass,N.L., Borkovich,K.,
            Dunlap,J., Lander,E., Nusbaum,C., Sachs,M. and Birren,B.
  TITLE     Version 3 gene predictions for the Neurospora crassa assembly 7
  JOURNAL   Unpublished
REFERENCE   3  (residues 1 to 519)
  AUTHORS   Birren,B., Galagan,J. and Henn,M.R.
  TITLE     Direct Submission
  JOURNAL   Submitted (06-JUL-2007) Broad Institute of MIT and Harvard, 7
            Cambridge Center, Cambridge, MA 02142, USA
REFERENCE   4  (residues 1 to 519)
  AUTHORS   Birren,B.
  TITLE     Direct Submission
  JOURNAL   Submitted (11-MAR-2003) Whitehead Institute/MIT Center for Genome
            Research, 320 Charles Street, Cambridge, MA 02142, USA
COMMENT     PROVISIONAL REFSEQ: This record has not yet been subject to final
            NCBI review. The reference sequence was derived from EAA30660.
            Method: conceptual translation.
FEATURES             Location/Qualifiers
     source          1..519
                     /organism="Neurospora crassa OR74A"
                     /strain="OR74A"
                     /db_xref="taxon:367110"
                     /chromosome="VII"
     Protein         1..519
                     /product="hypothetical protein"
                     /name="similar to phosphoglycerate mutase"
                     /calculated_mol_wt=56687
     Region          1..519
                     /region_name="Sulfatase"
                     /note="Sulfatase; cl10460"
                     /db_xref="CDD:175217"
     Region          2..518
                     /region_name="GpmI"
                     /note="Phosphoglyceromutase [Carbohydrate transport and
```

```
                     metabolism]; COG0696"
                     /db_xref="CDD:31040"
     CDS             1..519
                     /locus_tag="NCU02252"
                     /old_locus_tag="NCU02252.1"
                     /coded_by="XM_954803.2:116..1675"
                     /db_xref="GeneID:3876059"
```

Mascot: http://www.matrixscience.com/

Spot 39

Mascot Search Results

Protein View

Match to: **gi|145232889** Score: **123** Expect: **5e-006**
hypothetical protein An02g06820 [Aspergillus niger]

Nominal mass (M_r): **62977**; Calculated pI value: **6.29**
NCBI BLAST search of gi|145232889 against nr
Unformatted sequence string for pasting into other applications

Taxonomy: Aspergillus niger CBS 513.88
Links to retrieve other entries containing this sequence from NCBI Entrez:
gi|134056738 from Aspergillus niger

Fixed modifications: Carbamidomethyl (C)
Variable modifications: Oxidation (M)
Cleavage by Trypsin: cuts C-term side of KR unless next residue is P
Sequence Coverage: **18%**

Matched peptides shown in **Bold Red**

```
  1 MATDIATRDL RKPTTVAEYL FRRLHEVGVR SVHGVPGDYN LAALDYLPKC
 51 GLHWVGNCNE LNAGYAADGY ARVNGIGALI TTFGVGELSA LNAIAGSYSE
101 FVPVVHIVGQ PNTKSQKDGM LLHHTLGNGD FNVFAKMSAG ISCTLGRLNE
151 TLEAATLIDN AIRECWIRSR PVYISLPTDM IVKQIEGDRL DKPLDLSLPA
201 NDPEKEDYVV DVVLKYLHAA KKPVILVDAC AIRHRVLDEV HDLMEASGLP
251 TFVAPMGKGA VDETRPNYGG VYAGTGSNAG VREQVESSDL ILSIGAIKSD
301 FNTSGFSYHI GQLNTIDFHS TYVRVRYSEY PEINMKGVLR KVIQRMGAVN
351 AAPVPHLSNT LPESEKSSSS QEITHDWLWP NVGQWLKEND IVITETGTAN
401 FGIWETRFPA NVTAISQVLW GSIGYSVGAC QGAALAAKEL GNRRTVLFVG
451 DGSLQLTVQE LSTMIRNNLN PIIFVICNNG YTIERYIHGW DESYNDIQPW
501 DIEGLPRVFG AKDKYKGYKV KTRDELRQLF ANQEFASAPY LQFTCLVMML
551 PPRXKSRLRR LPPGTSK
```

Show predicted peptides also

Sort Peptides By ◉ Residue Number ○ Increasing Mass ○ Decreasing Mass

Start - End	Observed	Mr(expt)	Mr(calc)	ppm	Miss	Sequence
1 - 11	1278.6725	1277.6652	1277.6398	20	1	-.MATDIATRDLR.K Oxidation (M) (No match)
118 - 136	2086.0256	2085.0183	2085.0102	4	0	K.DGMLLHHTLGNGDFNVFAK.M (No match)
118 - 136	2086.0256	2085.0183	2085.0102	4	0	K.DGMLLHHTLGNGDFNVFAK.M (No match)
118 - 136	2102.0295	2101.0222	2101.0051	8	0	K.DGMLLHHTLGNGDFNVFAK.M Oxidation (M) (No match)

148 - 163	1756.9041	1755.8968	1755.9366	-23	0	R.LNETLEAATLIDNAIR.E	(No match)
169 - 183	1734.9370	1733.9297	1733.9386	-5	0	R.SRPVYISLPTDMIVK.Q	Oxidation (M) (No match)
236 - 258	2488.1782	2487.1709	2487.2026	-13	0	R.VLDEVHDLMEASGLPTFVAPMGK.G	2 Oxidation (M) (No match)
388 - 407	2266.1021	2265.0948	2265.0913	2	0	K.ENDIVITETGTANFGIWETR.F	(Ions score 101)
388 - 407	2266.1021	2265.0948	2265.0913	2	0	K.ENDIVITETGTANFGIWETR.F	(No match)

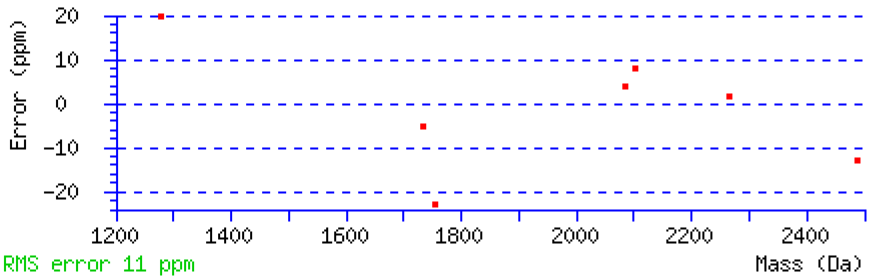

```
LOCUS       XP_001399817             567 aa            linear   PLN 28-FEB-2008
DEFINITION  hypothetical protein An02g06820 [Aspergillus niger].
ACCESSION   XP_001399817
VERSION     XP_001399817.1  GI:145232889
DBSOURCE    REFSEQ: accession XM_001399780.1
KEYWORDS    .
SOURCE      Aspergillus niger CBS 513.88
  ORGANISM  Aspergillus niger CBS 513.88
            Eukaryota; Fungi; Dikarya; Ascomycota; Saccharomyceta;
            Pezizomycotina; Leotiomyceta; Eurotiomycetes; Eurotiomycetidae;
            Eurotiales; Trichocomaceae; mitosporic Trichocomaceae; Aspergillus.
REFERENCE   1  (residues 1 to 567)
  AUTHORS   Pel,H.J., de Winde,J.H., Archer,D.B., Dyer,P.S., Hofmann,G.,
            Schaap,P.J., Turner,G., de Vries,R.P., Albang,R., Albermann,K.,
            Andersen,M.R., Bendtsen,J.D., Benen,J.A., van den Berg,M.,
            Breestraat,S., Caddick,M.X., Contreras,R., Cornell,M.,
            Coutinho,P.M., Danchin,E.G., Debets,A.J., Dekker,P., van
            Dijck,P.W., van Dijk,A., Dijkhuizen,L., Driessen,A.J., d'Enfert,C.,
            Geysens,S., Goosen,C., Groot,G.S., de Groot,P.W., Guillemette,T.,
            Henrissat,B., Herweijer,M., van den Hombergh,J.P., van den
            Hondel,C.A., van der Heijden,R.T., van der Kaaij,R.M., Klis,F.M.,
            Kools,H.J., Kubicek,C.P., van Kuyk,P.A., Lauber,J., Lu,X., van der
            Maarel,M.J., Meulenberg,R., Menke,H., Mortimer,M.A., Nielsen,J.,
            Oliver,S.G., Olsthoorn,M., Pal,K., van Peij,N.N., Ram,A.F.,
            Rinas,U., Roubos,J.A., Sagt,C.M., Schmoll,M., Sun,J., Ussery,D.,
            Varga,J., Vervecken,W., van de Vondervoort,P.J., Wedler,H.,
            Wosten,H.A., Zeng,A.P., van Ooyen,A.J., Visser,J. and Stam,H.
  TITLE     Genome sequencing and analysis of the versatile cell factory
            Aspergillus niger CBS 513.88
  JOURNAL   Nat. Biotechnol. 25 (2), 221-231 (2007)
```

```
   PUBMED   17259976
COMMENT     PROVISIONAL REFSEQ: This record has not yet been subject to final
            NCBI review. The reference sequence was derived from CAK44227.
FEATURES             Location/Qualifiers
     source          1..567
                     /organism="Aspergillus niger CBS 513.88"
                     /db_xref="taxon:425011"
                     /clone="An02"
     Protein         1..567
                     /product="hypothetical protein"
     Region          12..548
                     /region_name="COG3961"
                     /note="Pyruvate decarboxylase and related thiamine
                     pyrophosphate-requiring enzymes [Carbohydrate transport
                     and metabolism / Coenzyme metabolism / General function
                     prediction only]; COG3961"
                     /db_xref="CDD:33742"
     Region          18..178
                     /region_name="TPP_PYR_PDC_IPDC_like"
                     /note="Pyrimidine (PYR) binding domain of pyruvate
                     decarboxylase (PDC), indolepyruvate decarboxylase (IPDC)
                     and related proteins; cd07038"
                     /db_xref="CDD:132921"
     Site            order(36..37,45,48,58..59,85,92,123..124,126,137,140)
                     /site_type="other"
                     /note="dimer interface"
                     /db_xref="CDD:132921"
     Site            order(36..37,45,48,53,56..59,62,65..66,68,70,72..74,85,92,
                     96,100,123..124)
                     /site_type="other"
                     /note="PYR/PP interface"
                     /db_xref="CDD:132921"
     Site            order(36,60,85,124)
                     /site_type="other"
                     /note="TPP binding site"
                     /db_xref="CDD:132921"
     Region          210..>307
                     /region_name="TPP_enzyme_M"
                     /note="Thiamine pyrophosphate enzyme, central domain;
                     pfam00205"
                     /db_xref="CDD:143962"
     Region          373..537
                     /region_name="TPP_PDC_IPDC"
                     /note="TPP-binding module; composed of proteins similar to
                     pyruvate decarboxylase (PDC) and indolepyruvate
                     decarboxylase (IPDC). PDC, a key enzyme in alcoholic
                     fermentation, catalyzes the conversion of pyruvate to
                     acetaldehyde and CO2. It is able to utilize...; cd02005"
                     /db_xref="CDD:48168"
```

```
     Site            order(398,421,423,450..453,478,480..484)
                     /site_type="other"
                     /note="TPP-binding site"
                     /db_xref="CDD:48168"
     Site            order(419..421,455..456,459,462,466,484,488,493..494,498,
                     500..501,508..509)
                     /site_type="other"
                     /note="dimer interface"
                     /db_xref="CDD:48168"
     CDS             1..567
                     /locus_tag="An02g06820"
                     /coded_by="XM_001399780.1:1..1700"
                     /inference="profile:COGS:COG0028"
                     /inference="profile:PFAM:PF00205"
                     /inference="profile:PFAM:PF02775"
                     /inference="profile:PFAM:PF02776"
                     /inference="similar to AA sequence:UniProtKB:AF098293.1"
                     /exception="reasons given in citation"
                     /note="Catalytic activity: a 2-oxo acid = an aldehyde +
                     CO2 ; pyruvate = acetaldehyde + CO2.;
                     Pathway: glycolysis / gluconeogenesis.;
                     Remark: PDC levels are a major determinant of ethanol
                     production.;
                     Similarity: belongs to the thiamine-diphosphate protein
                     family.;
                     Title: strong similarity to pyruvate decarboxylase pdcA -
                     Aspergillus oryzae;
                     putative sequencing error"
                     /citation=[PUBMED 9210590]
                     /db_xref="GeneID:4979172"
```

Mascot: http://www.matrixscience.com/

Spot 40

Mascot Search Results

Protein View

Match to: **gi|116196014** Score: **369** Expect: **1.3e-030**
hypothetical protein CHGG_04605 [Chaetomium globosum CBS 148.51]

Nominal mass (M_r): **68195**; Calculated pI value: **5.57**
NCBI BLAST search of gi|116196014 against nr
Unformatted sequence string for pasting into other applications

Taxonomy: Chaetomium globosum CBS 148.51
Links to retrieve other entries containing this sequence from NCBI Entrez:
gi|88180518 from Chaetomium globosum CBS 148.51

Fixed modifications: Carbamidomethyl (C)
Variable modifications: Oxidation (M)
Cleavage by Trypsin: cuts C-term side of KR unless next residue is P
Sequence Coverage: **20%**

Matched peptides shown in **Bold Red**

```
  1 MMATRLSRAV STTNSAVAIM EGKTPKIIEN AEGARTTPSV VAFAEDGERL
 51 VGVAAKRQAV VNPENTLFAT KRLIGRKFTD AEVQRDIKEV PYKIVQHTNG
101 DAWVESRGQK YSPSQIGGFV LNKMKETAEA YLSKPIKNAV VTVPAYFNDS
151 QRQATKDAGQ ISGLNVLRVV NEPTAAALAY GLEKEADRVV AVYDLGGGTF
201 DISILEIQNG VFEVKSTNGD THLGGEDFDI HLVRHFVQQF KKESGIDLSG
251 DRMAIQRIRE AAEKAKIELS SSLQTDINLP FITADAGGPK HINSKLTRAQ
301 LEAMVDPLIS KTIEPVRKAL KDAGLQAKDI QEVILVGGMT RMPKVSESVK
351 SIFGRDPAKS VNPDEAVAIG AAVQGAVLSG EVKDLLLLDV TPLSLGIETL
401 GGVFTRLINR NTTIPTKKSQ VFSTAADFQT AVEIKVYQGE RELVRDNKLL
451 GNFQLVGIPP AHRGVPQVEV TFDIDADSIV HVHAKDKSTN KDQSITIASG
501 SGLSDAEIQQ MVEESEKYAE SDKERKAVIE TANRADSVVN DTEKALNEHA
551 DKLDKTEADQ IREKITSLRE FVAKAQSGEA TATSAEIKEK TDELQVASLN
601 LFDKMHKARA ESGEPAQNAE GEKKDEPKA
```

Show predicted peptides also

Sort Peptides By ◉ Residue Number ○ Increasing Mass ○ Decreasing Mass

Start - End	Observed	Mr(expt)	Mr(calc)	ppm	Miss	Sequence
58 - 71	1531.7997	1530.7924	1530.8042	-8	0	R.QAVVNPENTLFATK.R (No match)
58 - 72	1687.9081	1686.9008	1686.9053	-3	1	R.QAVVNPENTLFATKR.L (No match)
77 - 85	1093.5629	1092.5556	1092.5564	-1	1	R.KFTDAEVQR.D (No match)

78 - 85	965.4722	964.4649	964.4614	4	0	K.FTDAEVQR.D (No match)
138 - 152	1680.8400	1679.8327	1679.8267	4	0	K.NAVVTVPAYFNDSQR.Q (Ions score 68)
138 - 152	1680.8400	1679.8327	1679.8267	4	0	K.NAVVTVPAYFNDSQR.Q (No match)
157 - 168	1242.6836	1241.6763	1241.6728	3	0	K.DAGQISGLNVLR.V (Ions score 66)
157 - 168	1242.6836	1241.6763	1241.6728	3	0	K.DAGQISGLNVLR.V (No match)
169 - 184	1645.8757	1644.8684	1644.8723	-2	0	R.VVNEPTAAALAYGLEK.E (No match)
329 - 341	1430.7661	1429.7588	1429.7599	-1	0	K.DIQEVILVGGMTR.M (Ions score 91)
329 - 341	1430.7661	1429.7588	1429.7599	-1	0	K.DIQEVILVGGMTR.M (No match)
329 - 341	1446.7612	1445.7539	1445.7548	-1	0	K.DIQEVILVGGMTR.M Oxidation (M) (Ions score 26)
329 - 341	1446.7612	1445.7539	1445.7548	-1	0	K.DIQEVILVGGMTR.M Oxidation (M) (No match)
351 - 359	990.5372	989.5299	989.5294	1	1	K.SIFGRDPAK.S (Ions score 14)
351 - 359	990.5372	989.5299	989.5294	1	1	K.SIFGRDPAK.S (No match)
449 - 463	1631.9414	1630.9341	1630.9307	2	0	K.LLGNFQLVGIPPAHR.G (Ions score 75)
449 - 463	1631.9414	1630.9341	1630.9307	2	0	K.LLGNFQLVGIPPAHR.G (No match)
556 - 564	1089.5336	1088.5263	1088.5462	-18	1	K.TEADQIREK.I (No match)
589 - 604	1849.9631	1848.9558	1848.9469	5	1	K.EKTDELQVASLNLFDK.M (No match)

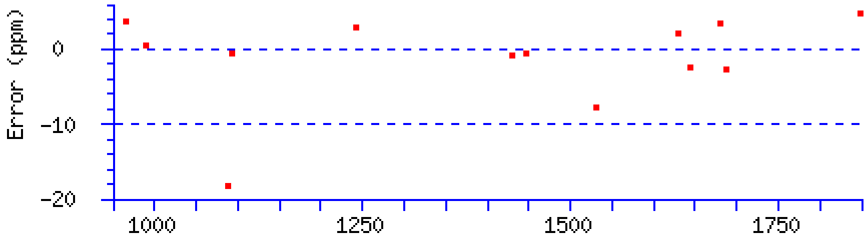

RMS error 5 ppm

```
LOCUS       XP_001223819             629 aa            linear   PLN 09-APR-2008
DEFINITION  hypothetical protein CHGG_04605 [Chaetomium globosum CBS 148.51].
ACCESSION   XP_001223819
VERSION     XP_001223819.1  GI:116196014
DBSOURCE    REFSEQ: accession XM_001223818.1
KEYWORDS    .
SOURCE      Chaetomium globosum CBS 148.51
  ORGANISM  Chaetomium globosum CBS 148.51
            Eukaryota; Fungi; Dikarya; Ascomycota; Saccharomyceta;
            Pezizomycotina; Leotiomyceta; Sordariomyceta; Sordariomycetes;
            Sordariomycetidae; Sordariales; Chaetomiaceae; Chaetomium.
REFERENCE   1  (residues 1 to 629)
  AUTHORS   Birren,B., Lander,E., Galagan,J., Devon,K., Nusbaum,C., Ma,L.-J.,
            Jaffe,D., Butler,J., Alvarez,P., Gnerre,S., Grabherr,M., Kleber,M.,
            Mauceli,E., Brockman,W., Rounsley,S., Young,S., LaButti,K.,
            Pushparaj,V., DeCaprio,D., Crawford,M., Koehrsen,M., Engels,R.,
            Montgomery,P., Pearson,M., Howarth,C., Kodira,C., Yandava,C.,
            Zeng,Q., Alvarado,L., Oleary,S. and Untereiner,W.
  CONSRTM   The Broad Institute Genome Sequencing Platform
```

```
  TITLE     Annotation of the Chaetomium globosum CBS 148.51 Genome
  JOURNAL   Unpublished (2004)
REFERENCE   2  (residues 1 to 629)
  AUTHORS   Lander,E., Birren,B. and Ma,L.
  CONSRTM   The Genome Sequencing Platform, The Genome Assembly Team
  TITLE     Direct Submission
  JOURNAL   Submitted (10-MAR-2005) Broad Institute of MIT and Harvard, 320
            Charles Street, Cambridge, MA 02141, USA
REFERENCE   3  (residues 1 to 629)
  AUTHORS   Untereiner,W.
  TITLE     Direct Submission
  JOURNAL   Submitted (10-MAR-2005) Brandon University, 270-18th, Brandon, MB
            R7A-6A9, Canada
COMMENT     PROVISIONAL REFSEQ: This record has not yet been subject to final
            NCBI review. The reference sequence was derived from EAQ87986.
            Method: conceptual translation.
FEATURES             Location/Qualifiers
     source          1..629
                     /organism="Chaetomium globosum CBS 148.51"
                     /strain="CBS 148.51"
                     /db_xref="taxon:306901"
     Protein         1..629
                     /product="hypothetical protein"
                     /name="similar to heat shock protein 70Kda"
                     /calculated_mol_wt=68105
     Region          12..625
                     /region_name="dnaK"
                     /note="molecular chaperone DnaK; Provisional; PRK00290"
                     /db_xref="CDD:166891"
     CDS             1..629
                     /locus_tag="CHGG_04605"
                     /coded_by="XM_001223818.1:1..1890"
                     /db_xref="GeneID:4392109"
```

Mascot: http://www.matrixscience.com/

Spot 41

Mascot Search Results

Protein View

Match to: **gi|258575103** Score: **335** Expect: **3.2e-027**
3'(2'),5'-bisphosphate nucleotidase [Uncinocarpus reesii 1704]

Nominal mass (M_r): **38061**; Calculated pI value: **5.03**
NCBI BLAST search of gi|258575103 against nr
Unformatted sequence string for pasting into other applications

Taxonomy: Uncinocarpus reesii 1704
Links to retrieve other entries containing this sequence from NCBI Entrez:
gi|237901999 from Uncinocarpus reesii 1704

Fixed modifications: Carbamidomethyl (C)
Variable modifications: Oxidation (M)
Cleavage by Trypsin: cuts C-term side of KR unless next residue is P
Sequence Coverage: **25%**

Matched peptides shown in **Bold Red**

```
  1 MSYQKELLVA QLAVQRASIL TQNVFHEKAK GTLSKDDFSP VTKGDFGAQA
 51 LIIQAIRTNF PEDEIVAEEE ASSLRENDSL RNEMWNLVKD IKLTDDESDR
101 ILGGPLKNET EMLEALDGGK SEGGPKGRIW ALDPIDGTKG FLRGGQYAVC
151 LGLIVDGDVK VGVIGCPNLP LSDSATLSAE IGQSGAADAE TGVLFSAVKG
201 QGATSRPLSQ GALPEGKAIS MRPVTDISQA CFCEGVEAGH SAQDDNAEVA
251 RRLGITSPSV RLDSQAKYCS IARGAGDIYL RLPVKADYQE KIWDHAAGDL
301 LVREAGGDVT DITGKRLDFS IGRKLSGNKG VVAAPKPIFE QVINAVRAVY
351 AAKASI
```

Show predicted peptides also

Sort Peptides By ◉ Residue Number ○ Increasing Mass ○ Decreasing Mass

Start - End	Observed	Mr(expt)	Mr(calc)	ppm	Miss	Sequence
17 - 30	1585.8514	1584.8441	1584.8624	-12	1	R.ASILTQNVFHEKAK.G (No match)
82 - 92	1405.6826	1404.6753	1404.7071	-23	1	R.NEMWNLVKDIK.L Oxidation (M) (No match)
108 - 120	1406.6847	1405.6774	1405.6395	27	0	K.NETEMLEALDGGK.S (No match)
127 - 139	1441.7802	1440.7729	1440.7725	0	1	K.GRIWALDPIDGTK.G (Ions score 43)
127 - 139	1441.7802	1440.7729	1440.7725	0	1	K.GRIWALDPIDGTK.G (No match)
129 - 139	1228.6548	1227.6475	1227.6499	-2	0	R.IWALDPIDGTK.G (Ions score 42)
129 - 139	1228.6548	1227.6475	1227.6499	-2	0	R.IWALDPIDGTK.G (No match)
274 - 281	864.4617	863.4544	863.4501	5	0	R.GAGDIYLR.L (Ions score 38)

274 - 281	864.4617	863.4544	863.4501	5	0	R.GAGDIYLR.L	(No match)
274 - 285	1301.7482	1300.7409	1300.7503	-7	1	R.GAGDIYLRLPVK.A	(No match)
292 - 303	1365.7291	1364.7218	1364.7201	1	0	K.IWDHAAGDLLVR.E	(Ions score 95)
292 - 303	1365.7291	1364.7218	1364.7201	1	0	K.IWDHAAGDLLVR.E	(No match)
316 - 323	963.5363	962.5290	962.5298	-1	1	K.RLDFSIGR.K	(Ions score 56)
316 - 323	963.5363	962.5290	962.5298	-1	1	K.RLDFSIGR.K	(No match)
348 - 356	893.4642	892.4569	892.5018	-50	1	R.AVYAAKASI.-	(No match)

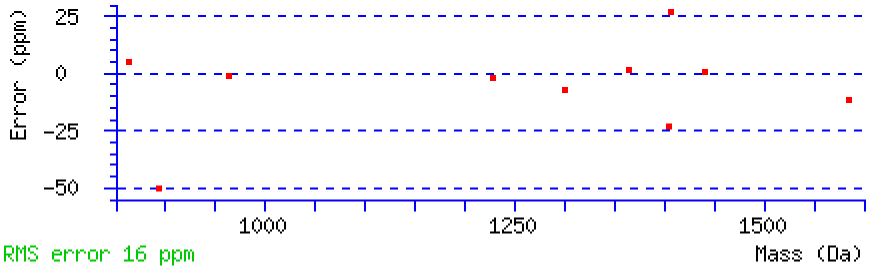

RMS error 16 ppm

```
LOCUS       XP_002541733            356 aa            linear   PLN 16-SEP-2009
DEFINITION  3'(2'),5'-bisphosphate nucleotidase [Uncinocarpus reesii 1704].
ACCESSION   XP_002541733
VERSION     XP_002541733.1  GI:258575103
DBSOURCE    REFSEQ: accession XM_002541687.1
KEYWORDS    .
SOURCE      Uncinocarpus reesii 1704
  ORGANISM  Uncinocarpus reesii 1704
            Eukaryota; Fungi; Dikarya; Ascomycota; Saccharomyceta;
            Pezizomycotina; Leotiomyceta; Eurotiomycetes; Eurotiomycetidae;
            Onygenales; Onygenaceae; Uncinocarpus.
REFERENCE   1  (residues 1 to 356)
  AUTHORS   Birren,B., Lander,E., Galagan,J., Nusbaum,C., Devon,K., Ma,L.-J.,
            Henn,M., Jaffe,D., Butler,J., Alvarez,P., Gnerre,S., Grabherr,M.,
            Kleber,M., Mauceli,E., Brockman,W., Rounsley,S., Young,S.,
            LaButti,K., Pushparaj,V., DeCaprio,D., Crawford,M., Koehrsen,M.,
            Engels,R., Montgomery,P., Pearson,M., Howarth,C., Larson,L.,
            Luoma,S., White,J., O'Leary,S., Kodira,C., Zeng,Q., Yandava,C.,
            Alvarado,L., Taylor,J. and Johannesson,H.
  CONSRTM   The Broad Institute Genome Sequencing Platform
  TITLE     Annotation of the Uncinocarpus reesii strain 1704 genome
  JOURNAL   Unpublished (2004)
REFERENCE   2  (residues 1 to 356)
  AUTHORS   Taylor,J. and Johannesson,H.
  TITLE     Direct Submission
  JOURNAL   Submitted (12-AUG-2005) Department of Plant and Microbial Biology,
            UC-Berkeley, 111 Koshland Hall, Berkeley, CA 94720-3102, USA
REFERENCE   3  (residues 1 to 356)
```

```
  AUTHORS   Lander,E., Birren,B. and Ma,L.
  CONSRTM   The Genome Sequencing Platform, The Genome Assembly Team
  TITLE     Direct Submission
  JOURNAL   Submitted (12-APR-2005) Broad Institute of MIT and Harvard, 320
            Charles Street, Cambridge, MA 02141, USA
COMMENT     PROVISIONAL REFSEQ: This record has not yet been subject to final
            NCBI review. The reference sequence is identical to EEP76400.
            Method: conceptual translation.
FEATURES             Location/Qualifiers
     source          1..356
                     /organism="Uncinocarpus reesii 1704"
                     /strain="1704"
                     /db_xref="taxon:336963"
     Protein         1..356
                     /product="3'(2'),5'-bisphosphate nucleotidase"
                     /calculated_mol_wt=37668
     Region          6..348
                     /region_name="PAP_phosphatase"
                     /note="PAP-phosphatase_like domains. PAP-phosphatase is a
                     member of the inositol monophosphatase family, and
                     catalyses the hydrolysis of
                     3'-phosphoadenosine-5'-phosphate (PAP) to AMP. In
                     Saccharomyces cerevisiae, HAL2 (MET22) is involved in
                     methionine...; cd01517"
                     /db_xref="CDD:73273"
     Site            order(39,45,68..69,133..136,138..139,240,263,293..294)
                     /site_type="active"
                     /db_xref="CDD:73273"
     Site            order(133,294)
                     /site_type="other"
                     /note="putative lithium-binding site"
                     /db_xref="CDD:73273"
     Site            order(240,267,281,290,294)
                     /site_type="other"
                     /note="substrate binding site"
                     /db_xref="CDD:73273"
     CDS             1..356
                     /locus_tag="UREG_01249"
                     /coded_by="XM_002541687.1:1..1071"
                     /db_xref="GeneID:8444609"
```

Mascot: http://www.matrixscience.com/

Spot 42

MATRIX SCIENCE Mascot Search Results

Protein View

Match to: **gi|258577135** Score: **396** Expect: **2.5e-033**
rab GDP dissociation inhibitor alpha [Uncinocarpus reesii 1704]

Nominal mass (M_r): **52040**; Calculated pI value: **5.66**
NCBI BLAST search of gi|258577135 against nr
Unformatted sequence string for pasting into other applications

Taxonomy: Uncinocarpus reesii 1704
Links to retrieve other entries containing this sequence from NCBI Entrez:
gi|237903015 from Uncinocarpus reesii 1704

Fixed modifications: Carbamidomethyl (C)
Variable modifications: Oxidation (M)
Cleavage by Trypsin: cuts C-term side of KR unless next residue is P
Sequence Coverage: **25%**

Matched peptides shown in **Bold Red**

```
  1 MDDIAPEYDV VVLGTGLTEC VLSGVLSVKG QKVLHIDRND HYGAEAASVN
 51 IETLFKKFGN LKPGEEPWKK YGRVNDWNVD LVPKLLMSNG ELTNILVSTD
101 VTRYLEFRQI AGSYVQQGSG SKAMVAKVPS DAGEALRSSL MGLFEKRRAK
151 KFLEWVGEFD EKNPSTHQGL NMANCTMKEV YDKFGLEDST RDFVGHSMAL
201 YQSDGYIGVP GGASETVNRI RLYVNSMARY GKSPYIYPLY GLGELPQGFA
251 RLSAIYGGTY MLNTDVDEIL YENGKVSGIK ATMKEKGEPG PGMRFTTKTK
301 KIIADPSYFP GKVRVTGYLL KAICILNHPI DKTDGSDSLQ LIIPQSQIGR
351 KHDVYIAMVS SAHNVCPKGY YVAIVSTIAE NEANHHLELE PGFQRLGKIE
401 EKFMGPPIPL YEPIDSGVED NIYISKSYDA TSHFETTTDD IRDIYKRAEG
451 HDLVVDGLRE GTTLVGEQ
```

Show predicted peptides also

Sort Peptides By ◉ Residue Number ○ Increasing Mass ○ Decreasing Mass

Start - End	Observed	Mr(expt)	Mr(calc)	ppm	Miss	Sequence
58 - 69	1401.7026	1400.6953	1400.7088	-10	0	K.FGNLKPGEEPWK.K (No match)
85 - 103	2076.1011	2075.0938	2075.0932	0	0	K.LLMSNGELTNILVSTDVTR.Y (Ions score 110)
85 - 103	2076.1011	2075.0938	2075.0932	0	0	K.LLMSNGELTNILVSTDVTR.Y (No match)
85 - 103	2092.0947	2091.0874	2091.0882	-0	0	K.LLMSNGELTNILVSTDVTR.Y Oxidation (M) (Ions score 20)
85 - 103	2092.0947	2091.0874	2091.0882	-0	0	K.LLMSNGELTNILVSTDVTR.Y Oxidation (M) (No match)
104 - 122	2117.9885	2116.9812	2117.0542	-34	1	R.YLEFRQIAGSYVQQGSGSK.A (No match)

128 - 137	1014.5245	1013.5172	1013.5142	3	0	K.VPSDAGEALR.S (Ions score 35)
128 - 137	1014.5245	1013.5172	1013.5142	3	0	K.VPSDAGEALR.S (No match)
179 - 191	1558.7596	1557.7523	1557.7311	14	1	K.EVYDKFGLEDSTR.D (No match)
184 - 191	924.4459	923.4386	923.4349	4	0	K.FGLEDSTR.D (No match)
222 - 229	953.4872	952.4799	952.4800	-0	0	R.LYVNSMAR.Y (Ions score 41)
222 - 229	953.4872	952.4799	952.4800	-0	0	R.LYVNSMAR.Y (No match)
222 - 229	969.4752	968.4679	968.4749	-7	0	R.LYVNSMAR.Y Oxidation (M) (No match)
233 - 251	2141.1133	2140.1060	2140.0993	3	0	K.SPYIYPLYGLGELPQGFAR.L (Ions score 113)
233 - 251	2141.1133	2140.1060	2140.0993	3	0	K.SPYIYPLYGLGELPQGFAR.L (No match)
427 - 446	2378.0828	2377.0755	2377.0710	2	1	K.SYDATSHFETTTDDIRDIYK.R (Ions score 54)
427 - 446	2378.0828	2377.0755	2377.0710	2	1	K.SYDATSHFETTTDDIRDIYK.R (No match)

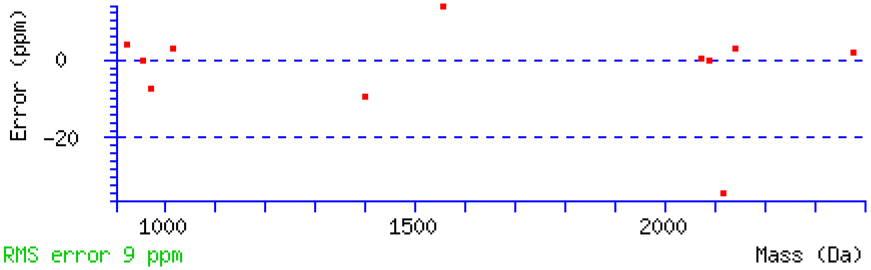

```
LOCUS       XP_002542749             468 aa            linear   PLN 16-SEP-2009
DEFINITION  rab GDP dissociation inhibitor alpha [Uncinocarpus reesii 1704].
ACCESSION   XP_002542749
VERSION     XP_002542749.1  GI:258577135
DBSOURCE    REFSEQ: accession XM_002542703.1
KEYWORDS    .
SOURCE      Uncinocarpus reesii 1704
  ORGANISM  Uncinocarpus reesii 1704
            Eukaryota; Fungi; Dikarya; Ascomycota; Saccharomyceta;
            Pezizomycotina; Leotiomyceta; Eurotiomycetes; Eurotiomycetidae;
            Onygenales; Onygenaceae; Uncinocarpus.
REFERENCE   1  (residues 1 to 468)
  AUTHORS   Birren,B., Lander,E., Galagan,J., Nusbaum,C., Devon,K., Ma,L.-J.,
            Henn,M., Jaffe,D., Butler,J., Alvarez,P., Gnerre,S., Grabherr,M.,
            Kleber,M., Mauceli,E., Brockman,W., Rounsley,S., Young,S.,
            LaButti,K., Pushparaj,V., DeCaprio,D., Crawford,M., Koehrsen,M.,
            Engels,R., Montgomery,P., Pearson,M., Howarth,C., Larson,L.,
            Luoma,S., White,J., O'Leary,S., Kodira,C., Zeng,Q., Yandava,C.,
            Alvarado,L., Taylor,J. and Johannesson,H.
  CONSRTM   The Broad Institute Genome Sequencing Platform
  TITLE     Annotation of the Uncinocarpus reesii strain 1704 genome
  JOURNAL   Unpublished (2004)
REFERENCE   2  (residues 1 to 468)
  AUTHORS   Taylor,J. and Johannesson,H.
```

```
  TITLE     Direct Submission
  JOURNAL   Submitted (12-AUG-2005) Department of Plant and Microbial Biology,
            UC-Berkeley, 111 Koshland Hall, Berkeley, CA 94720-3102, USA
REFERENCE   3  (residues 1 to 468)
  AUTHORS   Lander,E., Birren,B. and Ma,L.
  CONSRTM   The Genome Sequencing Platform, The Genome Assembly Team
  TITLE     Direct Submission
  JOURNAL   Submitted (12-APR-2005) Broad Institute of MIT and Harvard, 320
            Charles Street, Cambridge, MA 02141, USA
COMMENT     PROVISIONAL REFSEQ: This record has not yet been subject to final
            NCBI review. The reference sequence is identical to EEP77416.
            Method: conceptual translation.
FEATURES             Location/Qualifiers
     source          1..468
                     /organism="Uncinocarpus reesii 1704"
                     /strain="1704"
                     /db_xref="taxon:336963"
     Protein         1..468
                     /product="rab GDP dissociation inhibitor alpha"
                     /calculated_mol_wt=51714
     Region          1..460
                     /region_name="NADB_Rossmann"
                     /note="Rossmann-fold NAD(P)(+)-binding proteins; cl09931"
                     /db_xref="CDD:176428"
     CDS             1..468
                     /locus_tag="UREG_02265"
                     /coded_by="XM_002542703.1:1..1407"
                     /db_xref="GeneID:8441543"
```

Mascot: http://www.matrixscience.com/

Spot 43

Mascot Search Results

Protein View

Match to: **gi|255936655** Score: **107** Expect: **0.0002**
Pc13g09300 [Penicillium chrysogenum Wisconsin 54-1255]

Nominal mass (M_r): **63252**; Calculated pI value: **5.65**
NCBI BLAST search of gi|255936655 against nr
Unformatted sequence string for pasting into other applications

Taxonomy: Penicillium chrysogenum Wisconsin 54-1255
Links to retrieve other entries containing this sequence from NCBI Entrez:
gi|211583974 from Penicillium chrysogenum Wisconsin 54-1255

Fixed modifications: Carbamidomethyl (C)
Variable modifications: Oxidation (M)
Cleavage by Trypsin: cuts C-term side of KR unless next residue is P
Sequence Coverage: **19%**

Matched peptides shown in **Bold Red**

```
  1 MATTDIATRE LKNPVDVAEY LFRRLHEVGI RSLHGVPGDY NLAALDYVSK
 51 CGINWVGNCN ELNAGYAADG YARVNGISAL VTTFGVGELS ALNAIAGAYS
101 EFVPVVHIVG QPTTQSQKDG MLLHHTLGNG DFNVFTKMSE GISCYVARLN
151 EPHDAATLID SAIRECWIRS RPVYITLPTD IVAAKVNGDR LKTPIDLSLP
201 KNDPEKEDYV VGVVLKYLHA AKNPVILVDA CSIRHRVLEE VRDLVEKSGL
251 PTFVTPMGKG AVNETHKNFG GVYAGNGSNV GVSEAVESSD LILSIGAIKS
301 DFNTTGFTYR VGQLNTIDFH STFVRVRYSE YPDINMKGVL RKVIERMNPL
351 TPAPTPQITN RLPESEETSS DQTITHKWLW PIVGQWLKEK DIVLTETGTA
401 NFGIWDTRFP ANVTAVSQVL WGSIGYSMGA CQGAALAAKE QEDRRTILFI
451 GDGSIQLTVQ ELSTILKNKL NPIVFVICND GYTIERYIHG WDAAYNDIQP
501 WDFANIPKVF GAKDNYQGYR IKTRDELNKL FADEDFNASD KLRLVEVYMP
551 REDAPAGLKL TAEAAAQRNK
```

Show predicted peptides also

Sort Peptides By ( ) Residue Number ( ) Increasing Mass ( ) Decreasing Mass

Start - End	Observed	Mr(expt)	Mr(calc)	ppm	Miss	Sequence
165 - 185	2488.1902	2487.1829	2487.3308	-59	1	R.ECWIRSRPVYITLPTDIVAAK.V (No match)
248 - 267	2071.0776	2070.0703	2070.0568	7	1	K.SGLPTFVTPMGKGAVNETHK.N (No match)
300 - 310	1308.5881	1307.5808	1307.5782	2	0	K.SDFNTTGFTYR.V (Ions score 80)
300 - 310	1308.5881	1307.5808	1307.5782	2	0	K.SDFNTTGFTYR.V (No match)

328 - 337	1259.6108	1258.6035	1258.5540	39	0	R.YSEYPDINMK.G	(No match)
378 - 388	1425.8173	1424.8100	1424.7969	9	0	K.WLWPIVGQWLK.E	(No match)
378 - 388	1425.8173	1424.8100	1424.7969	9	0	K.WLWPIVGQWLK.E	(No match)
389 - 408	2266.1145	2265.1072	2265.1277	-9	1	K.EKDIVLTETGTANFGIWDTR.F	(No match)
468 - 486	2265.0947	2264.0874	2264.1623	-33	1	K.NKLNPIVFVICNDGYTIER.Y	(No match)
468 - 486	2265.0947	2264.0874	2264.1623	-33	1	K.NKLNPIVFVICNDGYTIER.Y	(No match)

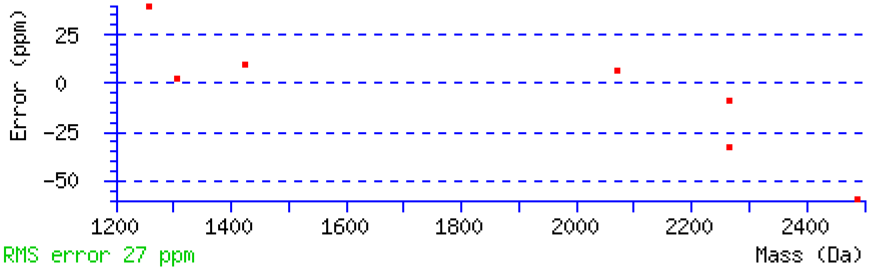

RMS error 27 ppm

```
LOCUS       XP_002559354             570 aa            linear   PLN 14-AUG-2009
DEFINITION  Pc13g09300 [Penicillium chrysogenum Wisconsin 54-1255].
ACCESSION   XP_002559354
VERSION     XP_002559354.1  GI:255936655
DBSOURCE    REFSEQ: accession XM_002559308.1
KEYWORDS    .
SOURCE      Penicillium chrysogenum Wisconsin 54-1255
  ORGANISM  Penicillium chrysogenum Wisconsin 54-1255
            Eukaryota; Fungi; Dikarya; Ascomycota; Saccharomyceta;
            Pezizomycotina; Leotiomyceta; Eurotiomycetes; Eurotiomycetidae;
            Eurotiales; Trichocomaceae; mitosporic Trichocomaceae; Penicillium;
            Penicillium chrysogenum complex.
REFERENCE   1  (residues 1 to 570)
  AUTHORS   van den Berg,M.A., Albang,R., Albermann,K., Badger,J.H.,
            Daran,J.M., Driessen,A.J., Garcia-Estrada,C., Fedorova,N.D.,
            Harris,D.M., Heijne,W.H., Joardar,V., Kiel,J.A., Kovalchuk,A.,
            Martin,J.F., Nierman,W.C., Nijland,J.G., Pronk,J.T., Roubos,J.A.,
            van der Klei,I.J., van Peij,N.N., Veenhuis,M., von Dohren,H.,
            Wagner,C., Wortman,J. and Bovenberg,R.A.
  TITLE     Genome sequencing and analysis of the filamentous fungus
            Penicillium chrysogenum
  JOURNAL   Nat. Biotechnol. 26 (10), 1161-1168 (2008)
   PUBMED   18820685
REFERENCE   2  (residues 1 to 570)
  AUTHORS   van den Berg,M.A.
  TITLE     Direct Submission
  JOURNAL   Submitted (22-NOV-2007) van den Berg M.A., DAI/INNO (624-0270), DSM
            Anti-Infectives, Alexander Fleminglaan 1, Delftn, 2613 AX,
            NETHERLANDS
```

```
COMMENT     PROVISIONAL REFSEQ: This record has not yet been subject to final
            NCBI review. The reference sequence is identical to CAP91999.
FEATURES             Location/Qualifiers
     source          1..570
                     /organism="Penicillium chrysogenum Wisconsin 54-1255"
                     /strain="Wisconsin 54-1255"
                     /db_xref="taxon:500485"
                     /clone="Pc00c13"
     Protein         1..570
                     /product="hypothetical protein"
                     /name="Pc13g09300"
                     /calculated_mol_wt=62761
     Region          12..570
                     /region_name="COG3961"
                     /note="Pyruvate decarboxylase and related thiamine
                     pyrophosphate-requiring enzymes [Carbohydrate transport
                     and metabolism / Coenzyme metabolism / General function
                     prediction only]; COG3961"
                     /db_xref="CDD:33742"
     Region          19..179
                     /region_name="TPP_PYR_PDC_IPDC_like"
                     /note="Pyrimidine (PYR) binding domain of pyruvate
                     decarboxylase (PDC), indolepyruvate decarboxylase (IPDC)
                     and related proteins; cd07038"
                     /db_xref="CDD:132921"
     Site            order(37..38,46,49,59..60,86,93,124..125,127,138,141)
                     /site_type="other"
                     /note="dimer interface"
                     /db_xref="CDD:132921"
     Site            order(37..38,46,49,54,57..60,63,66..67,69,71,73..75,86,93,
                     97,101,124..125)
                     /site_type="other"
                     /note="PYR/PP interface"
                     /db_xref="CDD:132921"
     Site            order(37,61,86,125)
                     /site_type="other"
                     /note="TPP binding site"
                     /db_xref="CDD:132921"
     Region          214..>307
                     /region_name="TPP_enzyme_M"
                     /note="Thiamine pyrophosphate enzyme, central domain;
                     pfam00205"
                     /db_xref="CDD:143962"
     Region          373..559
                     /region_name="TPP_PDC_IPDC"
                     /note="TPP-binding module; composed of proteins similar to
                     pyruvate decarboxylase (PDC) and indolepyruvate
                     decarboxylase (IPDC). PDC, a key enzyme in alcoholic
                     fermentation, catalyzes the conversion of pyruvate to
```

```
                     acetaldehyde and CO2. It is able to utilize...; cd02005"
                     /db_xref="CDD:48168"
     Site            order(399,422,424,451..454,479,481..485)
                     /site_type="other"
                     /note="TPP-binding site"
                     /db_xref="CDD:48168"
     Site            order(420..422,456..457,460,463,467,485,489,494..495,499,
                     501..502,509..510)
                     /site_type="other"
                     /note="dimer interface"
                     /db_xref="CDD:48168"
     CDS             1..570
                     /locus_tag="Pc13g09300"
                     /coded_by="XM_002559308.1:1..1713"
                     /inference="protein motif:COGS:COG0028"
                     /inference="protein motif:PFAM:PF00205"
                     /inference="protein motif:PFAM:PF02775"
                     /inference="protein motif:PFAM:PF02776"
                     /inference="similar to AA sequence:TREMBL:AF098293.1"
                     /db_xref="GeneID:8316057"
```

Mascot: http://www.matrixscience.com/

Spot 44

Mascot Search Results

Protein View

Match to: **gi|114321348** Score: **93** Expect: **0.0052**
peptidoglycan synthetase FtsI [Alkalilimnicola ehrlichii MLHE-1]

Nominal mass (M_r): **62727**; Calculated pI value: **7.96**
NCBI BLAST search of gi|114321348 against nr
Unformatted sequence string for pasting into other applications

Taxonomy: Alkalilimnicola ehrlichii MLHE-1
Links to retrieve other entries containing this sequence from NCBI Entrez:
gi|114227742 from Alkalilimnicola ehrlichii MLHE-1

Fixed modifications: Carbamidomethyl (C)
Variable modifications: Oxidation (M)
Cleavage by Trypsin: cuts C-term side of KR unless next residue is P
Sequence Coverage: **17%**

Matched peptides shown in **Bold Red**

```
  1 MSRRAQKTAM PQVAGWRLGV VALVFFLLVG ALMWRAVELQ VLDRQFLQTQ
 51 GEARQMRTRA MPAHRGVITD RHGEPLAISS PVDSVWADPG QLLDHPEAVT
101 ALAELVGMPR ARLQRRLEAR PGSEFAWVRR QVSPEQAEAV RRAALPGVAL
151 QQEYRRFYPS GEVSAHLLGF TNIDDQGQEG LELTYDAWLR GEPGRKRVIQ
201 DRLGRVVEDV ELLREPRAGR DLALSIDRRL QYLAYRELKA AVREHDARGG
251 SLVLLDVESG EVLAMVNQPG FNPHRRNEIG NGVQRNRAII DAYEPGSLIK
301 PFTVLAALRE GVVRPETSLS TSPGTLRVGR HTIRDIRDYG EIDVTTLLQK
351 SSNVGAVRLA LEMEPDALWG LLLEMGFGAS TFVGFQGEAT GRVSSMPPRD
401 EVQRATLSFG YGLTATPLQI ARAYATLAAA GVQRPVSLLH RNGQPPESEE
451 QVLDPALSRQ VLEMLETVTQ PGGTGTRAAV PGYRVAGKTG TVRKAGPTGY
501 GEDDGYIAMF AGVAPVSRPR LAMAVLVDGP RGDEYYGGQV AAPVFGQVMA
551 GALRLMNIAP DGDALPSGLI VAGGEGRP
```

Show predicted peptides also

Sort Peptides By ◉ Residue Number ○ Increasing Mass ○ Decreasing Mass

Start - End	Observed	Mr(expt)	Mr(calc)	ppm	Miss	Sequence	
36 - 44	1042.5596	1041.5523	1041.5818	-28	0	R.AVELQVLDR.Q	(No match)
58 - 65	955.4713	954.4640	954.4818	-19	1	R.TRAMPAHR.G	Oxidation (M) (No match)
60 - 71	1323.6753	1322.6680	1322.6877	-15	1	R.AMPAHRGVITDR.H	(Ions score 13)
60 - 71	1323.6753	1322.6680	1322.6877	-15	1	R.AMPAHRGVITDR.H	(No match)

230 - 239	1296.6749	1295.6676	1295.7237	-43	1	R.LQYLAYRELK.A	(No match)
240 - 248	1024.5590	1023.5517	1023.5210	30	1	K.AAVREHDAR.G	(No match)
240 - 248	1024.5590	1023.5517	1023.5210	30	1	K.AAVREHDAR.G	(No match)
276 - 285	1142.5887	1141.5814	1141.5952	-12	1	R.RNEIGNGVQR.N	(No match)
277 - 285	986.5504	985.5431	985.4941	50	0	R.NEIGNGVQR.N	(Ions score 31)
277 - 285	986.5504	985.5431	985.4941	50	0	R.NEIGNGVQR.N	(No match)
338 - 350	1494.7522	1493.7449	1493.7613	-11	0	R.DYGEIDVTTLLQK.S	(No match)
338 - 358	2265.0959	2264.0886	2264.1648	-34	1	R.DYGEIDVTTLLQKSSNVGAVR.L	(No match)
460 - 477	1932.9397	1931.9324	1931.9623	-15	0	R.QVLEMLETVTQPGGTGTR.A	Oxidation (M) (No match)
478 - 488	1088.5837	1087.5764	1087.6138	-34	1	R.AAVPGYRVAGK.T	(No match)

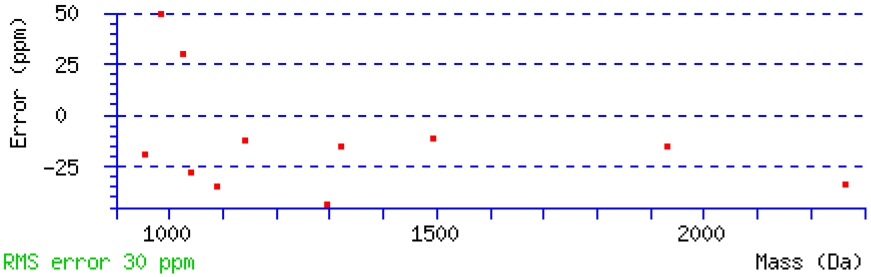


```
LOCUS       YP_743031                578 aa            linear   BCT 30-MAR-2010
DEFINITION  peptidoglycan synthetase FtsI [Alkalilimnicola ehrlichii MLHE-1].
ACCESSION   YP_743031
VERSION     YP_743031.1  GI:114321348
DBLINK      Project: 15763
DBSOURCE    REFSEQ: accession NC_008340.1
KEYWORDS    .
SOURCE      Alkalilimnicola ehrlichii MLHE-1
  ORGANISM  Alkalilimnicola ehrlichii MLHE-1
            Bacteria; Proteobacteria; Gammaproteobacteria; Chromatiales;
            Ectothiorhodospiraceae; Alkalilimnicola.
REFERENCE   1  (residues 1 to 578)
  AUTHORS   Copeland,A., Lucas,S., Lapidus,A., Barry,K., Detter,J.C., Glavina
            del Rio,T., Hammon,N., Israni,S., Dalin,E., Tice,H., Pitluck,S.,
            Sims,D., Brettin,T., Bruce,D., Han,C., Tapia,R., Gilna,P.,
            Schmutz,J., Larimer,F., Land,M., Hauser,L., Kyrpides,N.,
            Mikhailova,N., Oremland,R.S., Hoeft,S.E., Switzer-Blum,J., Kulp,T.,
            King,G., Tabita,R., Witte,B., Santini,J.M., Basu,P.,
            Hollibaugh,J.T., Xie,G., Stolz,J.F. and Richardson,P.
  CONSRTM   US DOE Joint Genome Institute
  TITLE     Complete sequence of Alkalilimnicola ehrilichei MLHE-1
  JOURNAL   Unpublished
REFERENCE   2  (residues 1 to 578)
  CONSRTM   NCBI Genome Project
  TITLE     Direct Submission
```

```
  JOURNAL   Submitted (20-SEP-2007) National Center for Biotechnology
            Information, NIH, Bethesda, MD 20894, USA
REFERENCE   3  (residues 1 to 578)
  AUTHORS   Copeland,A., Lucas,S., Lapidus,A., Barry,K., Detter,J.C., Glavina
            del Rio,T., Hammon,N., Israni,S., Dalin,E., Tice,H., Pitluck,S.,
            Sims,D., Brettin,T., Bruce,D., Han,C., Tapia,R., Gilna,P.,
            Schmutz,J., Larimer,F., Land,M., Hauser,L., Kyrpides,N.,
            Mikhailova,N., Oremland,R.S., Hoeft,S.E., Switzer-Blum,J., Kulp,T.,
            King,G., Tabita,R., Witte,B., Santini,J.M., Basu,P.,
            Hollibaugh,J.T., Xie,G., Stolz,J.F. and Richardson,P.
  CONSRTM   US DOE Joint Genome Institute
  TITLE     Direct Submission
  JOURNAL   Submitted (28-AUG-2006) US DOE Joint Genome Institute, 2800
            Mitchell Drive B100, Walnut Creek, CA 94598-1698, USA
COMMENT     PROVISIONAL REFSEQ: This record has not yet been subject to final
            NCBI review. The reference sequence was derived from ABI57541.
            Method: conceptual translation.
FEATURES             Location/Qualifiers
     source          1..578
                     /organism="Alkalilimnicola ehrlichii MLHE-1"
                     /strain="MLHE-1"
                     /db_xref="taxon:187272"
     Protein         1..578
                     /product="peptidoglycan synthetase FtsI"
                     /EC_number="2.4.1.129"
                     /calculated_mol_wt=62635
     Region          35..561
                     /region_name="FtsI"
                     /note="Cell division protein FtsI/penicillin-binding
                     protein 2 [Cell envelope biogenesis, outer membrane];
                     COG0768"
                     /db_xref="CDD:31111"
     Region          62..211
                     /region_name="PBP_dimer"
                     /note="Penicillin-binding Protein dimerization domain;
                     pfam03717"
                     /db_xref="CDD:146381"
     Region          250..550
                     /region_name="Transpeptidase"
                     /note="Penicillin binding protein transpeptidase domain;
                     cl01039"
                     /db_xref="CDD:154162"
     CDS             1..578
                     /locus_tag="Mlg_2199"
                     /coded_by="complement(NC_008340.1:2506008..2507744)"
                     /note="PFAM: penicillin-binding protein, transpeptidase;
                     Penicillin-binding protein, dimerisation domain;
                     KEGG: noc:Noc_2867 peptidoglycan glycosyltransferase"
                     /transl_table=11
```

```
/db_xref="InterPro:IPR001460"
/db_xref="InterPro:IPR005311"
/db_xref="GeneID:4268670"
```

**Mascot:** http://www.matrixscience.com/

Spot 45

Mascot Search Results

Protein View

Match to: **gi|39945784** Score: **344** Expect: **4e-028**
hypothetical protein MGG_08012 [Magnaporthe grisea 70-15]

Nominal mass (M_r): **40874**; Calculated pI value: **5.87**
NCBI BLAST search of gi|39945784 against nr
Unformatted sequence string for pasting into other applications

Taxonomy: Magnaporthe grisea 70-15
Links to retrieve other entries containing this sequence from NCBI Entrez:
gi|145019246 from Magnaporthe grisea 70-15

Fixed modifications: Carbamidomethyl (C)
Variable modifications: Oxidation (M)
Cleavage by Trypsin: cuts C-term side of KR unless next residue is P
Sequence Coverage: **15%**

Matched peptides shown in **Bold Red**

```
  1 MVRGTVLITG GTGYIGSFTS LALLENDYDV VIVDNLYNSS AVAIDRIELI
 51 CGKRPAFHNV DITDEAALDK VFDAHPEIDS VIHFAALKAV GESGEIPLEY
101 YRVNVGGSIS LLRSMQKHNV CNIVFSSSAT VYGDATRVPN MIPIPEHCPI
151 GPTNTYGRTK STIEDVISDH VNAQRNNLKK ADKPFDMWNG ALLRYFNPCG
201 AHPSGLMGED PQGVPFNLLP LLGQVATGQR EKLLVFGDDY SSRDGTAIRD
251 YIHVLDLAKG HLAALNYLRD NKPGVKAWNL GSGRGSTVFE MIKAFSSVVG
301 RDLPYEVVPR RQGDVLDLTA NPALANKELG WKTELRMEDA CQDLWKWVKN
351 NPQGYRQDPP QEFVAALKK
```

Show predicted peptides also

Sort Peptides By ◉ Residue Number ○ Increasing Mass ○ Decreasing Mass

Start - End	Observed	Mr(expt)	Mr(calc)	ppm	Miss	Sequence
89 - 102	1582.7773	1581.7700	1581.7675	2	0	K.AVGESGEIPLEYYR.V (Ions score 98)
89 - 102	1582.7773	1581.7700	1581.7675	2	0	K.AVGESGEIPLEYYR.V (No match)
138 - 158	2363.1743	2362.1670	2362.1562	5	0	R.VPNMIPIPEHCPIGPTNTYGR.T (Ions score 89)
138 - 158	2363.1743	2362.1670	2362.1562	5	0	R.VPNMIPIPEHCPIGPTNTYGR.T (No match)
138 - 158	2379.1694	2378.1621	2378.1511	5	0	R.VPNMIPIPEHCPIGPTNTYGR.T Oxidation (M) (Ions score 57)
138 - 158	2379.1694	2378.1621	2378.1511	5	0	R.VPNMIPIPEHCPIGPTNTYGR.T Oxidation (M) (No match)
244 - 259	1799.9655	1798.9582	1798.9577	0	1	R.DGTAIRDYIHVLDLAK.G (Ions score 85)
244 - 259	1799.9655	1798.9582	1798.9577	0	1	R.DGTAIRDYIHVLDLAK.G (No match)

```
250 - 259    1186.6367  1185.6294  1185.6394      -8   0  R.DYIHVLDLAK.G  (No match)
277 - 284     860.4391   859.4318   859.4300       2   0  K.AWNLGSGR.G  (Ions score 51)
277 - 284     860.4391   859.4318   859.4300       2   0  K.AWNLGSGR.G  (No match)
```

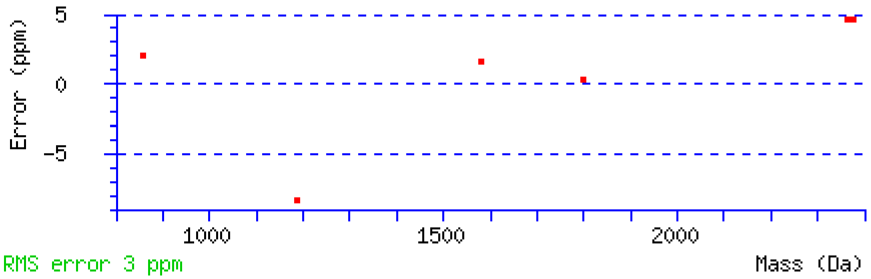


```
LOCUS       XP_362429                369 aa            linear   PLN 17-MAY-2010
DEFINITION  hypothetical protein MGG_08012 [Magnaporthe oryzae 70-15].
ACCESSION   XP_362429
VERSION     XP_362429.1  GI:39945784
DBSOURCE    REFSEQ: accession XM_362429.1
KEYWORDS    .
SOURCE      Magnaporthe oryzae 70-15
  ORGANISM  Magnaporthe oryzae 70-15
            Eukaryota; Fungi; Dikarya; Ascomycota; Saccharomyceta;
            Pezizomycotina; Leotiomyceta; Sordariomyceta; Sordariomycetes;
            Sordariomycetidae; Magnaporthales; Magnaporthaceae; Magnaporthe.
REFERENCE   1  (residues 1 to 369)
  AUTHORS   Dean,R.A., Talbot,N.J., Ebbole,D.J., Farman,M.L., Mitchell,T.K.,
            Orbach,M.J., Thon,M., Kulkarni,R., Xu,J.R., Pan,H., Read,N.D.,
            Lee,Y.H., Carbone,I., Brown,D., Oh,Y.Y., Donofrio,N., Jeong,J.S.,
            Soanes,D.M., Djonovic,S., Kolomiets,E., Rehmeyer,C., Li,W.,
            Harding,M., Kim,S., Lebrun,M.H., Bohnert,H., Coughlan,S.,
            Butler,J., Calvo,S., Ma,L.J., Nicol,R., Purcell,S., Nusbaum,C.,
            Galagan,J.E. and Birren,B.W.
  TITLE     The genome sequence of the rice blast fungus Magnaporthe grisea
  JOURNAL   Nature 434 (7036), 980-986 (2005)
   PUBMED   15846337
REFERENCE   2  (residues 1 to 369)
  AUTHORS   Birren,B., Lander,E., Galagan,J., Nusbaum,C., Devon,K., Jaffe,D.,
            Butler,J., Alvarez,P., Gnerre,S., Grabherr,M., Kleber,M.,
            Mauceli,E., Brockman,W., Rounsley,S., Young,S., LaButti,K.,
            Pushparaj,V., DeCaprio,D., Crawford,M., Koehrsen,M., Engels,R.,
            Montgomery,P., Pearson,M., Howarth,C., Kodira,C., Yandava,C.,
            Zeng,Q., Alvarado,L., Oleary,S., Dean,R., Mitchell,T., Brown,D.,
            Pan,H., Thon,M., Zhu,H. and Blackmon,B.
  CONSRTM   The Broad Institute Genome Sequencing Platform
  TITLE     Direct Submission
```

```
  JOURNAL   Submitted (26-SEP-2005) Broad Institute of MIT and Harvard, 320
            Charles Street, Cambridge, MA 02142, USA
COMMENT     PROVISIONAL REFSEQ: This record has not yet been subject to final
            NCBI review. The reference sequence was derived from EDK03474.
            Method: conceptual translation.
FEATURES             Location/Qualifiers
     source          1..369
                     /organism="Magnaporthe oryzae 70-15"
                     /strain="70-15"
                     /db_xref="taxon:242507"
                     /chromosome="I"
     Protein         1..369
                     /product="hypothetical protein"
                     /name="similar to UDP-glucose 4-epimerase Gal10"
                     /calculated_mol_wt=40483
     Region          1..355
                     /region_name="PLN02240"
                     /note="UDP-glucose 4-epimerase; PLN02240"
                     /db_xref="CDD:165883"
     Region          4..133
                     /region_name="NADB_Rossmann"
                     /note="Rossmann-fold NAD(P)(+)-binding proteins; cl09931"
                     /db_xref="CDD:176428"
     CDS             1..369
                     /locus_tag="MGG_08012"
                     /old_locus_tag="MG08012.4"
                     /coded_by="XM_362429.1:1..1110"
                     /db_xref="GeneID:2678278"
```

Mascot: http://www.matrixscience.com/

Spot 46

MATRIX SCIENCE Mascot Search Results

Protein View

Match to: **gi|145231098** Score: **198** Expect: **1.6e-013**
beta-isopropylmalate dehydrogenase A leu2A-Aspergillus niger

Nominal mass (M_r): **38955**; Calculated pI value: **5.39**
NCBI BLAST search of gi|145231098 against nr
Unformatted sequence string for pasting into other applications

Taxonomy: Aspergillus niger CBS 513.88
Links to retrieve other entries containing this sequence from NCBI Entrez:
gi|134055941 from Aspergillus niger

Fixed modifications: Carbamidomethyl (C)
Variable modifications: Oxidation (M)
Cleavage by Trypsin: cuts C-term side of KR unless next residue is P
Sequence Coverage: **16%**

Matched peptides shown in **Bold Red**

```
  1 MPAYNIVVFA GDHCGPEVTA EAIKVLRVIE KSRDDITLNL QDHLLGGASI
 51 DATGNPLTDE ALAAAKNADA VLLGAIGGPK WGTGAVRPEQ GLLKLRKEMG
101 TFGNLRPCNF AAPSLVEHSP LKASVCEGVD FNIIRELTGG IYFGDRKEDD
151 GSGYAMDTEP YSRAEIERIT RLAAHLALQH NPPLPVWSLD KANVLATSRL
201 WRKTVTEIMA KEFPQLKIEH QLIDSAAMIM VKNPRQLNGI IVTSNLFGDI
251 ISDEASVIPG SLGLLPSASL SGIPDGKGRV NGIYEPIHGS APDIAGKGIV
301 NPVAAILSVA MMMQYSFGRF DEARAIEAAV RNVLESGVRT GDIGGKATTS
351 EVGDAVAAEL EKLLK
```

Show predicted peptides also

Sort Peptides By ◉ Residue Number ○ Increasing Mass ○ Decreasing Mass

Start - End	Observed	Mr(expt)	Mr(calc)	ppm	Miss	Sequence
67 - 80	1295.7308	1294.7235	1294.7245	-1	0	K.NADAVLLGAIGGPK.W (Ions score 68)
67 - 80	1295.7308	1294.7235	1294.7245	-1	0	K.NADAVLLGAIGGPK.W (No match)
136 - 146	1227.6025	1226.5952	1226.5932	2	0	R.ELTGGIYFGDR.K (Ions score 59)
136 - 146	1227.6025	1226.5952	1226.5932	2	0	R.ELTGGIYFGDR.K (No match)
136 - 147	1355.6980	1354.6907	1354.6881	2	1	R.ELTGGIYFGDRK.E (Ions score 48)
136 - 147	1355.6980	1354.6907	1354.6881	2	1	R.ELTGGIYFGDRK.E (No match)
218 - 235	2066.0540	2065.0467	2065.0812	-17	1	K.IEHQLIDSAAMIMVKNPR.Q (No match)
347 - 362	1590.8090	1589.8017	1589.7784	15	0	K.ATTSEVGDAVAAELEK.L (Ions score 1)

347 - 362 1590.8090 1589.8017 1589.7784 15 0 K.ATTSEVGDAVAAELEK.L (No match)

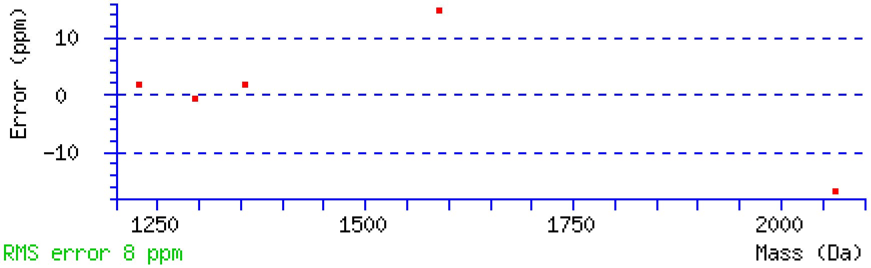


```
LOCUS       XP_001389813             365 aa            linear   PLN 28-FEB-2008
DEFINITION  beta-isopropylmalate dehydrogenase A leu2A-Aspergillus niger.
ACCESSION   XP_001389813
VERSION     XP_001389813.1  GI:145231098
DBSOURCE    REFSEQ: accession XM_001389776.1
KEYWORDS    .
SOURCE      Aspergillus niger CBS 513.88
  ORGANISM  Aspergillus niger CBS 513.88
            Eukaryota; Fungi; Dikarya; Ascomycota; Saccharomyceta;
            Pezizomycotina; Leotiomyceta; Eurotiomycetes; Eurotiomycetidae;
            Eurotiales; Trichocomaceae; mitosporic Trichocomaceae; Aspergillus.
REFERENCE   1  (residues 1 to 365)
  AUTHORS   Pel,H.J., de Winde,J.H., Archer,D.B., Dyer,P.S., Hofmann,G.,
            Schaap,P.J., Turner,G., de Vries,R.P., Albang,R., Albermann,K.,
            Andersen,M.R., Bendtsen,J.D., Benen,J.A., van den Berg,M.,
            Breestraat,S., Caddick,M.X., Contreras,R., Cornell,M.,
            Coutinho,P.M., Danchin,E.G., Debets,A.J., Dekker,P., van
            Dijck,P.W., van Dijk,A., Dijkhuizen,L., Driessen,A.J., d'Enfert,C.,
            Geysens,S., Goosen,C., Groot,G.S., de Groot,P.W., Guillemette,T.,
            Henrissat,B., Herweijer,M., van den Hombergh,J.P., van den
            Hondel,C.A., van der Heijden,R.T., van der Kaaij,R.M., Klis,F.M.,
            Kools,H.J., Kubicek,C.P., van Kuyk,P.A., Lauber,J., Lu,X., van der
            Maarel,M.J., Meulenberg,R., Menke,H., Mortimer,M.A., Nielsen,J.,
            Oliver,S.G., Olsthoorn,M., Pal,K., van Peij,N.N., Ram,A.F.,
            Rinas,U., Roubos,J.A., Sagt,C.M., Schmoll,M., Sun,J., Ussery,D.,
            Varga,J., Vervecken,W., van de Vondervoort,P.J., Wedler,H.,
            Wosten,H.A., Zeng,A.P., van Ooyen,A.J., Visser,J. and Stam,H.
  TITLE     Genome sequencing and analysis of the versatile cell factory
            Aspergillus niger CBS 513.88
  JOURNAL   Nat. Biotechnol. 25 (2), 221-231 (2007)
   PUBMED   17259976
COMMENT     PROVISIONAL REFSEQ: This record has not yet been subject to final
            NCBI review. The reference sequence was derived from CAK37418.
FEATURES             Location/Qualifiers
```

```
source          1..365
                /organism="Aspergillus niger CBS 513.88"
                /db_xref="taxon:425011"
                /clone="An01"
Protein         1..365
                /product="beta-isopropylmalate dehydrogenase A
                leu2A-Aspergillus niger"
                /EC_number="1.1.1.85"
                /calculated_mol_wt=38677
Region          4..363
                /region_name="Iso_dh"
                /note="Isocitrate/isopropylmalate dehydrogenase; cl00445"
                /db_xref="CDD:174206"
Region          6..363
                /region_name="PRK06451"
                /note="isocitrate dehydrogenase; Validated; PRK06451"
                /db_xref="CDD:168554"
CDS             1..365
                /gene="leu2A"
                /locus_tag="An01g14130"
                /coded_by="XM_001389776.1:1..1098"
                /inference="profile:COGS:COG0473"
                /inference="profile:PFAM:PF00180"
                /inference="similar to AA sequence:PIR:S72209"
                /note="Catalytic activity:
                3-Carboxy-2-hydroxy-4-methylpentanoate + NAD+ =
                3-Carboxy-4-methyl-2-oxopentanoate + NADH.;
                Gene-ID: leu2A;
                Pathway: valine, leucine and isoleucine biosynthesis;
                Remark: in A. niger are two highly
                divergent,differentially regulated, isozymes for
                beta-isopropylmalate dehydrogenase present.;
                Remark: the differences in the sequence might be caused by
                strain variations or sequencing errors."
                /citation=[PUBMED 8781173]
                /db_xref="GOA:A2QB74"
                /db_xref="GeneID:4977432"
```

Mascot: http://www.matrixscience.com/

Spot 47

{MATRIX SCIENCE} Mascot Search Results

Protein View

Match to: **gi|74627960** Score: **191** Expect: **7.9e-013**
RecName: Full=Mannitol-1-phosphate 5-dehydrogenase; Short=M1PDH; Short=MPDH; Short=MPD

Nominal mass (M_r): **43695**; Calculated pI value: **5.96**
NCBI BLAST search of gi|74627960 against nr
Unformatted sequence string for pasting into other applications

Taxonomy: Alternaria alternata
Links to retrieve other entries containing this sequence from NCBI Entrez:
gi|34329597 from Alternaria alternata

Fixed modifications: Carbamidomethyl (C)
Variable modifications: Oxidation (M)
Cleavage by Trypsin: cuts C-term side of KR unless next residue is P
Sequence Coverage: **17%**

Matched peptides shown in **Bold Red**

```
  1 MSYEKKAVHF GGGNIGRGFV AEFLHNSGYE VVFVDVMDSI IESLQKTKTY
 51 TVTEIGDDGE RKFTIDHYRA INSKHEMDKV VQEIASADVV TCAVGPNILK
101 FVAEPVAKAI EARTLDYPIA VIACENAINA TTTWRGFIES KLSEETKKNI
151 DSKARFANSA IDRIVPQQPP NGGLDVVIEK FHEWCVEQKP FENGGKKPDV
201 KGIHYVDDLE PYIERKLFTV NTSHATAAYY GHQNKVQYIH EVLHDKKLHD
251 TVRDAVKETA HLIVTKHGVE TAEQDAYVEE IIKRISNPVL KDNVERVGRA
301 PLRKLSRKER FIGPAAQLAE RGEKVDALLG AVEQAYRFQN VEGDEESVEL
351 AKILKENSAE EVVTKVNGIE KGQPLFDRLV AIVKKVQGGS
```

Show predicted peptides also

Sort Peptides By (•) Residue Number ( ) Increasing Mass ( ) Decreasing Mass

Start - End	Observed	Mr(expt)	Mr(calc)	ppm	Miss	Sequence
6 - 17	1212.6582	1211.6509	1211.6523	-1	1	K.KAVHFGGGNIGR.G (No match)
7 - 17	1084.5658	1083.5585	1083.5574	1	0	K.AVHFGGGNIGR.G (Ions score 87)
7 - 17	1084.5658	1083.5585	1083.5574	1	0	K.AVHFGGGNIGR.G (No match)
62 - 69	1079.5262	1078.5189	1078.5560	-34	1	R.KFTIDHYR.A (No match)
156 - 163	893.4529	892.4456	892.4403	6	0	R.FANSAIDR.I (Ions score 36)
156 - 163	893.4529	892.4456	892.4403	6	0	R.FANSAIDR.I (No match)
322 - 337	1718.8867	1717.8794	1717.8999	-12	1	R.GEKVDALLGAVEQAYR.F (No match)
338 - 352	1693.7928	1692.7855	1692.7842	1	0	R.FQNVEGDEESVELAK.I (Ions score 37)

338 - 352	1693.7928	1692.7855	1692.7842	1	0	R.FQNVEGDEESVELAK.I	(No match)
356 - 365	1105.5876	1104.5803	1104.5299	46	0	K.ENSAEEVVTK.V	(No match)

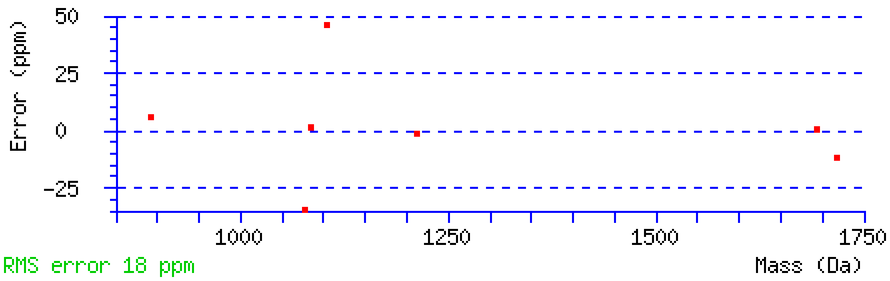


```
LOCUS       MTLD_ALTAL                390 aa            linear   PLN 13-JUL-2010
DEFINITION  RecName: Full=Mannitol-1-phosphate 5-dehydrogenase; Short=M1PDH;
            Short=MPDH; Short=MPD.
ACCESSION   Q6UQ76
VERSION     Q6UQ76.1  GI:74627960
DBSOURCE    UniProtKB: locus MTLD_ALTAL, accession Q6UQ76;
            class: standard.
            created: May 5, 2009.
            sequence updated: Jul 5, 2004.
            annotation updated: Jul 13, 2010.
            xrefs: AY364263.1, AAQ63948.1
            xrefs (non-sequence databases): SMR:Q6UQ76, GO:0050662, GO:0008926,
            GO:0055114, InterPro:IPR008927, InterPro:IPR013328,
            InterPro:IPR013118, InterPro:IPR000669, InterPro:IPR013131,
            InterPro:IPR016040, Gene3D:G3DSA:3.40.50.720,
            Gene3D:G3DSA:1.10.1040.10, Pfam:PF01232, Pfam:PF08125,
            PRINTS:PR00084, SUPFAM:SSF48179, PROSITE:PS00974
KEYWORDS    NAD; Oxidoreductase.
SOURCE      Alternaria alternata
  ORGANISM  Alternaria alternata
            Eukaryota; Fungi; Dikarya; Ascomycota; Saccharomyceta;
            Pezizomycotina; Leotiomyceta; Dothideomyceta; Dothideomycetes;
            Pleosporomycetidae; Pleosporales; Pleosporineae; Pleosporaceae;
            mitosporic Pleosporaceae; Alternaria; Alternaria alternata group.
REFERENCE   1  (residues 1 to 390)
  AUTHORS   Velez,H., Glassbrook,N.J. and Daub,M.E.
  TITLE     Mannitol metabolism in the phytopathogenic fungus Alternaria
            alternata
  JOURNAL   Fungal Genet. Biol. 44 (4), 258-268 (2007)
   PUBMED   17092745
  REMARK    NUCLEOTIDE SEQUENCE [GENOMIC DNA], AND FUNCTION.
COMMENT     [FUNCTION] Catalyzes the NAD(H)-dependent interconversion of
            D-fructose 6-phosphate and D-mannitol 1-phosphate in the mannitol
```

```
            metabolic pathway. Has a strong preference for NADH over NADPH.
            [CATALYTIC ACTIVITY] D-mannitol 1-phosphate + NAD(+) = D-fructose
            6-phosphate + NADH.
            [SUBUNIT] Monomer (By similarity).
            [SIMILARITY] Belongs to the mannitol dehydrogenase family.
FEATURES             Location/Qualifiers
     source          1..390
                     /organism="Alternaria alternata"
                     /db_xref="taxon:5599"
     Protein         1..390
                     /product="Mannitol-1-phosphate 5-dehydrogenase"
                     /EC_number="1.1.1.17"
                     /note="M1PDH; MPDH"
                     /UniProtKB_evidence="Inferred from homology"
     Region          1..390
                     /region_name="Mature chain"
                     /experiment="experimental evidence, no additional details
                     recorded"
                     /note="Mannitol-1-phosphate 5-dehydrogenase.
                     /FTId=PRO_0000371517."
     Region          5..387
                     /region_name="PRK02318"
                     /note="mannitol-1-phosphate 5-dehydrogenase; Provisional;
                     PRK02318"
                     /db_xref="CDD:167365"
     Region          6..126
                     /region_name="Mannitol_dh"
                     /note="Mannitol dehydrogenase Rossmann domain; pfam01232"
                     /db_xref="CDD:144722"
     Site            7..18
                     /site_type="np-binding"
                     /inference="non-experimental evidence, no additional
                     details recorded"
                     /note="NAD (By similarity)."
     Region          156..375
                     /region_name="Mannitol_dh_C"
                     /note="Mannitol dehydrogenase C-terminal domain;
                     pfam08125"
                     /db_xref="CDD:149275"
     Site            216
                     /site_type="active"
                     /inference="non-experimental evidence, no additional
                     details recorded"
                     /note="By similarity."
```

Mascot: http://www.matrixscience.com/

Spot 48

Mascot Search Results

Protein View

Match to: **gi|255942351** Score: **250** Expect: **1e-018**
Pc18g00980 [Penicillium chrysogenum Wisconsin 54-1255]

Nominal mass (M_r): **57162**; Calculated pI value: **5.62**
NCBI BLAST search of gi|255942351 against nr
Unformatted sequence string for pasting into other applications

Taxonomy: Penicillium chrysogenum Wisconsin 54-1255
Links to retrieve other entries containing this sequence from NCBI Entrez:
gi|211586677 from Penicillium chrysogenum Wisconsin 54-1255

Fixed modifications: Carbamidomethyl (C)
Variable modifications: Oxidation (M)
Cleavage by Trypsin: cuts C-term side of KR unless next residue is P
Sequence Coverage: **21%**

Matched peptides shown in **Bold Red**

```
  1 MTHPDISVDV LVIGAGPTGL GAAKRLNQIN GPSWMIIDSN ETPGGLASTD
 51 VTPEGFLYDV GGHVIFSHYK YFDDCIDEAL PKEEDWYTHE RISYVRCQEQ
101 WVPYPFQNNI SMLPKEEQVK CIDGMIDAAL EARVSNTKPK DFDEWIVRMM
151 GTGIADLFMR PYNYKVWAVP TTKMQCAWLG ERVAAPNVKA VTTNVILNKT
201 AGNWGPNATF RFPARDGTGG IWIAVANTIP KENTRYGPKN KVEKVNAYNK
251 TVTLADGTTI GYGKLVSTMA VDYLAEAMND TELMPLTKQL FYSSTHVIGV
301 GIRGARPDRI GDKCWLYFPE NDCPFYRATI FSNYSPHNQP EASKKLPTIQ
351 LADGSKPKST EPQEGPYWSV MLEVSESSMK PVNNETLLAE SIQGLVNTQM
401 LQPGDEIVST YHRRFDHGYP TPTLEREGAL TQILPKLQEK GIWSRGRFGS
451 WRYEVGNQDH SFMLGVEAVD NIVNGAVELT LNYPDFVNGR QNTERRLVDG
501 AQIFAKKQ
```

Show predicted peptides also

Sort Peptides By ◉ Residue Number ○ Increasing Mass ○ Decreasing Mass

Start - End	Observed	Mr(expt)	Mr(calc)	ppm	Miss	Sequence
71 - 82	1485.6563	1484.6490	1484.6493	-0	0	K.YFDDCIDEALPK.E (Ions score 61)
71 - 82	1485.6563	1484.6490	1484.6493	-0	0	K.YFDDCIDEALPK.E (No match)
141 - 148	1079.5154	1078.5081	1078.5084	-0	0	K.DFDEWIVR.M (Ions score 53)
141 - 148	1079.5154	1078.5081	1078.5084	-0	0	K.DFDEWIVR.M (No match)
149 - 165	2007.9590	2006.9517	2006.9416	5	0	R.MMGTGIADLFMRPYNYK.V (No match)

149 - 165	2023.9508	2022.9435	2022.9365	3	0	R.MMGTGIADLFMRPYNYK.V	Oxidation (M) (No match)
149 - 165	2039.9510	2038.9437	2038.9315	6	0	R.MMGTGIADLFMRPYNYK.V	2 Oxidation (M) (No match)
166 - 173	901.5137	900.5064	900.5069	-1	0	K.VWAVPTTK.M	(No match)
216 - 231	1612.8663	1611.8590	1611.8621	-2	0	R.DGTGGIWIAVANTIPK.E	(Ions score 34)
216 - 231	1612.8663	1611.8590	1611.8621	-2	0	R.DGTGGIWIAVANTIPK.E	(No match)
242 - 250	1064.5836	1063.5763	1063.5662	10	1	K.VEKVNAYNK.T	(No match)
314 - 327	1966.8308	1965.8235	1965.8178	3	0	K.CWLYFPENDCPFYR.A	(Ions score 32)
314 - 327	1966.8308	1965.8235	1965.8178	3	0	K.CWLYFPENDCPFYR.A	(No match)
328 - 344	1890.9747	1889.9674	1889.8908	41	0	R.ATIFSNYSPHNQPEASK.K	(No match)
437 - 445	1116.5822	1115.5749	1115.6087	-30	1	K.LQEKGIWSR.G	(Ions score 28)
437 - 445	1116.5822	1115.5749	1115.6087	-30	1	K.LQEKGIWSR.G	(No match)

Error: try setting browser cache to automatic.

```
LOCUS       XP_002561944             508 aa            linear   PLN 14-AUG-2009
DEFINITION  Pc18g00980 [Penicillium chrysogenum Wisconsin 54-1255].
ACCESSION   XP_002561944
VERSION     XP_002561944.1  GI:255942351
DBSOURCE    REFSEQ: accession XM_002561898.1
KEYWORDS    .
SOURCE      Penicillium chrysogenum Wisconsin 54-1255
  ORGANISM  Penicillium chrysogenum Wisconsin 54-1255
            Eukaryota; Fungi; Dikarya; Ascomycota; Saccharomyceta;
            Pezizomycotina; Leotiomyceta; Eurotiomycetes; Eurotiomycetidae;
            Eurotiales; Trichocomaceae; mitosporic Trichocomaceae; Penicillium;
            Penicillium chrysogenum complex.
REFERENCE   1  (residues 1 to 508)
  AUTHORS   van den Berg,M.A., Albang,R., Albermann,K., Badger,J.H.,
            Daran,J.M., Driessen,A.J., Garcia-Estrada,C., Fedorova,N.D.,
            Harris,D.M., Heijne,W.H., Joardar,V., Kiel,J.A., Kovalchuk,A.,
            Martin,J.F., Nierman,W.C., Nijland,J.G., Pronk,J.T., Roubos,J.A.,
            van der Klei,I.J., van Peij,N.N., Veenhuis,M., von Dohren,H.,
            Wagner,C., Wortman,J. and Bovenberg,R.A.
  TITLE     Genome sequencing and analysis of the filamentous fungus
            Penicillium chrysogenum
  JOURNAL   Nat. Biotechnol. 26 (10), 1161-1168 (2008)
   PUBMED   18820685
REFERENCE   2  (residues 1 to 508)
```

```
  AUTHORS   van den Berg,M.A.
  TITLE     Direct Submission
  JOURNAL   Submitted (22-NOV-2007) van den Berg M.A., DAI/INNO (624-0270), DSM
            Anti-Infectives, Alexander Fleminglaan 1, Delftn, 2613 AX,
            NETHERLANDS
COMMENT     PROVISIONAL REFSEQ: This record has not yet been subject to final
            NCBI review. The reference sequence is identical to CAP94322.
FEATURES             Location/Qualifiers
     source          1..508
                     /organism="Penicillium chrysogenum Wisconsin 54-1255"
                     /strain="Wisconsin 54-1255"
                     /db_xref="taxon:500485"
                     /clone="Pc00c18"
     Protein         1..508
                     /product="hypothetical protein"
                     /name="Pc18g00980"
                     /calculated_mol_wt=56726
     Region          9..>78
                     /region_name="NADB_Rossmann"
                     /note="Rossmann-fold NAD(P)(+)-binding proteins; cl09931"
                     /db_xref="CDD:176428"
     Region          10..472
                     /region_name="HemY"
                     /note="Protoporphyrinogen oxidase [Coenzyme metabolism];
                     COG1232"
                     /db_xref="CDD:31425"
     CDS             1..508
                     /locus_tag="Pc18g00980"
                     /coded_by="XM_002561898.1:1..1527"
                     /inference="protein motif:COGS:COG1232"
                     /inference="protein motif:COGS:COG1233"
                     /inference="protein motif:COGS:COG3349"
                     /inference="similar to AA sequence:TREMBL:AB053356.1"
                     /db_xref="GeneID:8314645"
```

Mascot: http://www.matrixscience.com/

Spot 49

Mascot Search Results

Protein View

Match to: **gi|145232889** Score: **118** Expect: **1.6e-005**
hypothetical protein An02g06820 [Aspergillus niger]

Nominal mass (M_r): **62977**; Calculated pI value: **6.29**
NCBI BLAST search of gi|145232889 against nr
Unformatted sequence string for pasting into other applications

Taxonomy: Aspergillus niger CBS 513.88
Links to retrieve other entries containing this sequence from NCBI Entrez:
gi|134056738 from Aspergillus niger

Fixed modifications: Carbamidomethyl (C)
Variable modifications: Oxidation (M)
Cleavage by Trypsin: cuts C-term side of KR unless next residue is P
Sequence Coverage: **18%**

Matched peptides shown in **Bold Red**

```
  1 MATDIATRDL RKPTTVAEYL FRRLHEVGVR SVHGVPGDYN LAALDYLPKC
 51 GLHWVGNCNE LNAGYAADGY ARVNGIGALI TTFGVGELSA LNAIAGSYSE
101 FVPVVHIVGQ PNTKSQKDGM LLHHTLGNGD FNVFAKMSAG ISCTLGRLNE
151 TLEAATLIDN AIRECWIRSR PVYISLPTDM IVKQIEGDRL DKPLDLSLPA
201 NDPEKEDYVV DVVLKYLHAA KKPVILVDAC AIRHRVLDEV HDLMEASGLP
251 TFVAPMGKGA VDETRPNYGG VYAGTGSNAG VREQVESSDL ILSIGAIKSD
301 FNTSGFSYHI GQLNTIDFHS TYVRVRYSEY PEINMKGVLR KVIQRMGAVN
351 AAPVPHLSNT LPESEKSSSS QEITHDWLWP NVGQWLKEND IVITETGTAN
401 FGIWETRFPA NVTAISQVLW GSIGYSVGAC QGAALAAKEL GNRRTVLFVG
451 DGSLQLTVQE LSTMIRNNLN PIIFVICNNG YTIERYIHGW DESYNDIQPW
501 DIEGLPRVFG AKDKYKGYKV KTRDELRQLF ANQEFASAPY LQFTCLVMML
551 PPRXKSRLRR LPPGTSK
```

Show predicted peptides also

Sort Peptides By ◉ Residue Number ○ Increasing Mass ○ Decreasing Mass

Start - End	Observed	Mr(expt)	Mr(calc)	ppm	Miss	Sequence
1 - 11	1278.6693	1277.6620	1277.6398	17	1	-.MATDIATRDLR.K Oxidation (M) (No match)
118 - 136	2086.0234	2085.0161	2085.0102	3	0	K.DGMLLHHTLGNGDFNVFAK.M (No match)
118 - 136	2086.0234	2085.0161	2085.0102	3	0	K.DGMLLHHTLGNGDFNVFAK.M (No match)
118 - 136	2102.0361	2101.0288	2101.0051	11	0	K.DGMLLHHTLGNGDFNVFAK.M Oxidation (M) (No match)

148 - 163	1756.9080	1755.9007	1755.9366	-20	0	R.LNETLEAATLIDNAIR.E	(No match)
148 - 163	1756.9080	1755.9007	1755.9366	-20	0	R.LNETLEAATLIDNAIR.E	(No match)
169 - 183	1734.9344	1733.9271	1733.9386	-7	0	R.SRPVYISLPTDMIVK.Q	Oxidation (M) (No match)
236 - 258	2488.1782	2487.1709	2487.2026	-13	0	R.VLDEVHDLMEASGLPTFVAPMGK.G	2 Oxidation (M) (No match)
388 - 407	2266.1086	2265.1013	2265.0913	4	0	K.ENDIVITETGTANFGIWETR.F	(Ions score 97)
388 - 407	2266.1086	2265.1013	2265.0913	4	0	K.ENDIVITETGTANFGIWETR.F	(No match)

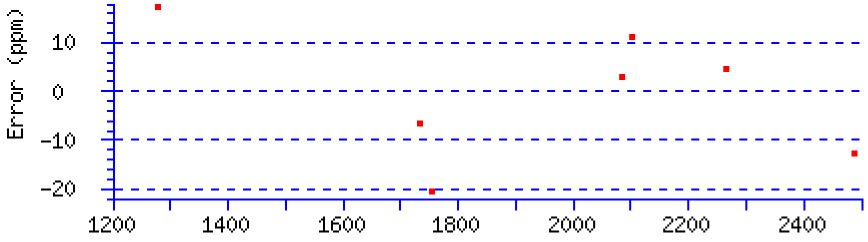

RMS error 12 ppm

```
LOCUS       XP_001399817             567 aa            linear   PLN 28-FEB-2008
DEFINITION  hypothetical protein An02g06820 [Aspergillus niger].
ACCESSION   XP_001399817
VERSION     XP_001399817.1  GI:145232889
DBSOURCE    REFSEQ: accession XM_001399780.1
KEYWORDS    .
SOURCE      Aspergillus niger CBS 513.88
  ORGANISM  Aspergillus niger CBS 513.88
            Eukaryota; Fungi; Dikarya; Ascomycota; Saccharomyceta;
            Pezizomycotina; Leotiomyceta; Eurotiomycetes; Eurotiomycetidae;
            Eurotiales; Trichocomaceae; mitosporic Trichocomaceae; Aspergillus.
REFERENCE   1  (residues 1 to 567)
  AUTHORS   Pel,H.J., de Winde,J.H., Archer,D.B., Dyer,P.S., Hofmann,G.,
            Schaap,P.J., Turner,G., de Vries,R.P., Albang,R., Albermann,K.,
            Andersen,M.R., Bendtsen,J.D., Benen,J.A., van den Berg,M.,
            Breestraat,S., Caddick,M.X., Contreras,R., Cornell,M.,
            Coutinho,P.M., Danchin,E.G., Debets,A.J., Dekker,P., van
            Dijck,P.W., van Dijk,A., Dijkhuizen,L., Driessen,A.J., d'Enfert,C.,
            Geysens,S., Goosen,C., Groot,G.S., de Groot,P.W., Guillemette,T.,
            Henrissat,B., Herweijer,M., van den Hombergh,J.P., van den
            Hondel,C.A., van der Heijden,R.T., van der Kaaij,R.M., Klis,F.M.,
            Kools,H.J., Kubicek,C.P., van Kuyk,P.A., Lauber,J., Lu,X., van der
            Maarel,M.J., Meulenberg,R., Menke,H., Mortimer,M.A., Nielsen,J.,
            Oliver,S.G., Olsthoorn,M., Pal,K., van Peij,N.N., Ram,A.F.,
            Rinas,U., Roubos,J.A., Sagt,C.M., Schmoll,M., Sun,J., Ussery,D.,
            Varga,J., Vervecken,W., van de Vondervoort,P.J., Wedler,H.,
            Wosten,H.A., Zeng,A.P., van Ooyen,A.J., Visser,J. and Stam,H.
  TITLE     Genome sequencing and analysis of the versatile cell factory
            Aspergillus niger CBS 513.88
```

```
  JOURNAL   Nat. Biotechnol. 25 (2), 221-231 (2007)
   PUBMED   17259976
COMMENT     PROVISIONAL REFSEQ: This record has not yet been subject to final
            NCBI review. The reference sequence was derived from CAK44227.
FEATURES             Location/Qualifiers
     source          1..567
                     /organism="Aspergillus niger CBS 513.88"
                     /db_xref="taxon:425011"
                     /clone="An02"
     Protein         1..567
                     /product="hypothetical protein"
     Region          12..548
                     /region_name="COG3961"
                     /note="Pyruvate decarboxylase and related thiamine
                     pyrophosphate-requiring enzymes [Carbohydrate transport
                     and metabolism / Coenzyme metabolism / General function
                     prediction only]; COG3961"
                     /db_xref="CDD:33742"
     Region          18..178
                     /region_name="TPP_PYR_PDC_IPDC_like"
                     /note="Pyrimidine (PYR) binding domain of pyruvate
                     decarboxylase (PDC), indolepyruvate decarboxylase (IPDC)
                     and related proteins; cd07038"
                     /db_xref="CDD:132921"
     Site            order(36..37,45,48,58..59,85,92,123..124,126,137,140)
                     /site_type="other"
                     /note="dimer interface"
                     /db_xref="CDD:132921"
     Site            order(36..37,45,48,53,56..59,62,65..66,68,70,72..74,85,92,
                     96,100,123..124)
                     /site_type="other"
                     /note="PYR/PP interface"
                     /db_xref="CDD:132921"
     Site            order(36,60,85,124)
                     /site_type="other"
                     /note="TPP binding site"
                     /db_xref="CDD:132921"
     Region          210..>307
                     /region_name="TPP_enzyme_M"
                     /note="Thiamine pyrophosphate enzyme, central domain;
                     pfam00205"
                     /db_xref="CDD:143962"
     Region          373..537
                     /region_name="TPP_PDC_IPDC"
                     /note="TPP-binding module; composed of proteins similar to
                     pyruvate decarboxylase (PDC) and indolepyruvate
                     decarboxylase (IPDC). PDC, a key enzyme in alcoholic
                     fermentation, catalyzes the conversion of pyruvate to
                     acetaldehyde and CO2. It is able to utilize...; cd02005"
```

```
                     /db_xref="CDD:48168"
     Site            order(398,421,423,450..453,478,480..484)
                     /site_type="other"
                     /note="TPP-binding site"
                     /db_xref="CDD:48168"
     Site            order(419..421,455..456,459,462,466,484,488,493..494,498,
                     500..501,508..509)
                     /site_type="other"
                     /note="dimer interface"
                     /db_xref="CDD:48168"
     CDS             1..567
                     /locus_tag="An02g06820"
                     /coded_by="XM_001399780.1:1..1700"
                     /inference="profile:COGS:COG0028"
                     /inference="profile:PFAM:PF00205"
                     /inference="profile:PFAM:PF02775"
                     /inference="profile:PFAM:PF02776"
                     /inference="similar to AA sequence:UniProtKB:AF098293.1"
                     /exception="reasons given in citation"
                     /note="Catalytic activity: a 2-oxo acid = an aldehyde +
                     CO2 ; pyruvate = acetaldehyde + CO2.;
                     Pathway: glycolysis / gluconeogenesis.;
                     Remark: PDC levels are a major determinant of ethanol
                     production.;
                     Similarity: belongs to the thiamine-diphosphate protein
                     family.;
                     Title: strong similarity to pyruvate decarboxylase pdcA -
                     Aspergillus oryzae;
                     putative sequencing error"
                     /citation=[PUBMED 9210590]
                     /db_xref="GeneID:4979172"
```

Mascot: http://www.matrixscience.com/

Spot 50

Mascot Search Results

Protein View

Match to: **gi|256729098** Score: **240** Expect: **1e-017**
predicted protein [Nectria haematococca mpVI 77-13-4]

Nominal mass (M_r): **37591**; Calculated pI value: **6.39**
NCBI BLAST search of gi|256729098 against nr
Unformatted sequence string for pasting into other applications

Taxonomy: Nectria haematococca mpVI 77-13-4

Fixed modifications: Carbamidomethyl (C)
Variable modifications: Oxidation (M)
Cleavage by Trypsin: cuts C-term side of KR unless next residue is P
Sequence Coverage: **16%**

Matched peptides shown in **Bold Red**

```
  1 MAAEIPTTQW AQVVEKNGGP TKYQQVPVRQ PGPDEVLVNV KFSGVCHTDL
 51 HAMMGDWPLD TKLPLIGGHE GAGVVVSRGQ LVEDVEIGDY VGIKWLHGSC
101 LQCSFCQTSD EPLCAKALLS GYTVDGSFQQ YAVAKAAHVA RIPKECDLEA
151 ISPILCAGIT VYKGLKESGV KAGQTVAIVG AGGGLGSIAL QYAKAMGIHT
201 IAIDGGDDKK ALCESLGASA FVDFTKSSNL VAEVKAATSD GLGPHAVLLV
251 AVQEKPFQQA TQYVRSRGTV VCIGLPANAS LSAPVFDTVV RMISIKGSYV
301 GNRADTAEAI EFYRRGLIKV PFKTVGLSEL QSVYDLMTAG KIAGRYVVDT
351 SR
```

Show predicted peptides also

Sort Peptides By ○ Residue Number ● Increasing Mass ○ Decreasing Mass

Start - End	Observed	Mr(expt)	Mr(calc)	ppm	Miss	Sequence
79 - 94	1733.7544	1732.7471	1732.8883	-81	0	R.GQLVEDVEIGDYVGIK.W (No match)
172 - 194	2102.1584	2101.1511	2101.1532	-1	0	K.AGQTVAIVGAGGGLGSIALQYAK.A (Ions score 225)
172 - 194	2102.1584	2101.1511	2101.1532	-1	0	K.AGQTVAIVGAGGGLGSIALQYAK.A (No match)
227 - 235	946.5216	945.5143	945.5131	1	0	K.SSNLVAEVK.A (No match)
304 - 314	1285.6066	1284.5993	1284.5986	1	0	R.ADTAEAIEFYR.R (No match)

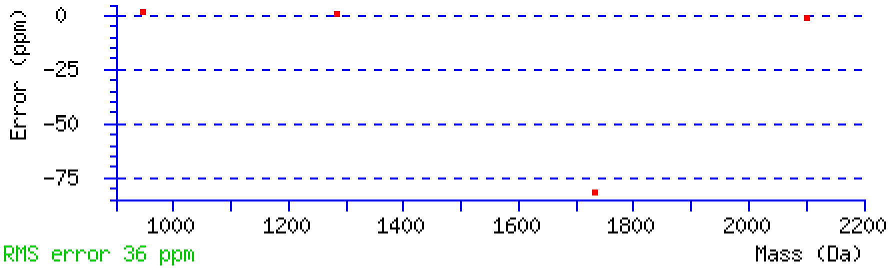


```
LOCUS       EEU42453                 352 aa            linear   PLN 30-AUG-2009
DEFINITION  predicted protein [Nectria haematococca mpVI 77-13-4].
ACCESSION   EEU42453
VERSION     EEU42453.1  GI:256729098
DBLINK      Project: 16586
DBSOURCE    accession GG698905.1
KEYWORDS    .
SOURCE      Nectria haematococca mpVI 77-13-4
  ORGANISM  Nectria haematococca mpVI 77-13-4
            Eukaryota; Fungi; Dikarya; Ascomycota; Saccharomyceta;
            Pezizomycotina; Leotiomyceta; Sordariomyceta; Sordariomycetes;
            Hypocreomycetidae; Hypocreales; Nectriaceae; Nectria; Nectria
            haematococca complex.
REFERENCE   1  (residues 1 to 352)
  AUTHORS   Coleman,J.J., Rounsley,S.D., Rodriguez-Carres,M., Kuo,A.,
            Wasmann,C.C., Grimwood,J., Schmutz,J., Taga,M., White,G.J.,
            Zhou,S., Schwartz,D.C., Freitag,M., Ma,L.J., Danchin,E.G.,
            Henrissat,B., Coutinho,P.M., Nelson,D.R., Straney,D., Napoli,C.A.,
            Barker,B.M., Gribskov,M., Rep,M., Kroken,S., Molnar,I., Rensing,C.,
            Kennell,J.C., Zamora,J., Farman,M.L., Selker,E.U., Salamov,A.,
            Shapiro,H., Pangilinan,J., Lindquist,E., Lamers,C., Grigoriev,I.V.,
            Geiser,D.M., Covert,S.F., Temporini,E. and Vanetten,H.D.
  TITLE     The genome of Nectria haematococca: contribution of supernumerary
            chromosomes to gene expansion
  JOURNAL   PLoS Genet. 5 (8), E1000618 (2009)
   PUBMED   19714214
REFERENCE   2  (residues 1 to 352)
  AUTHORS   Kuo,A., Salamov,A., Grimwood,J., Schmutz,J., Pangilinan,J.,
            Shapiro,H., Lindquist,E., Lucas,S., Pitluck,S., Henrissat,B.,
            Geiser,D.M., Coleman,J.J., Kroken,S., Napoli,C.A., Rep,M.,
            Covert,S.F., Temporini,E., VanEtten,H.D. and Grigoriev,I.
  CONSRTM   US DOE Joint Genome Institute (JGI-PGF)
  TITLE     Direct Submission
  JOURNAL   Submitted (14-JUL-2009) US DOE Joint Genome Institute, 2800
            Mitchell Drive, Walnut Creek, CA 94598-1698, USA
```

```
COMMENT     Method: conceptual translation.
FEATURES             Location/Qualifiers
     source          1..352
                     /organism="Nectria haematococca mpVI 77-13-4"
                     /isolate="77-13-4"
                     /culture_collection="FGSC:9596"
                     /db_xref="taxon:660122"
                     /chromosome="5"
                     /country="USA"
                     /note="mpVI; mating population VI"
     Protein         1..352
                     /product="predicted protein"
     Region          6..351
                     /region_name="AdhP"
                     /note="Zn-dependent alcohol dehydrogenases [General
                     function prediction only]; COG1064"
                     /db_xref="CDD:31264"
     Region          9..349
                     /region_name="CAD3"
                     /note="Cinnamyl alcohol dehydrogenases (CAD); cd08297"
                     /db_xref="CDD:176257"
     Site            order(46..48,51,156,160,180,182..183,185,203..204,209,
                     250..253,256,273..275,298..300,345)
                     /site_type="other"
                     /note="NAD binding site"
                     /db_xref="CDD:176257"
     Site            order(46,48,57,69,95,156,275,300)
                     /site_type="other"
                     /note="substrate binding site"
                     /db_xref="CDD:176257"
     Site            order(46,69,156)
                     /site_type="other"
                     /note="catalytic Zn binding site"
                     /db_xref="CDD:176257"
     Site            order(57,102,104..105,113..115,171..173,195..197,199,211,
                     214..215,257,265,267,272,274..275,285..286,291..299,
                     303..304,308,312,315)
                     /site_type="other"
                     /note="tetramer interface"
                     /db_xref="CDD:176257"
     Site            order(100,103,106,114)
                     /site_type="other"
                     /note="structural Zn binding site"
                     /db_xref="CDD:176257"
     CDS             1..352
                     /locus_tag="NECHADRAFT_69038"
                     /coded_by="complement(join(GG698905.1:621397..622397,
                     GG698905.1:622599..622656))"
                     /note="GO_function: GO:4024; zinc ion binding  - alcohol
```

```
dehydrogenase activity, zinc-dependent  [PMID 8270]"
/db_xref="InterPro:IPR002085"
/db_xref="JGIDB:Necha2_69038"
```

Mascot: http://www.matrixscience.com/
